# Publisher Correction: Network inference from glycoproteomics data reveals new reactions in the IgG glycosylation pathway

**DOI:** 10.1038/s41467-017-02379-2

**Published:** 2018-02-13

**Authors:** Elisa Benedetti, Maja Pučić-Baković, Toma Keser, Annika Wahl, Antti Hassinen, Jeong-Yeh Yang, Lin Liu, Irena Trbojević-Akmačić, Genadij Razdorov, Jerko Štambuk, Lucija Klarić, Ivo Ugrina, Maurice H. J. Selman, Manfred Wuhrer, Igor Rudan, Ozren Polasek, Caroline Hayward, Harald Grallert, Konstantin Strauch, Annette Peters, Thomas Meitinger, Christian Gieger, Marija Vilaj, Geert-Jan Boons, Kelley W. Moremen, Tatiana Ovchinnikova, Nicolai Bovin, Sakari Kellokumpu, Fabian J. Theis, Gordan Lauc, Jan Krumsiek

**Affiliations:** 1 Institute of Computational Biology, Helmholtz Zentrum München—German Research Center for Environmental Health, 85764 Neuherberg, Germany; 2 Genos Glycoscience Research Laboratory, 10000 Zagreb, Croatia; 3 Faculty of Pharmacy and Biochemistry, University of Zagreb, 10000 Zagreb, Croatia; 4 Institute of Epidemiology 2, Research Unit Molecular Epidemiology, Helmholtz Zentrum München—German Research Center for Environmental Health, 85764 Neuherberg, Germany; 5 Institute of Epidemiology 2, Helmholtz Zentrum München—German Research Center for Environmental Health, 85764 Neuherberg, Germany; 6 Faculty of Biochemistry and Molecular Medicine, University of Oulu, FI-90014 Oulu, Finland; 7 Complex Carbohydrate Research Center, University of Georgia, Athens, GA 30602 USA; 8 Usher Institute of Population Health Sciences and Informatics, University of Edinburgh, EH8 9AG Edinburgh, UK; 9 Medical Research Council Human Genetics Unit, Institute of Genetics and Molecular Medicine, University of Edinburgh, EH4 2XU Edinburgh, UK; 10 Faculty of Science, University of Split, 21000 Split, Croatia; 11 Intellomics Ltd., 10000 Zagreb, Croatia; 12 Leiden University Medical Center, 2333 ZA Leiden, The Netherlands; 13 University of Split School of Medicine, 21000 Split, Croatia; 14 Gen-info Ltd., 10000 Zagreb, Croatia; 15 German Center for Diabetes Research (DZD), 40225 Düsseldorf, Germany; 16 Institute of Genetic Epidemiology, Helmholtz Zentrum München—German Research Center for Environmental Health, 85764 Neuherberg, Germany; 17 Institute of Medical Informatics, Biometry and Epidemiology, Chair of Genetic Epidemiology, Ludwig-Maximilians Universität, 81577 Munich, Germany; 18 Institute of Human Genetics, Helmholtz Zentrum München—German Research Center for Environmental Health, 85764 Neuherberg, Germany; 19 Department of Chemical Biology and Drug Discovery, Utrecht Institute for Pharmaceutical Sciences, and Bijvoet Center for Biomolecular Research, Utrecht University, 3584 CG Utrecht, The Netherlands; 20 Shemyakin and Ovchinnikov Institute of Bioorganic Chemistry, Russian Academy of Sciences, 117997 Moscow, Russia; 21 Department of Mathematics, Technical University Munich, 85748 Garching bei München, Germany

Correction to: *Nature* *Communications* 10.1038/s41467-017-01525-0, published online 14 November 2017

The originally published version of this Article did not acknowledge Fabian J. Theis and Gordan Lauc as corresponding authors. Furthermore, the author Jan Krumsiek was incorrectly listed as being affiliated with institutions 1 and 14, and should have been affiliated with institutions 1 and 15. These errors have now been corrected in both the PDF and HTML versions of the manuscript.

