# Supplementary material for: Network inference from glycoproteomics data reveals new reactions in the IgG glycosylation pathway
Source: Nat Commun. 2017 Nov 14;8:1483. doi: 10.1038/s41467-017-01525-0 (PMC5684356; doi:10.1038/s41467-017-01525-0)
Supplement: Supplementary file 1 — Supplementary Information [file 41467_2017_1525_MOESM1_ESM.pdf]

Supplementary Figures

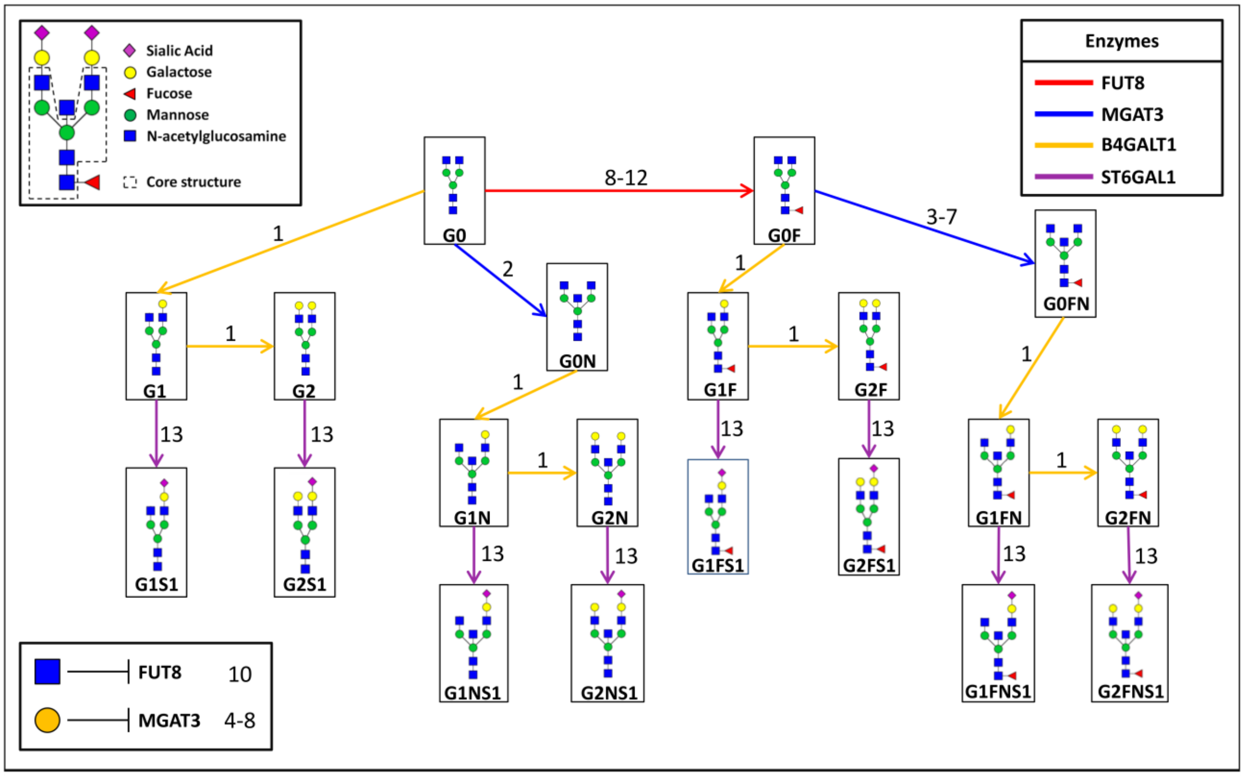

Supplementary Figure 1. Primary Literature for the determination of the known IgG glycosylation pathway.

For each reaction in the known IgG glycosylation pathway, the relevant literature is indicated [1–13].

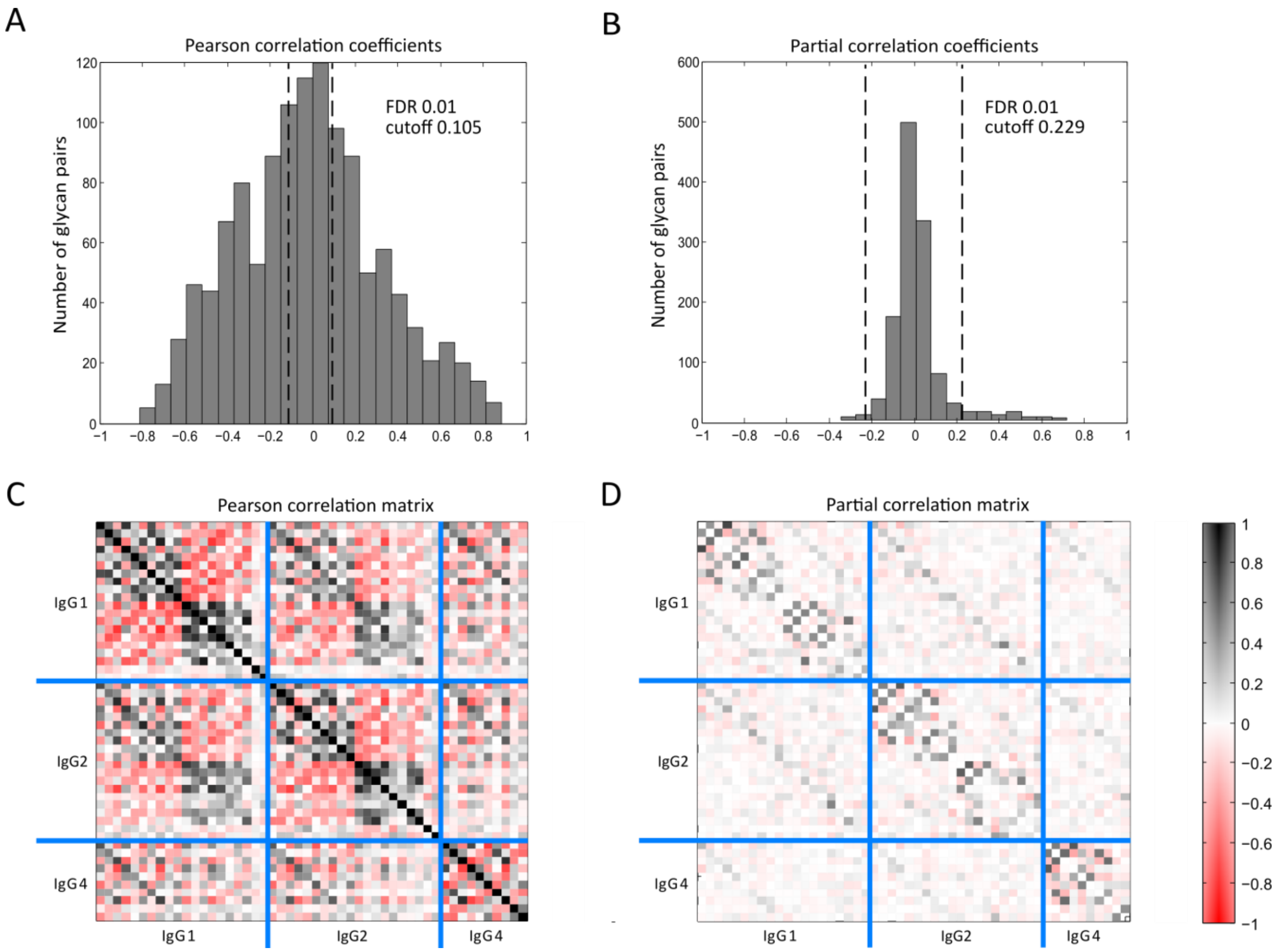

Supplementary Figure 2. Pearson and partial correlations of IgG glycans.

A, B Histograms of all pairwise Pearson and partial correlation coefficients, respectively. Black dashed lines indicate the significance cutoff (FDR = 0.01). The Pearson correlation matrix contains a large number of significant coefficients, which are evenly distributed around zero. Partial correlations are generally lower, and a much smaller proportion was statistically significant. Moreover, most significant partial correlations were positive.

C, D Pearson and partial correlation matrices, respectively. Black and red indicate positive and negative coefficients, respectively. Blue lines separate the different IgG subclasses. The ordering of the glycans is the same for all subclasses. The stronger signal around the inter-subclass diagonals represents connections between glycans with the same structure in different IgG subclasses.

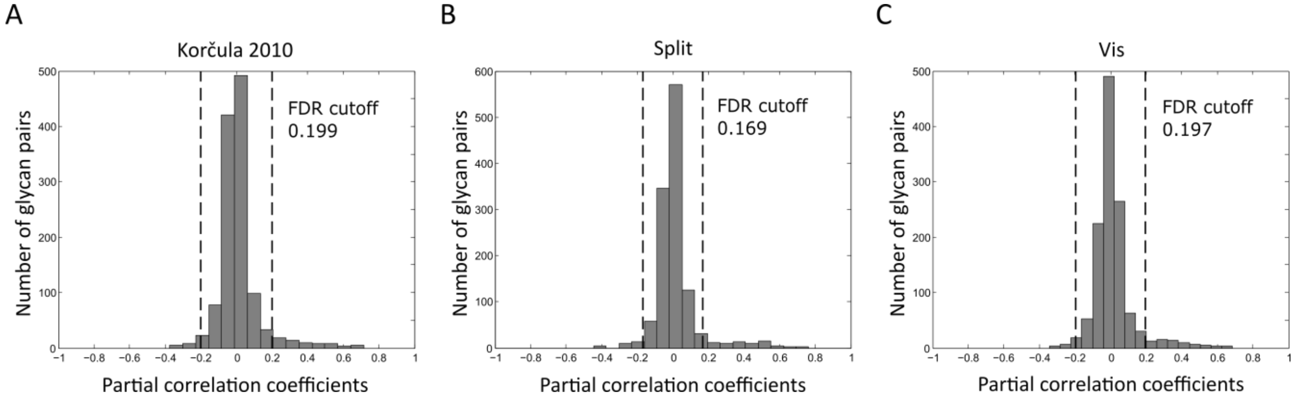

Supplementary Figure 3. Distributions of partial correlation coefficients in the replication cohorts.

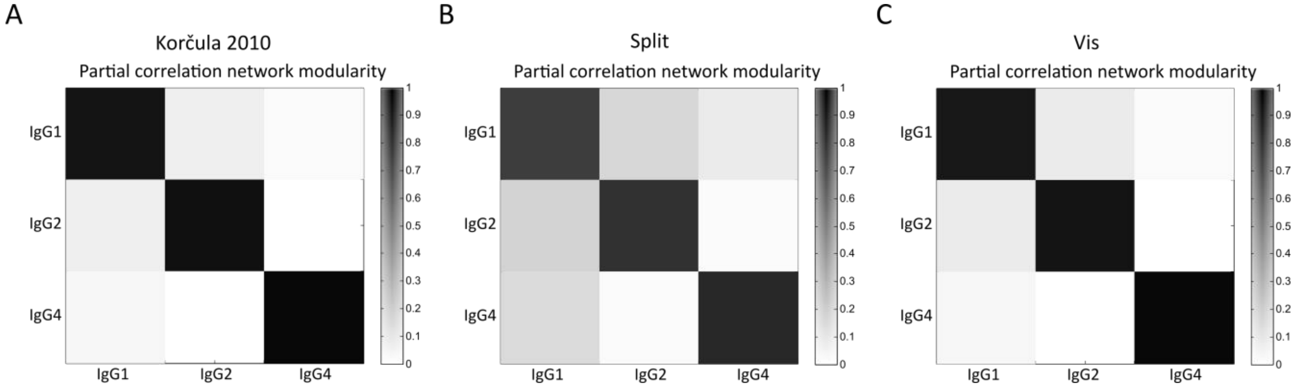

Supplementary Figure 4. Network modularity of the partial correlation coefficients in the replication cohorts.

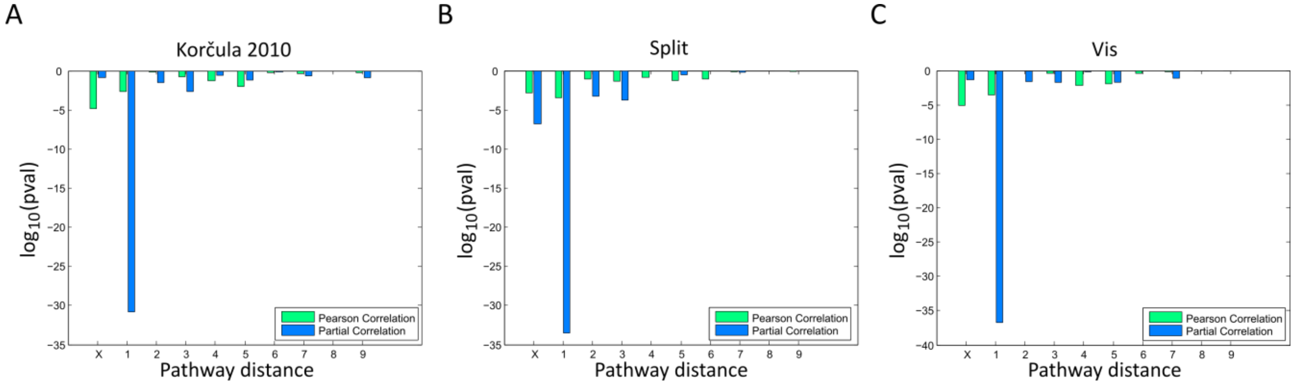

Supplementary Figure 5. Pathway analysis in the replication cohorts.

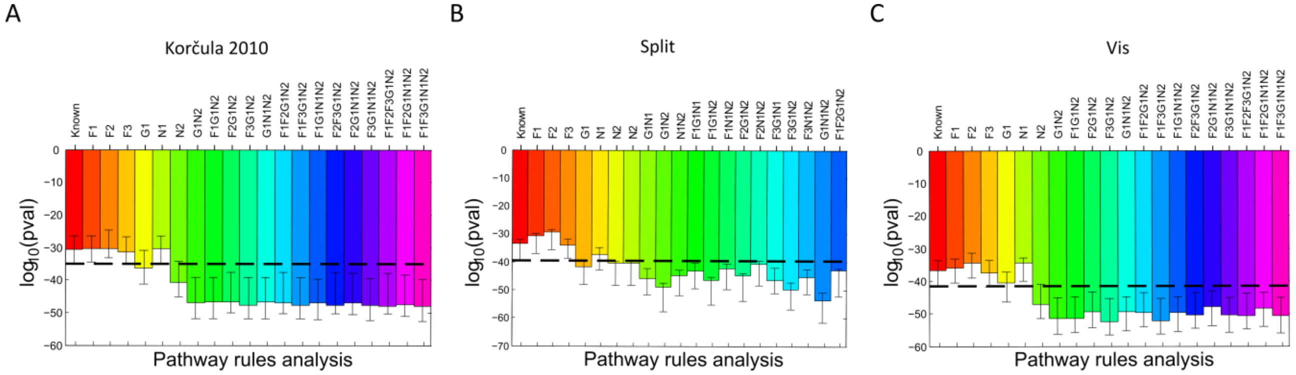

Supplementary Figure 6. Rule-based pathway inference in the replication cohorts.

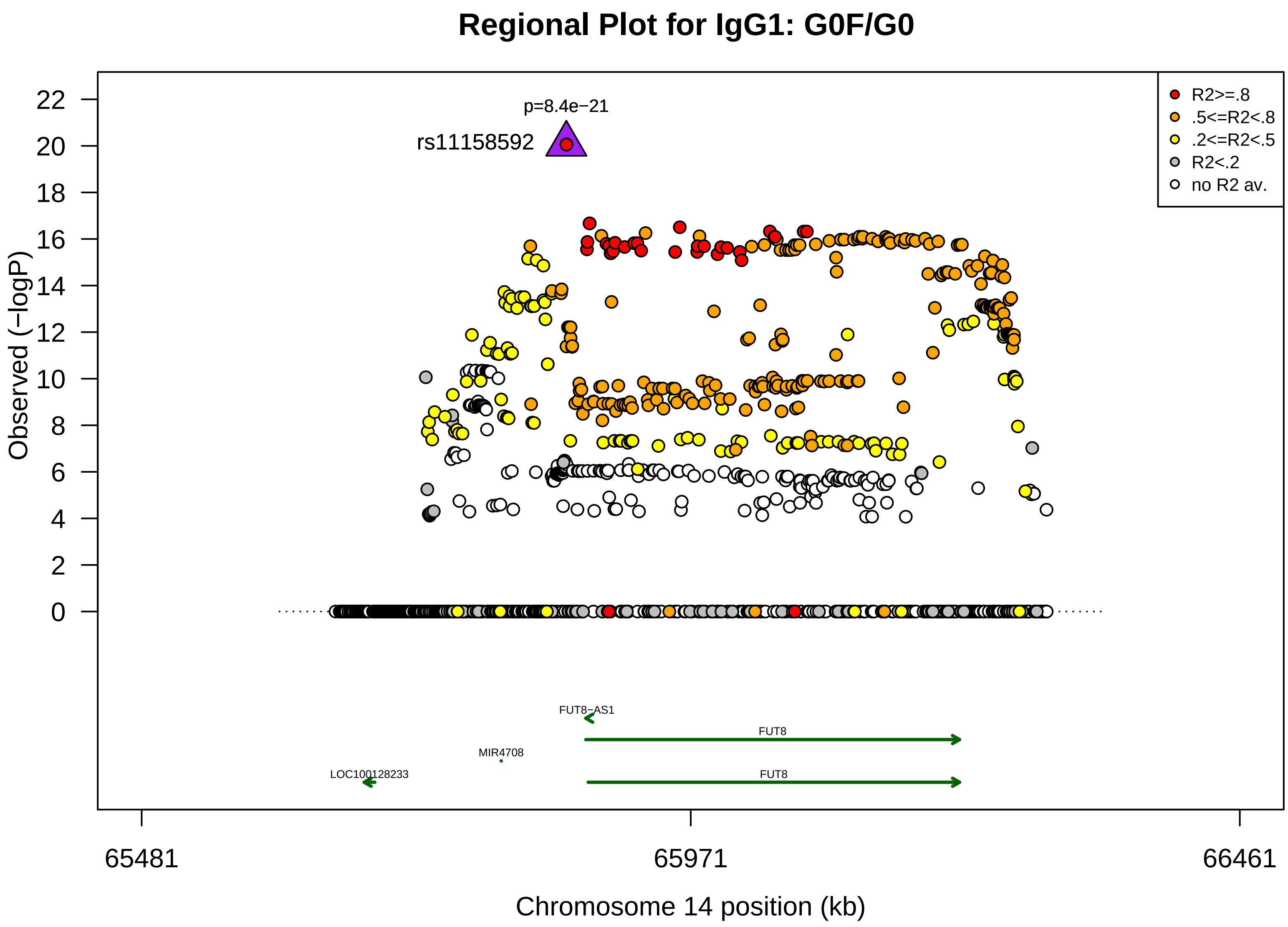
Regional Plot for IgG1: G0F/G0
p=8.4e−21
rs11158592
R2>=.8
.5<=R2<.8
.2<=R2<.5
R2<.2
no R2 av.
Observed (−logP)
0
2
4
6
8
10
12
14
16
18
20
22
FUT8−AS1
FUT8
MIR4708
LOC100128233
FUT8
65481
65971
66461
Chromosome 14 position (kb)

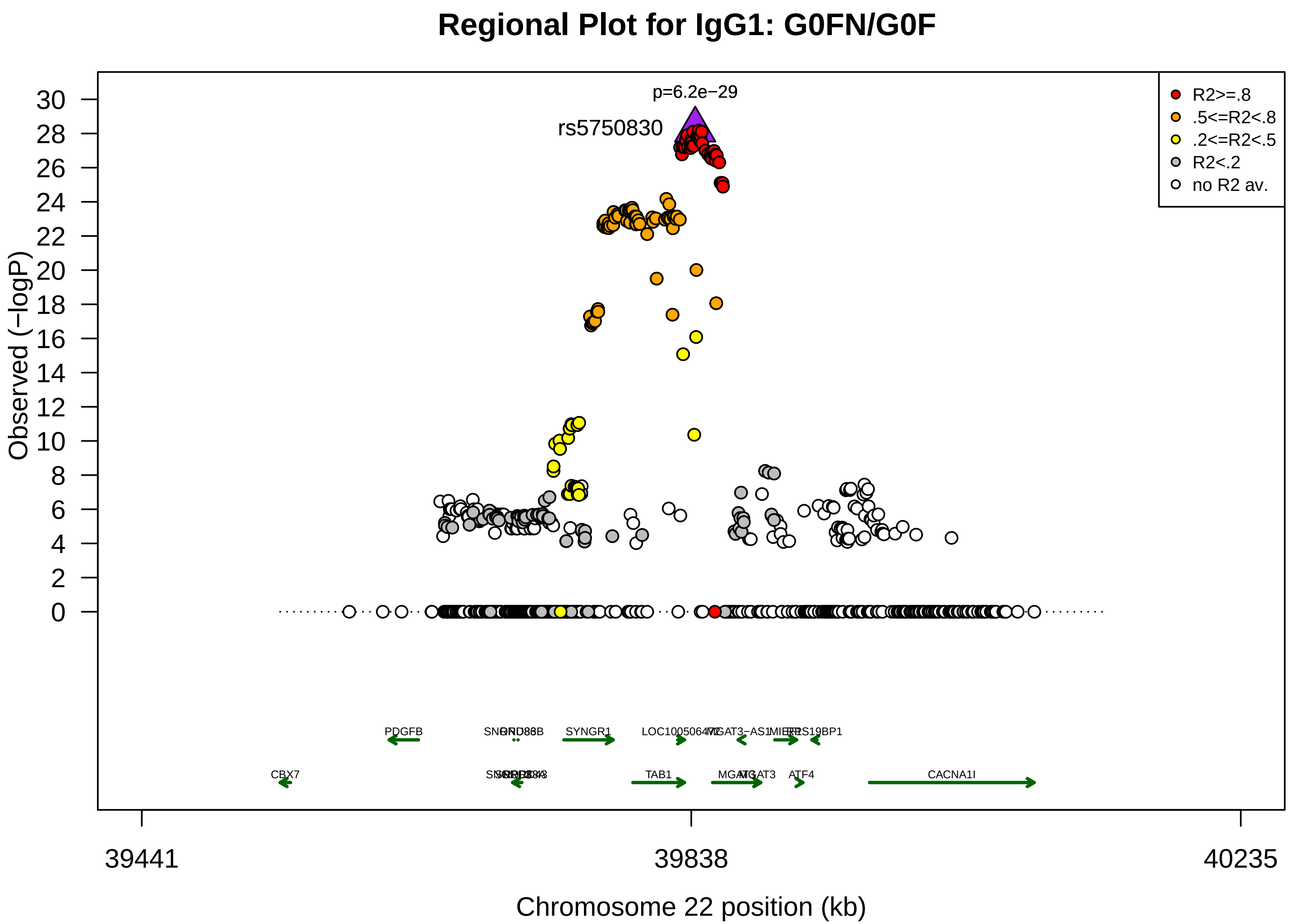
Regional Plot for IgG1: G0FN/G0F
p=6.2e−29
rs5750830
R2>=.8
.5<=R2<.8
.2<=R2<.5
R2<.2
no R2 av.
Observed (−logP)
Chromosome 22 position (kb)
39441
39838
40235
PDGFB
SYNGR1
CBX7
TAB1
MGAT3
ATF4
CACNA1I

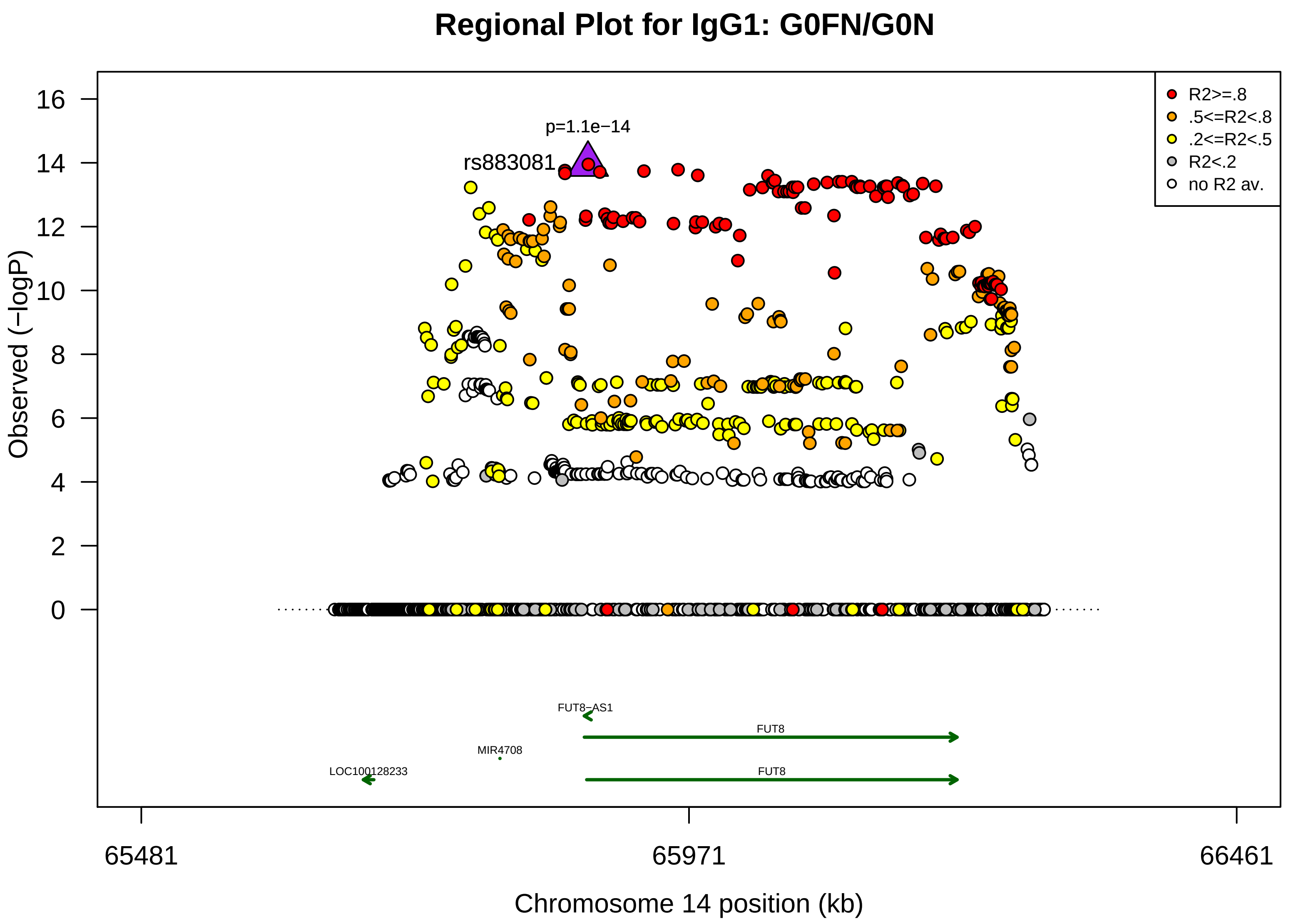

Regional Plot for IgG1: G0FN/G0N
R2>=.8
.5<=R2<.8
.2<=R2<.5
R2<.2
no R2 av.
p=1.1e−14
rs883081
16
14
12
10
8
6
4
2
0
Observed (−logP)
FUT8−AS1
FUT8
MIR4708
LOC100128233
FUT8
65481
65971
66461
Chromosome 14 position (kb)

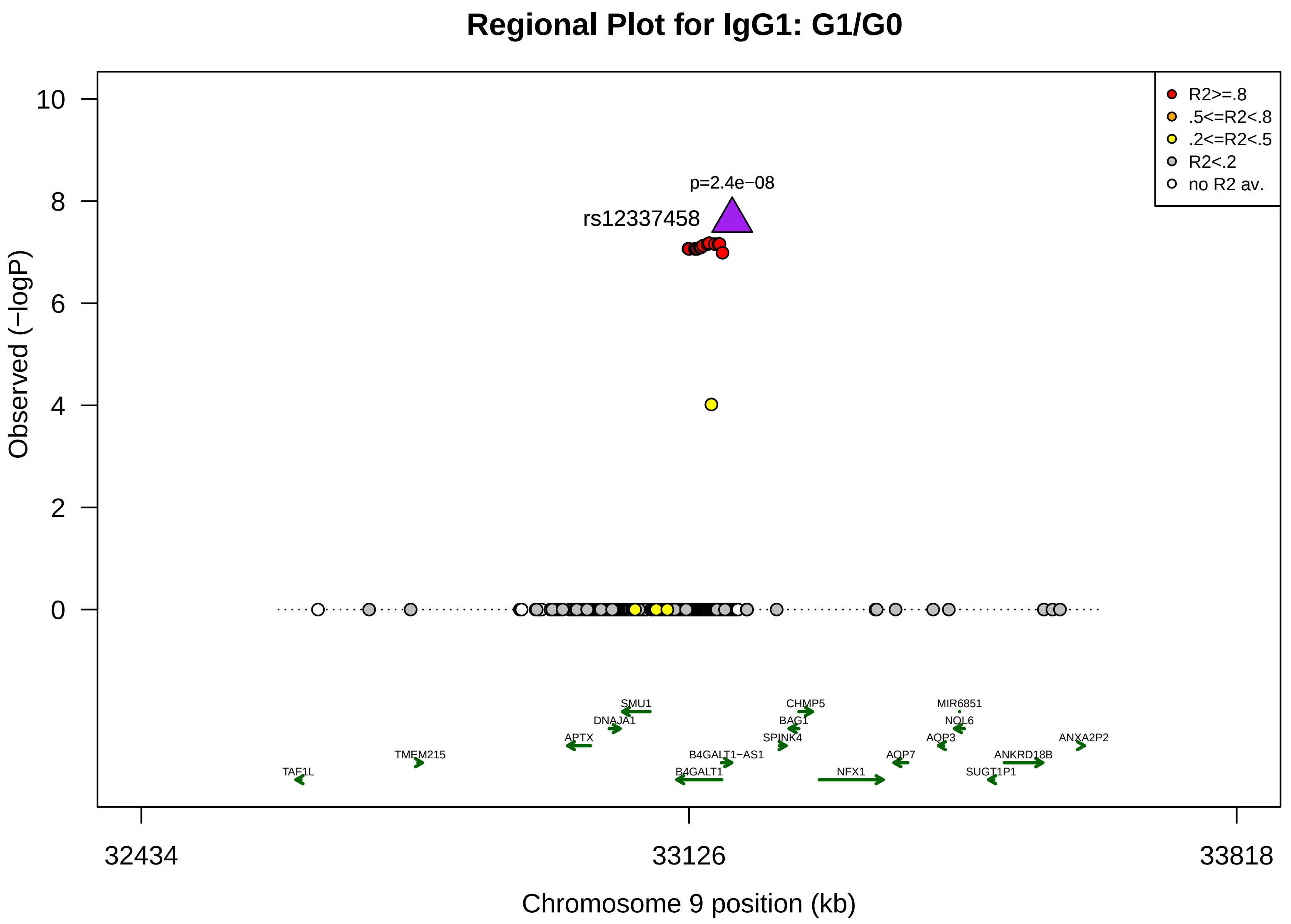
Regional Plot for IgG1: G1/G0
Observed (−logP)
Chromosome 9 position (kb)
32434
33126
33818
0
2
4
6
8
10
R2>=.8
.5<=R2<.8
.2<=R2<.5
R2<.2
no R2 av.
p=2.4e−08
rs12337458
SMU1
DNAJA1
APTX
TMEM215
TAF1L
B4GALT1−AS1
B4GALT1
CHMP5
BAG1
SPINK4
NFX1
AQP7
MIR6851
NOL6
AQP3
ANKRD18B
SUGT1P1
ANXA2P2

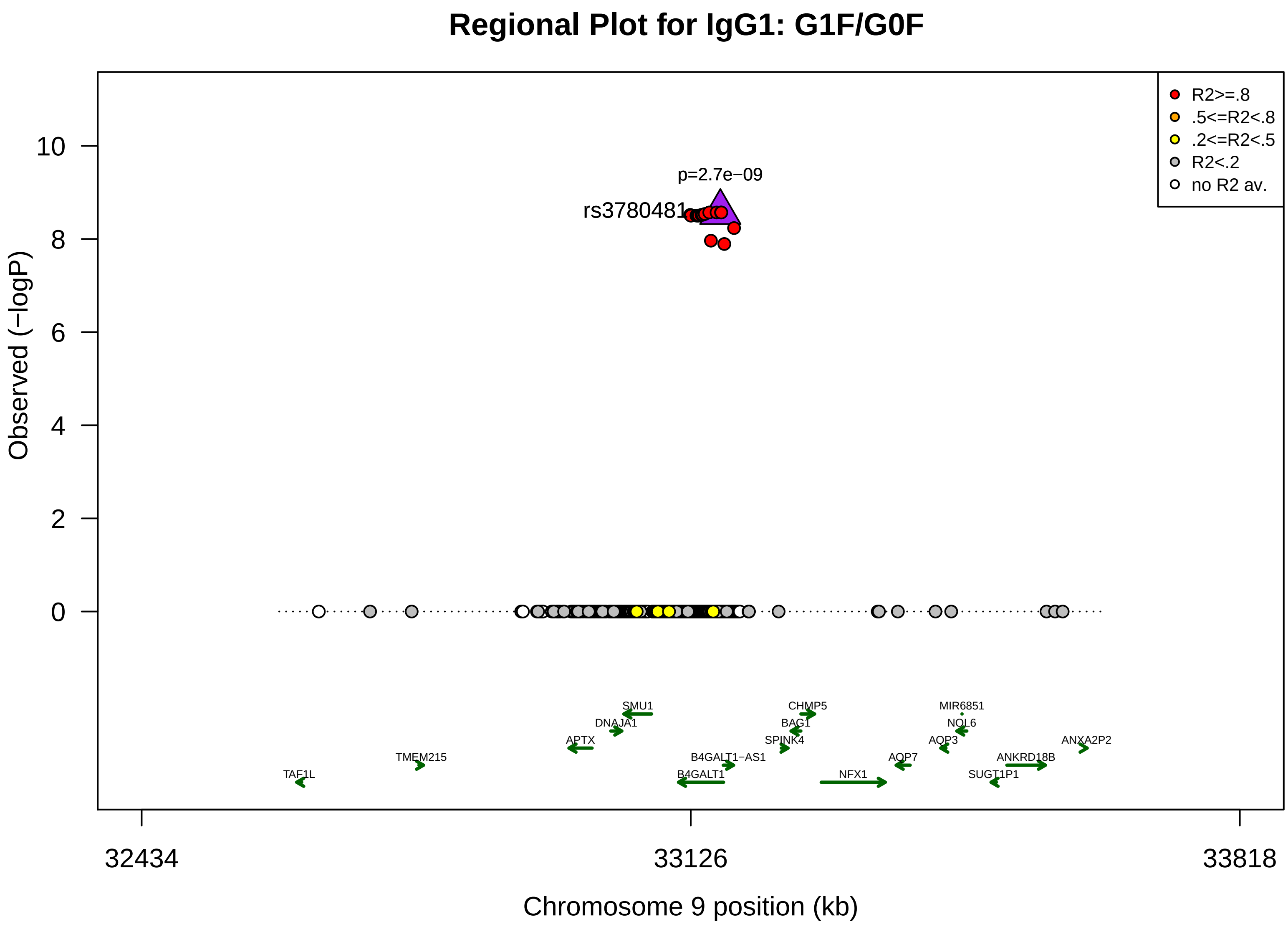

Regional Plot for IgG1: G1F/G0F
R2>=.8
.5<=R2<.8
.2<=R2<.5
R2<.2
no R2 av.
p=2.7e−09
rs3780481
Observed (−logP)
10
8
6
4
2
0
SMU1
CHMP5
MIR6851
DNAJA1
BAG1
NOL6
APTX
SPINK4
AQP3
ANXA2P2
TMEM215
B4GALT1−AS1
AQP7
ANKRD18B
TAF1L
B4GALT1
NFX1
SUGT1P1
32434
33126
33818
Chromosome 9 position (kb)

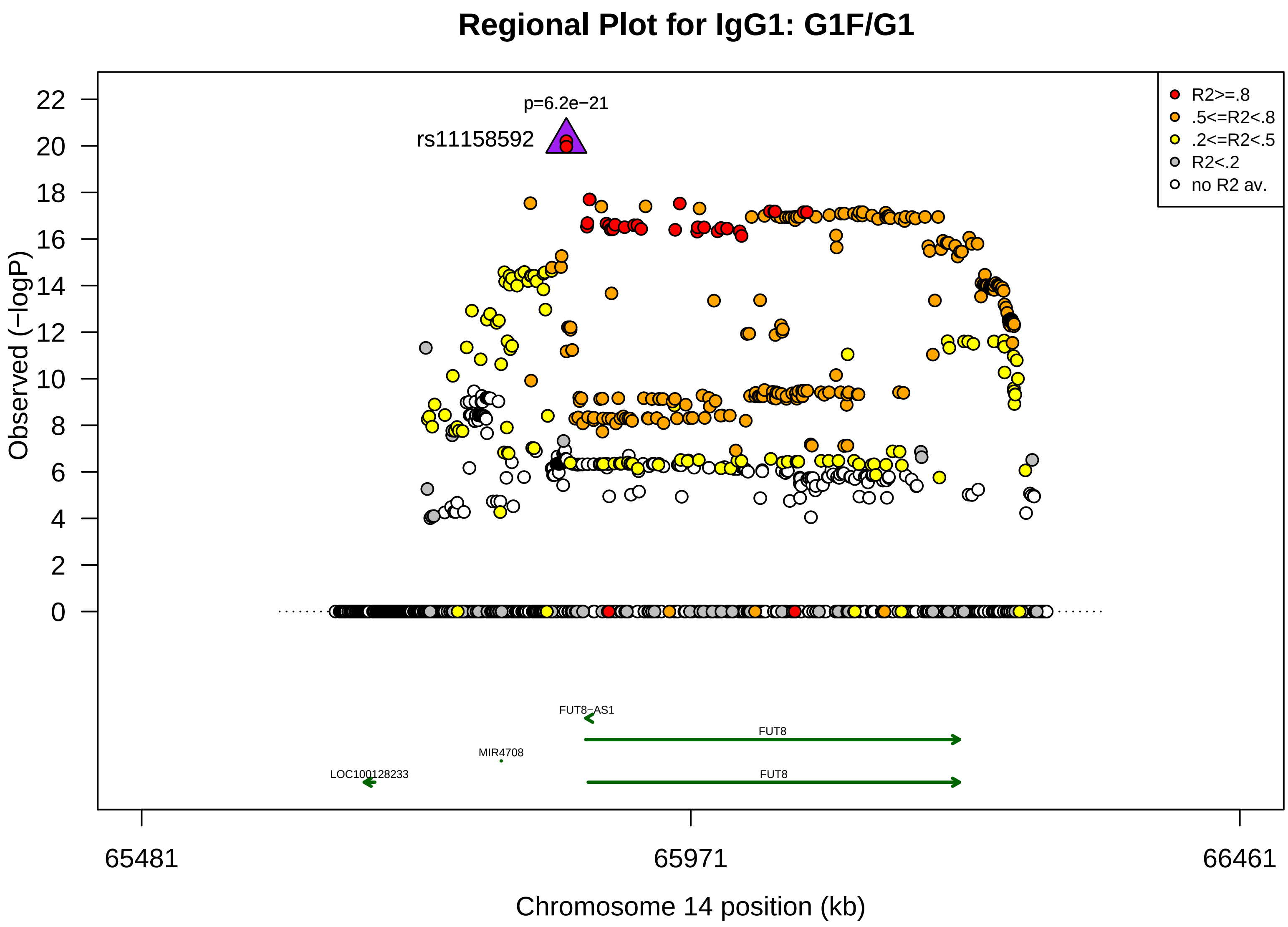

Regional Plot for IgG1: G1F/G1
p=6.2e−21
rs11158592
R2>=.8
.5<=R2<.8
.2<=R2<.5
R2<.2
no R2 av.
Observed (−logP)
22
20
18
16
14
12
10
8
6
4
2
0
FUT8−AS1
FUT8
MIR4708
LOC100128233
FUT8
65481
65971
66461
Chromosome 14 position (kb)

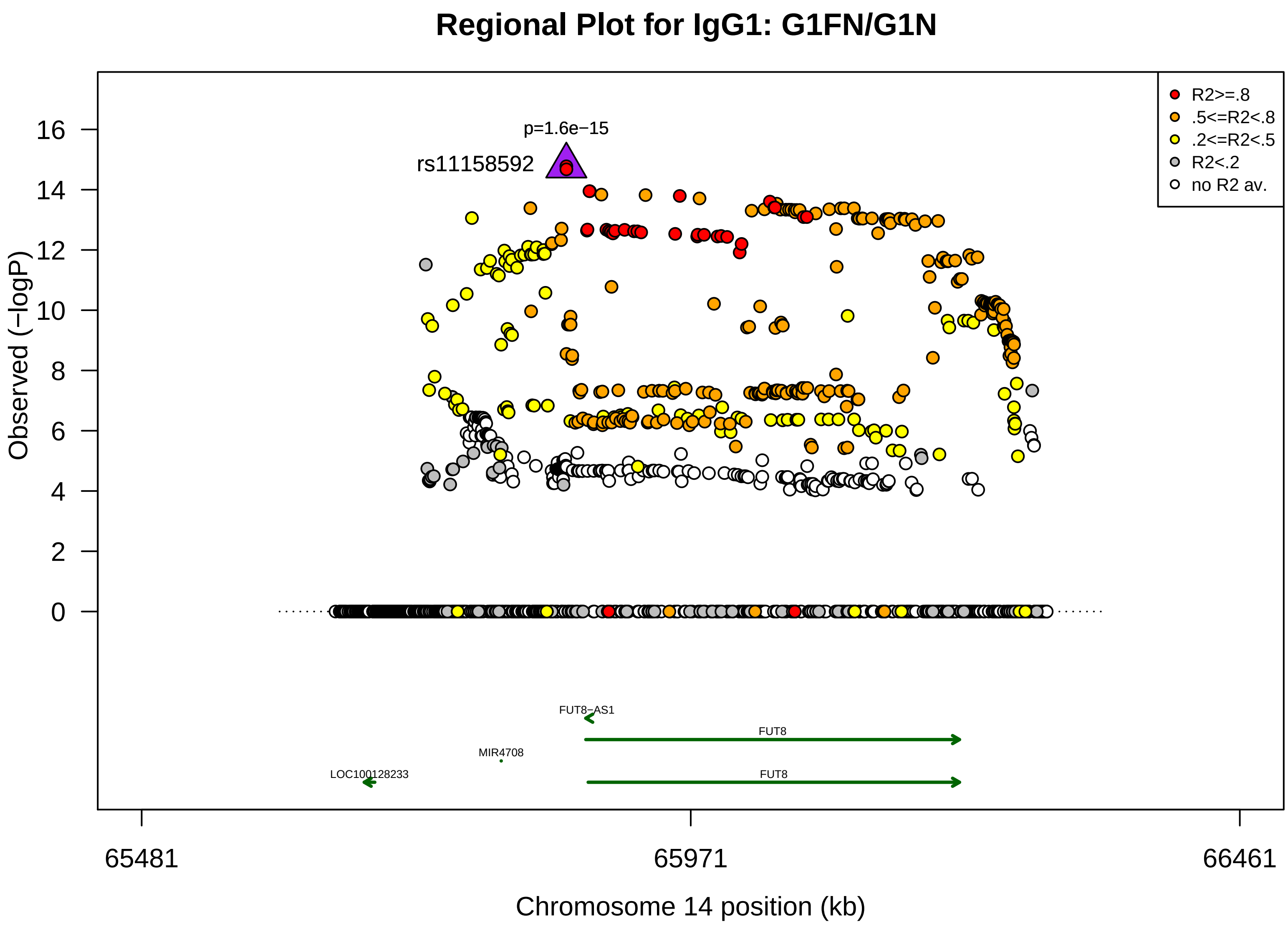

Regional Plot for IgG1: G1FN/G1N
p=1.6e−15
rs11158592
R2>=.8
.5<=R2<.8
.2<=R2<.5
R2<.2
no R2 av.
Observed (−logP)
0
2
4
6
8
10
12
14
16
FUT8−AS1
FUT8
MIR4708
LOC100128233
FUT8
65481
65971
66461
Chromosome 14 position (kb)

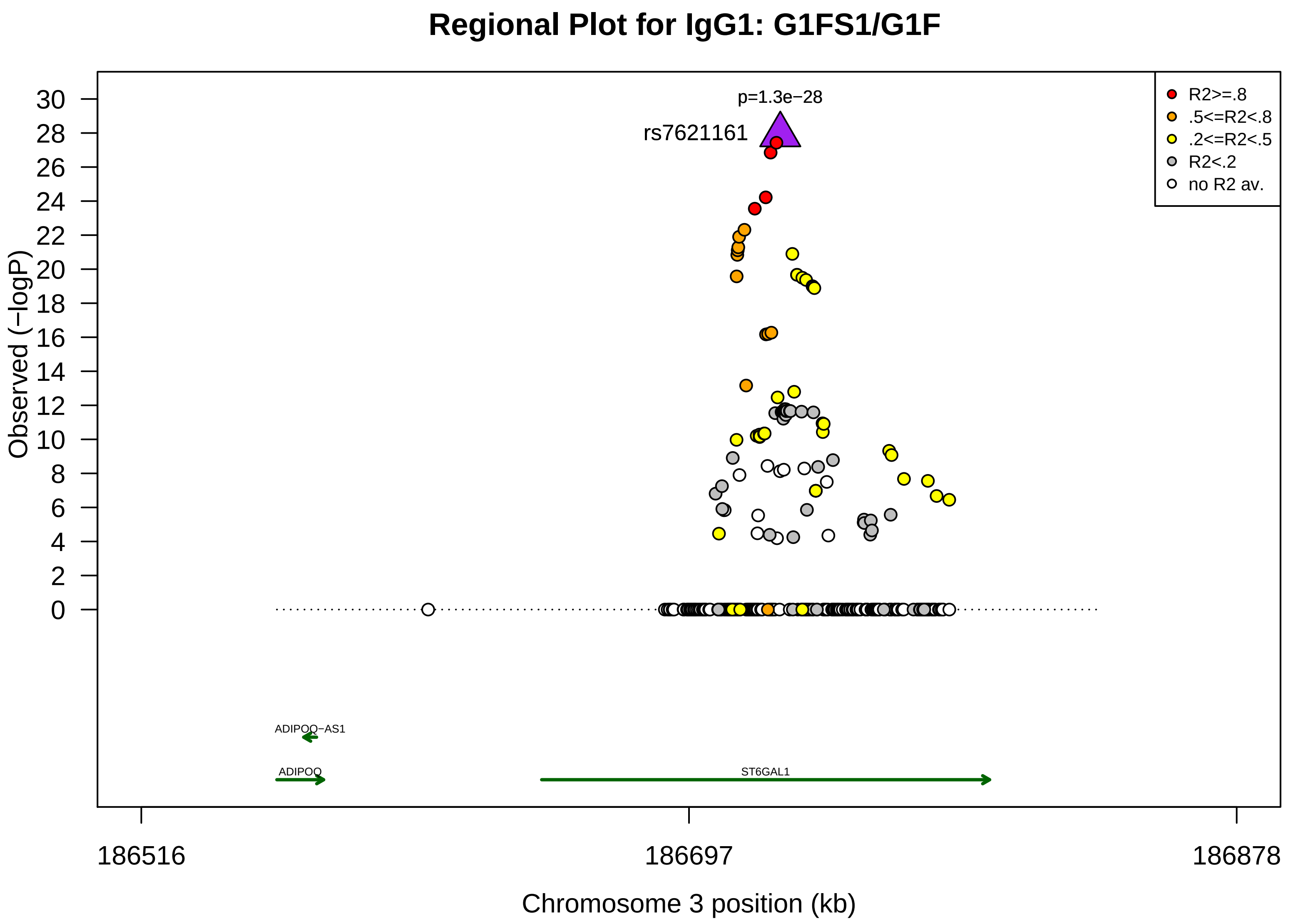
Regional Plot for IgG1: G1FS1/G1F
p=1.3e−28
rs7621161
R2>=.8
.5<=R2<.8
.2<=R2<.5
R2<.2
no R2 av.
Observed (−logP)
30
28
26
24
22
20
18
16
14
12
10
8
6
4
2
0
ADIPOQ−AS1
ADIPOQ
ST6GAL1
186516
186697
186878
Chromosome 3 position (kb)

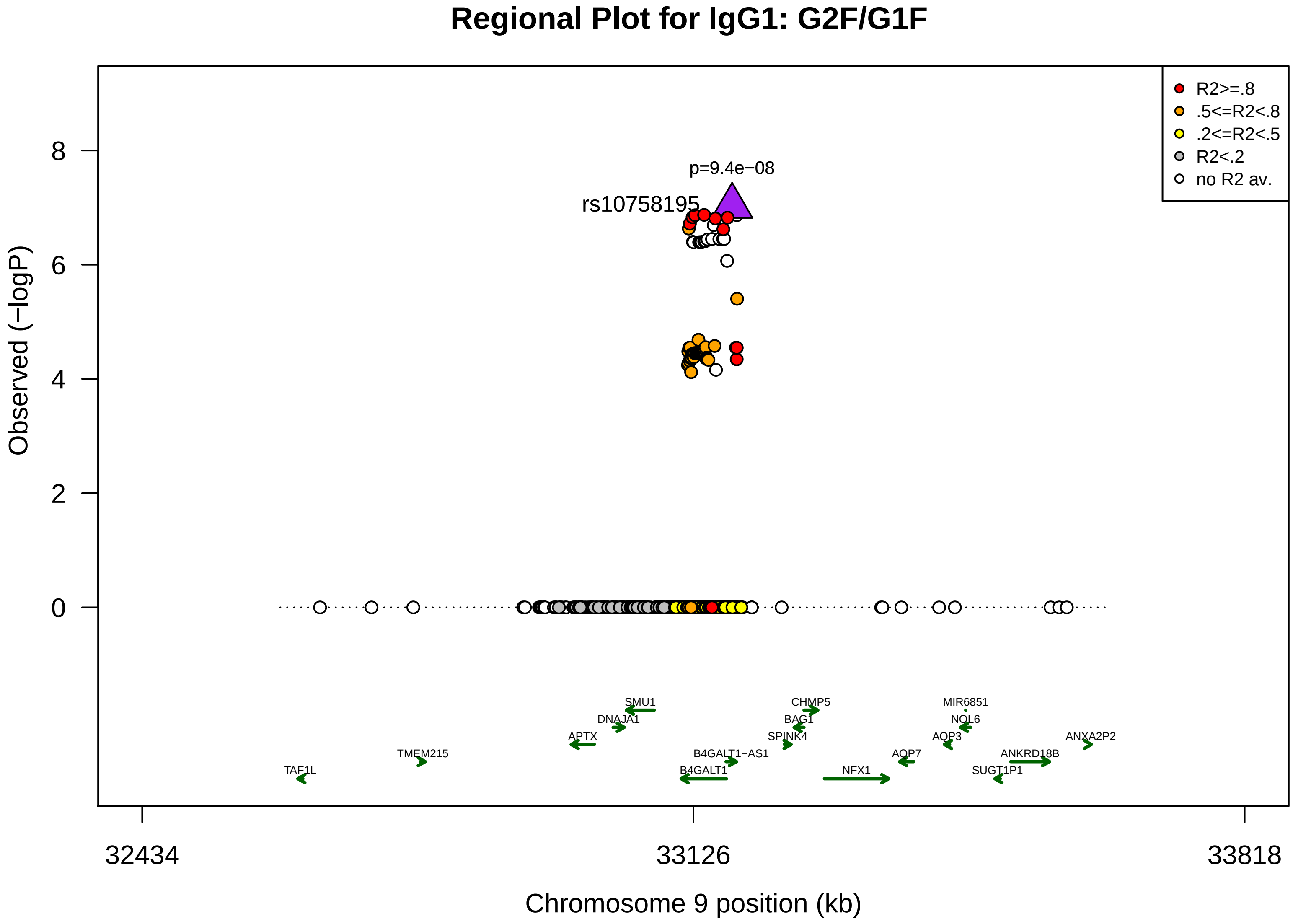

Regional Plot for IgG1: G2F/G1F
Observed (−logP)
0
2
4
6
8
p=9.4e−08
rs10758195
R2>=.8
.5<=R2<.8
.2<=R2<.5
R2<.2
no R2 av.
SMU1
CHMP5
MIR6851
DNAJA1
BAG1
NOL6
APTX
SPINK4
AQP3
ANXA2P2
TMEM215
B4GALT1−AS1
AQP7
ANKRD18B
TAF1L
B4GALT1
NFX1
SUGT1P1
32434
33126
33818
Chromosome 9 position (kb)

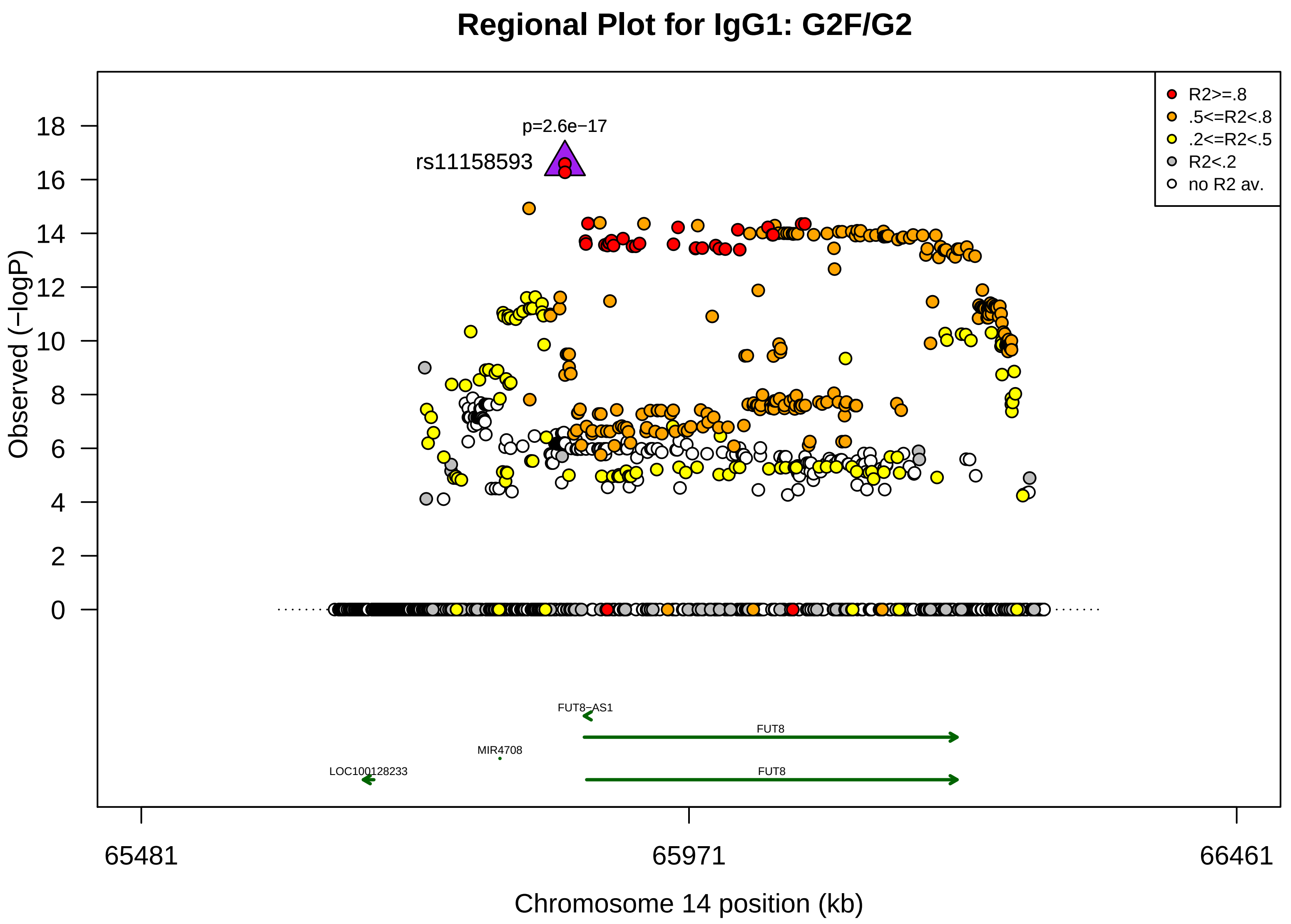

Regional Plot for IgG1: G2F/G2
p=2.6e−17
rs11158593
R2>=.8
.5<=R2<.8
.2<=R2<.5
R2<.2
no R2 av.
Observed (−logP)
0
2
4
6
8
10
12
14
16
18
FUT8−AS1
FUT8
MIR4708
LOC100128233
FUT8
65481
65971
66461
Chromosome 14 position (kb)

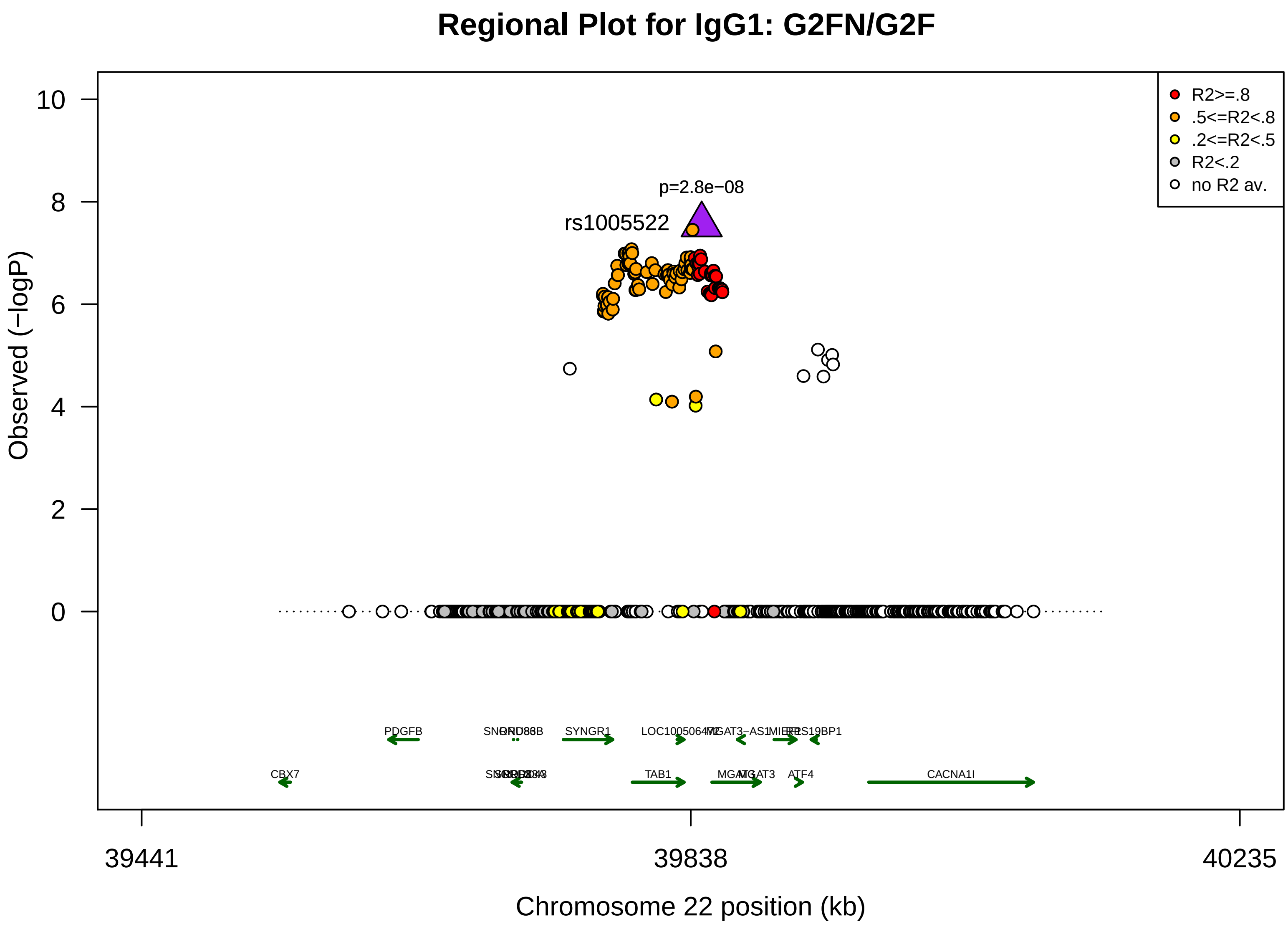

Regional Plot for IgG1: G2FN/G2F
Observed (−logP)
10
8
6
4
2
0
p=2.8e−08
rs1005522
R2>=.8
.5<=R2<.8
.2<=R2<.5
R2<.2
no R2 av.
PDGFB
SYNGR1
CBX7
TAB1
ATF4
CACNA1I
39441
39838
40235
Chromosome 22 position (kb)

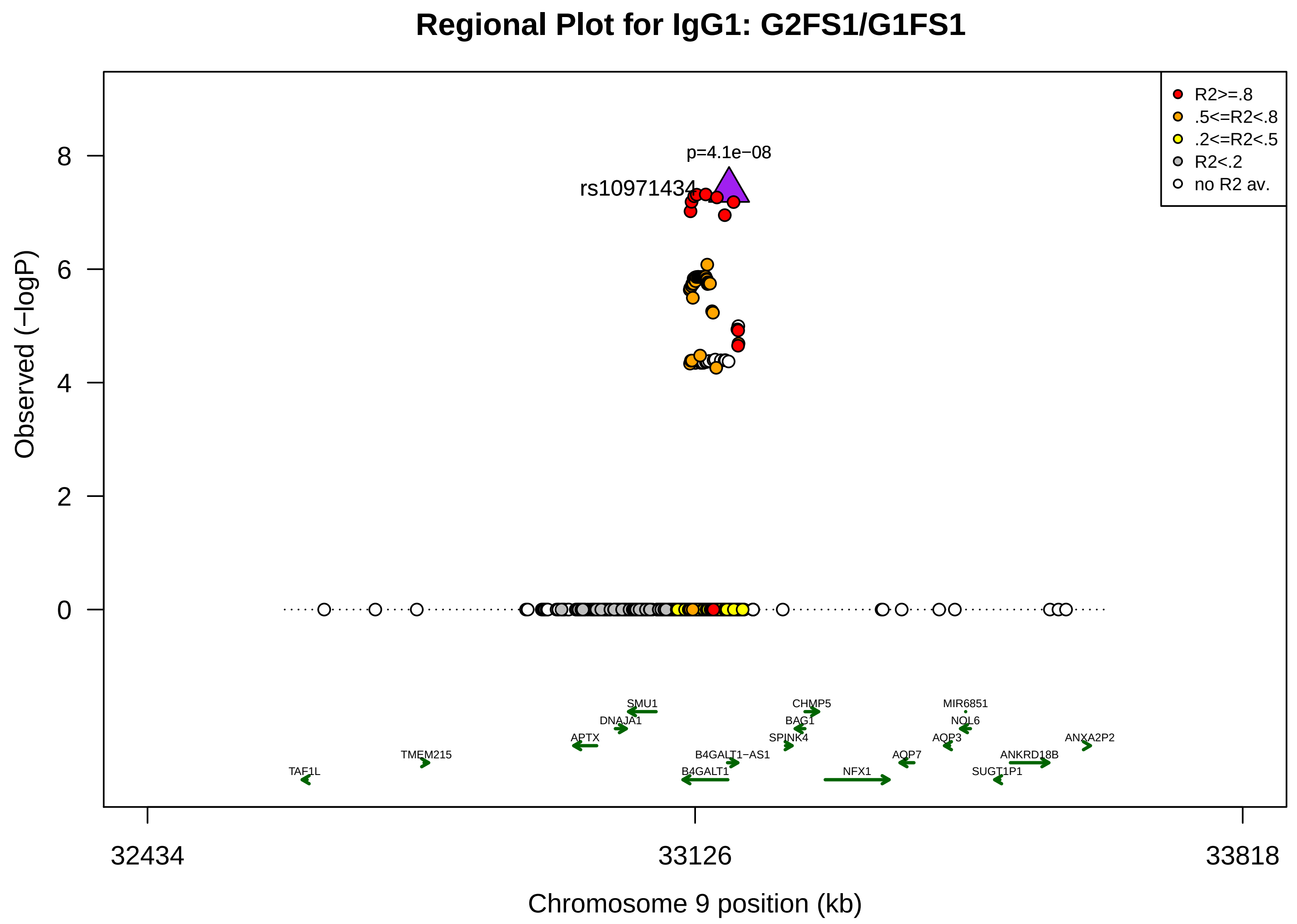
Regional Plot for IgG1: G2FS1/G1FS1
Observed (−logP)
Chromosome 9 position (kb)
32434
33126
33818
0
2
4
6
8
p=4.1e−08
rs10971434
R2>=.8
.5<=R2<.8
.2<=R2<.5
R2<.2
no R2 av.
SMU1
DNAJA1
APTX
TMEM215
TAF1L
B4GALT1−AS1
B4GALT1
CHMP5
BAG1
SPINK4
NFX1
AQP7
AQP3
MIR6851
NOL6
ANKRD18B
SUGT1P1
ANXA2P2

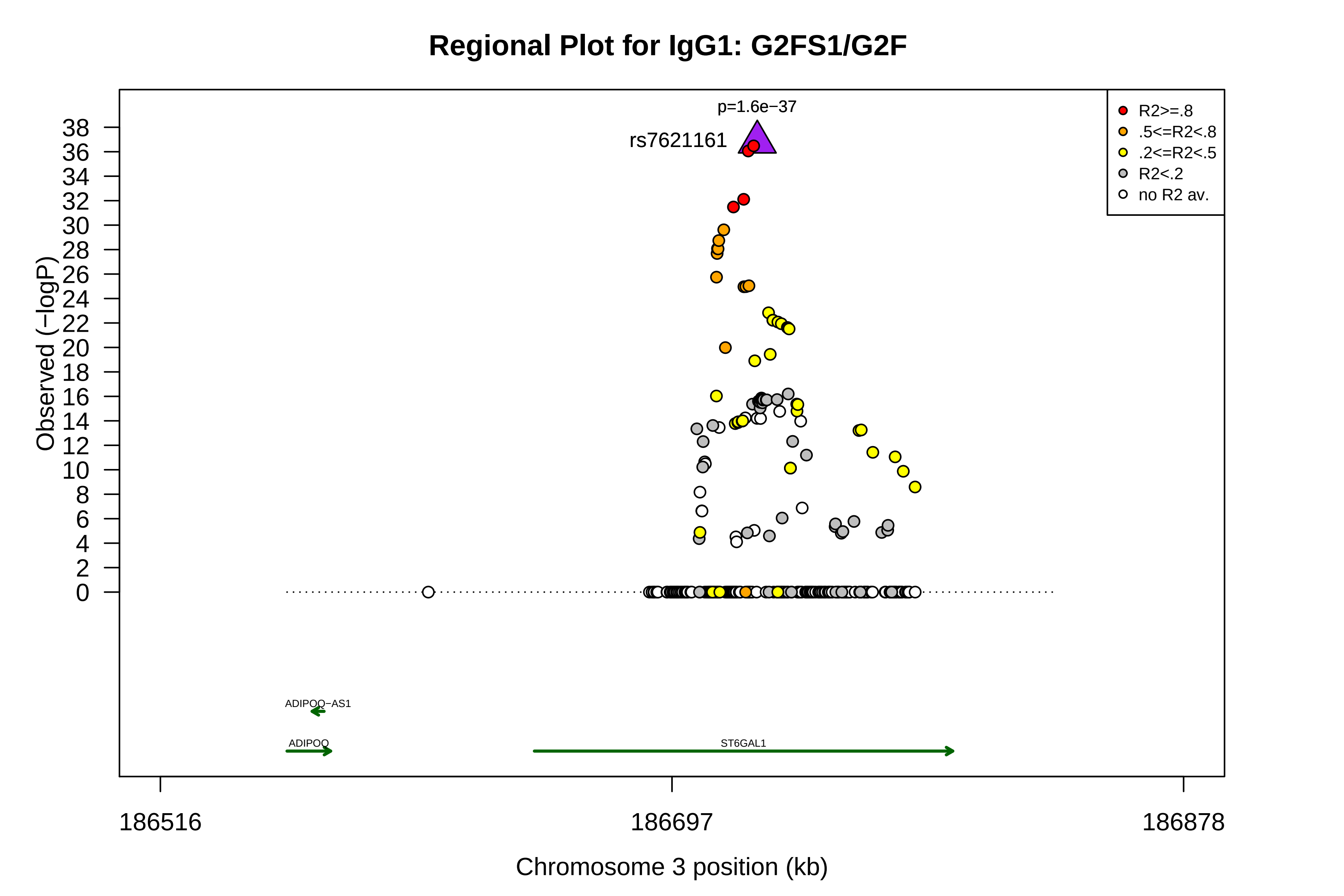
Regional Plot for IgG1: G2FS1/G2F
p=1.6e−37
rs7621161
R2>=.8
.5<=R2<.8
.2<=R2<.5
R2<.2
no R2 av.
Observed (−logP)
0
2
4
6
8
10
12
14
16
18
20
22
24
26
28
30
32
34
36
38
ADIPOQ−AS1
ADIPOQ
ST6GAL1
186516
186697
186878
Chromosome 3 position (kb)

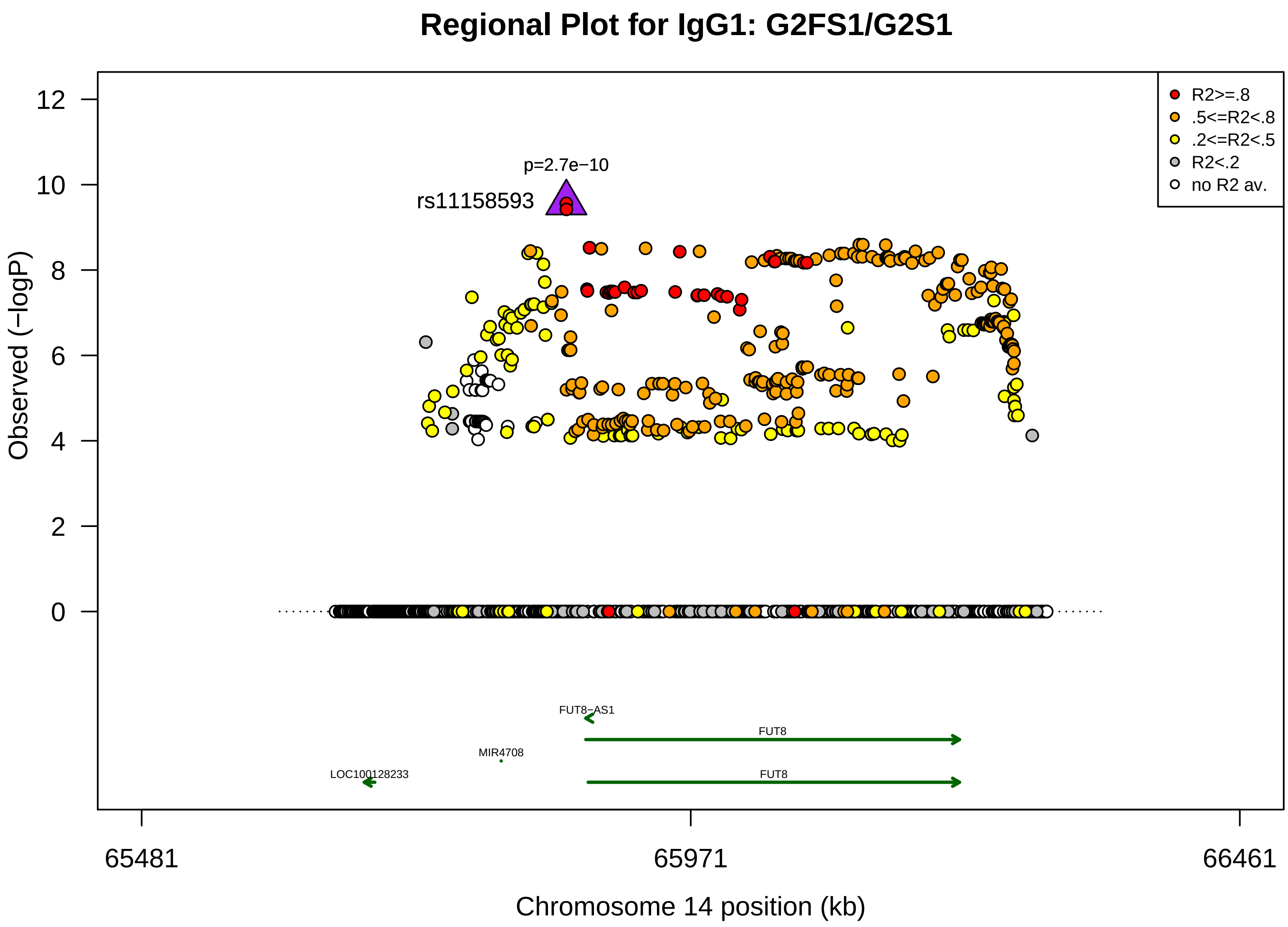

Regional Plot for IgG1: G2FS1/G2S1
R2>=.8
.5<=R2<.8
.2<=R2<.5
R2<.2
no R2 av.
p=2.7e−10
rs11158593
Observed (−logP)
0
2
4
6
8
10
12
FUT8−AS1
FUT8
MIR4708
LOC100128233
FUT8
65481
65971
66461
Chromosome 14 position (kb)

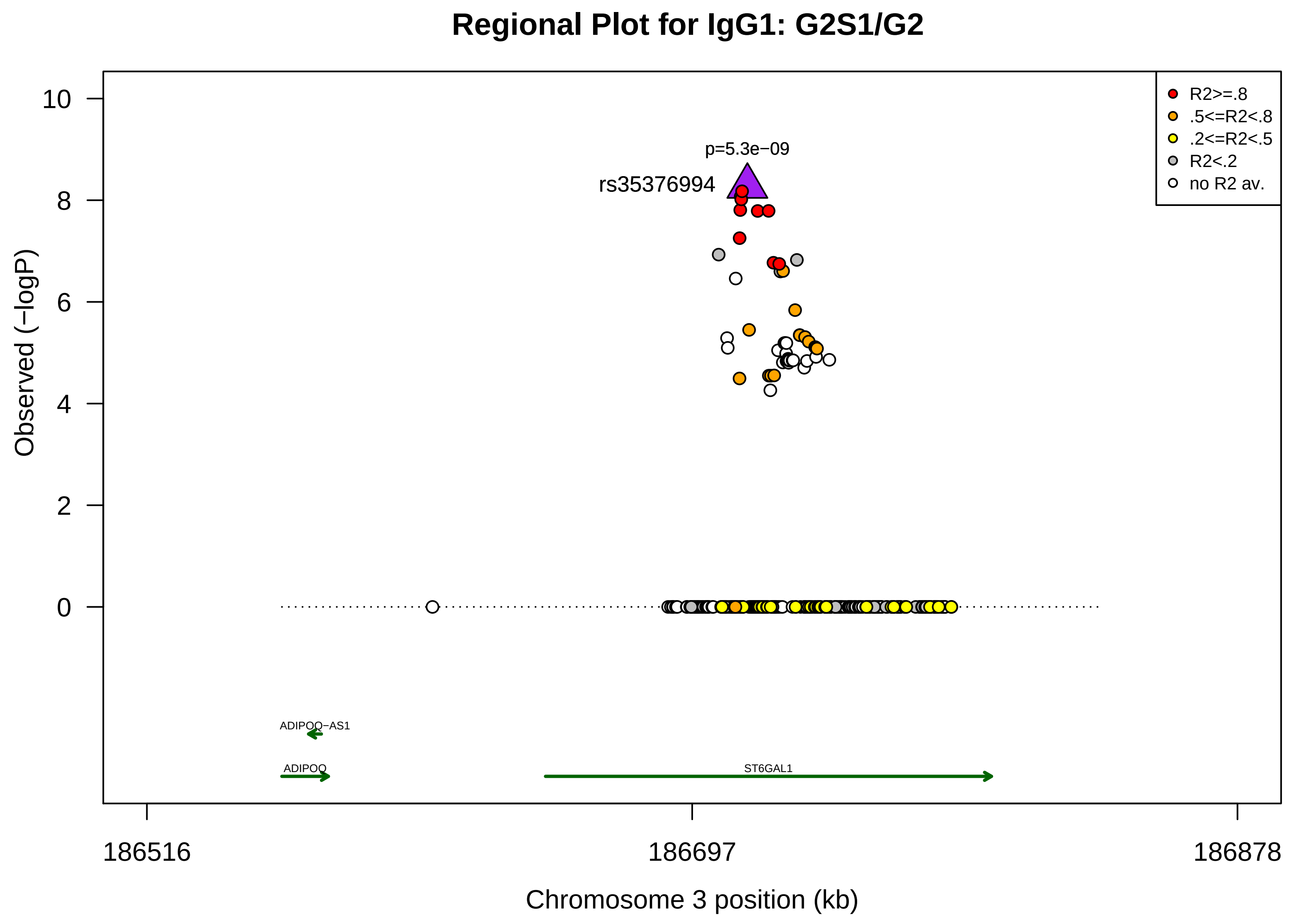

Regional Plot for IgG1: G2S1/G2
R2>=.8
.5<=R2<.8
.2<=R2<.5
R2<.2
no R2 av.
p=5.3e−09
rs35376994
Observed (−logP)
10
8
6
4
2
0
ADIPOQ−AS1
ADIPOQ
ST6GAL1
186516
186697
186878
Chromosome 3 position (kb)

Regional Plot for IgG2: G0FN/G0F

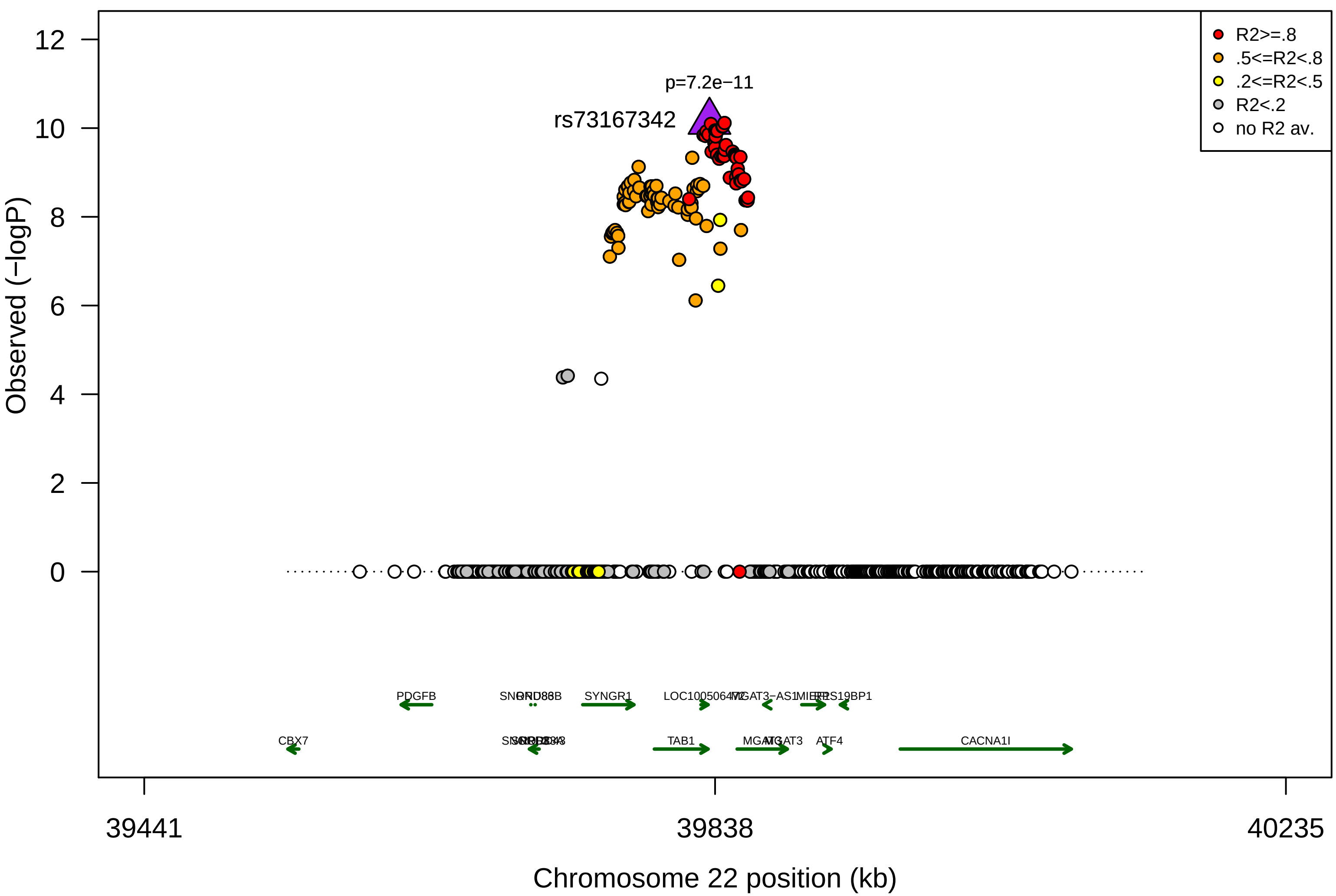

Regional Plot for IgG2: G1FN/G0FN

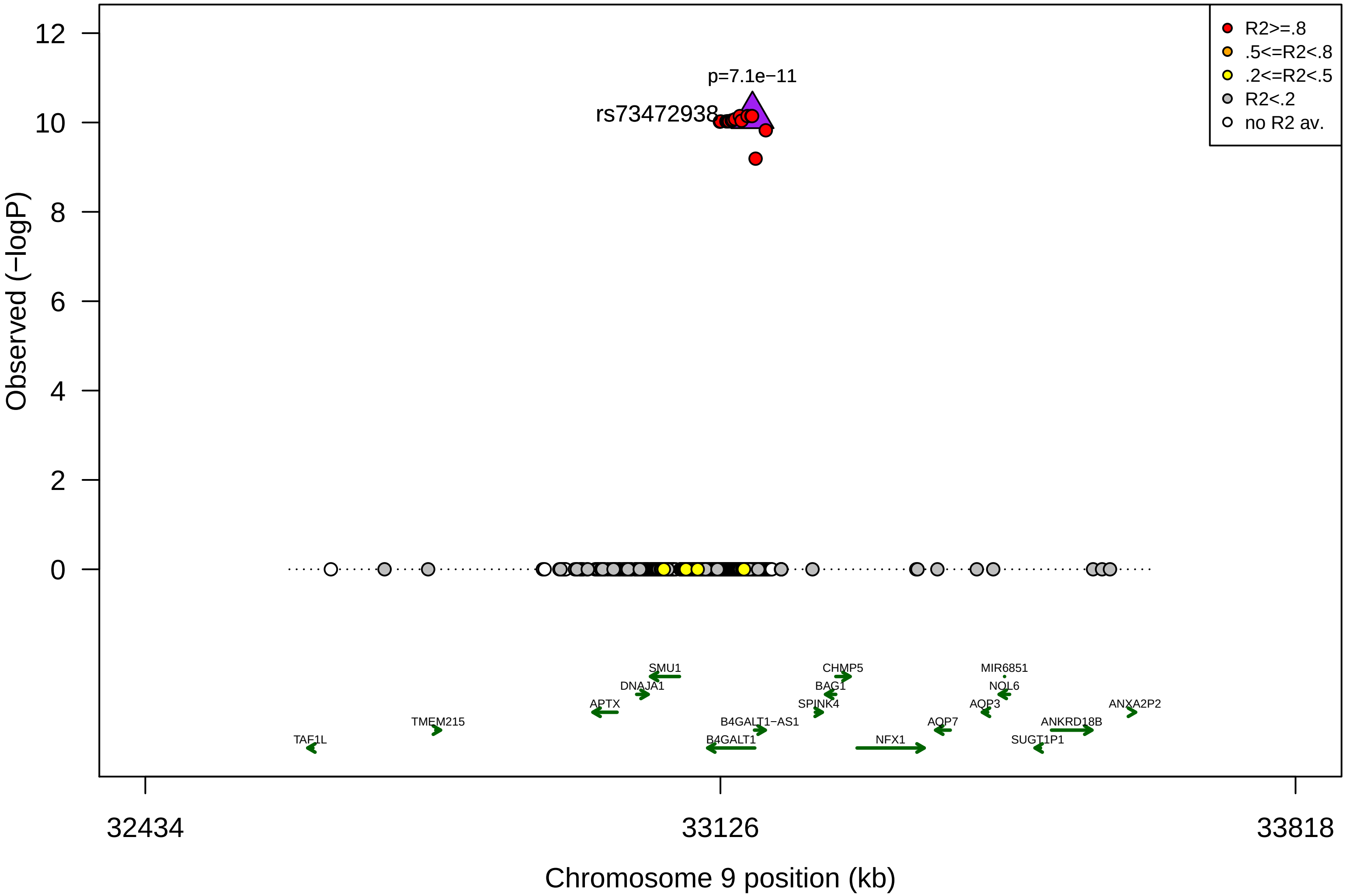

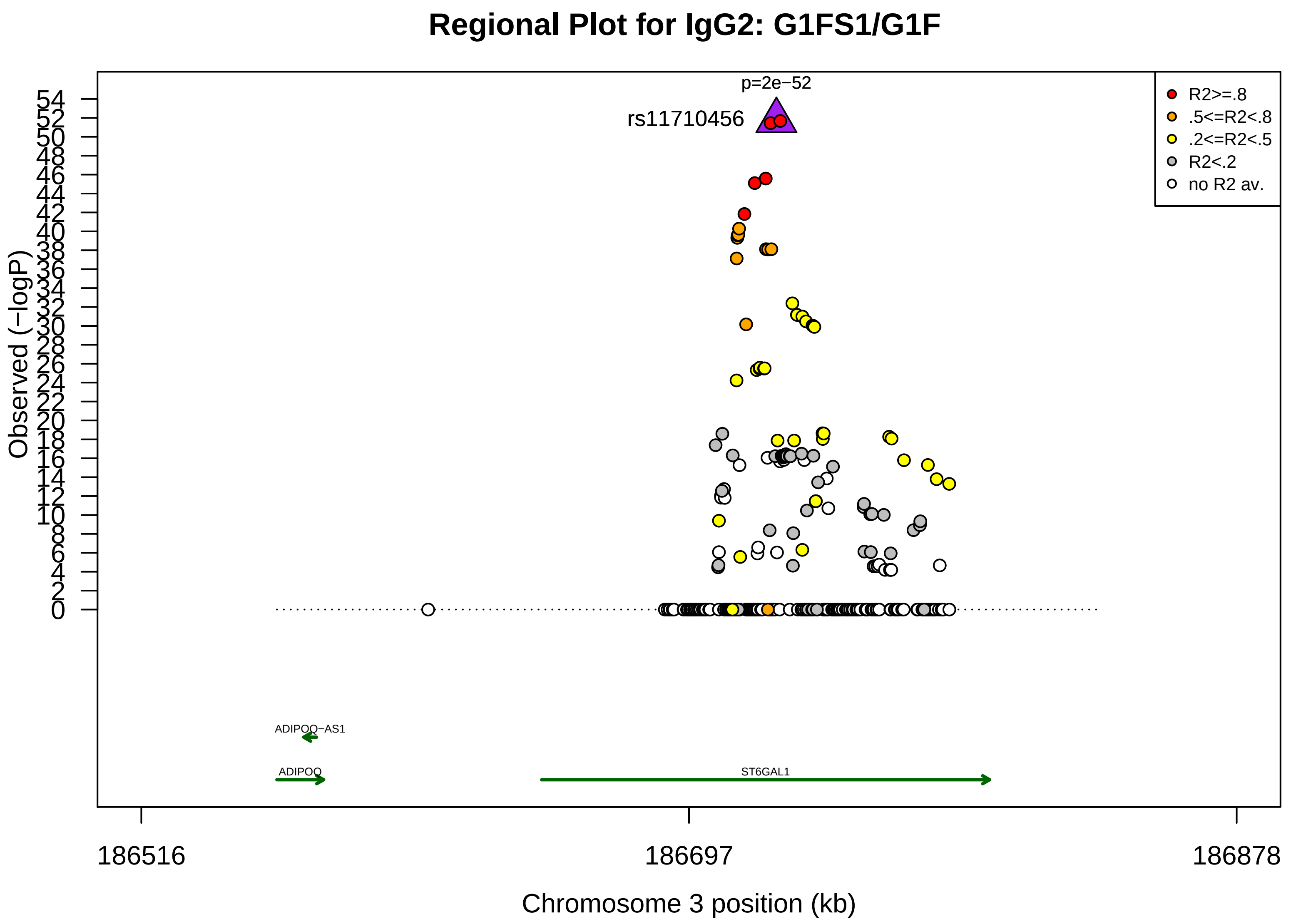

Regional Plot for IgG2: G1FS1/G1F
p=2e−52
rs11710456
R2>=.8
.5<=R2<.8
.2<=R2<.5
R2<.2
no R2 av.
Observed (−logP)
ADIPOQ−AS1
ADIPOQ
ST6GAL1
186516
186697
186878
Chromosome 3 position (kb)

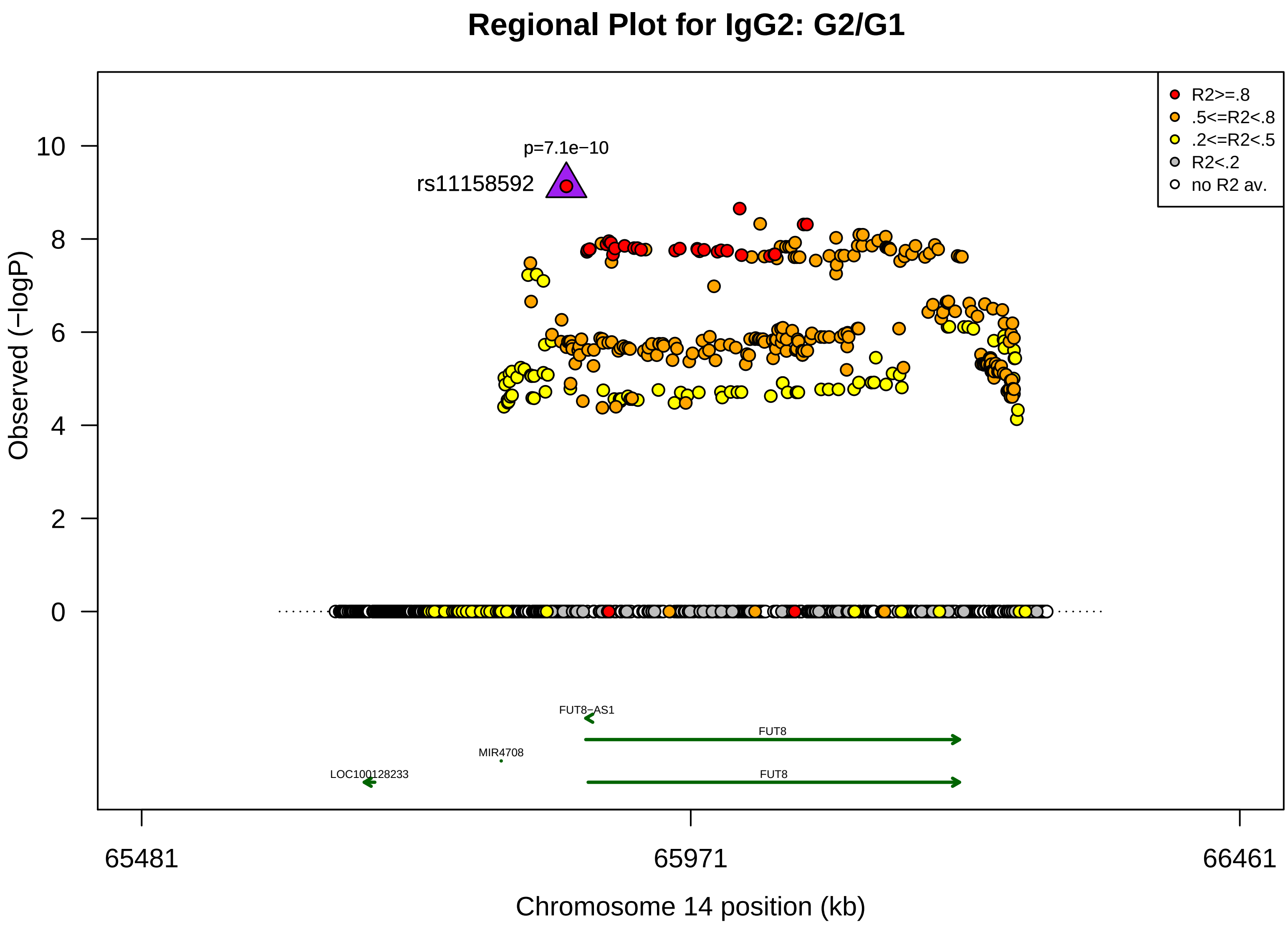
Regional Plot for IgG2: G2/G1
R2>=.8
.5<=R2<.8
.2<=R2<.5
R2<.2
no R2 av.
p=7.1e−10
rs11158592
Observed (−logP)
10
8
6
4
2
0
FUT8−AS1
FUT8
MIR4708
LOC100128233
FUT8
65481
65971
66461
Chromosome 14 position (kb)

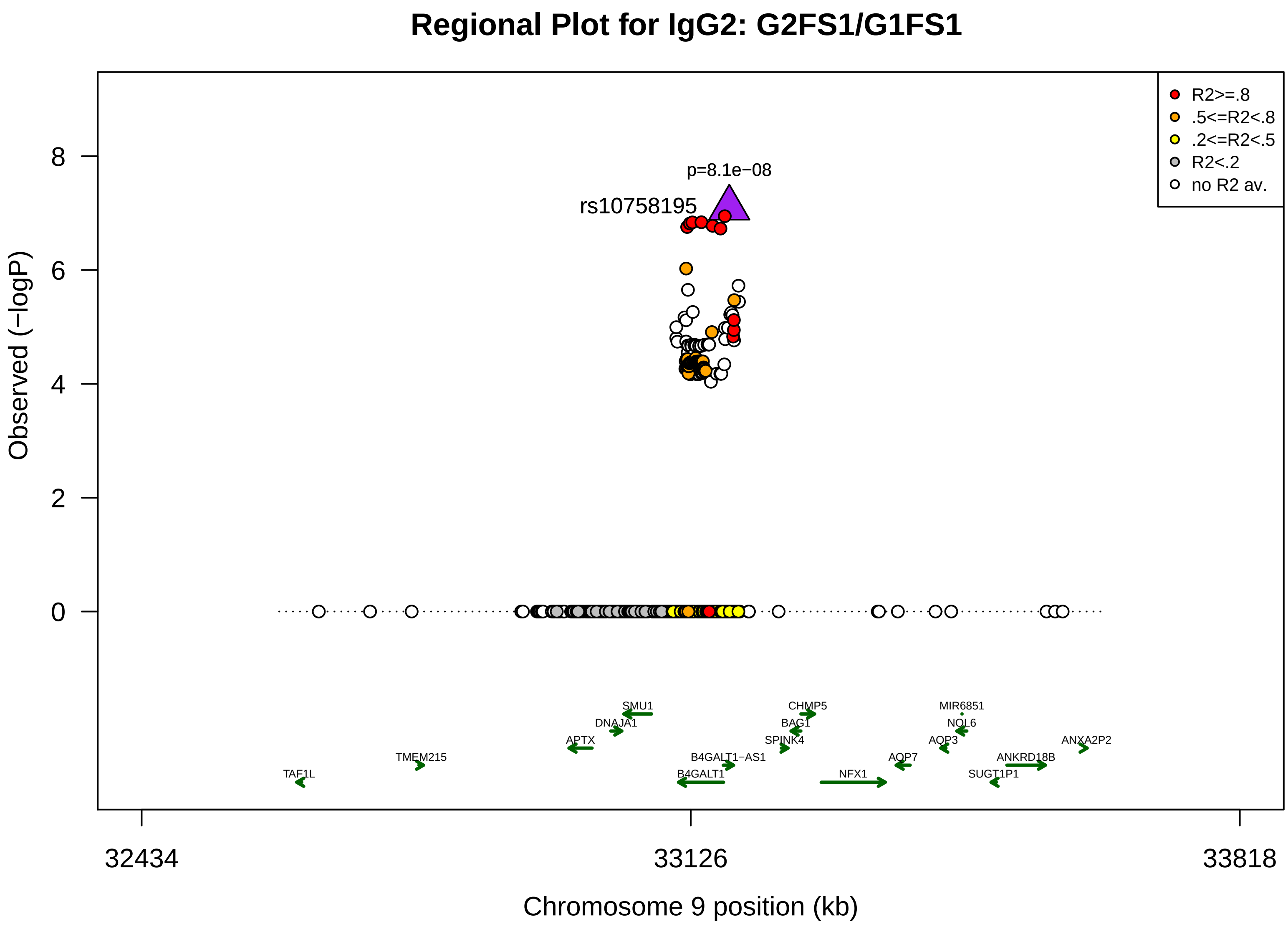

Regional Plot for IgG2: G2FS1/G1FS1
R2>=.8
.5<=R2<.8
.2<=R2<.5
R2<.2
no R2 av.
p=8.1e−08
rs10758195
Observed (−logP)
8
6
4
2
0
SMU1
DNAJA1
APTX
TMEM215
TAF1L
CHMP5
BAG1
SPINK4
B4GALT1−AS1
B4GALT1
NFX1
MIR6851
NOL6
AQP3
AQP7
ANKRD18B
SUGT1P1
ANXA2P2
32434
33126
33818
Chromosome 9 position (kb)

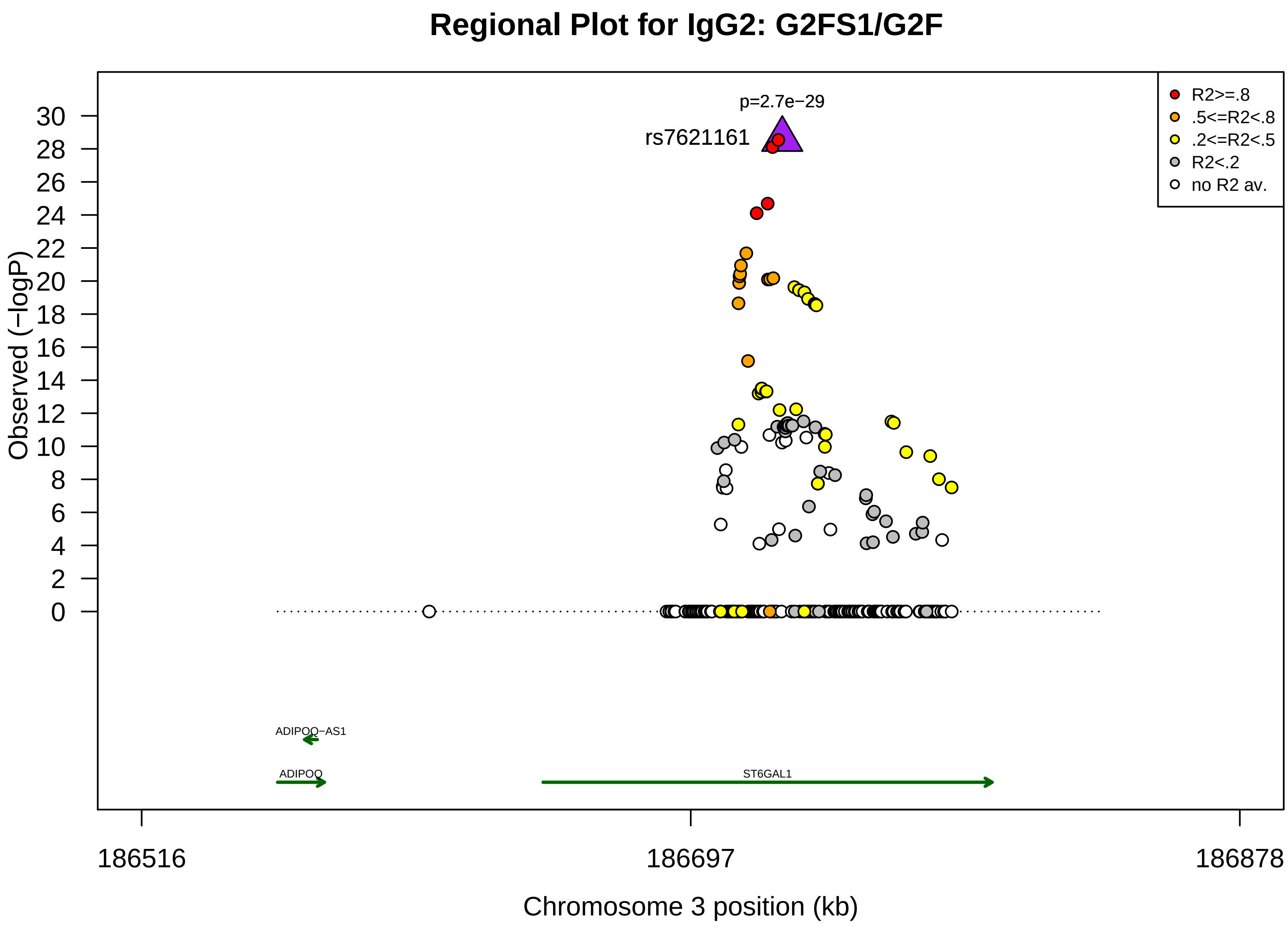

Regional Plot for IgG2: G2FS1/G2F
p=2.7e−29
rs7621161
R2>=.8
.5<=R2<.8
.2<=R2<.5
R2<.2
no R2 av.
Observed (−logP)
0
2
4
6
8
10
12
14
16
18
20
22
24
26
28
30
ADIPOQ−AS1
ADIPOQ
ST6GAL1
186516
186697
186878
Chromosome 3 position (kb)

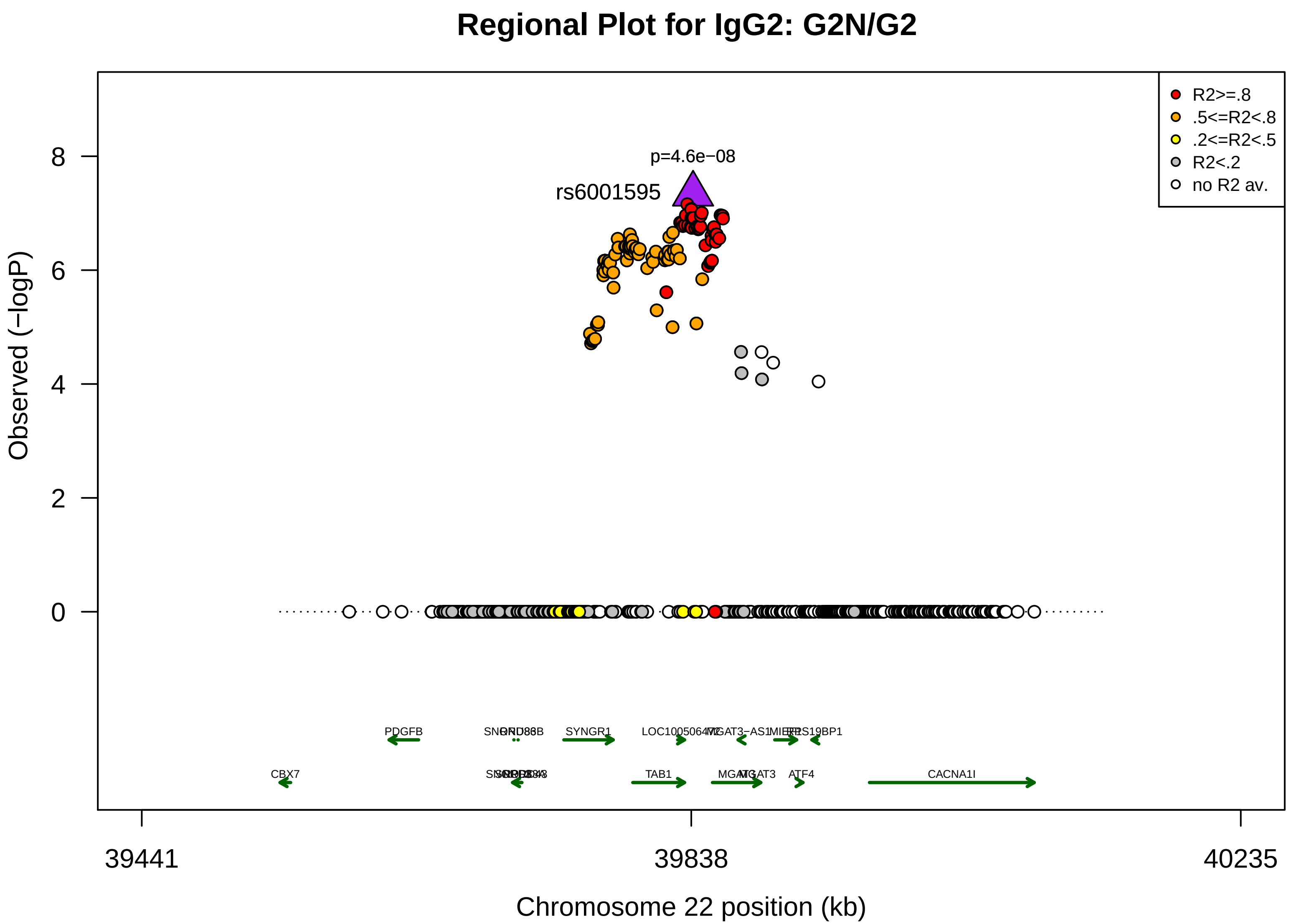
Regional Plot for IgG2: G2N/G2
Observed (−logP)
8
6
4
2
0
p=4.6e−08
rs6001595
R2>=.8
.5<=R2<.8
.2<=R2<.5
R2<.2
no R2 av.
PDGFB
SYNGR1
CBX7
TAB1
ATF4
CACNA1I
39441
39838
40235
Chromosome 22 position (kb)

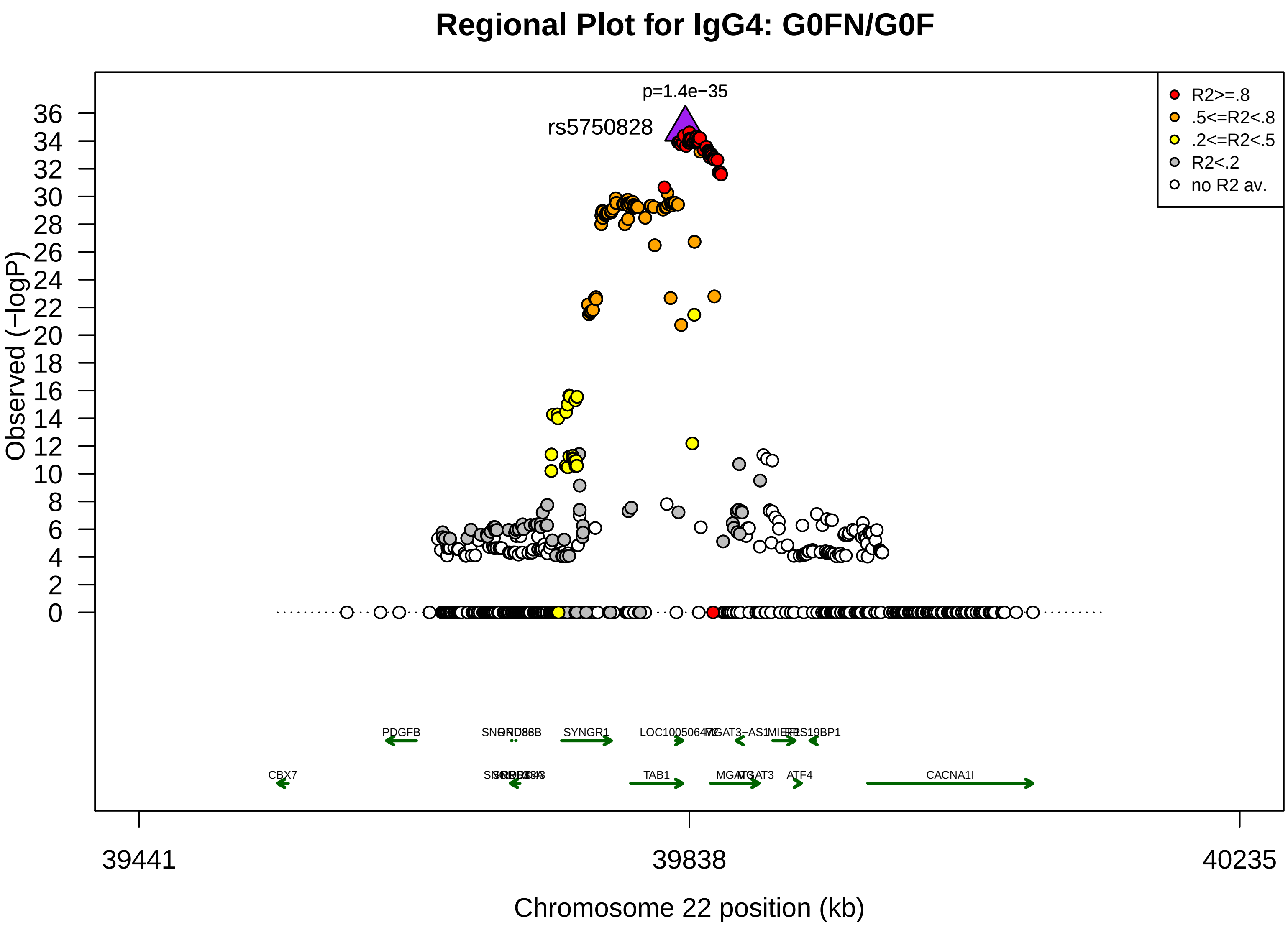

Regional Plot for IgG4: G0FN/G0F
p=1.4e−35
rs5750828
R2>=.8
.5<=R2<.8
.2<=R2<.5
R2<.2
no R2 av.
Observed (−logP)
0
2
4
6
8
10
12
14
16
18
20
22
24
26
28
30
32
34
36
PDGFB
SYNGR1
CBX7
TAB1
ATF4
CACNA1I
39441
39838
40235
Chromosome 22 position (kb)

Regional Plot for IgG4: G1FN/G1F

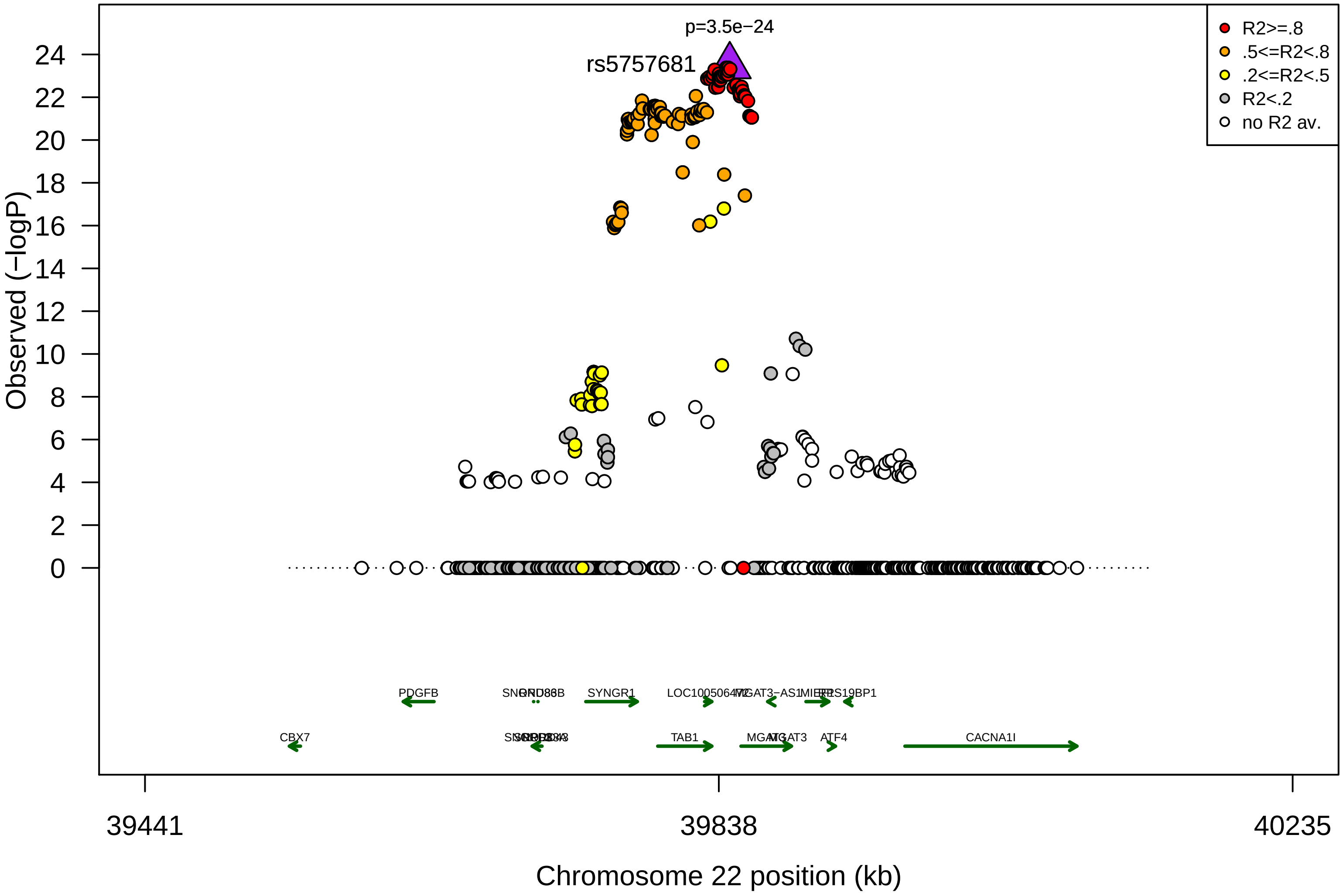

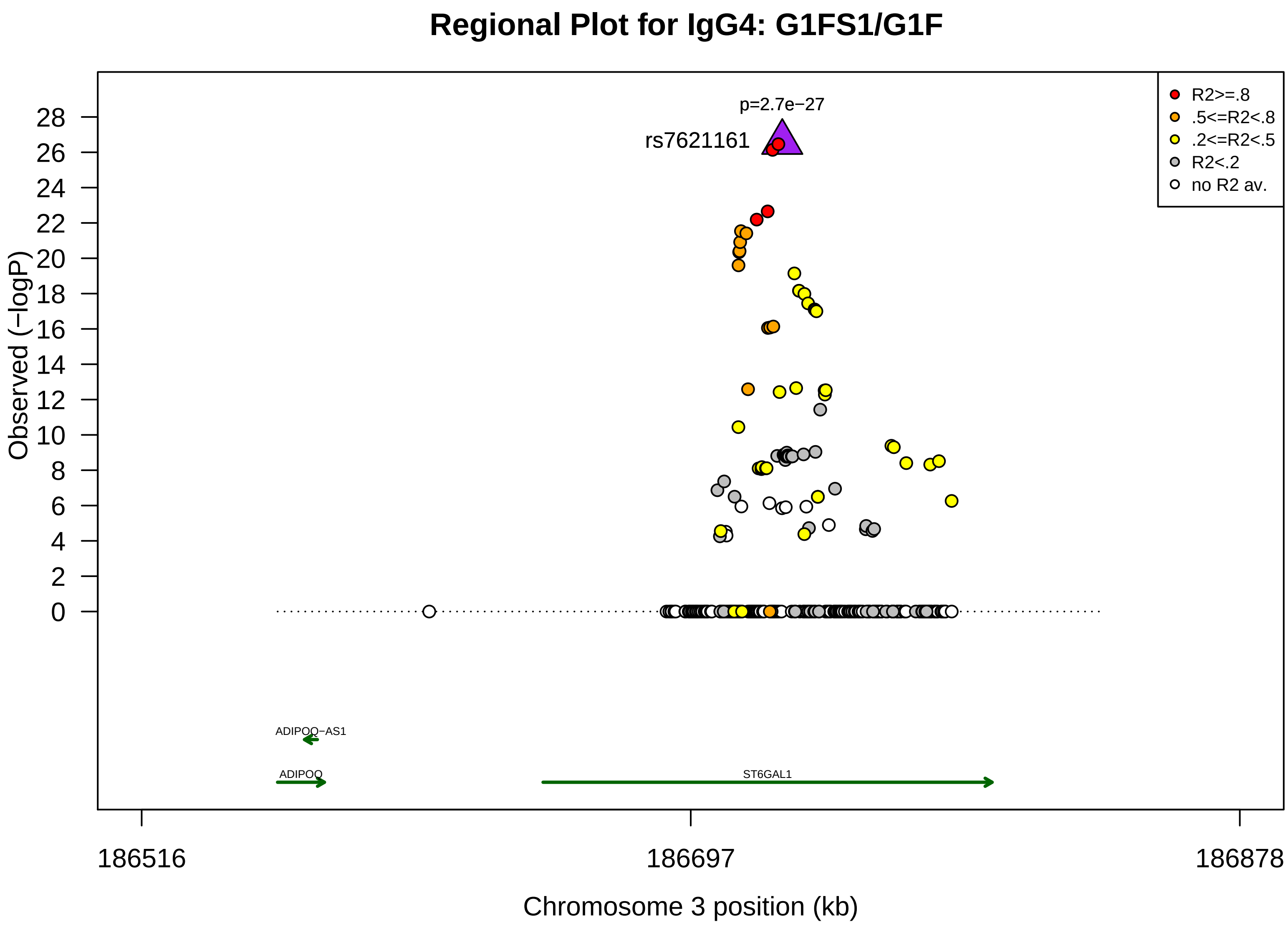

Regional Plot for IgG4: G1FS1/G1F
p=2.7e−27
rs7621161
R2>=.8
.5<=R2<.8
.2<=R2<.5
R2<.2
no R2 av.
Observed (−logP)
28
26
24
22
20
18
16
14
12
10
8
6
4
2
0
ADIPOQ−AS1
ADIPOQ
ST6GAL1
186516
186697
186878
Chromosome 3 position (kb)

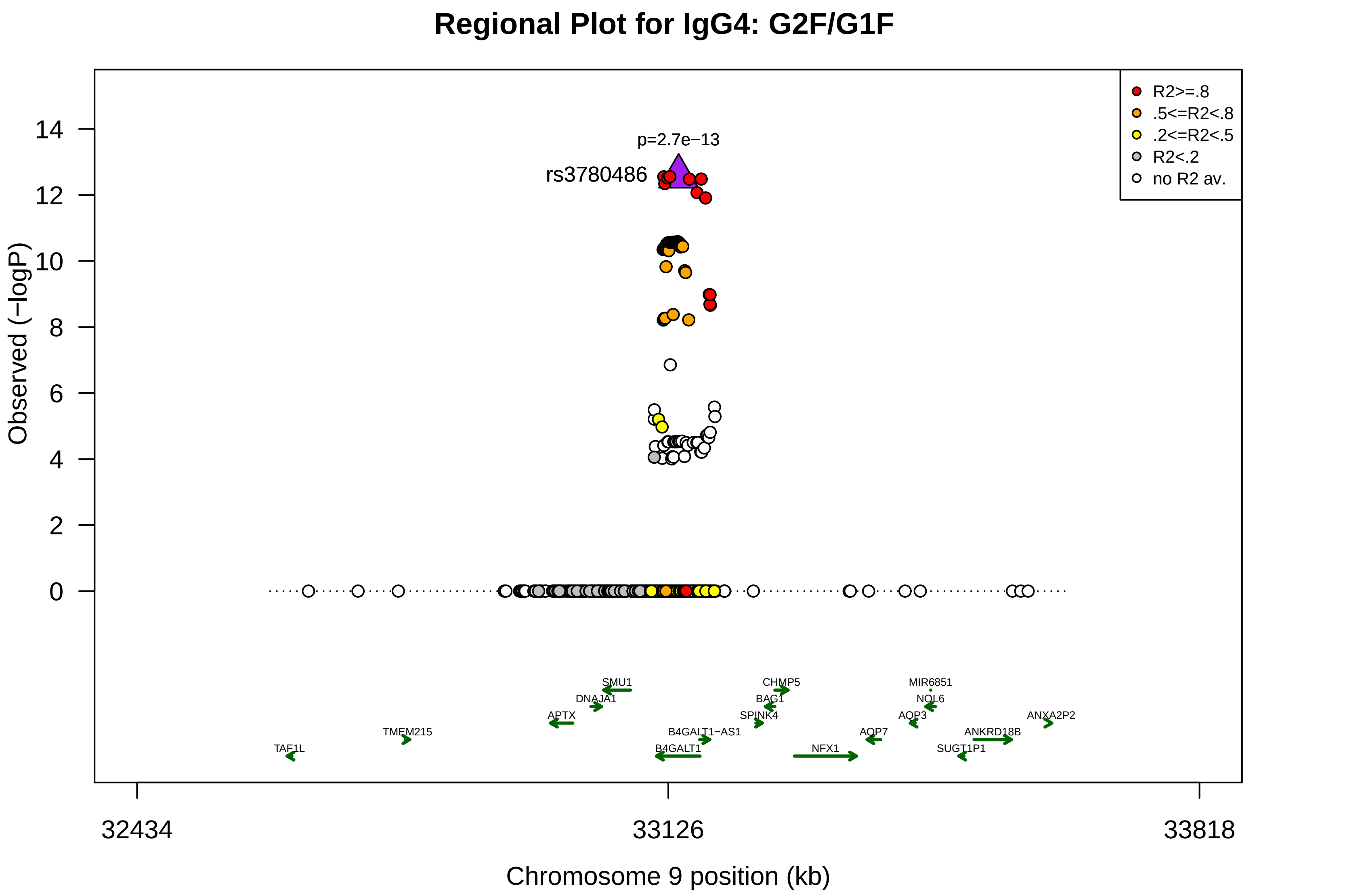

Regional Plot for IgG4: G2F/G1F
R2>=.8
.5<=R2<.8
.2<=R2<.5
R2<.2
no R2 av.
p=2.7e−13
rs3780486
Observed (−logP)
0
2
4
6
8
10
12
14
SMU1
DNAJA1
APTX
TMEM215
TAF1L
CHMP5
BAG1
SPINK4
B4GALT1−AS1
B4GALT1
NFX1
MIR6851
NOL6
AQP3
AQP7
ANXA2P2
ANKRD18B
SUGT1P1
32434
33126
33818
Chromosome 9 position (kb)

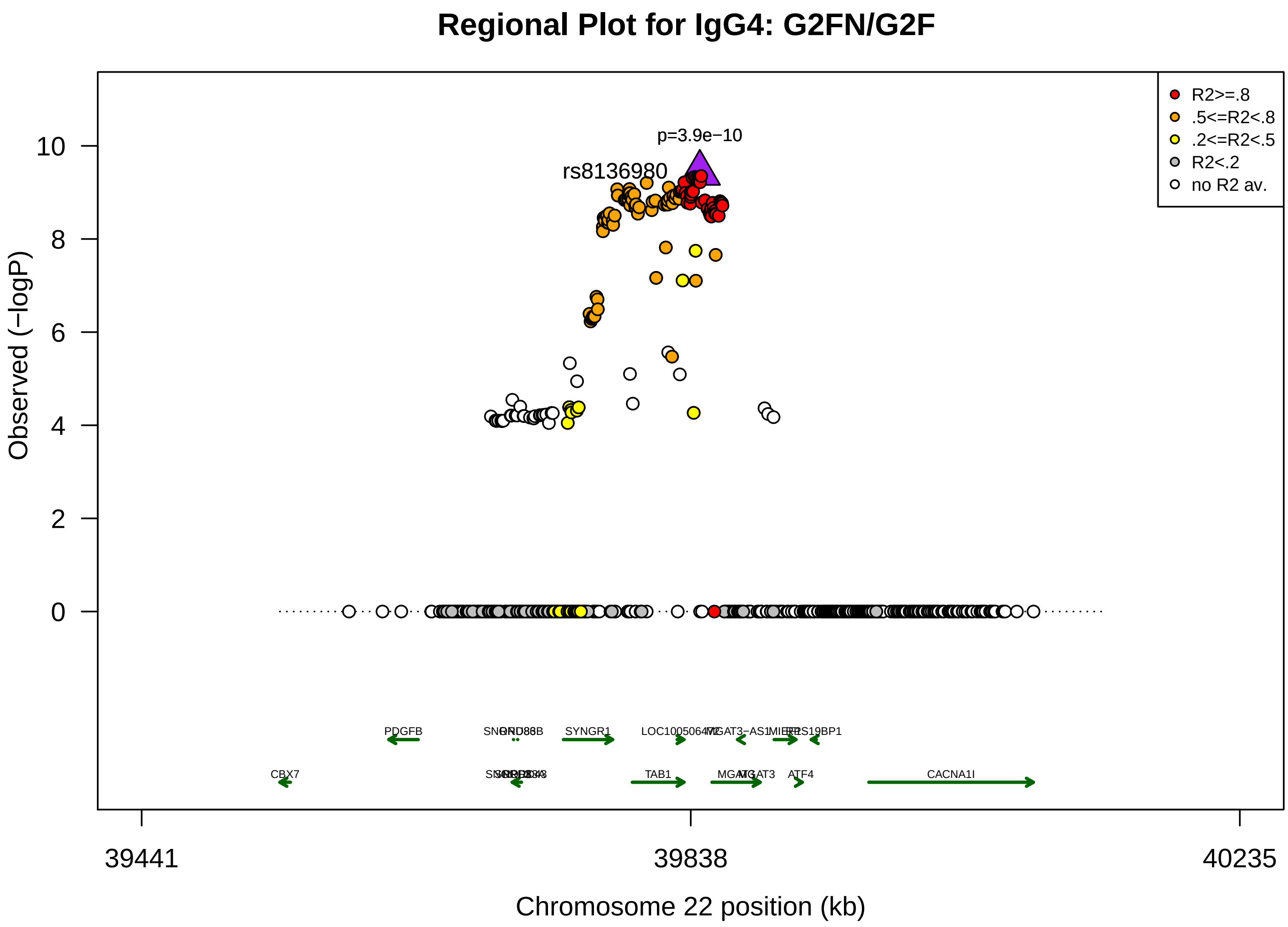
Regional Plot for IgG4: G2FN/G2F
R2>=.8
.5<=R2<.8
.2<=R2<.5
R2<.2
no R2 av.
p=3.9e−10
rs8136980
Observed (−logP)
0
2
4
6
8
10
PDGFB
SYNGR1
CBX7
TAB1
ATF4
CACNA1I
39441
39838
40235
Chromosome 22 position (kb)

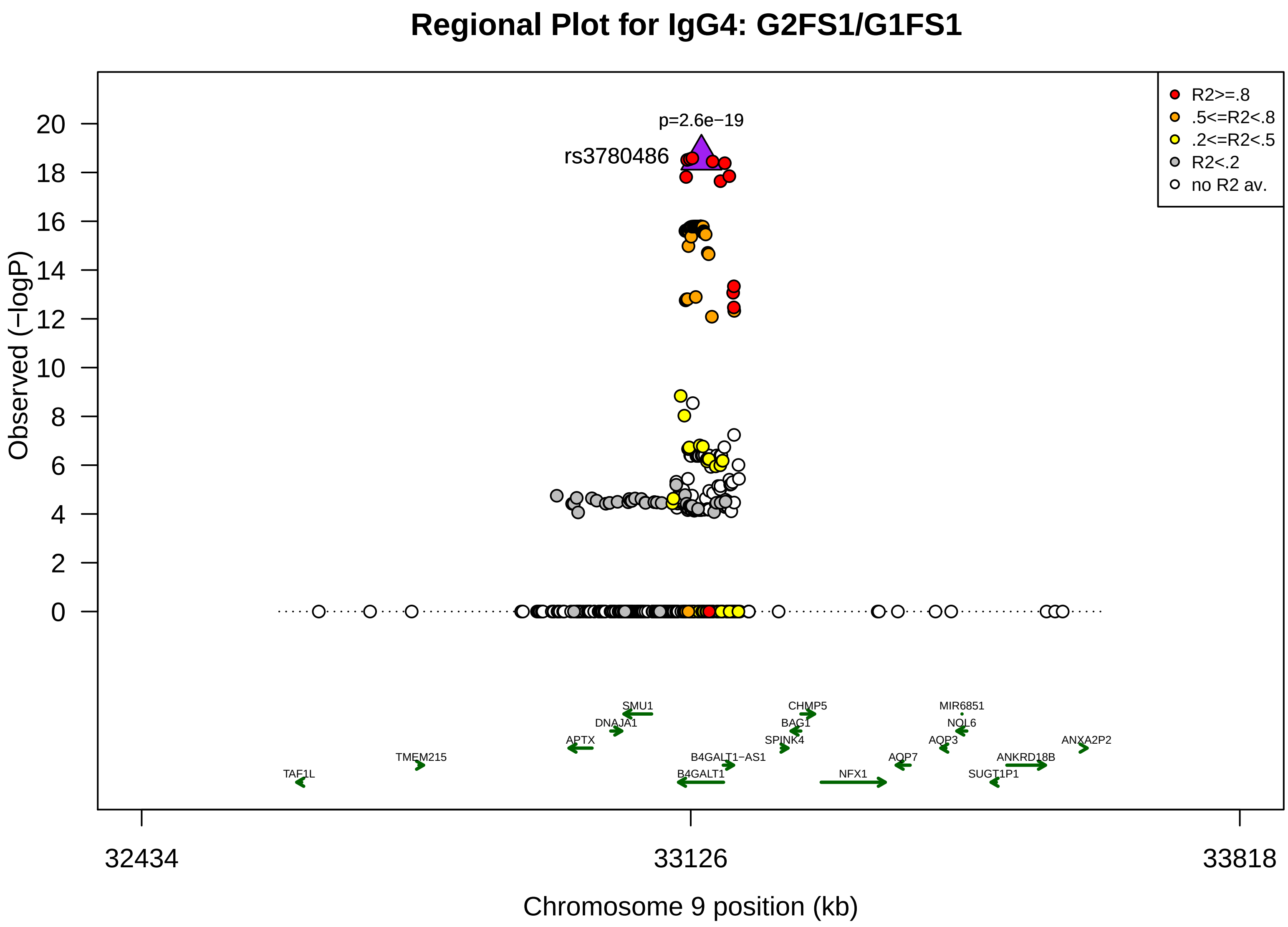
Regional Plot for IgG4: G2FS1/G1FS1
p=2.6e−19
rs3780486
R2>=.8
.5<=R2<.8
.2<=R2<.5
R2<.2
no R2 av.
Observed (−logP)
0
2
4
6
8
10
12
14
16
18
20
SMU1
CHMP5
MIR6851
DNAJA1
BAG1
NOL6
APTX
SPINK4
AQP3
ANXA2P2
TMEM215
B4GALT1−AS1
AQP7
ANKRD18B
TAF1L
B4GALT1
NFX1
SUGT1P1
32434
33126
33818
Chromosome 9 position (kb)

Regional Plot for IgG4: G2FS1/G2F

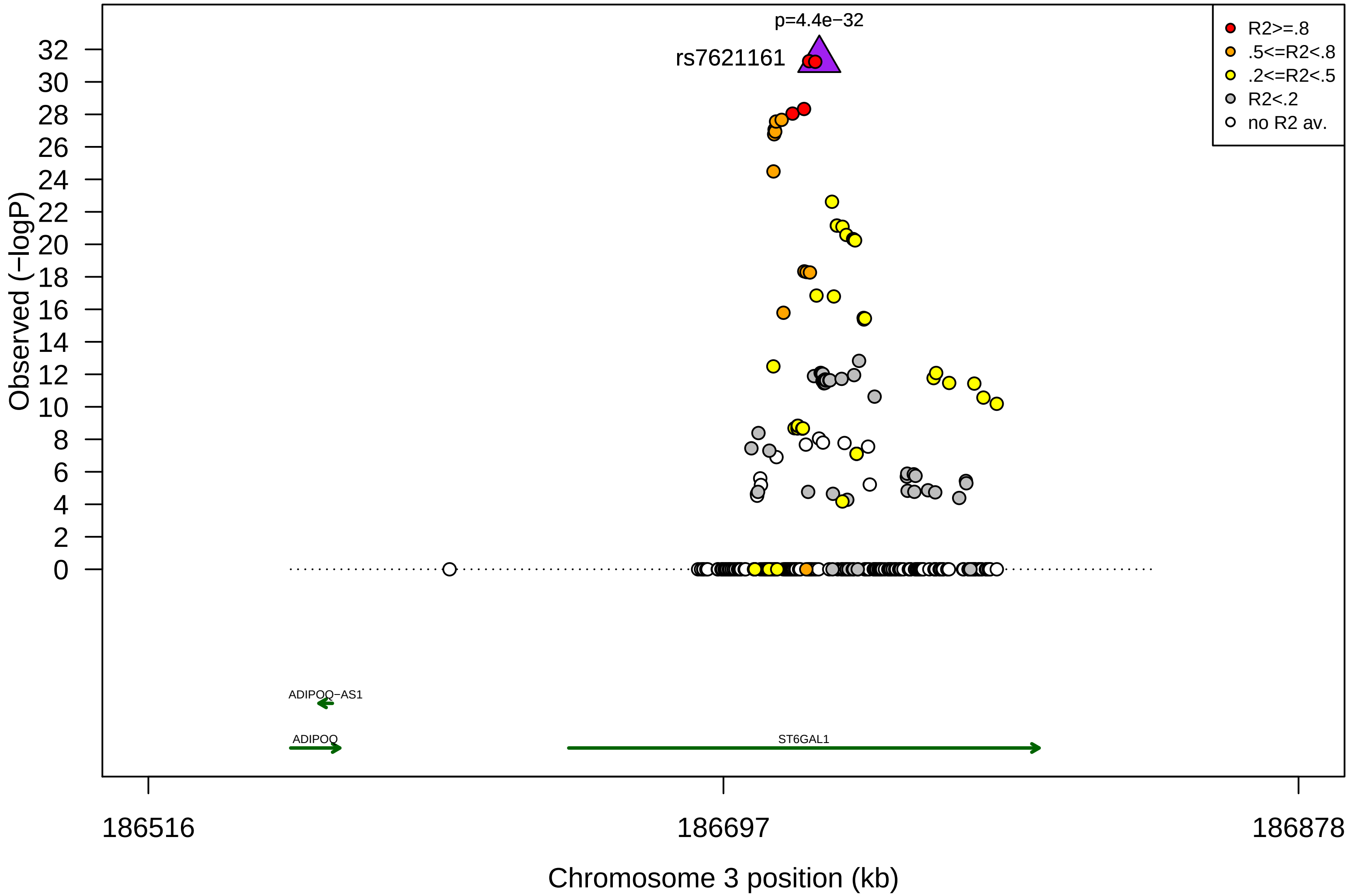

Supplementary Figure 7. Regional Association plots.

For all ratios that showed at least a suggestive (p-value ≤ 10^{-7}) or significant (p-value ≤ $5.26 \cdot 10^{-10}$) associations with SNPs in a given glycosyltransferase gene with a p-gain≥10, regional association plots were created for the SNP with the lowest p-value using gene information obtained from UCSC Genome Browser (GRCh37/hg19)[14] and LD information from SNIPA[15]. The plot includes all SNPs having a p-value < 10^{-4} or an R^2 ≥ 0.1 to the lead-SNP. LD information is given by R^2 in color, the y-axis shows the p-values of the association of the single SNPs with the indicated glycan ratio and SNPs with p-values above 10^{-4} are located on the x-axis.

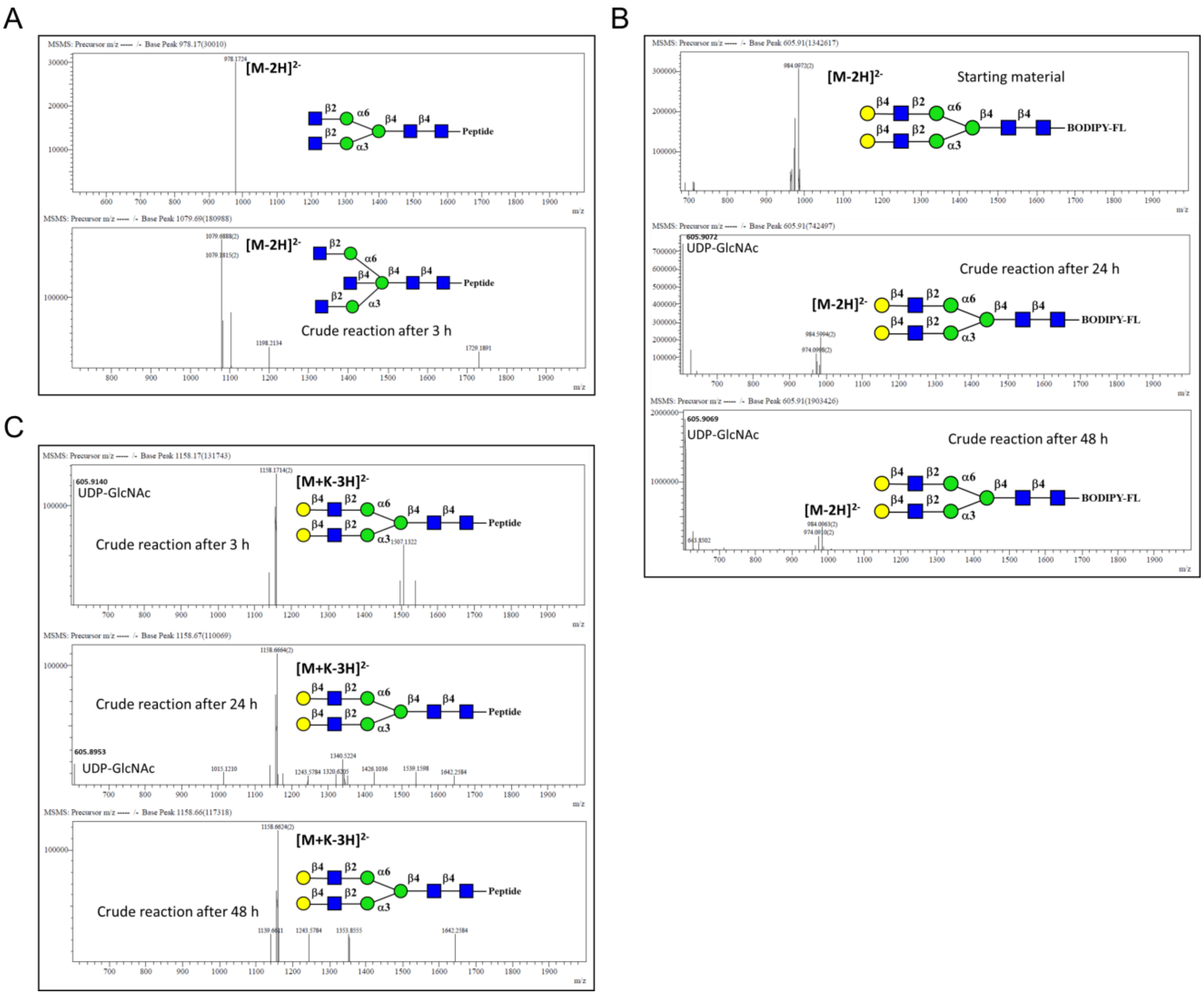

Supplementary Figure 8: Enzymatic assay on synthetic substrates for MGAT3.

A Initial glycopeptide: G0. The structure is a known substrate for MGAT3 and after three hours the substrate was completely converted into product (G0N).

B Initial glycopeptide: G2. The substrate did not show any conversion to the product after 24 or 48 hours. This is compatible with our prediction that rule N1 is unfeasible

C Initial structure: fluorescently labeled G2. The previous result was confirmed also with this alternative substrate, which did not show any conversion after up to 48 hours.

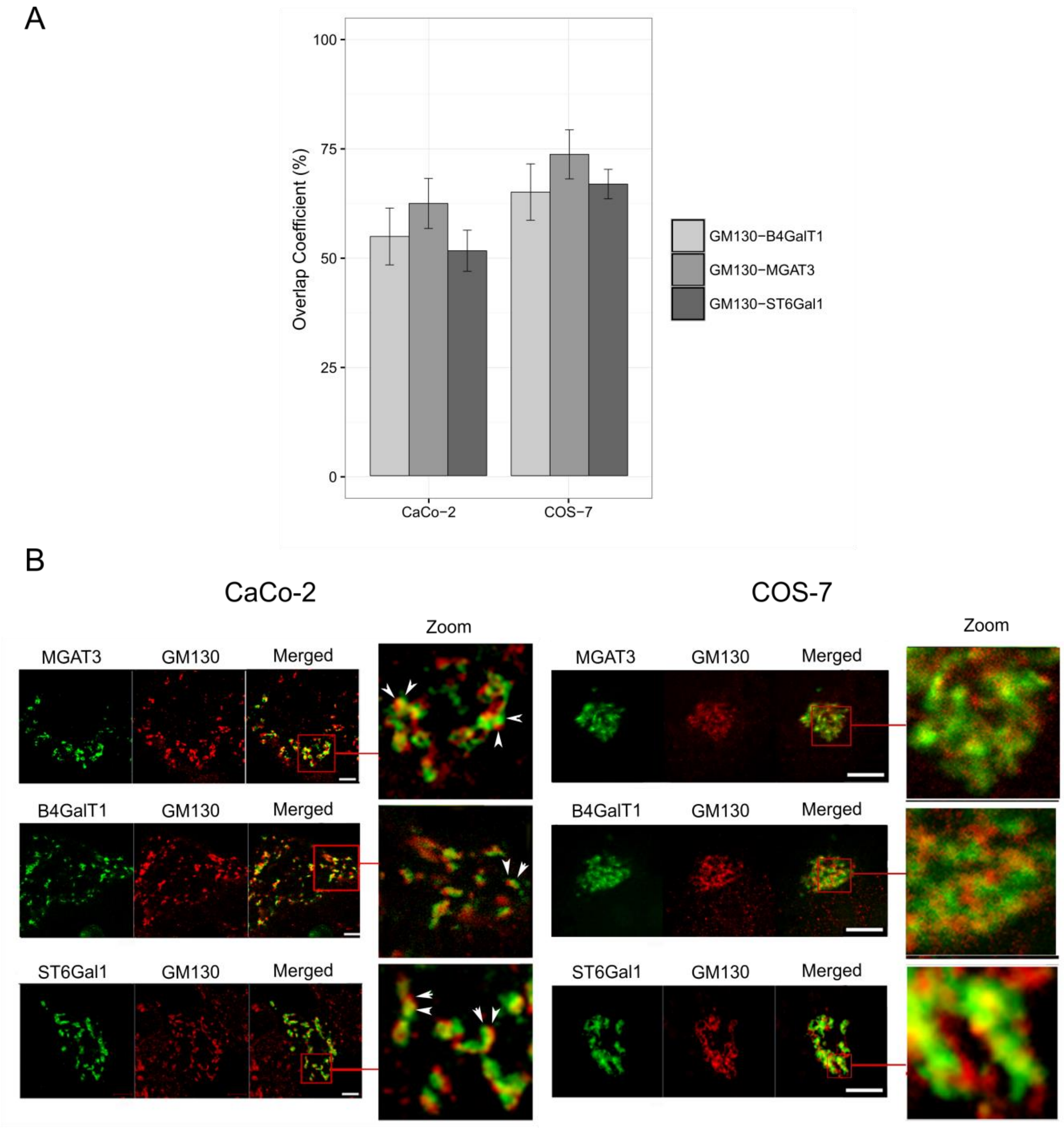

Supplementary Figure 9: Colocalization experiment.

A Quantitative overlap between the three enzymes and the cis-Golgi marker GM130. The overall colocalization of each enzyme pair was expressed as overlap coefficient percentage (mean % ± standard deviation) obtained using pixel per pixel comparison of each Z-stack image and by combining the values across 5 cells each.

B Colocalization images of B4GalT1, ST6Gal-1 and MGAT3 with GM130 in CaCo-2 (left) and COS-7 (right) cells. COS-7 cells and CaCo-2 cells were grown on cell culture plates and transfected with the defined plasmids, and processed for confocal Z-stack imaging (0.3 µm sections). The individual figures represent a typical view from 5 different Golgi areas examined. In the images labeled as "Merged" and "Zoom", yellow areas represent localization overlap. The cis-Golgi

GM130 marker protein and the three enzymes showed differential localization in CaCo-2 cells, indicating that the dispersed Golgi stacks are functionally polarized in this colorectal cancer cell line. This was evident from the observed differential distribution of the green and red colours within each Golgi stack of cisternae (arrowheads in figure). Yet, each enzyme showed partial colocalization with the cis-Golgi-marker GM130 based on coalescence of the two colours (yellow areas in figure) in the middle of the Golgi stack. Higher overlap percentages were detected in COS-7 cells due to the more compact Golgi architecture in the cells. Bar represents 5µm.

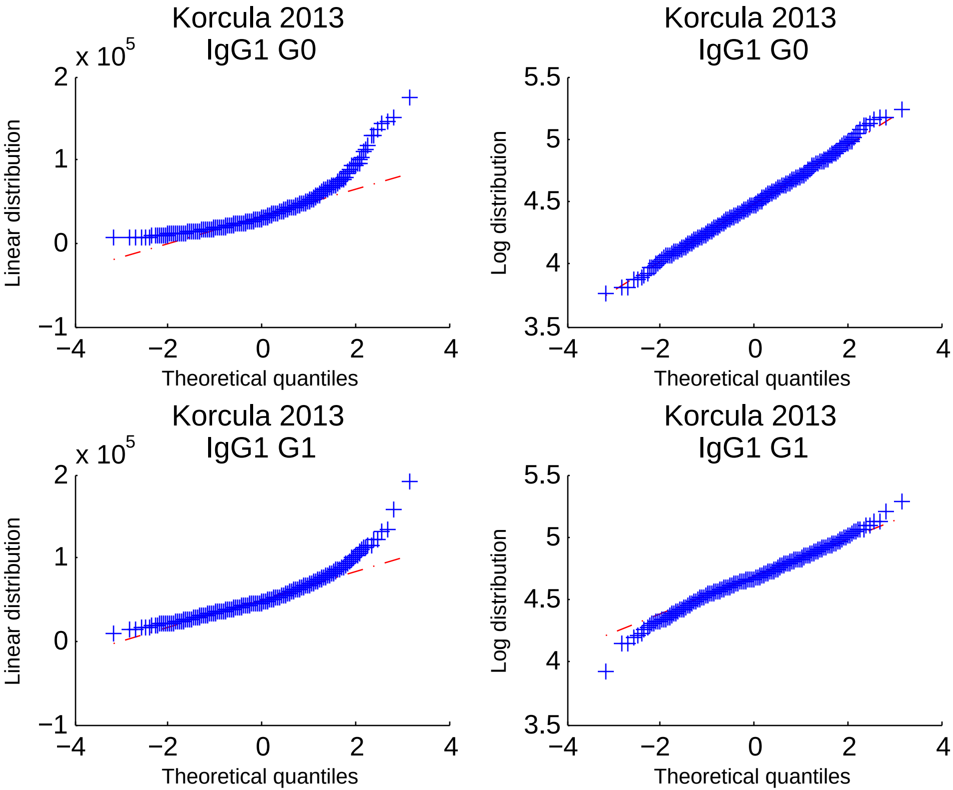
Korcula 2013
IgG1 G0
x 10^5
Linear distribution
Theoretical quantiles
Korcula 2013
IgG1 G0
Log distribution
Theoretical quantiles
Korcula 2013
IgG1 G1
x 10^5
Linear distribution
Theoretical quantiles
Korcula 2013
IgG1 G1
Log distribution
Theoretical quantiles

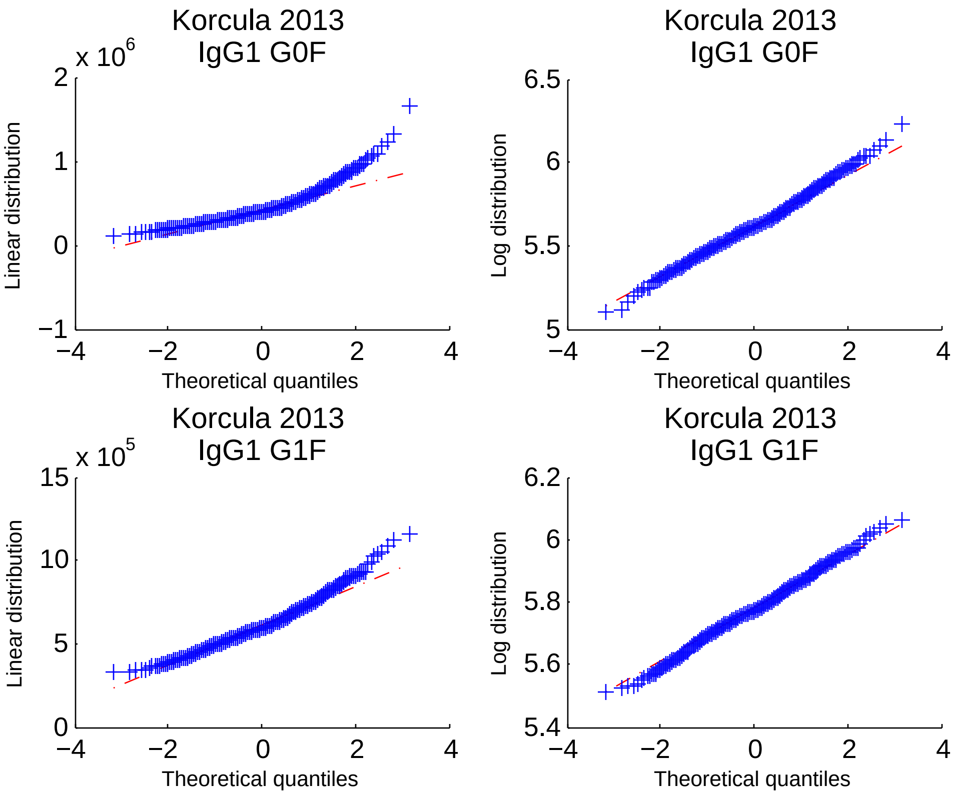

Korcula 2013
IgG1 G0F
x 10^6
Linear distribution
Theoretical quantiles
Korcula 2013
IgG1 G0F
Log distribution
Theoretical quantiles
Korcula 2013
IgG1 G1F
x 10^5
Linear distribution
Theoretical quantiles
Korcula 2013
IgG1 G1F
Log distribution
Theoretical quantiles

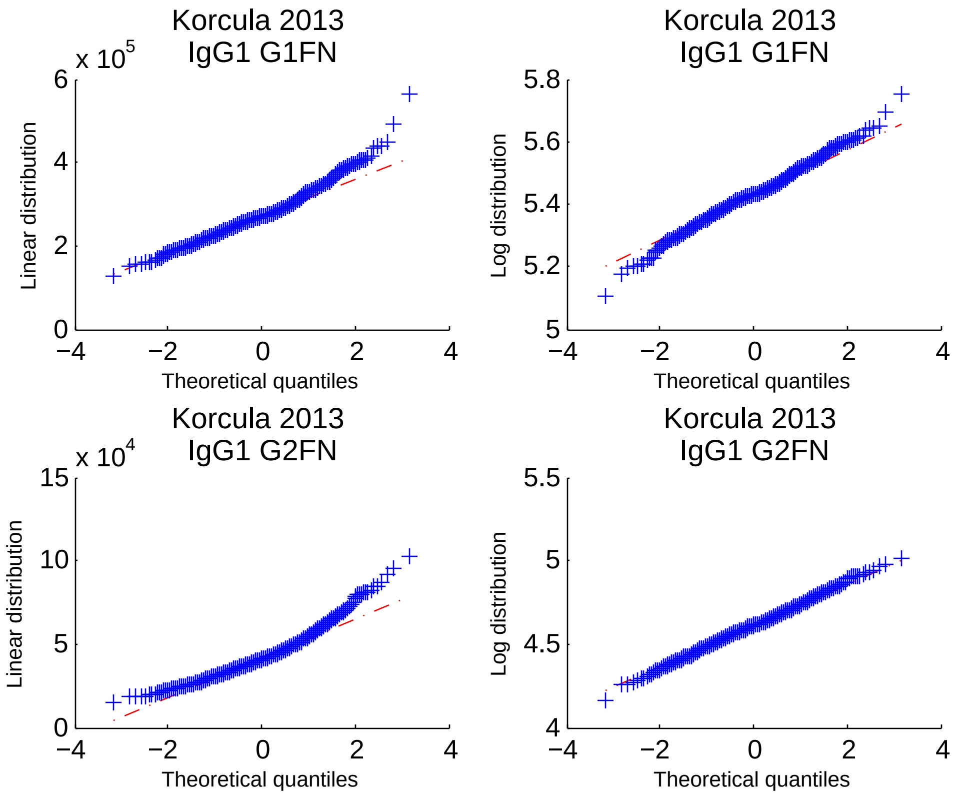
Korcula 2013
IgG1 G1FN
x 10^5
Linear distribution
Theoretical quantiles
6
4
2
0
−4
−2
0
2
4
Korcula 2013
IgG1 G1FN
Log distribution
Theoretical quantiles
5.8
5.6
5.4
5.2
5
−4
−2
0
2
4
Korcula 2013
IgG1 G2FN
x 10^4
Linear distribution
Theoretical quantiles
15
10
5
0
−4
−2
0
2
4
Korcula 2013
IgG1 G2FN
Log distribution
Theoretical quantiles
5.5
5
4.5
4
−4
−2
0
2
4

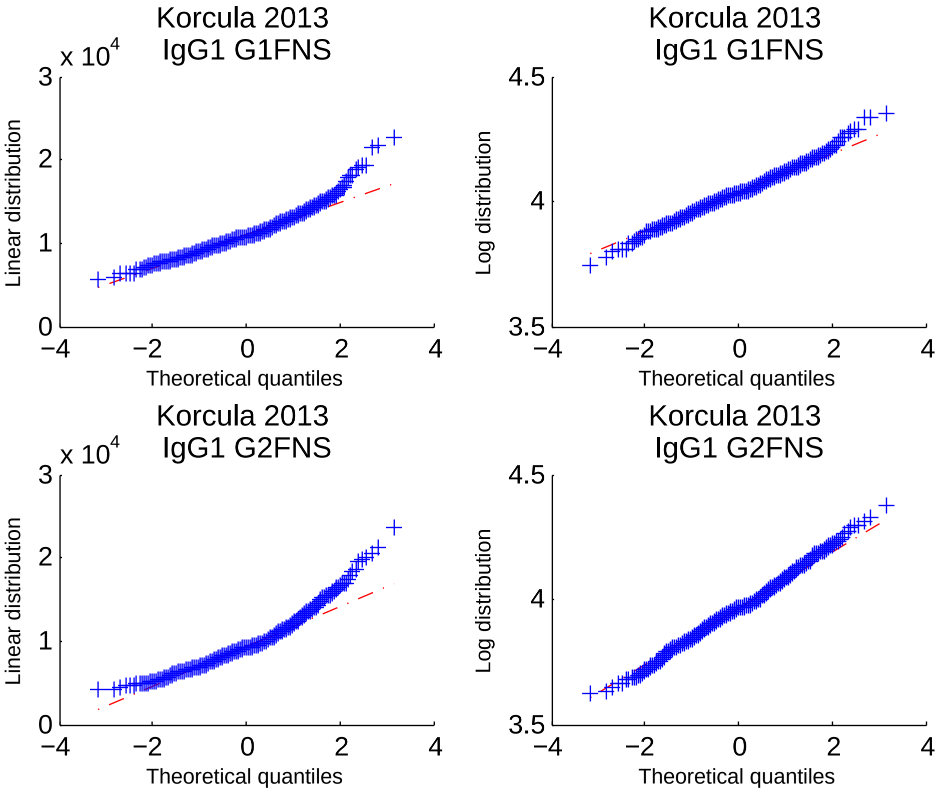
Korcula 2013
IgG1 G1FNS
x 10^4
Linear distribution
Theoretical quantiles
Korcula 2013
IgG1 G1FNS
Log distribution
Theoretical quantiles
Korcula 2013
IgG1 G2FNS
x 10^4
Linear distribution
Theoretical quantiles
Korcula 2013
IgG1 G2FNS
Log distribution
Theoretical quantiles

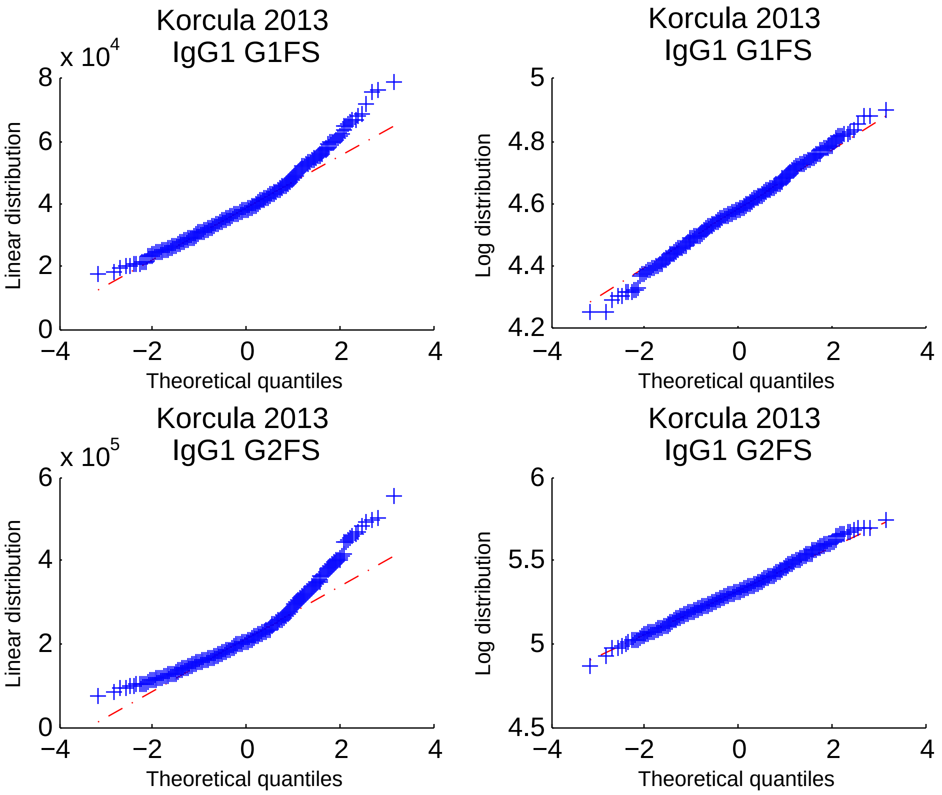
Korcula 2013
IgG1 G1FS
x 10^4
Linear distribution
Theoretical quantiles
Korcula 2013
IgG1 G1FS
Log distribution
Theoretical quantiles
Korcula 2013
IgG1 G2FS
x 10^5
Linear distribution
Theoretical quantiles
Korcula 2013
IgG1 G2FS
Log distribution
Theoretical quantiles

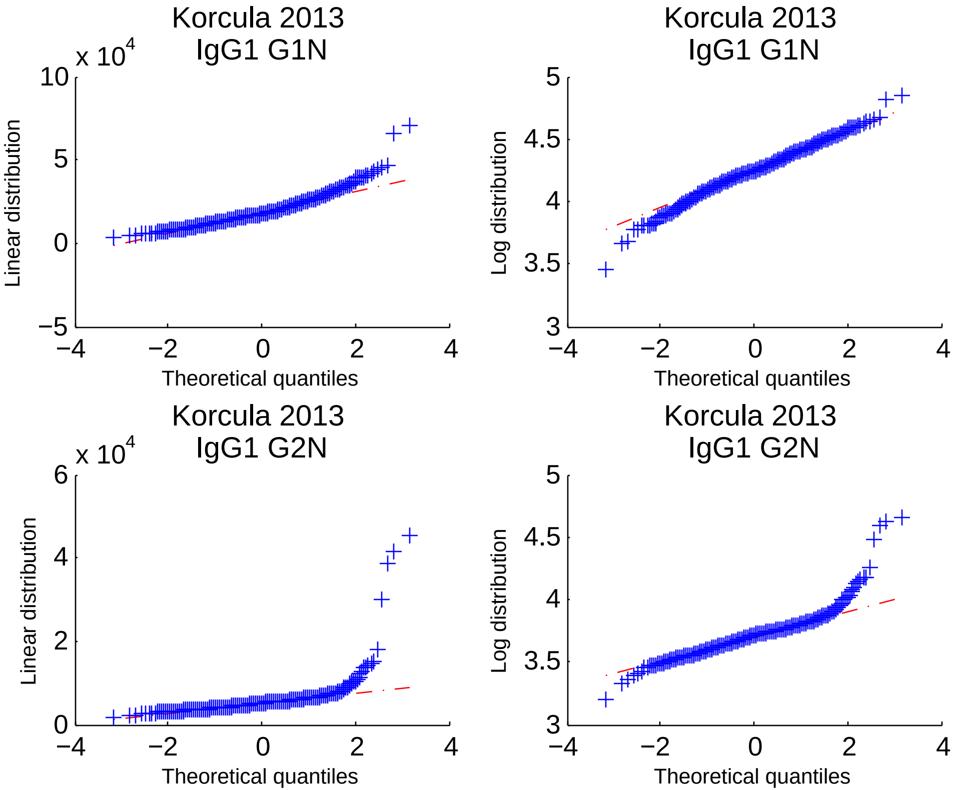

Korcula 2013
IgG1 G1N
x 10^4
10
5
0
−5
Linear distribution
−4
−2
0
2
4
Theoretical quantiles
Korcula 2013
IgG1 G1N
5
4.5
4
3.5
3
Log distribution
−4
−2
0
2
4
Theoretical quantiles
Korcula 2013
IgG1 G2N
x 10^4
6
4
2
0
Linear distribution
−4
−2
0
2
4
Theoretical quantiles
Korcula 2013
IgG1 G2N
5
4.5
4
3.5
3
Log distribution
−4
−2
0
2
4
Theoretical quantiles

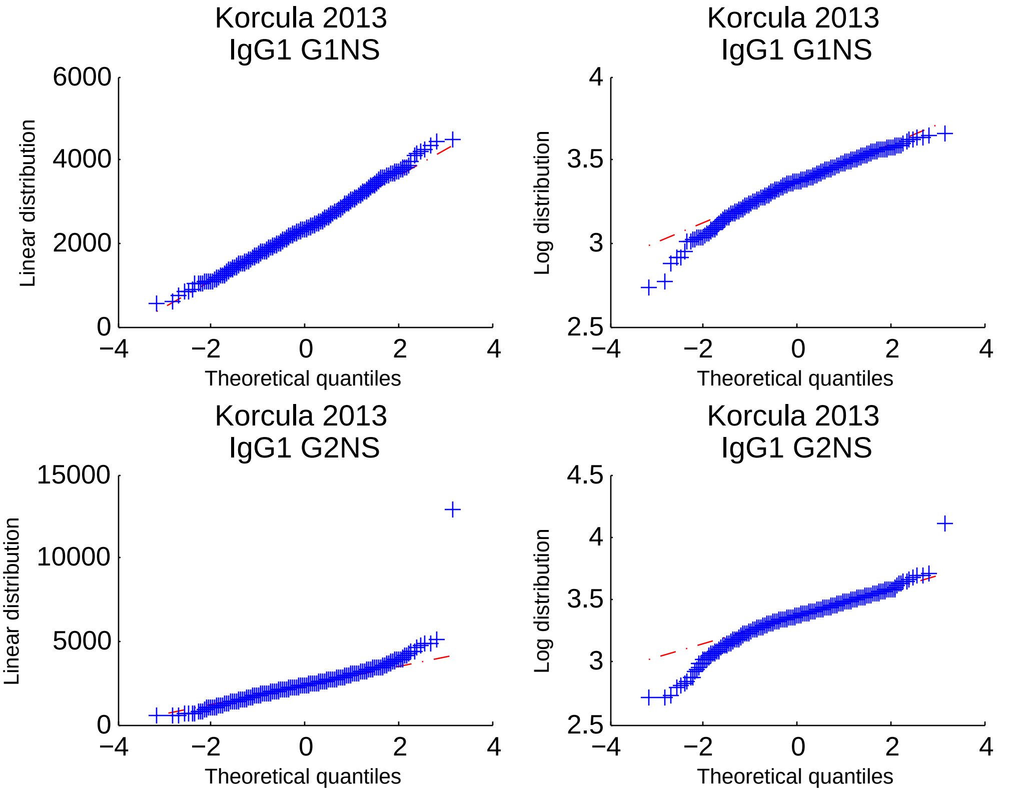
Korcula 2013
IgG1 G1NS
Linear distribution
Theoretical quantiles
Korcula 2013
IgG1 G1NS
Log distribution
Theoretical quantiles
Korcula 2013
IgG1 G2NS
Linear distribution
Theoretical quantiles
Korcula 2013
IgG1 G2NS
Log distribution
Theoretical quantiles

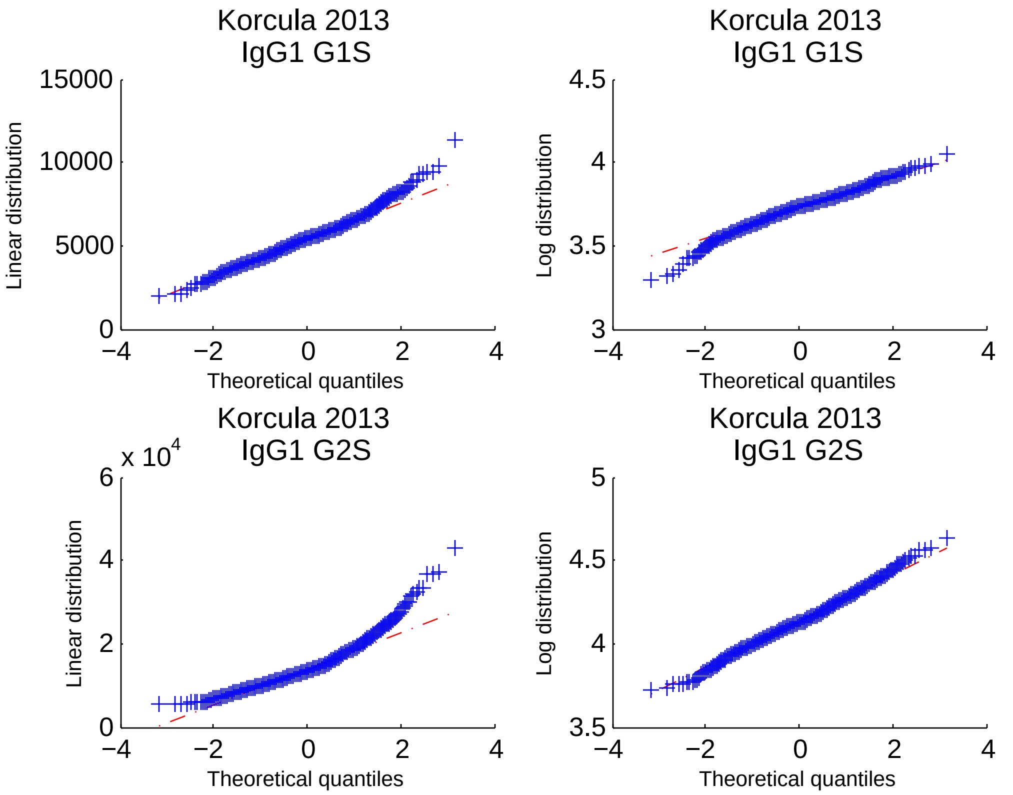
Korcula 2013
IgG1 G1S
Linear distribution
Theoretical quantiles
15000
10000
5000
0
−4
−2
0
2
4
Korcula 2013
IgG1 G1S
Log distribution
Theoretical quantiles
4.5
4
3.5
3
Korcula 2013
IgG1 G2S
x 10^4
Linear distribution
6
4
2
0
Korcula 2013
IgG1 G2S
Log distribution
5
4.5
4
3.5

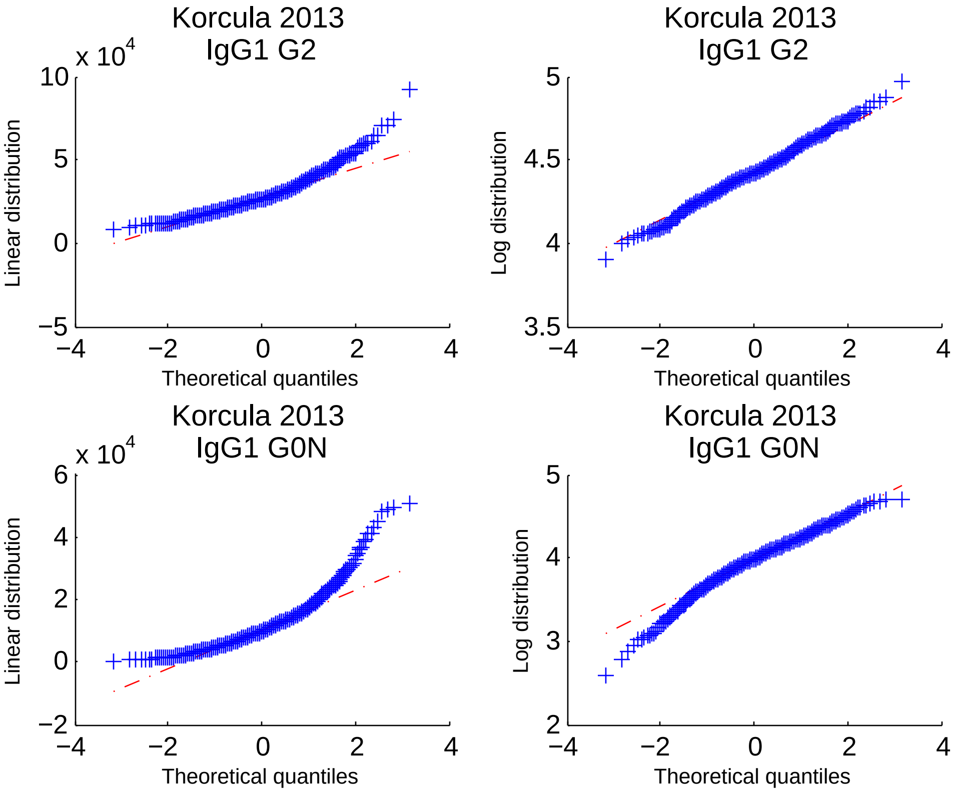
Korcula 2013
IgG1 G2
x 10^4
Linear distribution
Theoretical quantiles
Korcula 2013
IgG1 G2
Log distribution
Theoretical quantiles
Korcula 2013
IgG1 G0N
x 10^4
Linear distribution
Theoretical quantiles
Korcula 2013
IgG1 G0N
Log distribution
Theoretical quantiles

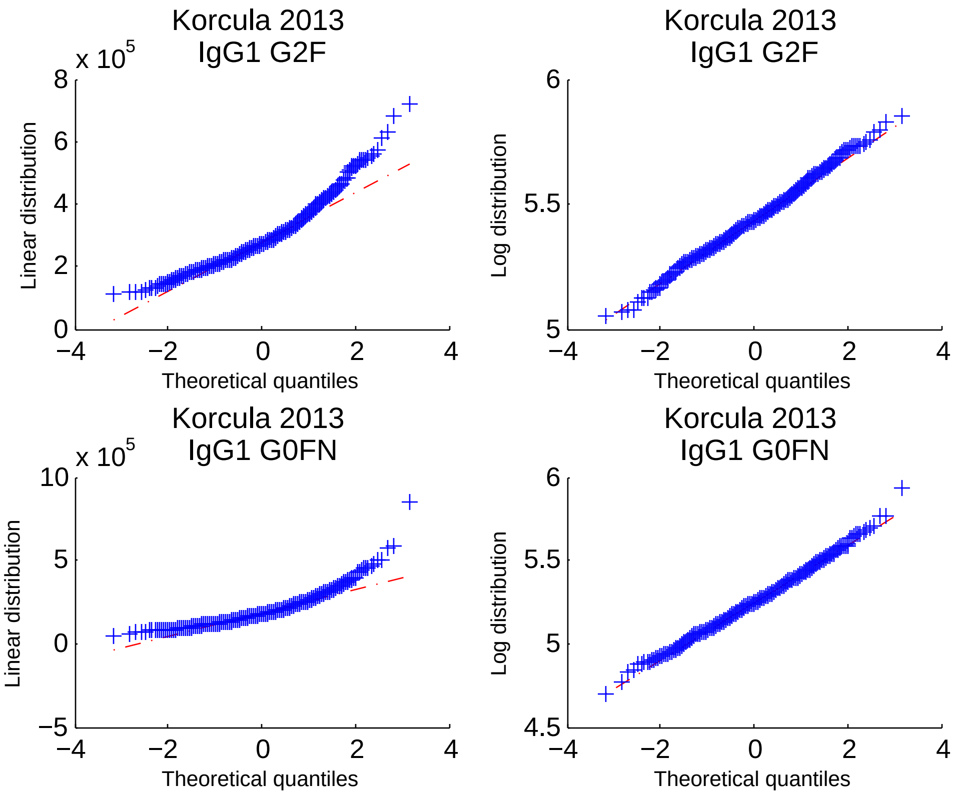

Korcula 2013
IgG1 G2F
x 10^5
8
6
4
2
0
−4
−2
0
2
4
Linear distribution
Theoretical quantiles
Korcula 2013
IgG1 G2F
6
5.5
5
Log distribution
Theoretical quantiles
Korcula 2013
IgG1 G0FN
x 10^5
10
5
0
−5
Linear distribution
Theoretical quantiles
Korcula 2013
IgG1 G0FN
6
5.5
5
4.5
Log distribution
Theoretical quantiles

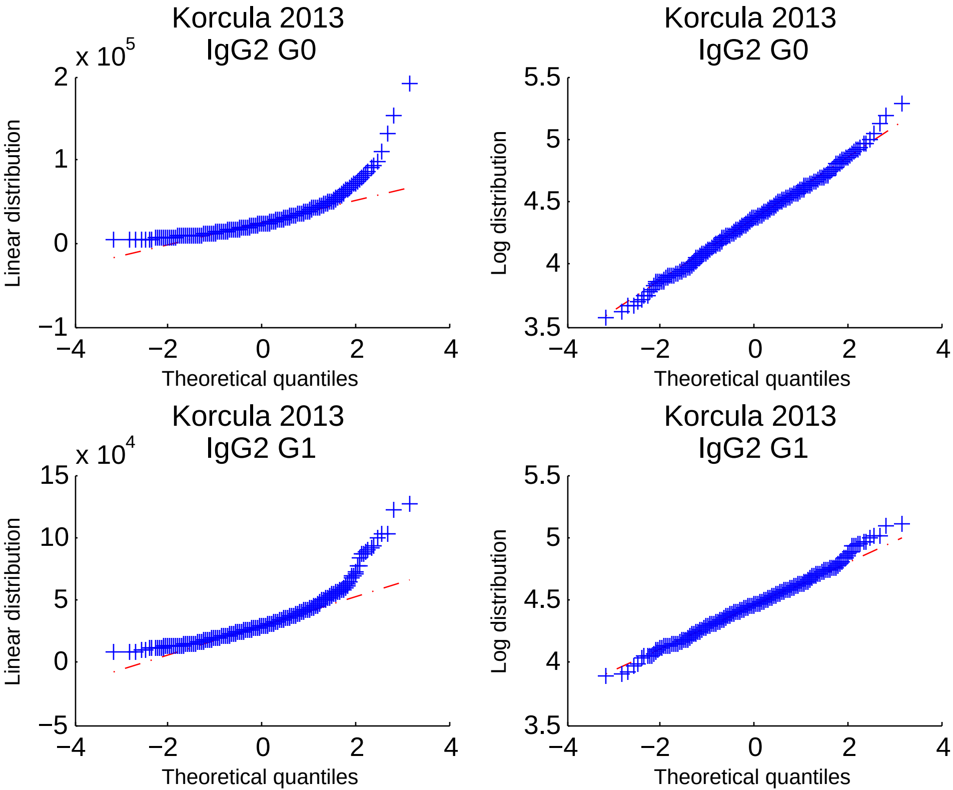

Korcula 2013
IgG2 G0
x 10^5
Linear distribution
Theoretical quantiles
Korcula 2013
IgG2 G0
Log distribution
Theoretical quantiles
Korcula 2013
IgG2 G1
x 10^4
Linear distribution
Theoretical quantiles
Korcula 2013
IgG2 G1
Log distribution
Theoretical quantiles

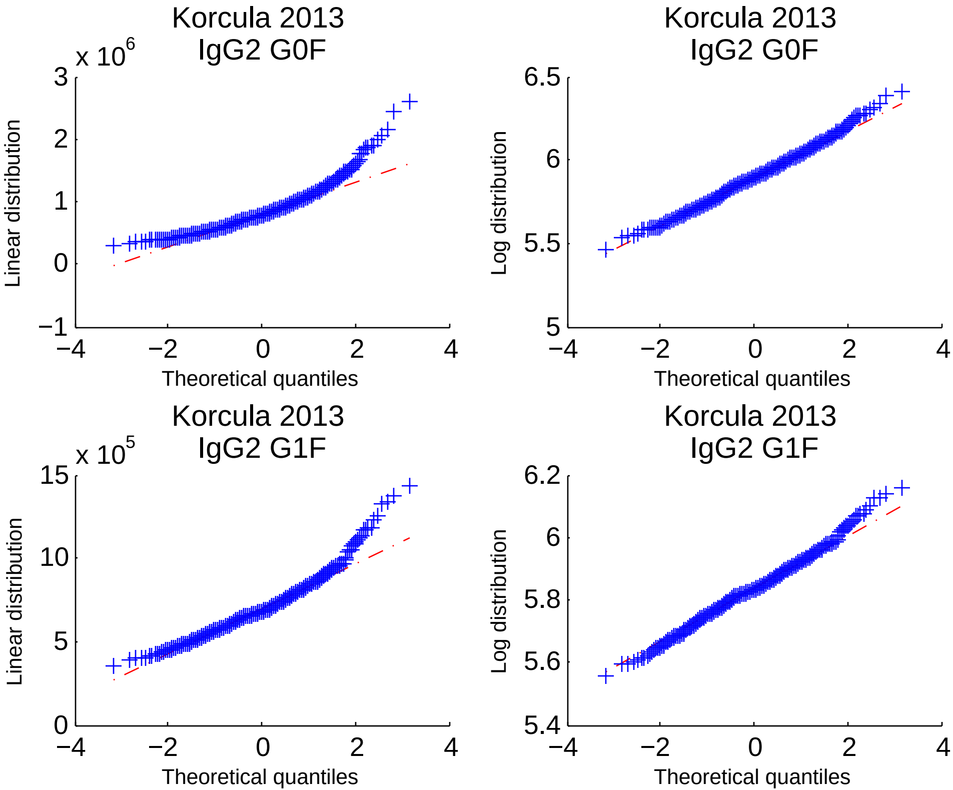

Korcula 2013
IgG2 G0F
x 10^6
Linear distribution
Theoretical quantiles
Korcula 2013
IgG2 G0F
Log distribution
Theoretical quantiles
Korcula 2013
IgG2 G1F
x 10^5
Linear distribution
Theoretical quantiles
Korcula 2013
IgG2 G1F
Log distribution
Theoretical quantiles

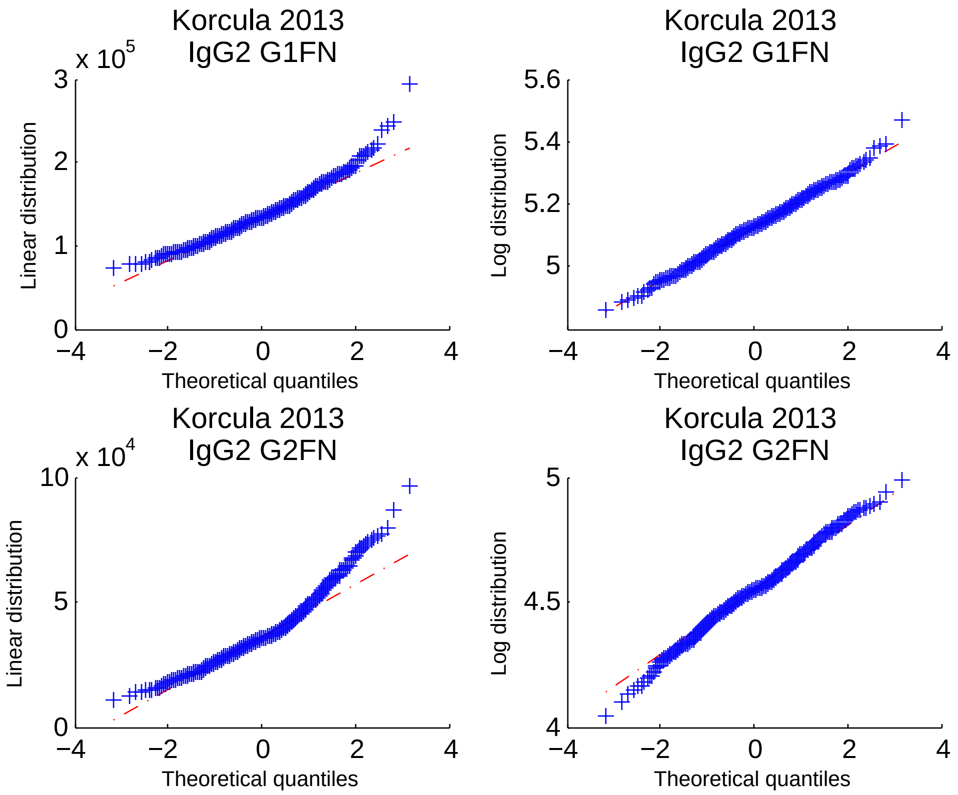

Korcula 2013
IgG2 G1FN
x 10^5
Linear distribution
Theoretical quantiles
Korcula 2013
IgG2 G1FN
Log distribution
Theoretical quantiles
Korcula 2013
IgG2 G2FN
x 10^4
Linear distribution
Theoretical quantiles
Korcula 2013
IgG2 G2FN
Log distribution
Theoretical quantiles

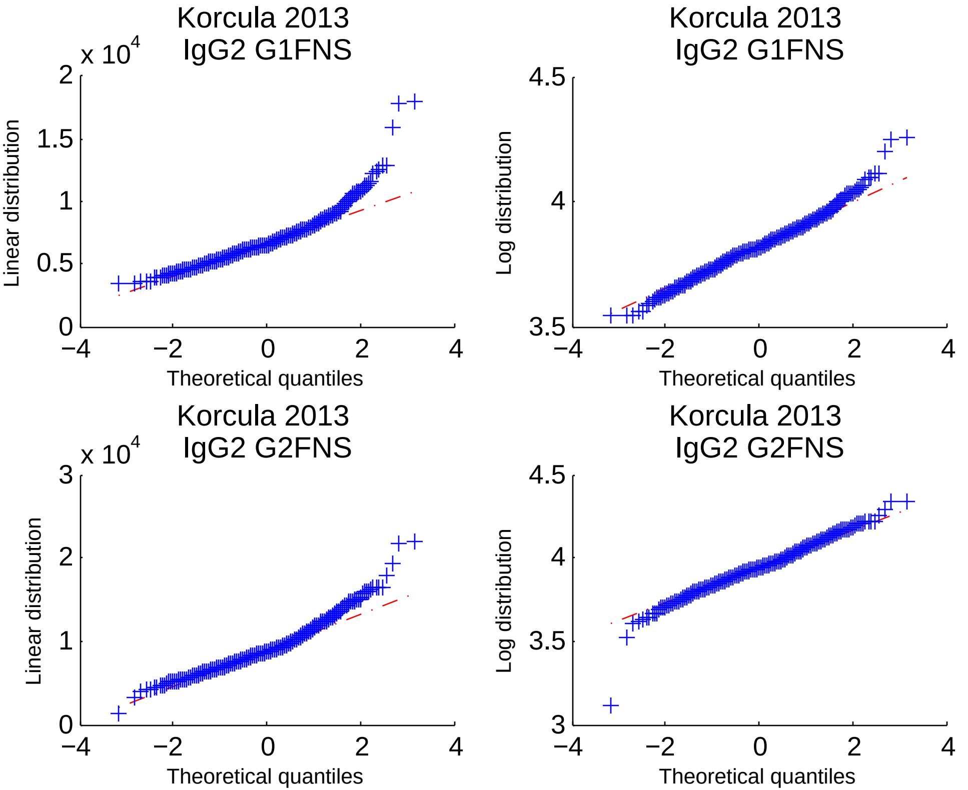

Korcula 2013
IgG2 G1FNS
x 10^4
2
1.5
1
0.5
0
Linear distribution
−4
−2
0
2
4
Theoretical quantiles
Korcula 2013
IgG2 G1FNS
4.5
4
3.5
Log distribution
−4
−2
0
2
4
Theoretical quantiles
Korcula 2013
IgG2 G2FNS
x 10^4
3
2
1
0
Linear distribution
−4
−2
0
2
4
Theoretical quantiles
Korcula 2013
IgG2 G2FNS
4.5
4
3.5
3
Log distribution
−4
−2
0
2
4
Theoretical quantiles

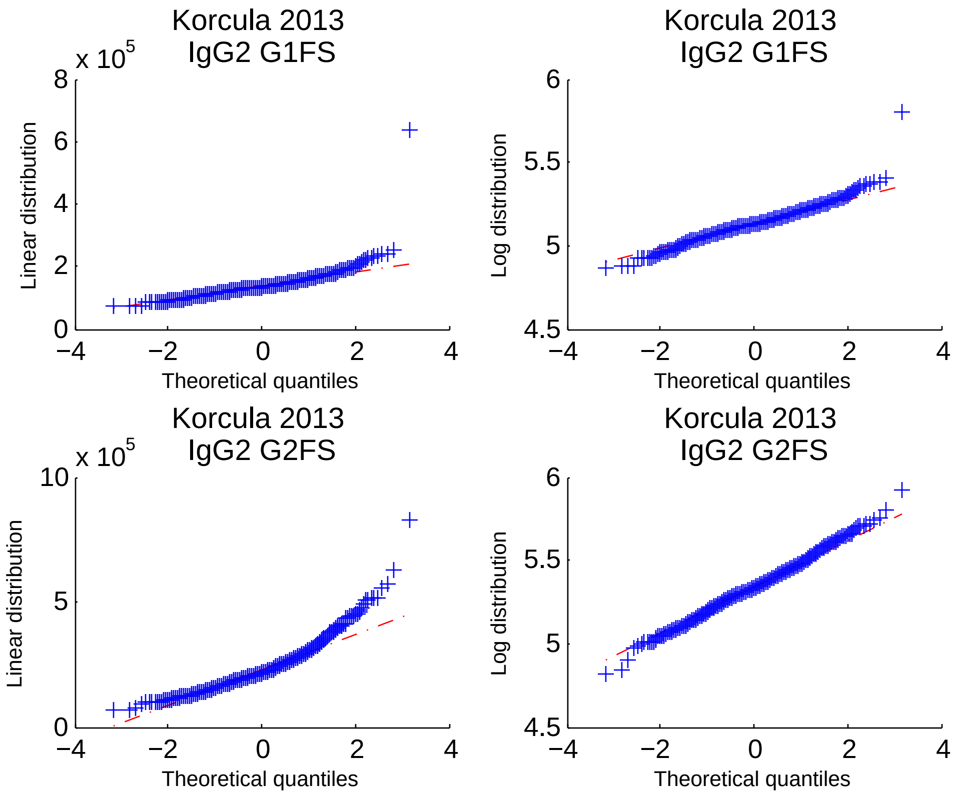
Korcula 2013
IgG2 G1FS
x 10^5
Linear distribution
Theoretical quantiles
Korcula 2013
IgG2 G1FS
Log distribution
Theoretical quantiles
Korcula 2013
IgG2 G2FS
x 10^5
Linear distribution
Theoretical quantiles
Korcula 2013
IgG2 G2FS
Log distribution
Theoretical quantiles

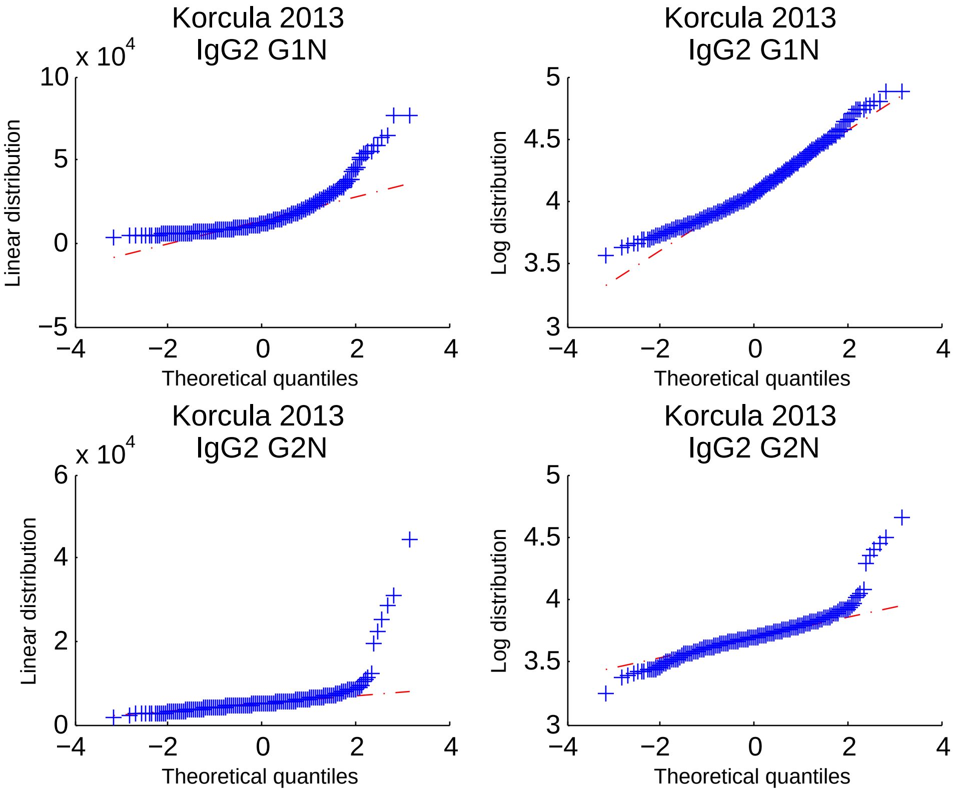

Korcula 2013
IgG2 G1N
x 10^4
Linear distribution
Theoretical quantiles
Korcula 2013
IgG2 G1N
Log distribution
Theoretical quantiles
Korcula 2013
IgG2 G2N
x 10^4
Linear distribution
Theoretical quantiles
Korcula 2013
IgG2 G2N
Log distribution
Theoretical quantiles

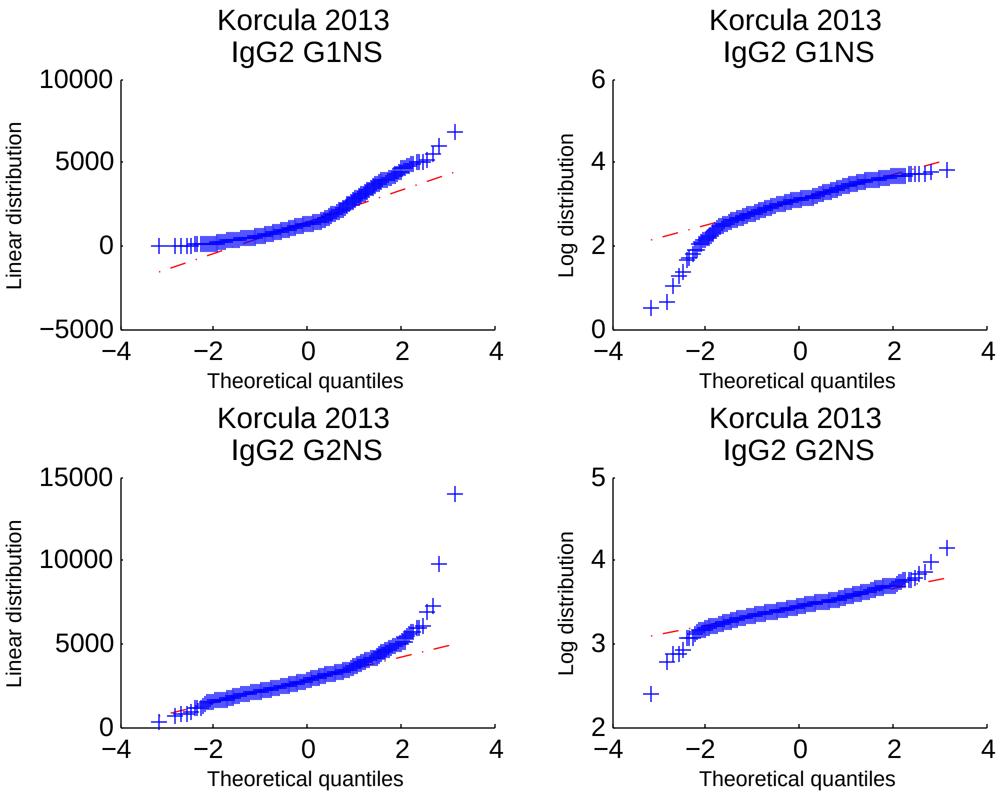
Korcula 2013
IgG2 G1NS
Linear distribution
Theoretical quantiles
10000
5000
0
−5000
−4
−2
0
2
4
Korcula 2013
IgG2 G1NS
Log distribution
Theoretical quantiles
6
4
2
0
Korcula 2013
IgG2 G2NS
Linear distribution
Theoretical quantiles
15000
10000
5000
0
Korcula 2013
IgG2 G2NS
Log distribution
Theoretical quantiles
5
4
3
2

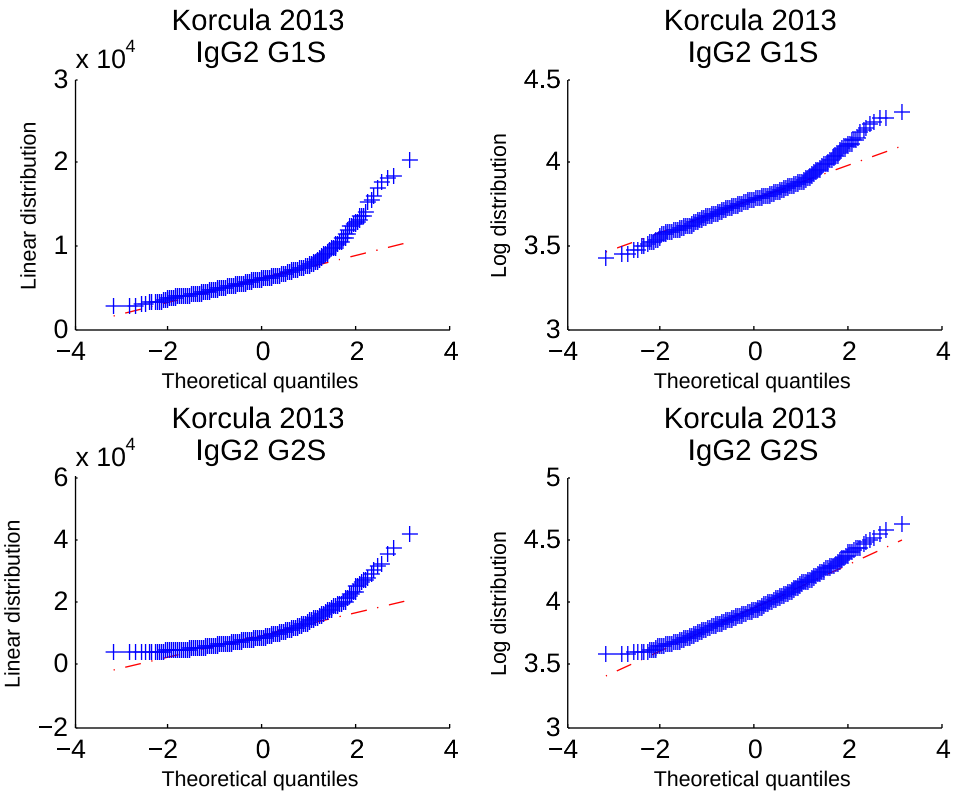

Korcula 2013
IgG2 G1S
x 10^4
Linear distribution
Theoretical quantiles
Korcula 2013
IgG2 G1S
Log distribution
Theoretical quantiles
Korcula 2013
IgG2 G2S
x 10^4
Linear distribution
Theoretical quantiles
Korcula 2013
IgG2 G2S
Log distribution
Theoretical quantiles

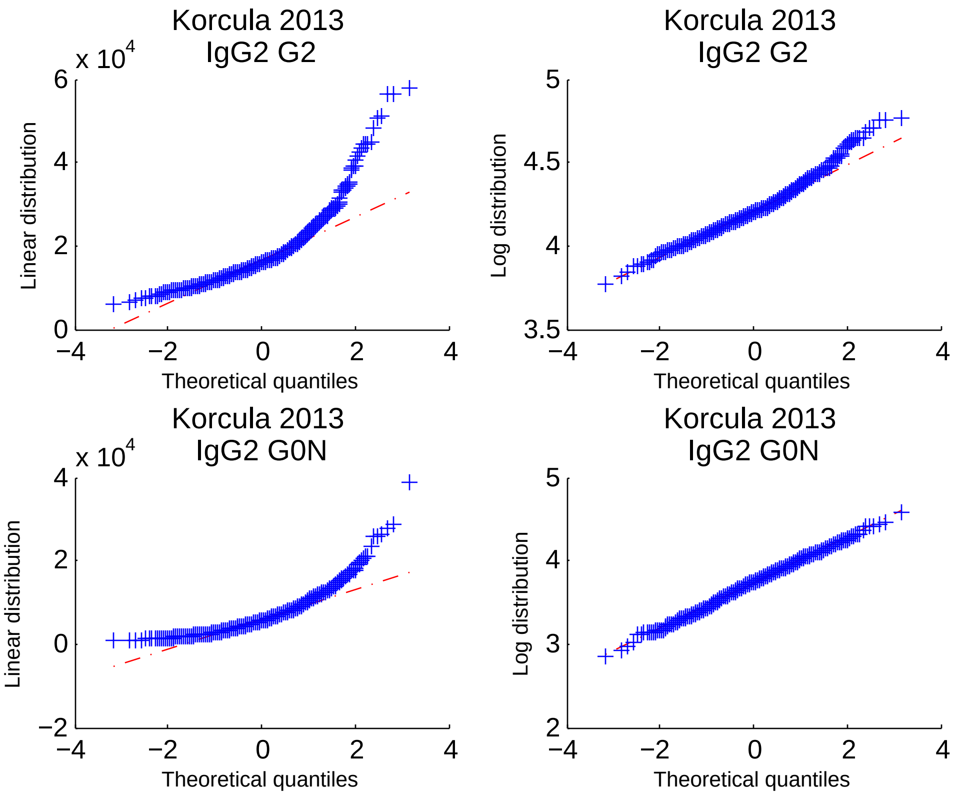

Korcula 2013
IgG2 G2
x 10^4
6
4
2
0
−4
−2
0
2
4
Linear distribution
Theoretical quantiles
Korcula 2013
IgG2 G2
5
4.5
4
3.5
−4
−2
0
2
4
Log distribution
Theoretical quantiles
Korcula 2013
IgG2 G0N
x 10^4
4
2
0
−2
−4
−2
0
2
4
Linear distribution
Theoretical quantiles
Korcula 2013
IgG2 G0N
5
4
3
2
−4
−2
0
2
4
Log distribution
Theoretical quantiles

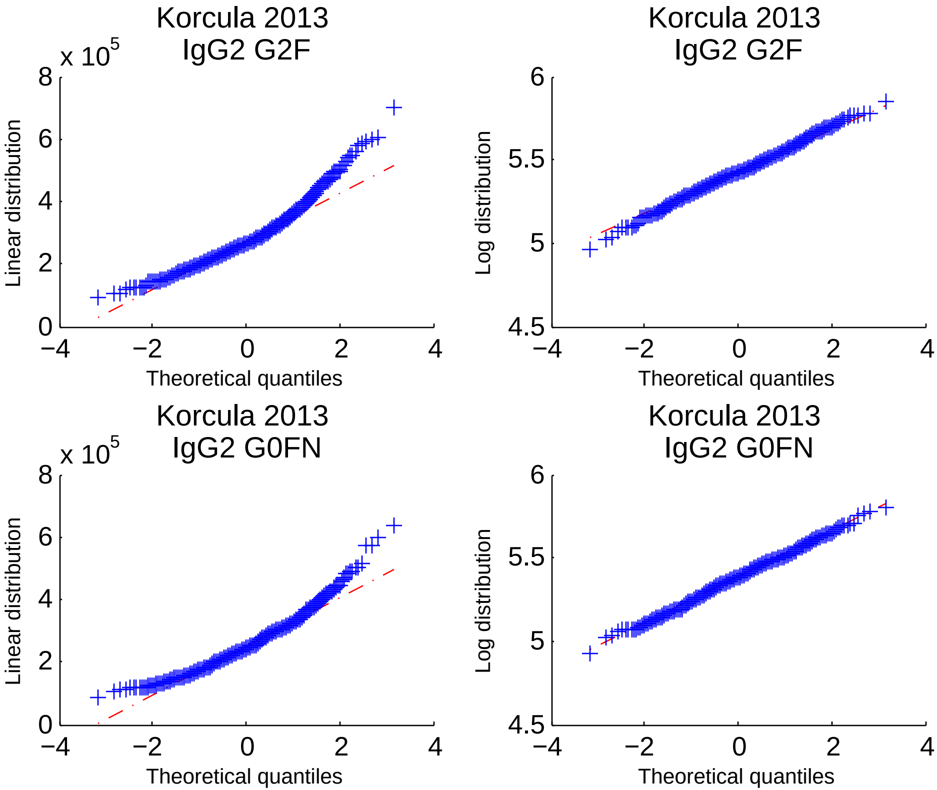
Korcula 2013
IgG2 G2F
x 10^5
Linear distribution
Theoretical quantiles
8
6
4
2
0
−4
−2
0
2
4
Korcula 2013
IgG2 G2F
Log distribution
Theoretical quantiles
6
5.5
5
4.5
−4
−2
0
2
4
Korcula 2013
IgG2 G0FN
x 10^5
Linear distribution
Theoretical quantiles
8
6
4
2
0
−4
−2
0
2
4
Korcula 2013
IgG2 G0FN
Log distribution
Theoretical quantiles
6
5.5
5
4.5
−4
−2
0
2
4

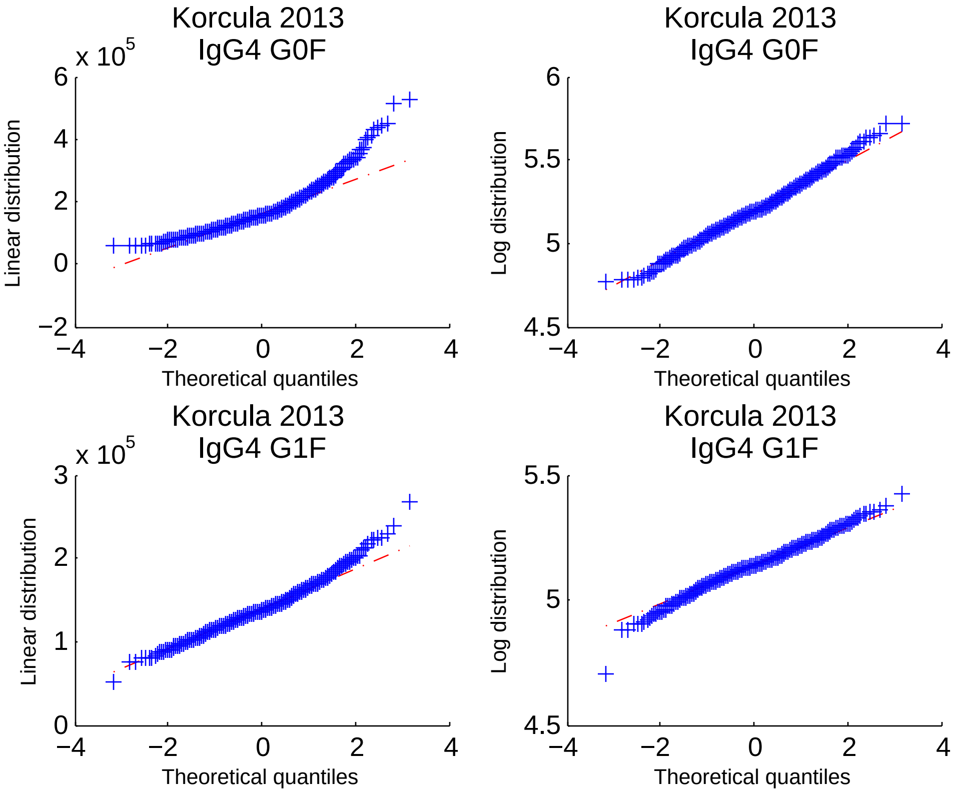

Korcula 2013
IgG4 G0F
x 10^5
Linear distribution
Theoretical quantiles
Korcula 2013
IgG4 G0F
Log distribution
Theoretical quantiles
Korcula 2013
IgG4 G1F
x 10^5
Linear distribution
Theoretical quantiles
Korcula 2013
IgG4 G1F
Log distribution
Theoretical quantiles

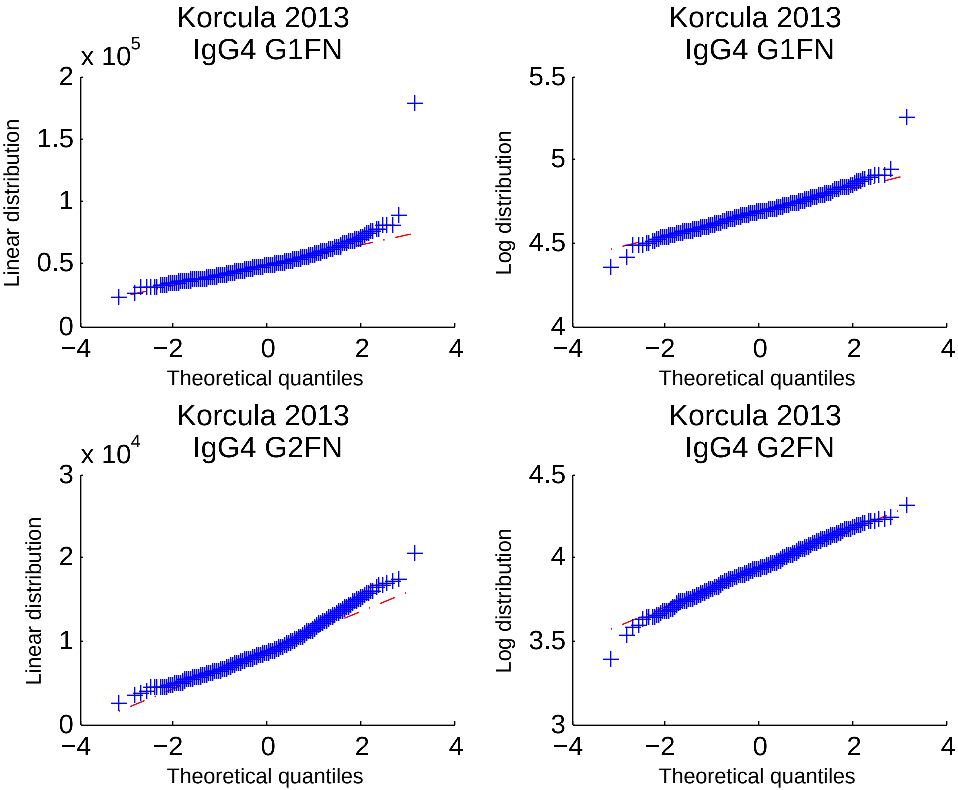
Korcula 2013
IgG4 G1FN
x 10^5
2
1.5
1
0.5
0
Linear distribution
−4
−2
0
2
4
Theoretical quantiles
Korcula 2013
IgG4 G1FN
5.5
5
4.5
4
Log distribution
−4
−2
0
2
4
Theoretical quantiles
Korcula 2013
IgG4 G2FN
x 10^4
3
2
1
0
Linear distribution
−4
−2
0
2
4
Theoretical quantiles
Korcula 2013
IgG4 G2FN
4.5
4
3.5
3
Log distribution
−4
−2
0
2
4
Theoretical quantiles

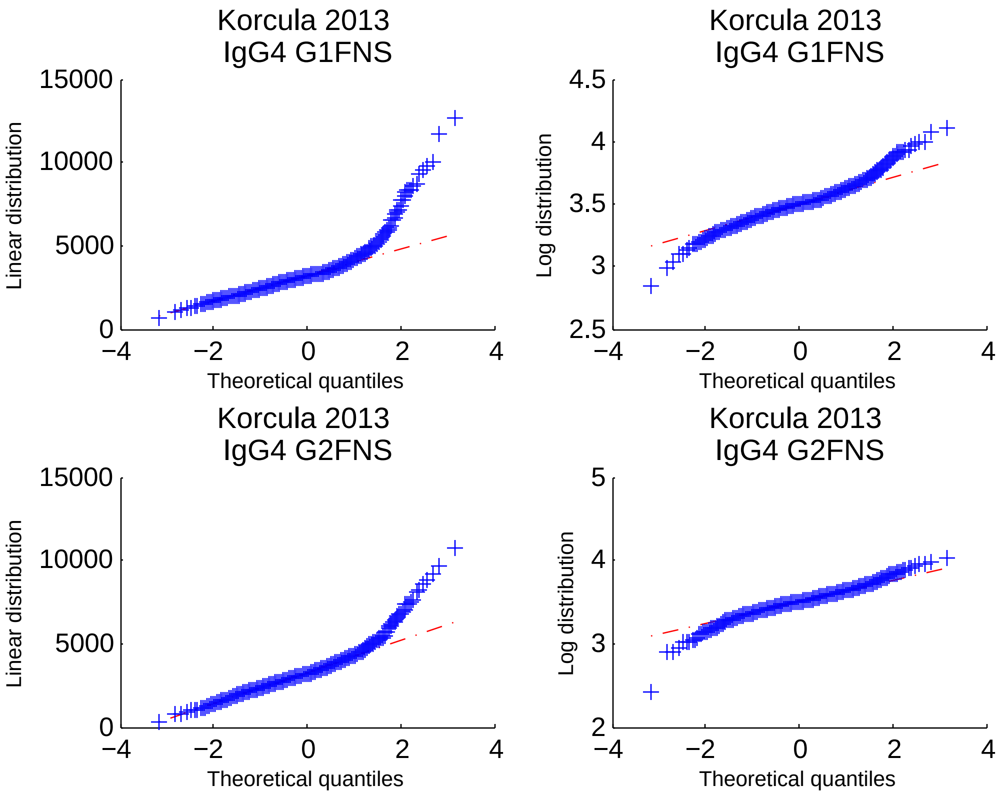
Korcula 2013
IgG4 G1FNS
Linear distribution
15000
10000
5000
0
−4
−2
0
2
4
Theoretical quantiles
Korcula 2013
IgG4 G1FNS
Log distribution
4.5
4
3.5
3
2.5
−4
−2
0
2
4
Theoretical quantiles
Korcula 2013
IgG4 G2FNS
Linear distribution
15000
10000
5000
0
−4
−2
0
2
4
Theoretical quantiles
Korcula 2013
IgG4 G2FNS
Log distribution
5
4
3
2
−4
−2
0
2
4
Theoretical quantiles

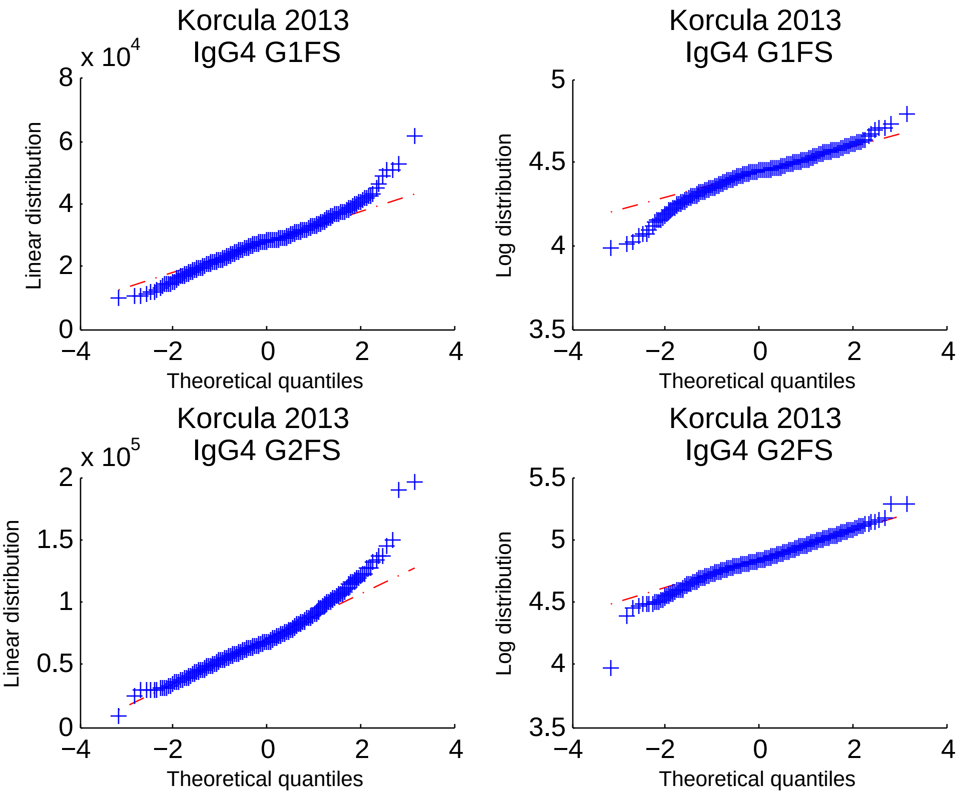
Korcula 2013
IgG4 G1FS
x 10^4
Linear distribution
Theoretical quantiles
Korcula 2013
IgG4 G1FS
Log distribution
Theoretical quantiles
Korcula 2013
IgG4 G2FS
x 10^5
Linear distribution
Theoretical quantiles
Korcula 2013
IgG4 G2FS
Log distribution
Theoretical quantiles

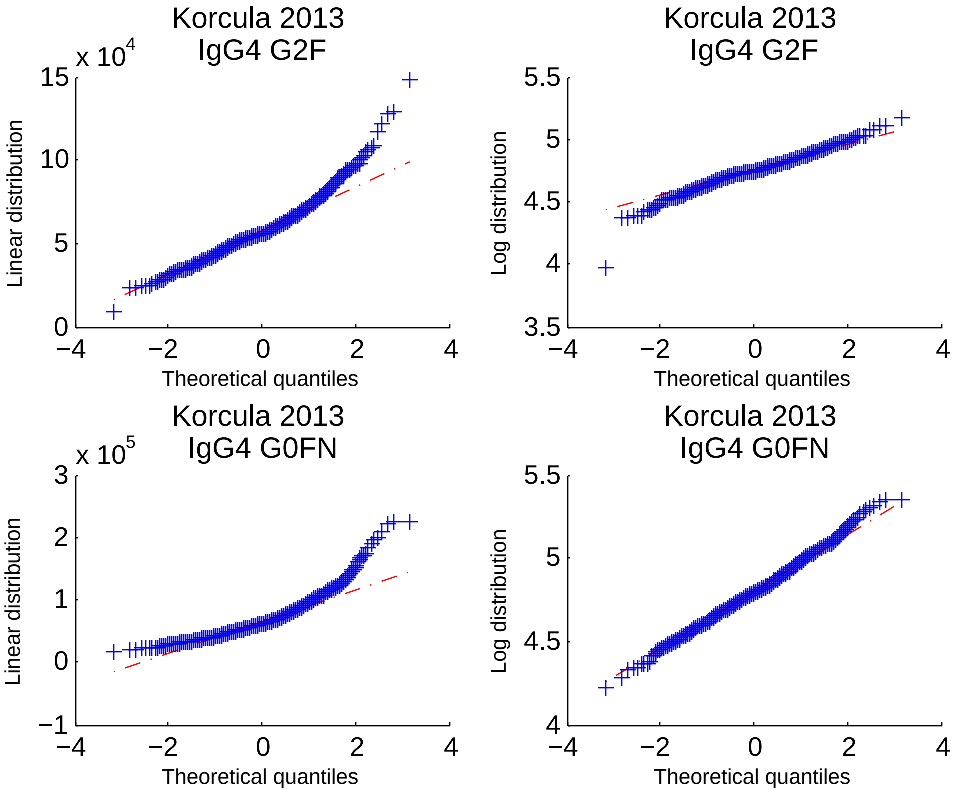

Korcula 2013
IgG4 G2F
x 10^4
Linear distribution
Theoretical quantiles
Korcula 2013
IgG4 G2F
Log distribution
Theoretical quantiles
Korcula 2013
IgG4 G0FN
x 10^5
Linear distribution
Theoretical quantiles
Korcula 2013
IgG4 G0FN
Log distribution
Theoretical quantiles

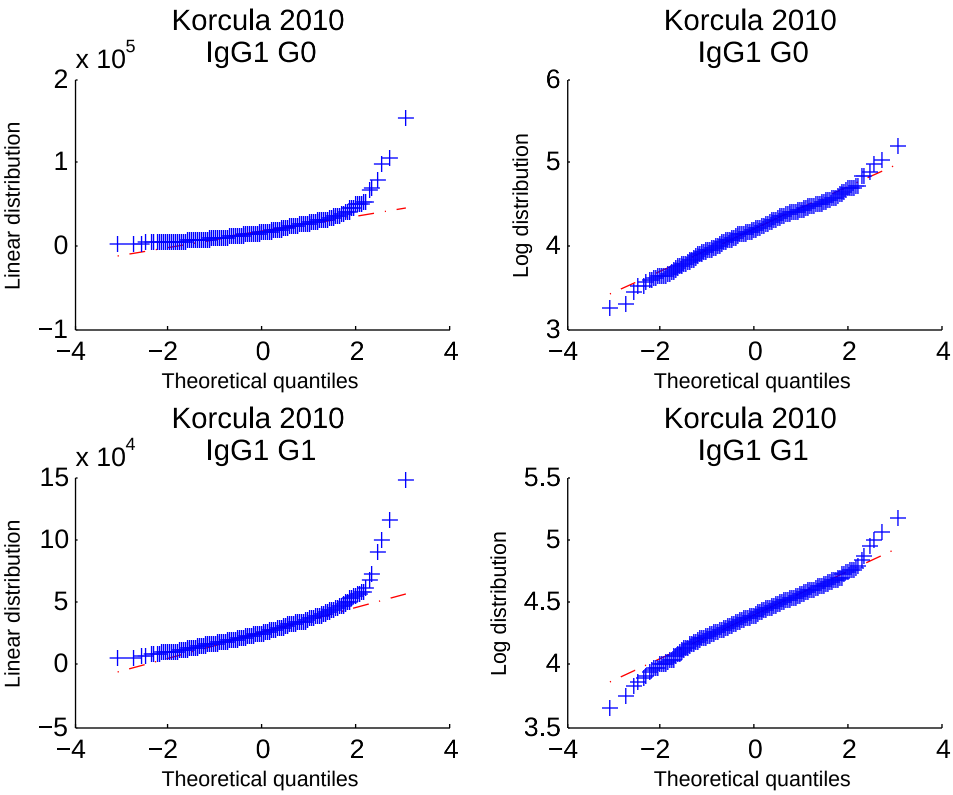
Korcula 2010
IgG1 G0
x 10^5
Linear distribution
Theoretical quantiles
Korcula 2010
IgG1 G0
Log distribution
Theoretical quantiles
Korcula 2010
IgG1 G1
x 10^4
Linear distribution
Theoretical quantiles
Korcula 2010
IgG1 G1
Log distribution
Theoretical quantiles

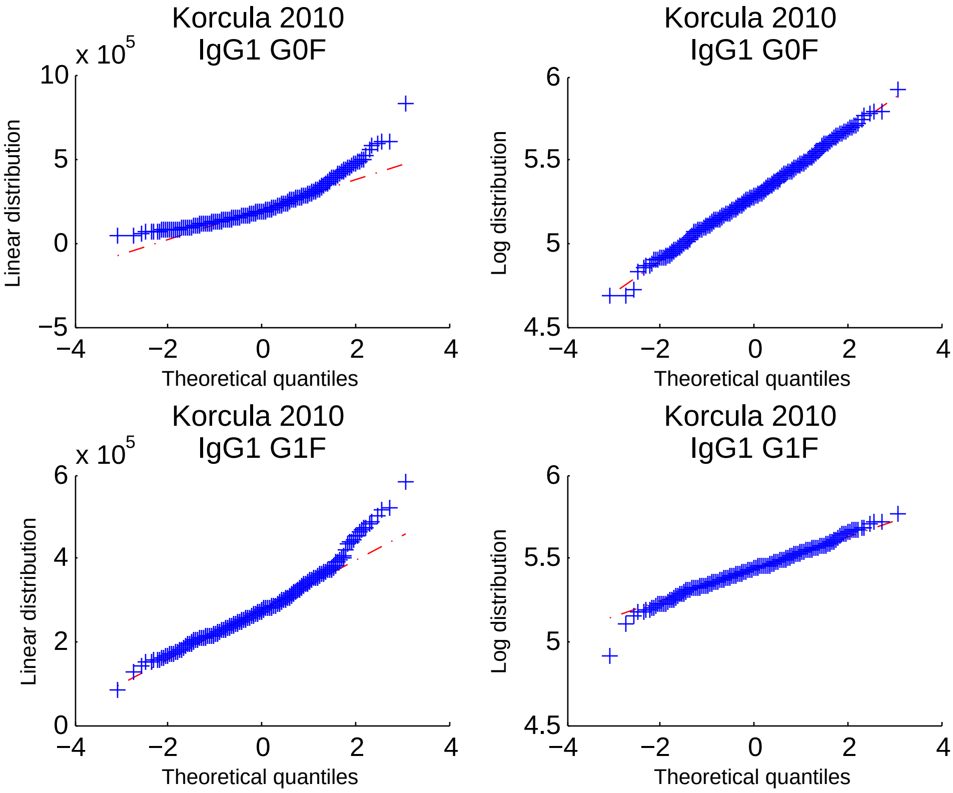

Korcula 2010
IgG1 G0F
x 10^5
Linear distribution
Theoretical quantiles
Korcula 2010
IgG1 G0F
Log distribution
Theoretical quantiles
Korcula 2010
IgG1 G1F
x 10^5
Linear distribution
Theoretical quantiles
Korcula 2010
IgG1 G1F
Log distribution
Theoretical quantiles

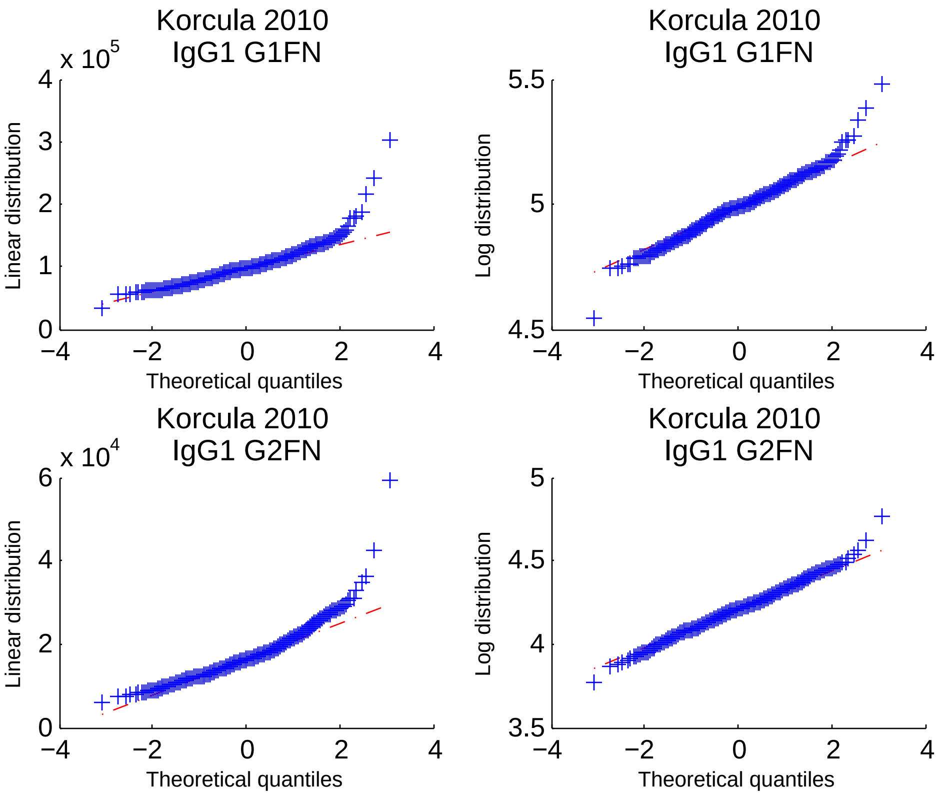

Korcula 2010
IgG1 G1FN
x 10^5
4
3
2
1
0
Linear distribution
−4
−2
0
2
4
Theoretical quantiles
Korcula 2010
IgG1 G1FN
5.5
5
4.5
Log distribution
−4
−2
0
2
4
Theoretical quantiles
Korcula 2010
IgG1 G2FN
x 10^4
6
4
2
0
Linear distribution
−4
−2
0
2
4
Theoretical quantiles
Korcula 2010
IgG1 G2FN
5
4.5
4
3.5
Log distribution
−4
−2
0
2
4
Theoretical quantiles

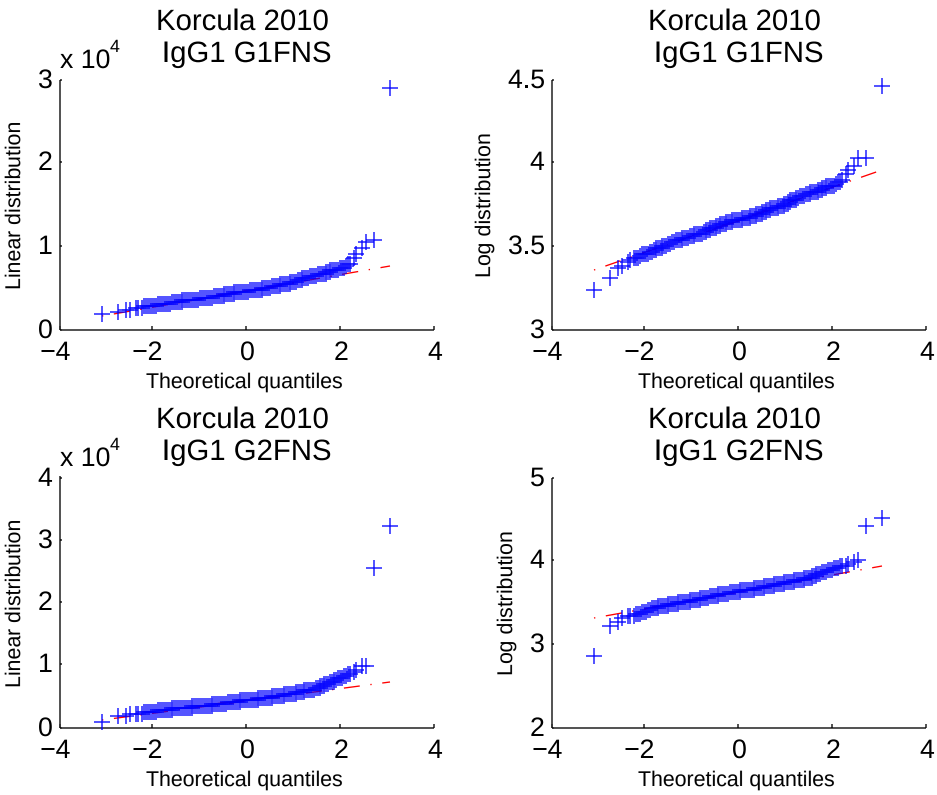
Korcula 2010
IgG1 G1FNS
x 10^4
Linear distribution
Theoretical quantiles
Korcula 2010
IgG1 G1FNS
Log distribution
Theoretical quantiles
Korcula 2010
IgG1 G2FNS
x 10^4
Linear distribution
Theoretical quantiles
Korcula 2010
IgG1 G2FNS
Log distribution
Theoretical quantiles

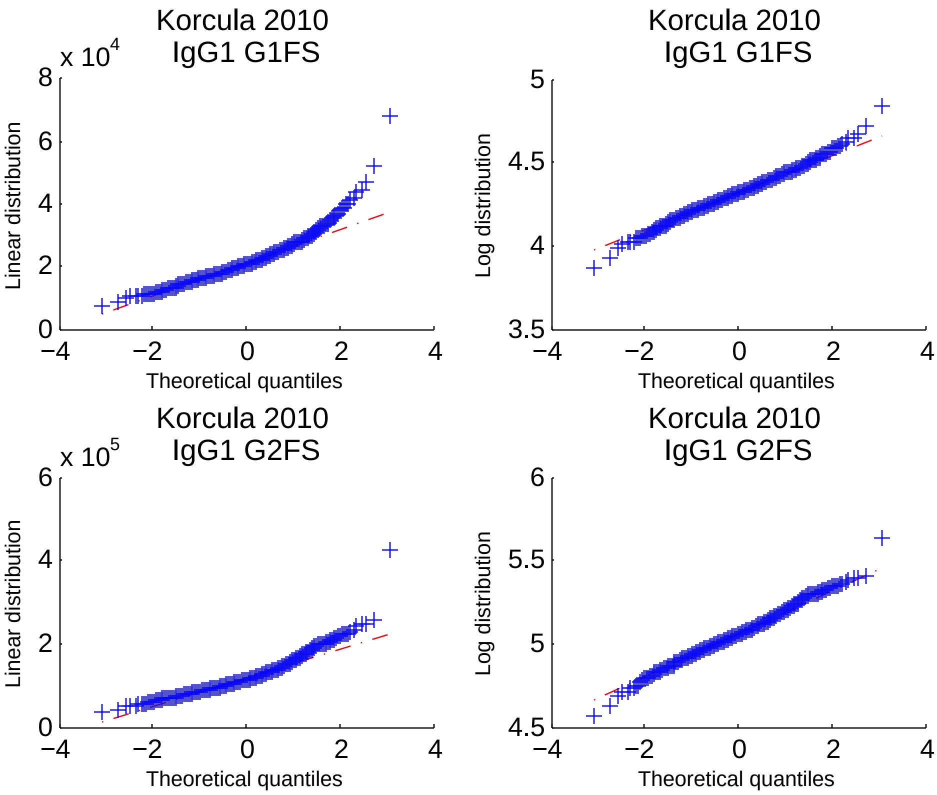
Korcula 2010
IgG1 G1FS
x 10^4
Linear distribution
Theoretical quantiles
Korcula 2010
IgG1 G1FS
Log distribution
Theoretical quantiles
Korcula 2010
IgG1 G2FS
x 10^5
Linear distribution
Theoretical quantiles
Korcula 2010
IgG1 G2FS
Log distribution
Theoretical quantiles

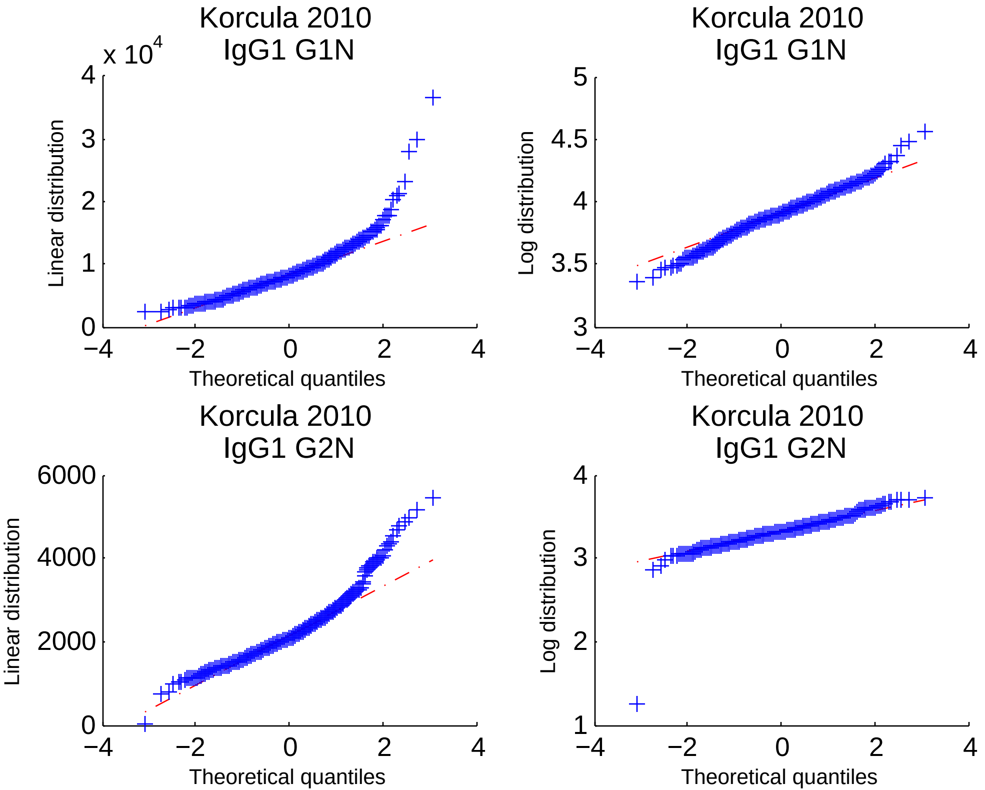
Korcula 2010
IgG1 G1N
x 10^4
4
3
2
1
0
Linear distribution
−4
−2
0
2
4
Theoretical quantiles
Korcula 2010
IgG1 G1N
5
4.5
4
3.5
3
Log distribution
−4
−2
0
2
4
Theoretical quantiles
Korcula 2010
IgG1 G2N
6000
4000
2000
0
Linear distribution
−4
−2
0
2
4
Theoretical quantiles
Korcula 2010
IgG1 G2N
4
3
2
1
Log distribution
−4
−2
0
2
4
Theoretical quantiles

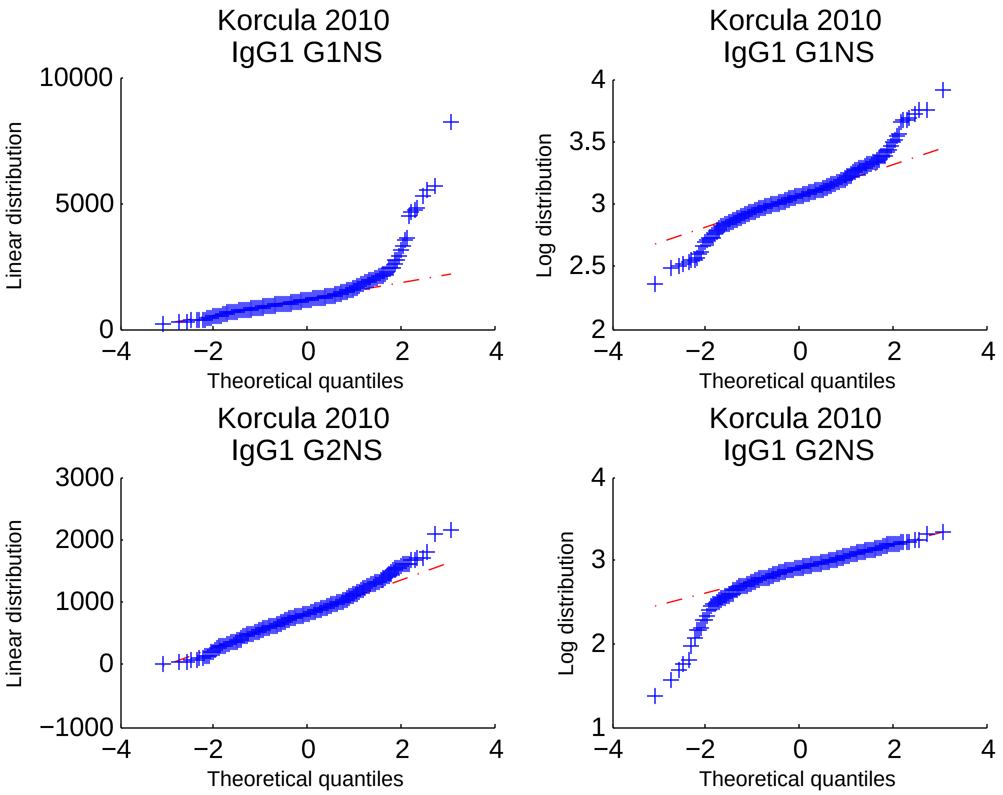
Korcula 2010
IgG1 G1NS
Linear distribution
10000
5000
0
−4
−2
0
2
4
Theoretical quantiles
Korcula 2010
IgG1 G1NS
Log distribution
4
3.5
3
2.5
2
−4
−2
0
2
4
Theoretical quantiles
Korcula 2010
IgG1 G2NS
Linear distribution
3000
2000
1000
0
−1000
−4
−2
0
2
4
Theoretical quantiles
Korcula 2010
IgG1 G2NS
Log distribution
4
3
2
1
−4
−2
0
2
4
Theoretical quantiles

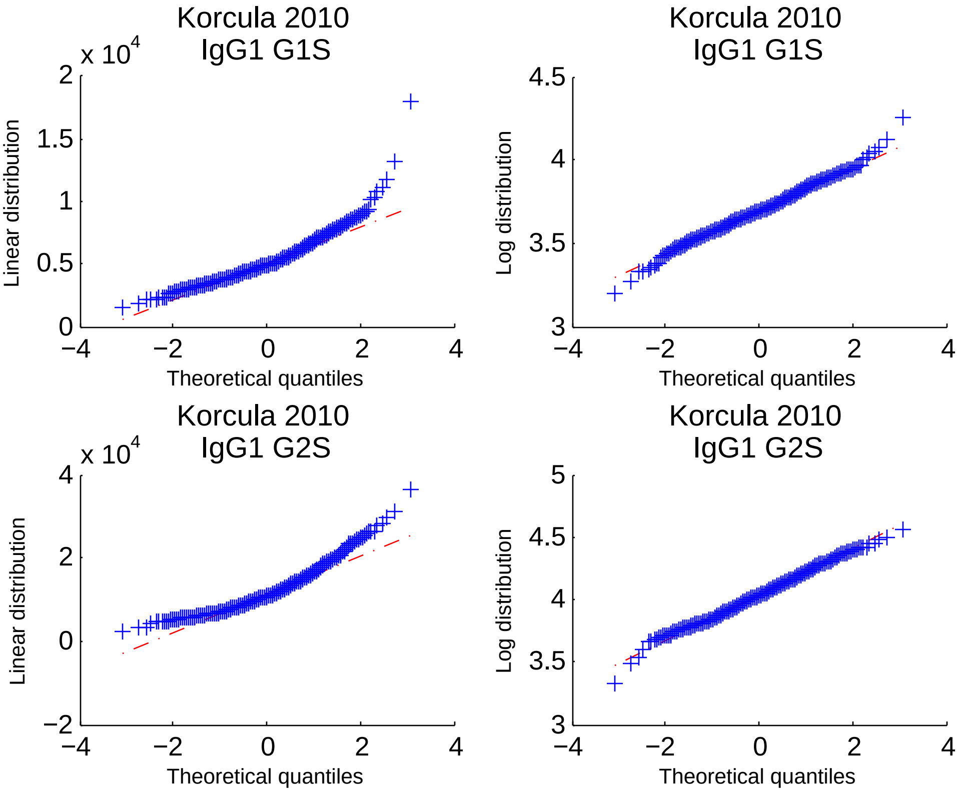
Korcula 2010
IgG1 G1S
x 10^4
Linear distribution
Theoretical quantiles
Korcula 2010
IgG1 G1S
Log distribution
Theoretical quantiles
Korcula 2010
IgG1 G2S
x 10^4
Linear distribution
Theoretical quantiles
Korcula 2010
IgG1 G2S
Log distribution
Theoretical quantiles

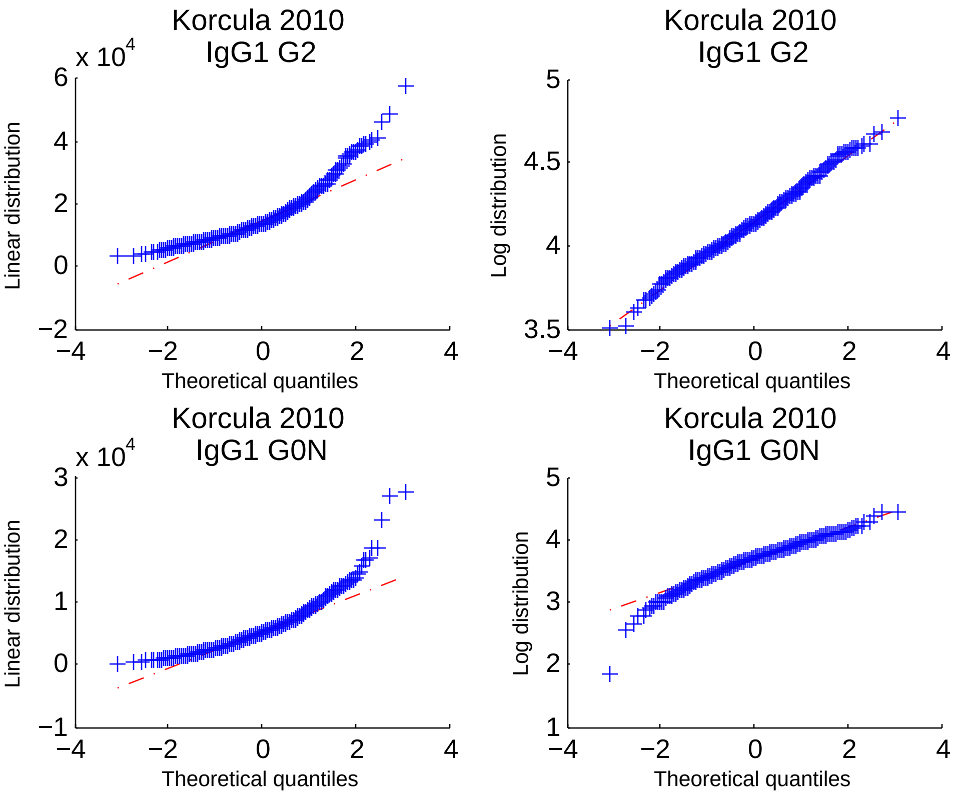

Korcula 2010
IgG1 G2
x 10^4
Linear distribution
Theoretical quantiles
Korcula 2010
IgG1 G2
Log distribution
Theoretical quantiles
Korcula 2010
IgG1 G0N
x 10^4
Linear distribution
Theoretical quantiles
Korcula 2010
IgG1 G0N
Log distribution
Theoretical quantiles

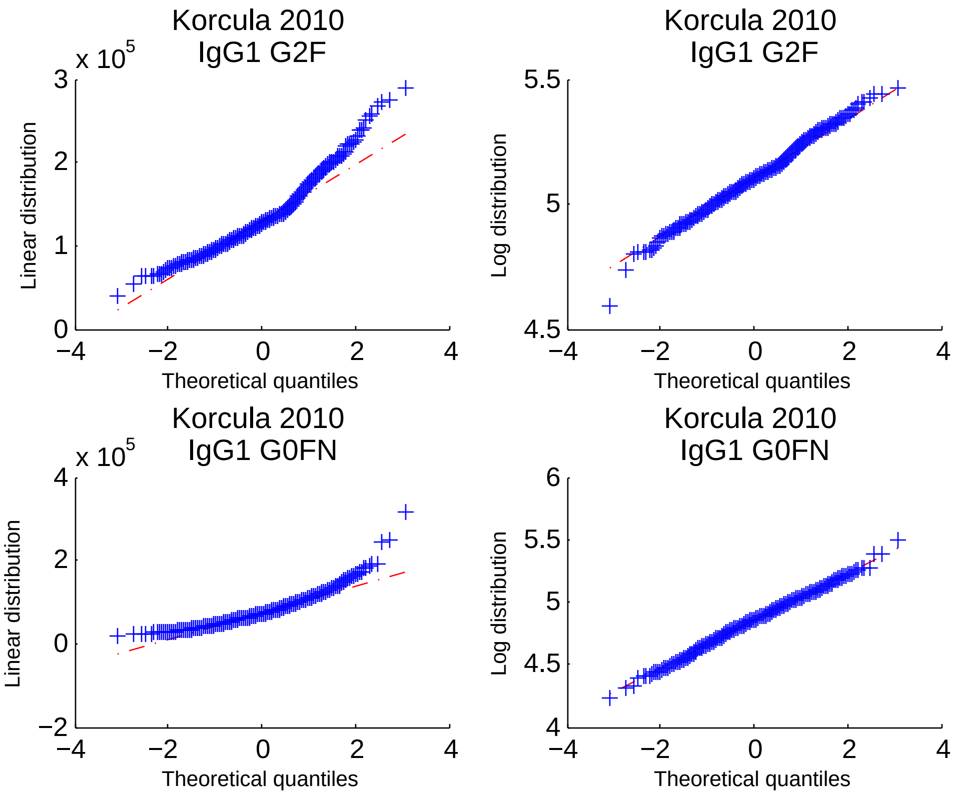
Korcula 2010
IgG1 G2F
x 10^5
Linear distribution
Theoretical quantiles
Korcula 2010
IgG1 G2F
Log distribution
Theoretical quantiles
Korcula 2010
IgG1 G0FN
x 10^5
Linear distribution
Theoretical quantiles
Korcula 2010
IgG1 G0FN
Log distribution
Theoretical quantiles

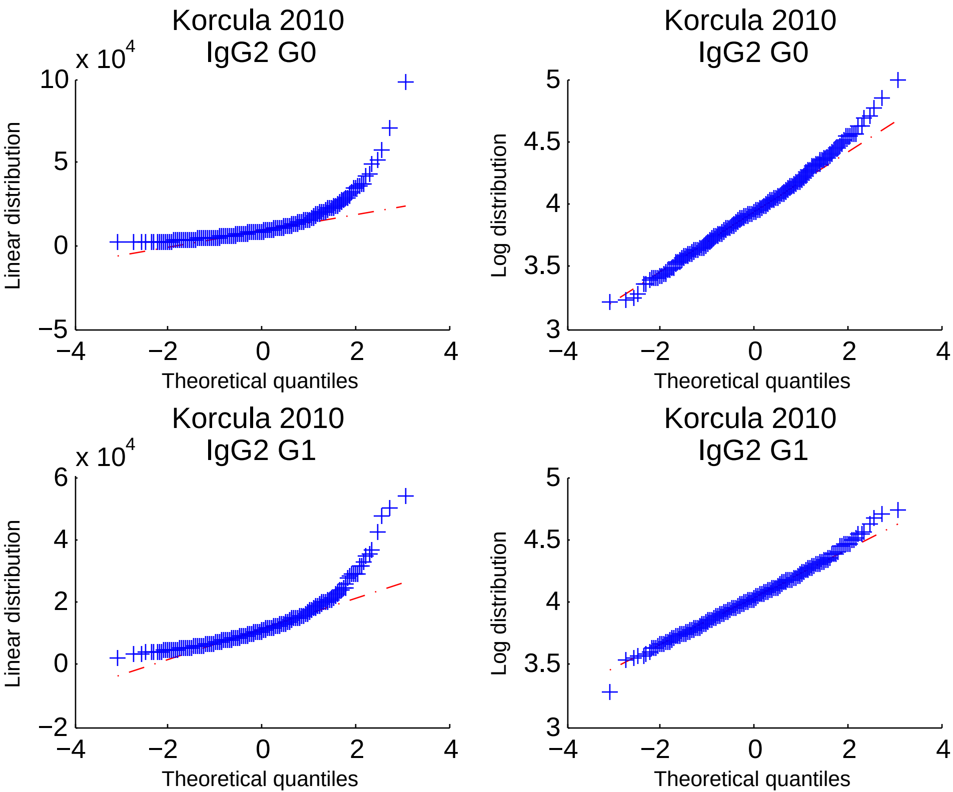
Korcula 2010
IgG2 G0
x 10^4
Linear distribution
Theoretical quantiles
Korcula 2010
IgG2 G0
Log distribution
Theoretical quantiles
Korcula 2010
IgG2 G1
x 10^4
Linear distribution
Theoretical quantiles
Korcula 2010
IgG2 G1
Log distribution
Theoretical quantiles

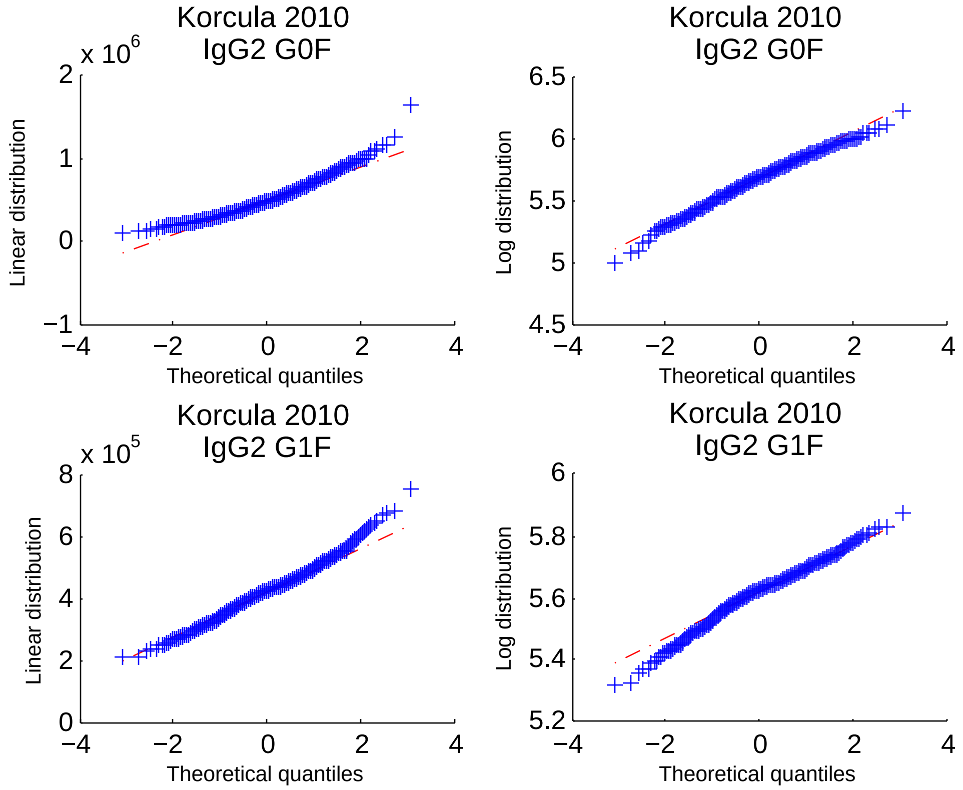
Korcula 2010
IgG2 G0F
x 10^6
2
1
0
−1
Linear distribution
−4
−2
0
2
4
Theoretical quantiles
Korcula 2010
IgG2 G0F
6.5
6
5.5
5
4.5
Log distribution
Theoretical quantiles
Korcula 2010
IgG2 G1F
x 10^5
8
6
4
2
0
Linear distribution
Theoretical quantiles
Korcula 2010
IgG2 G1F
6
5.8
5.6
5.4
5.2
Log distribution
Theoretical quantiles

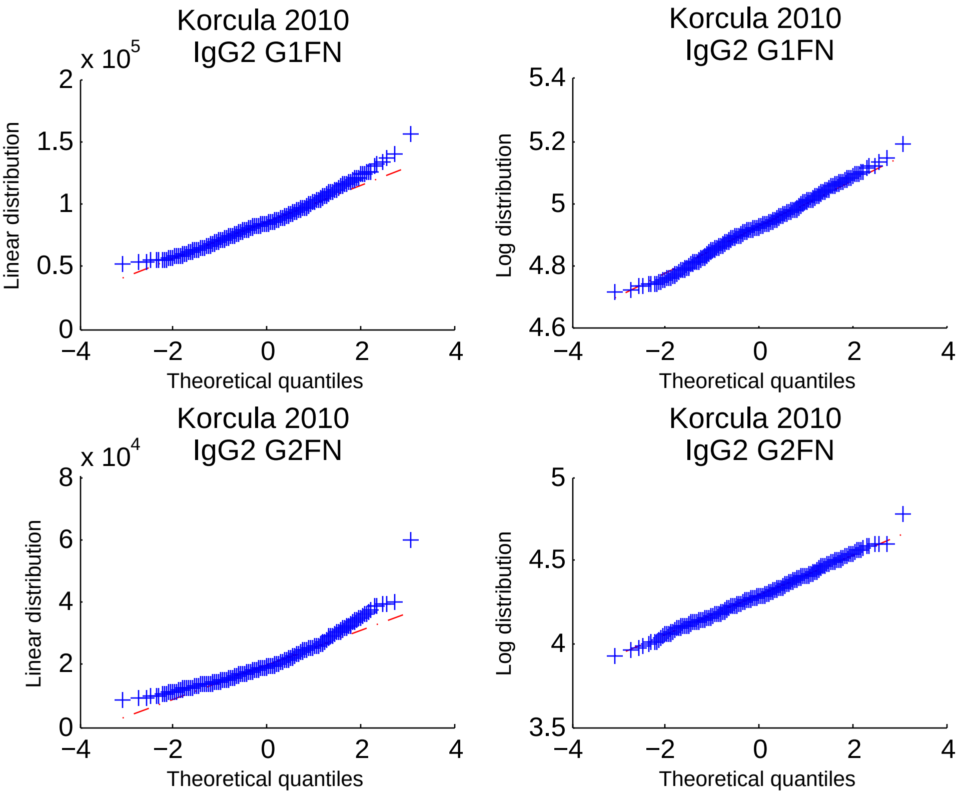
Korcula 2010
IgG2 G1FN
x 10^5
Linear distribution
Theoretical quantiles
Korcula 2010
IgG2 G1FN
Log distribution
Theoretical quantiles
Korcula 2010
IgG2 G2FN
x 10^4
Linear distribution
Theoretical quantiles
Korcula 2010
IgG2 G2FN
Log distribution
Theoretical quantiles

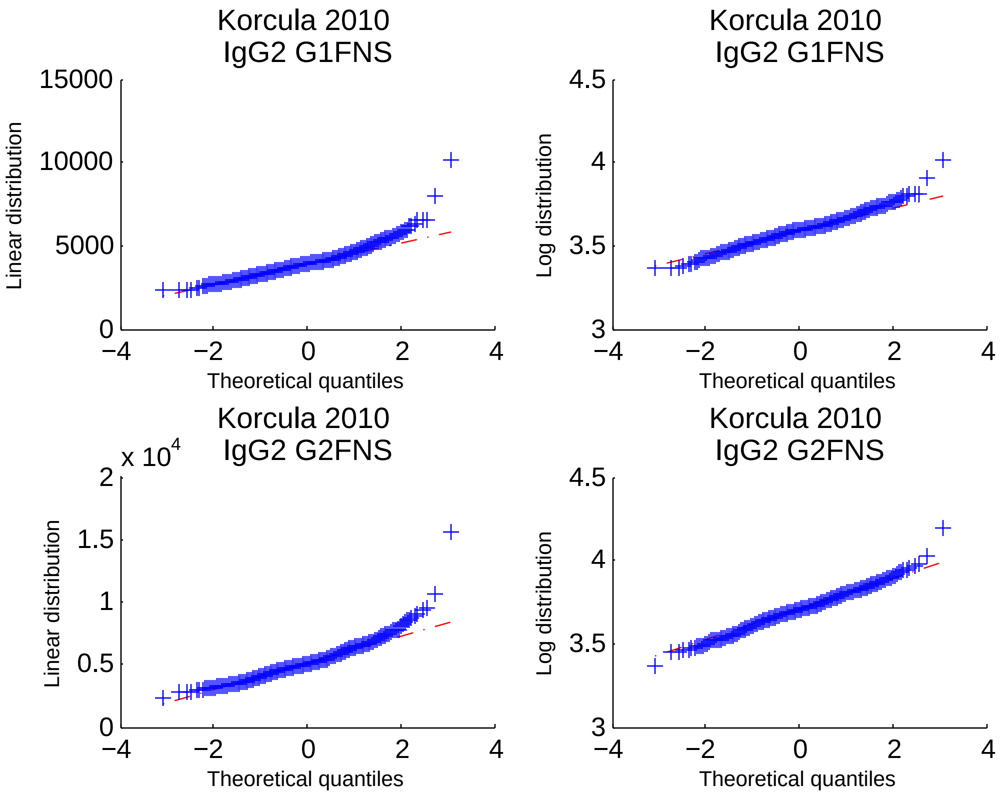

Korcula 2010
IgG2 G1FNS
Linear distribution
Theoretical quantiles
Korcula 2010
IgG2 G1FNS
Log distribution
Theoretical quantiles
Korcula 2010
IgG2 G2FNS
x 10^4
Linear distribution
Theoretical quantiles
Korcula 2010
IgG2 G2FNS
Log distribution
Theoretical quantiles

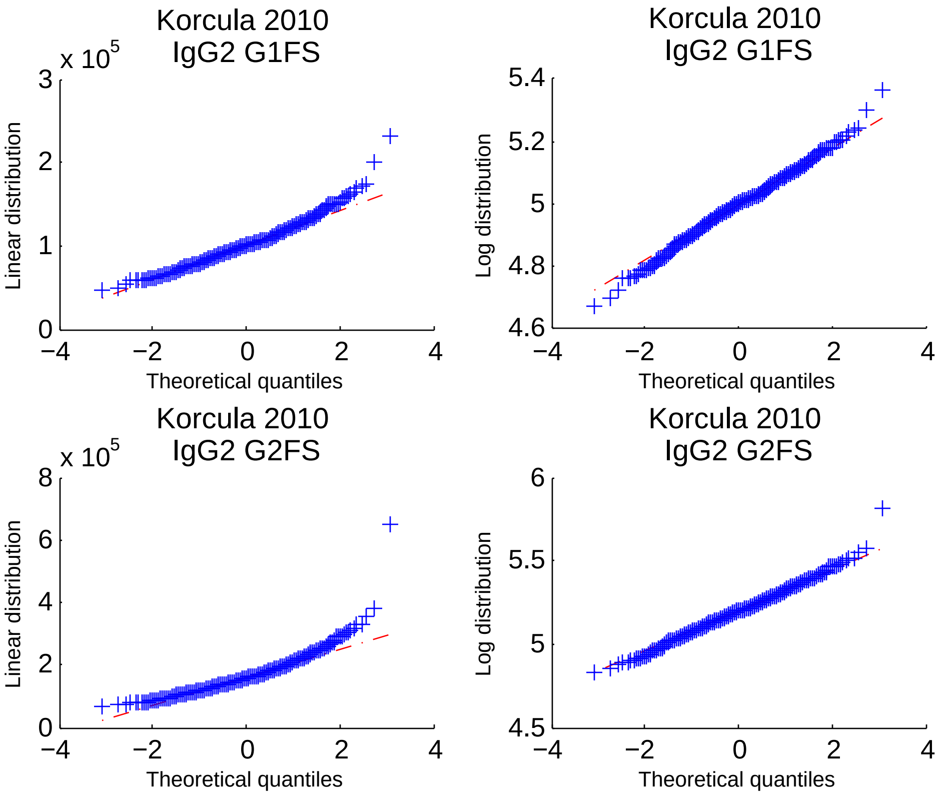

Korcula 2010
IgG2 G1FS
x 10^5
Linear distribution
Theoretical quantiles
Korcula 2010
IgG2 G1FS
Log distribution
Theoretical quantiles
Korcula 2010
IgG2 G2FS
x 10^5
Linear distribution
Theoretical quantiles
Korcula 2010
IgG2 G2FS
Log distribution
Theoretical quantiles

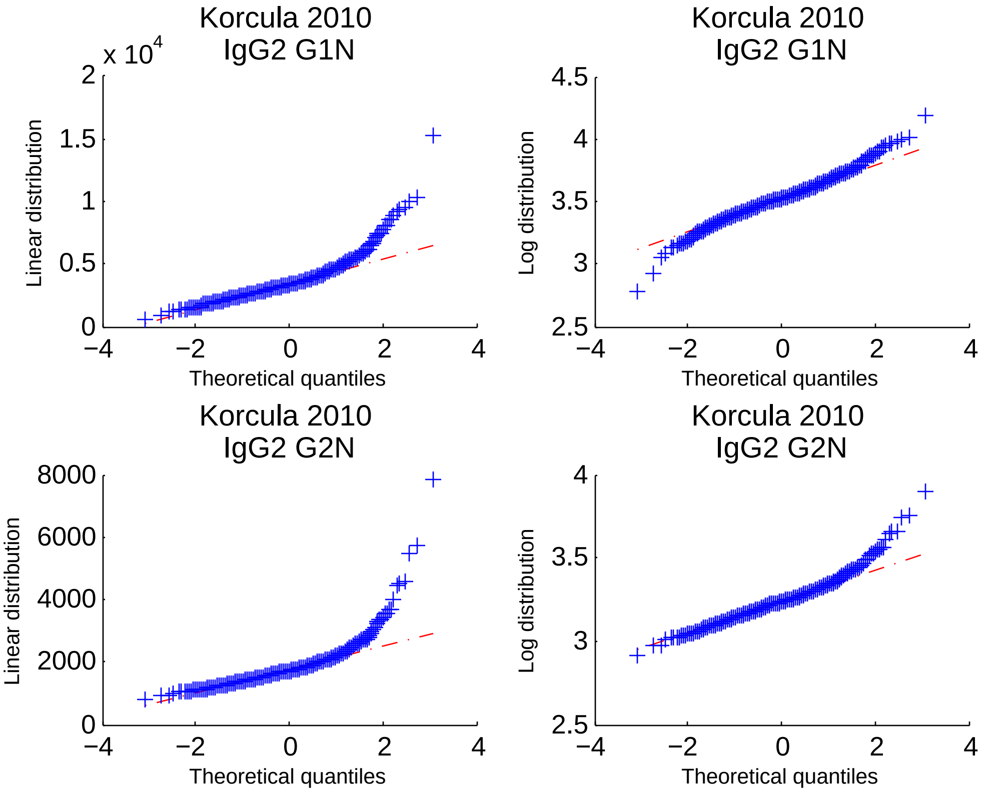

Korcula 2010
IgG2 G1N
x 10^4
2
1.5
1
0.5
0
Linear distribution
−4
−2
0
2
4
Theoretical quantiles
Korcula 2010
IgG2 G1N
4.5
4
3.5
3
2.5
Log distribution
−4
−2
0
2
4
Theoretical quantiles
Korcula 2010
IgG2 G2N
8000
6000
4000
2000
0
Linear distribution
−4
−2
0
2
4
Theoretical quantiles
Korcula 2010
IgG2 G2N
4
3.5
3
2.5
Log distribution
−4
−2
0
2
4
Theoretical quantiles

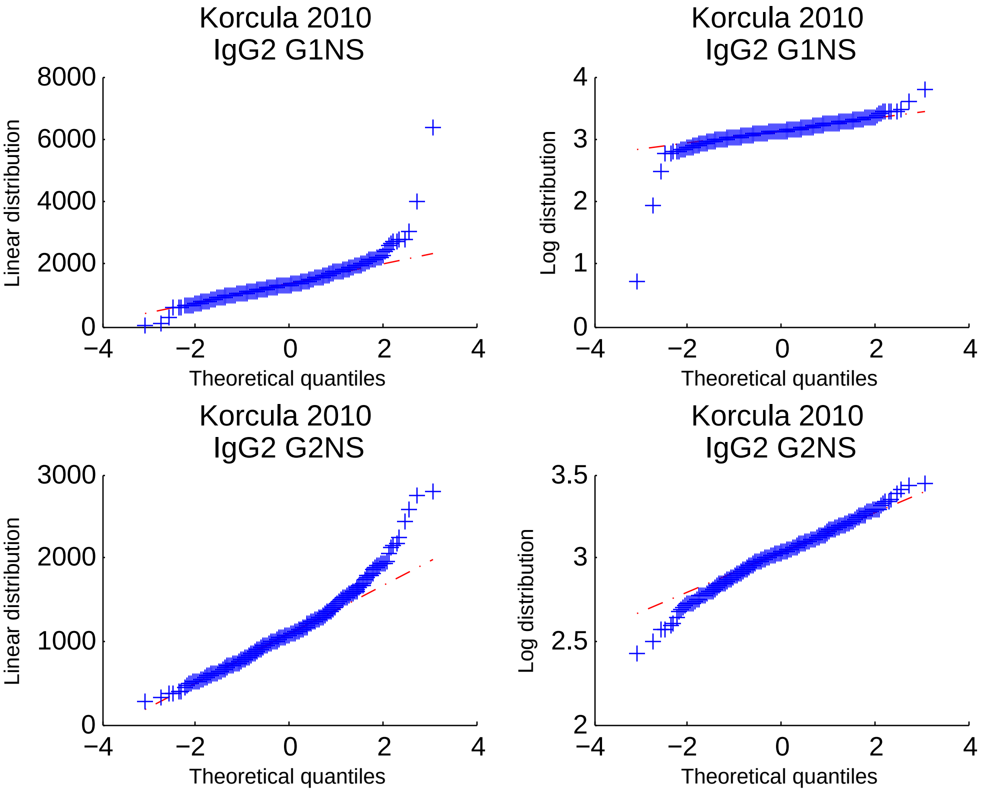
Korcula 2010
IgG2 G1NS
Linear distribution
Theoretical quantiles
8000
6000
4000
2000
0
−4
−2
0
2
4
Korcula 2010
IgG2 G1NS
Log distribution
Theoretical quantiles
4
3
2
1
0
−4
−2
0
2
4
Korcula 2010
IgG2 G2NS
Linear distribution
Theoretical quantiles
3000
2000
1000
0
−4
−2
0
2
4
Korcula 2010
IgG2 G2NS
Log distribution
Theoretical quantiles
3.5
3
2.5
2
−4
−2
0
2
4

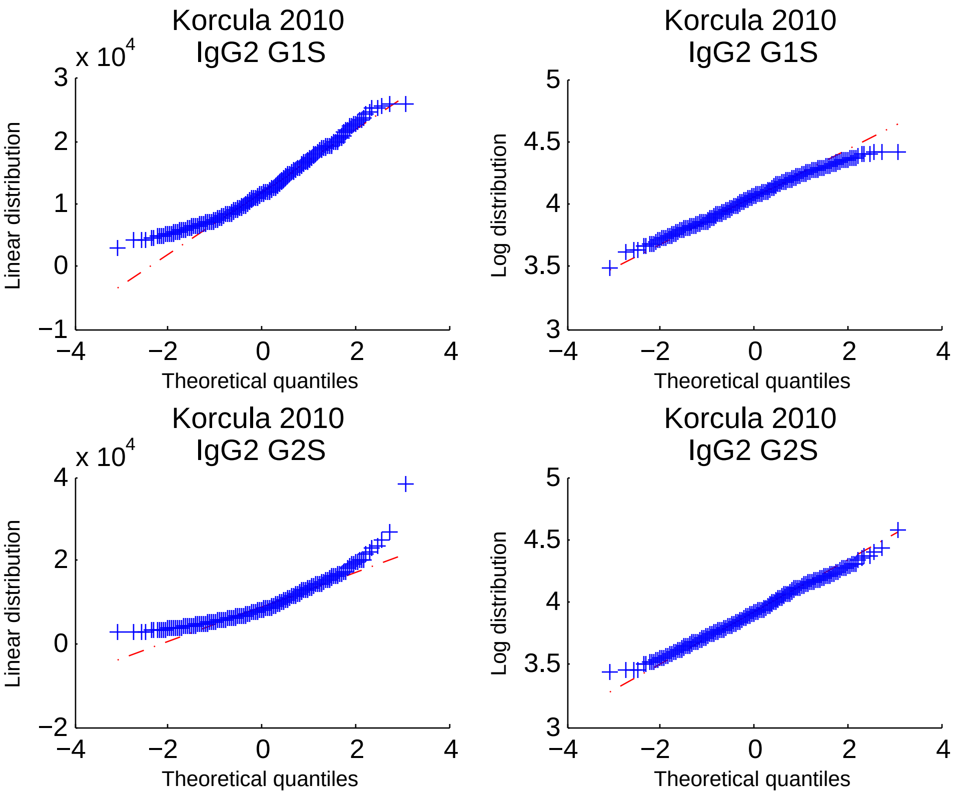
Korcula 2010
IgG2 G1S
x 10^4
Linear distribution
Theoretical quantiles
Korcula 2010
IgG2 G1S
Log distribution
Theoretical quantiles
Korcula 2010
IgG2 G2S
x 10^4
Linear distribution
Theoretical quantiles
Korcula 2010
IgG2 G2S
Log distribution
Theoretical quantiles

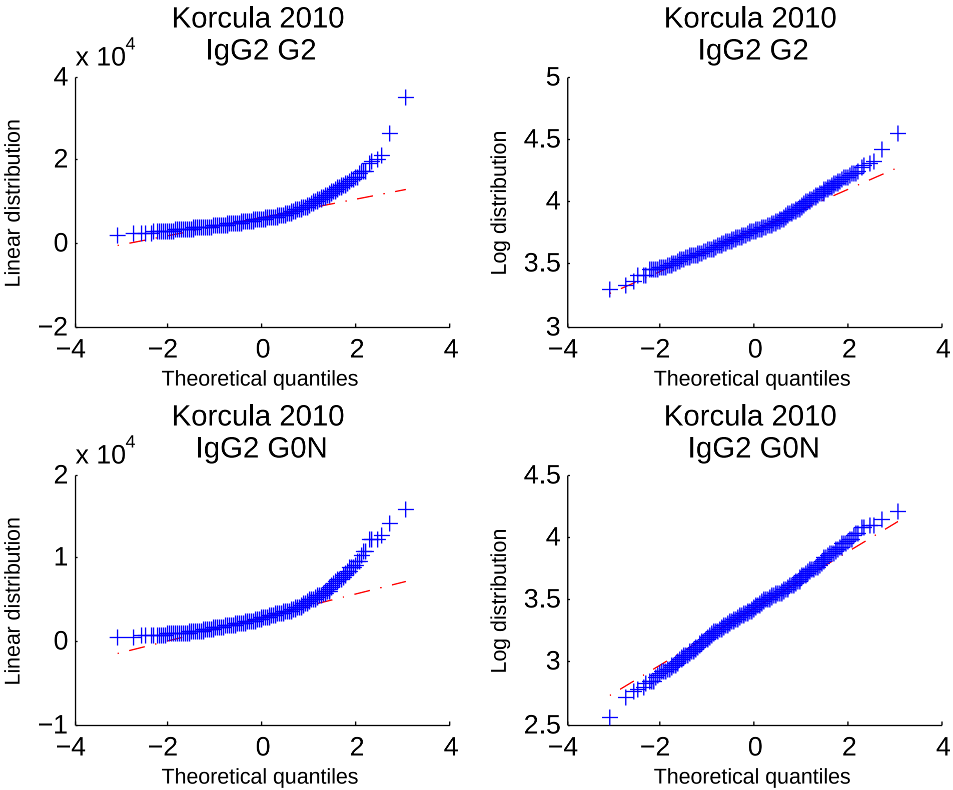

Korcula 2010
IgG2 G2
x 10^4
Linear distribution
Theoretical quantiles
Korcula 2010
IgG2 G2
Log distribution
Theoretical quantiles
Korcula 2010
IgG2 G0N
x 10^4
Linear distribution
Theoretical quantiles
Korcula 2010
IgG2 G0N
Log distribution
Theoretical quantiles

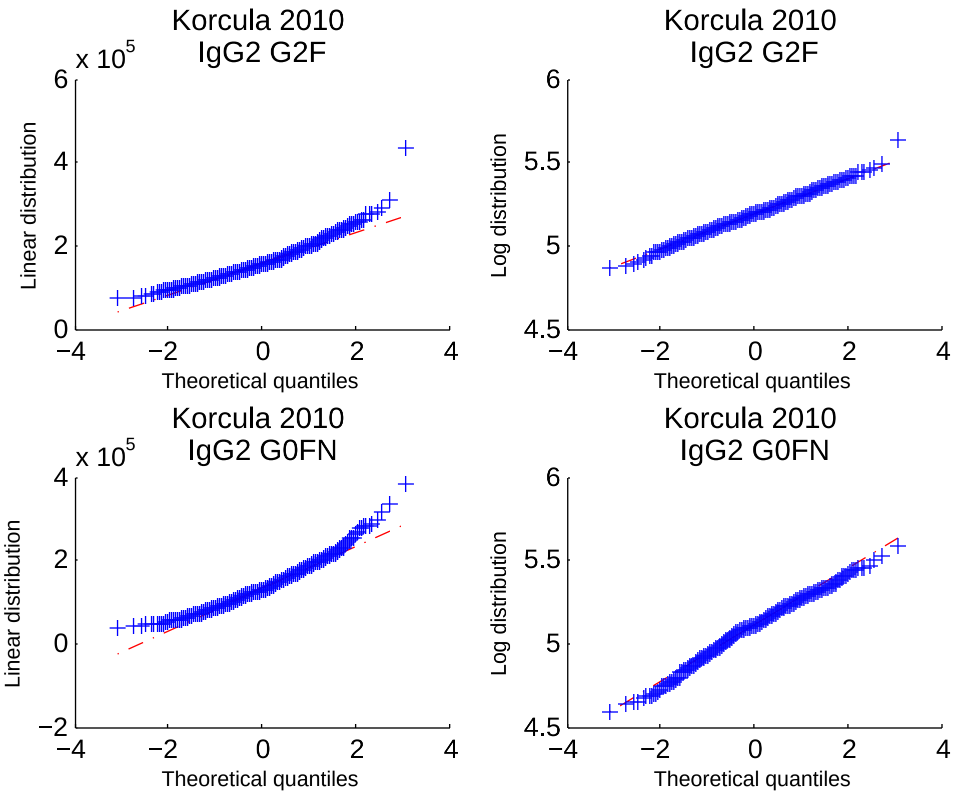

Korcula 2010
IgG2 G2F
x 10^5
Linear distribution
Theoretical quantiles
Korcula 2010
IgG2 G2F
Log distribution
Theoretical quantiles
Korcula 2010
IgG2 G0FN
x 10^5
Linear distribution
Theoretical quantiles
Korcula 2010
IgG2 G0FN
Log distribution
Theoretical quantiles

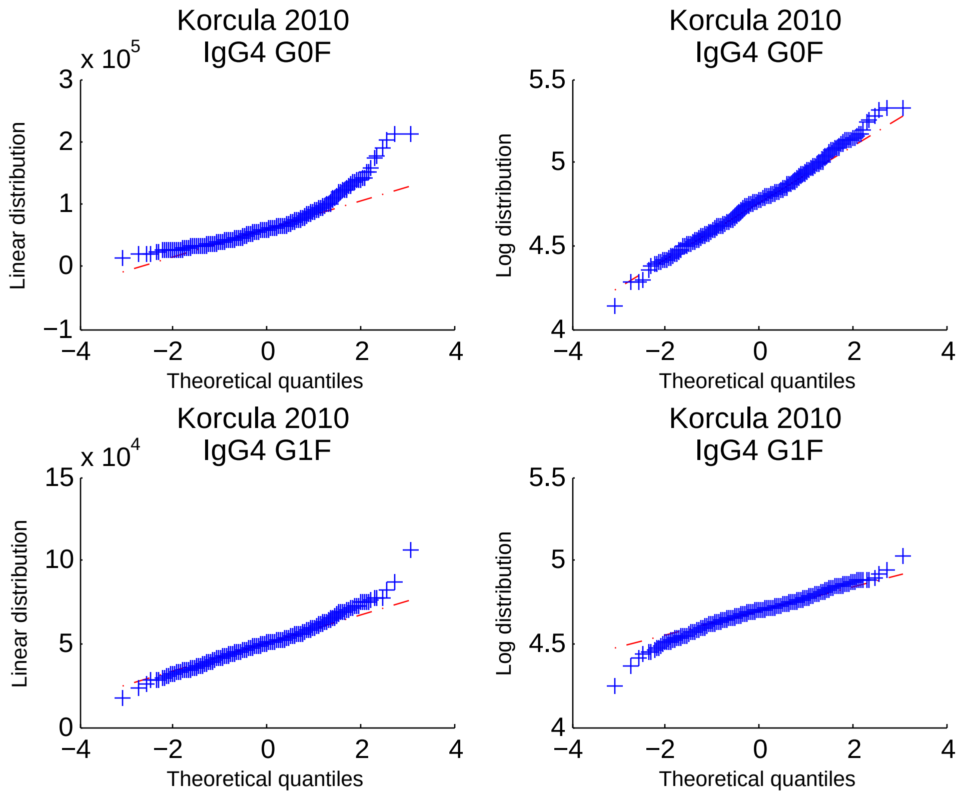
Korcula 2010
IgG4 G0F
x 10^5
Linear distribution
Theoretical quantiles
Korcula 2010
IgG4 G0F
Log distribution
Theoretical quantiles
Korcula 2010
IgG4 G1F
x 10^4
Linear distribution
Theoretical quantiles
Korcula 2010
IgG4 G1F
Log distribution
Theoretical quantiles

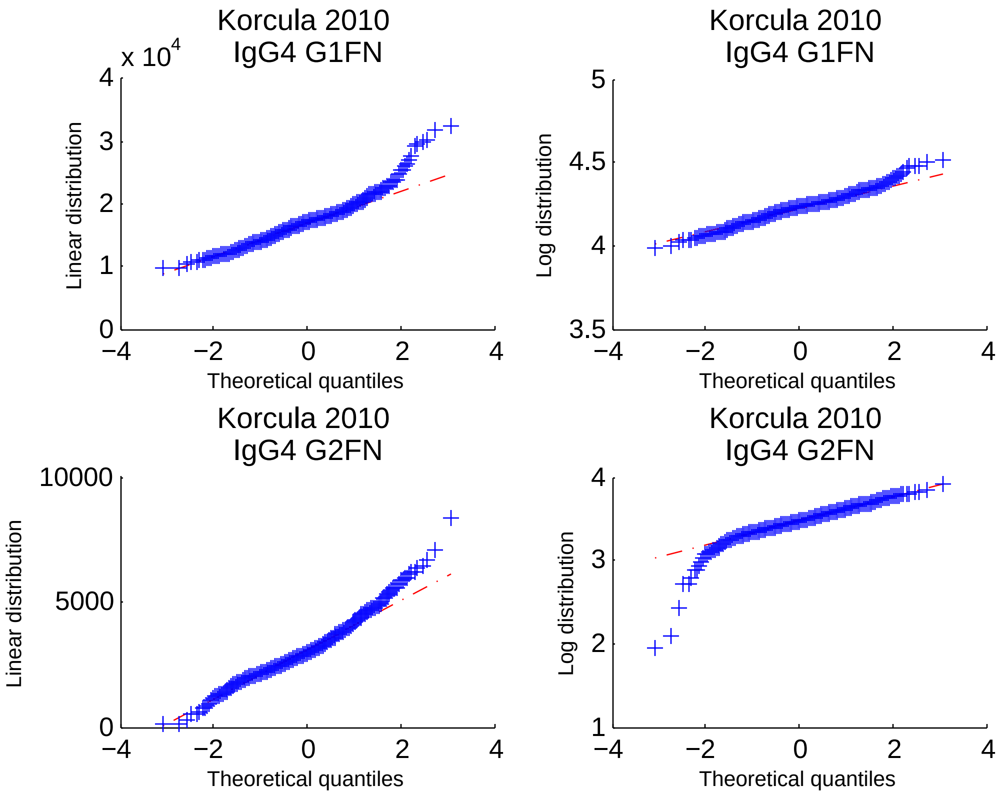

Korcula 2010
IgG4 G1FN
x 10^4
Linear distribution
Theoretical quantiles
Korcula 2010
IgG4 G1FN
Log distribution
Theoretical quantiles
Korcula 2010
IgG4 G2FN
Linear distribution
Theoretical quantiles
Korcula 2010
IgG4 G2FN
Log distribution
Theoretical quantiles

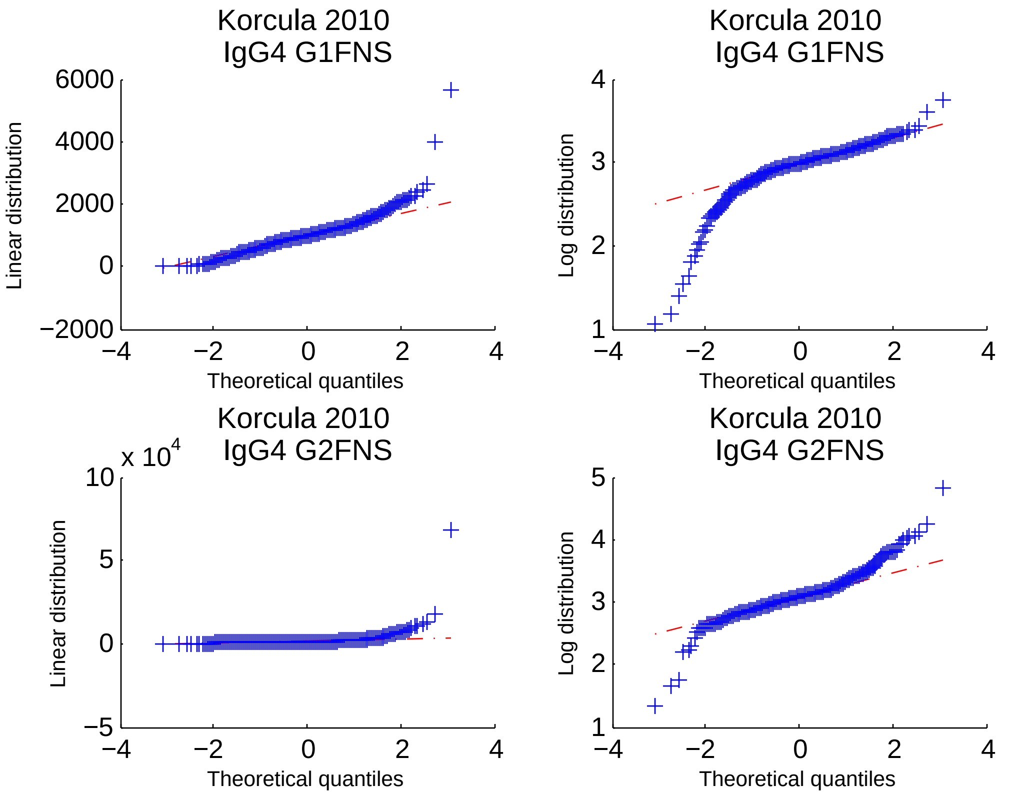
Korcula 2010
IgG4 G1FNS
Linear distribution
6000
4000
2000
0
−2000
−4
−2
0
2
4
Theoretical quantiles
Korcula 2010
IgG4 G1FNS
Log distribution
4
3
2
1
−4
−2
0
2
4
Theoretical quantiles
Korcula 2010
IgG4 G2FNS
x 10^4
Linear distribution
10
5
0
−5
−4
−2
0
2
4
Theoretical quantiles
Korcula 2010
IgG4 G2FNS
Log distribution
5
4
3
2
1
−4
−2
0
2
4
Theoretical quantiles

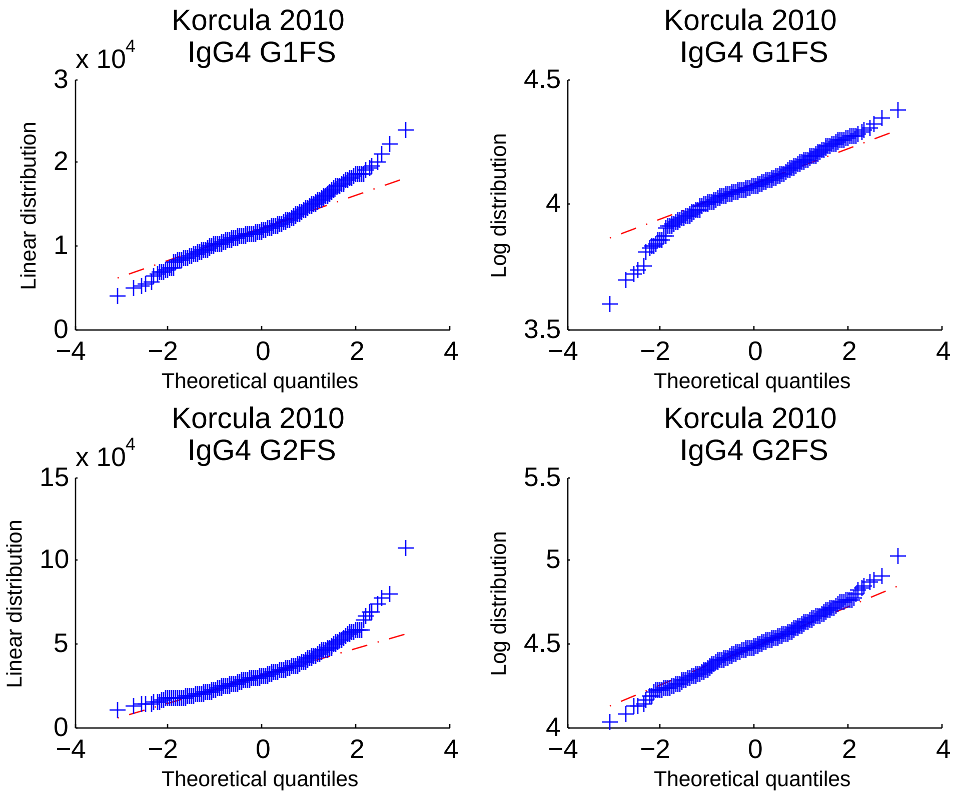
Korcula 2010
IgG4 G1FS
x 10^4
Linear distribution
Theoretical quantiles
Korcula 2010
IgG4 G1FS
Log distribution
Theoretical quantiles
Korcula 2010
IgG4 G2FS
x 10^4
Linear distribution
Theoretical quantiles
Korcula 2010
IgG4 G2FS
Log distribution
Theoretical quantiles

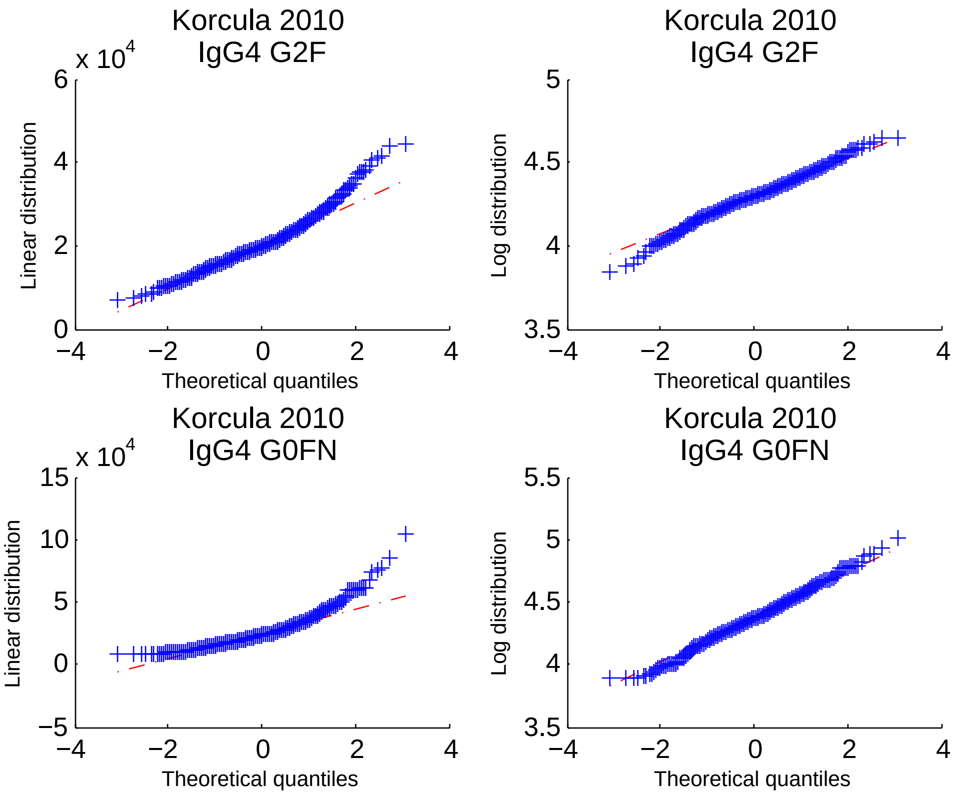

Korcula 2010
IgG4 G2F
x 10^4
Linear distribution
Theoretical quantiles
Korcula 2010
IgG4 G2F
Log distribution
Theoretical quantiles
Korcula 2010
IgG4 G0FN
x 10^4
Linear distribution
Theoretical quantiles
Korcula 2010
IgG4 G0FN
Log distribution
Theoretical quantiles

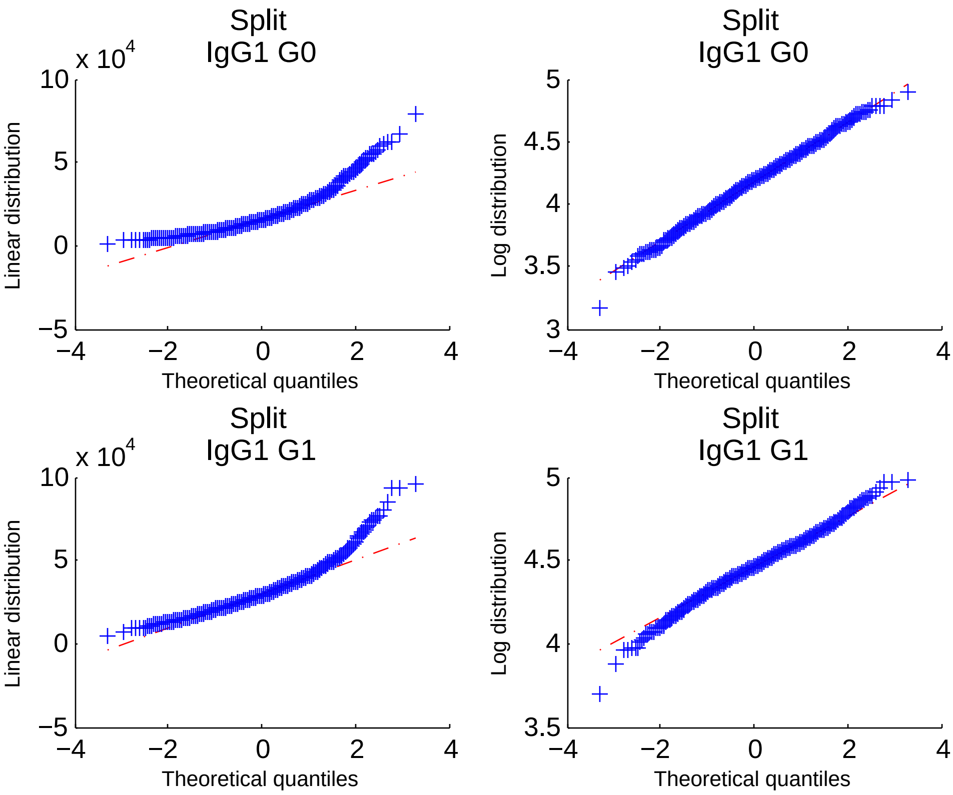

Split
IgG1 G0
x 10^4
Linear distribution
Theoretical quantiles
Split
IgG1 G0
Log distribution
Theoretical quantiles
Split
IgG1 G1
x 10^4
Linear distribution
Theoretical quantiles
Split
IgG1 G1
Log distribution
Theoretical quantiles

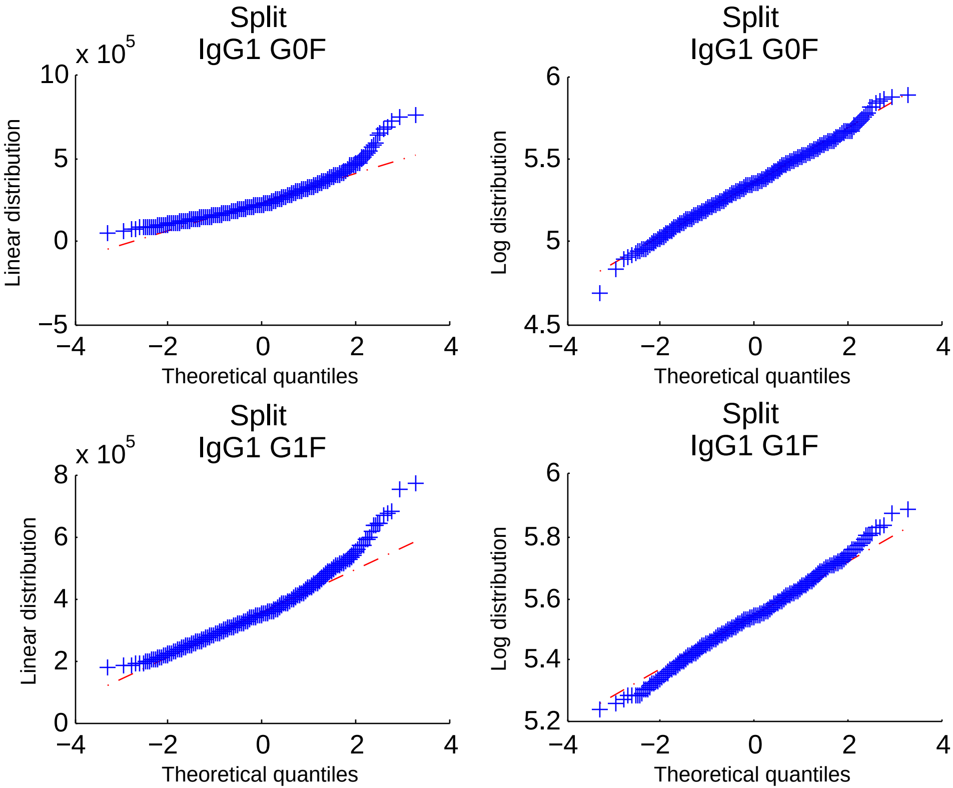
Split
IgG1 G0F
x 10^5
10
5
0
−5
Linear distribution
−4
−2
0
2
4
Theoretical quantiles
Split
IgG1 G0F
6
5.5
5
4.5
Log distribution
−4
−2
0
2
4
Theoretical quantiles
Split
IgG1 G1F
x 10^5
8
6
4
2
0
Linear distribution
−4
−2
0
2
4
Theoretical quantiles
Split
IgG1 G1F
6
5.8
5.6
5.4
5.2
Log distribution
−4
−2
0
2
4
Theoretical quantiles

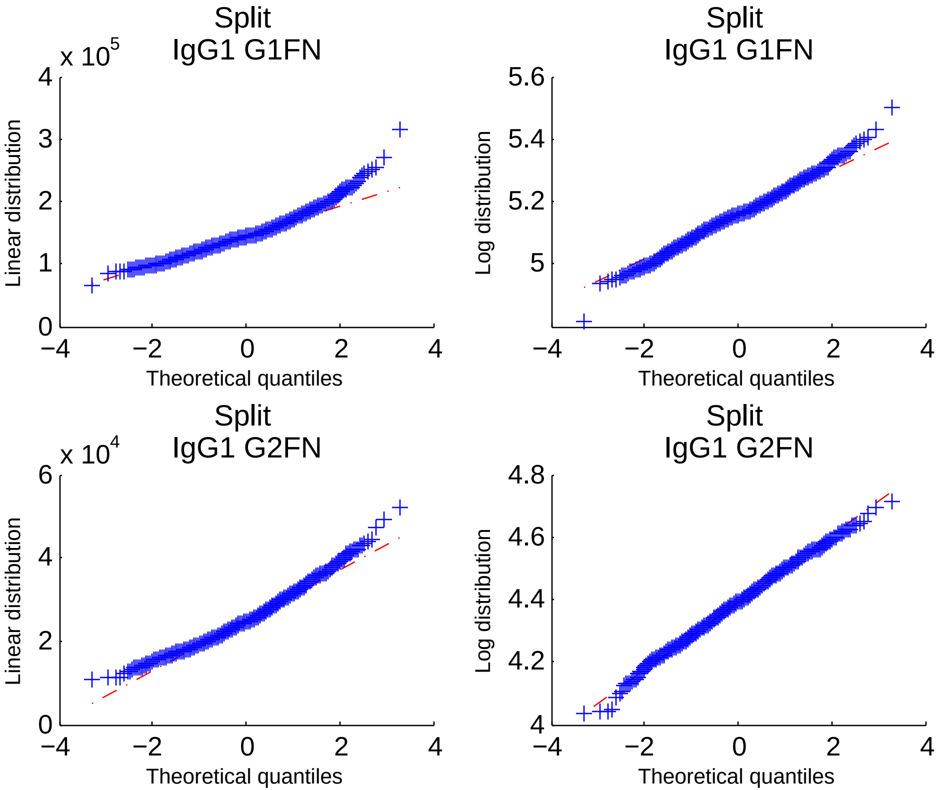

Split
IgG1 G1FN
x 10^5
Linear distribution
Theoretical quantiles
Split
IgG1 G1FN
Log distribution
Theoretical quantiles
Split
IgG1 G2FN
x 10^4
Linear distribution
Theoretical quantiles
Split
IgG1 G2FN
Log distribution
Theoretical quantiles

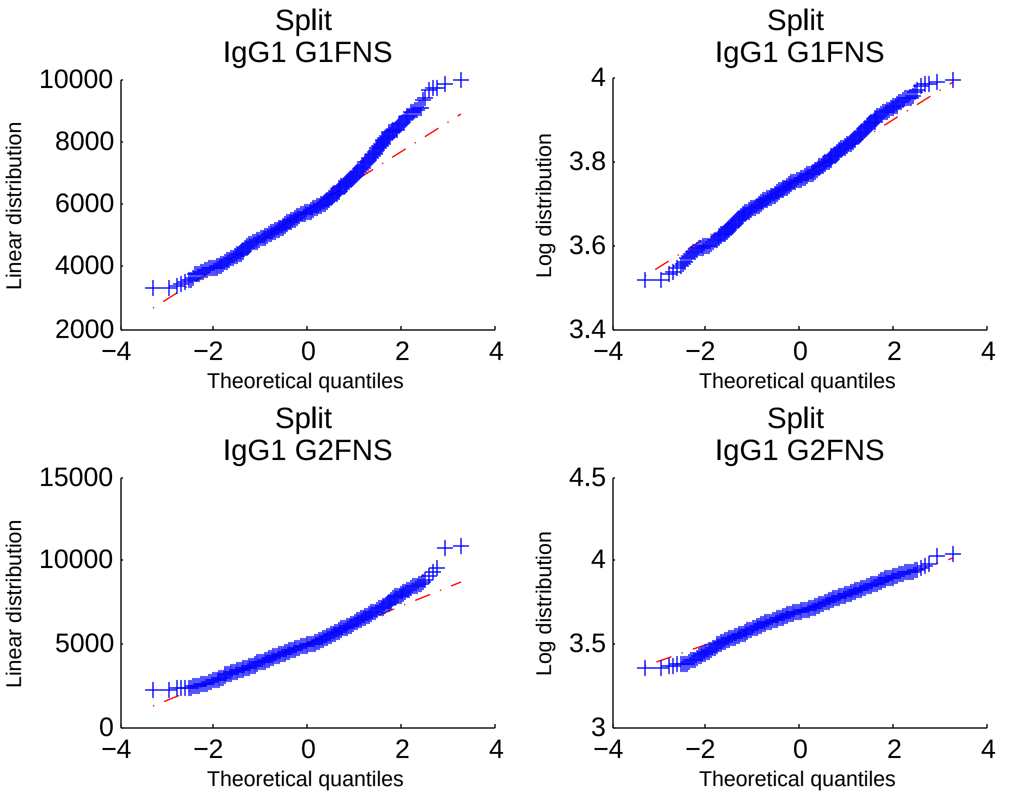
Split
IgG1 G1FNS
Linear distribution
10000
8000
6000
4000
2000
−4
−2
0
2
4
Theoretical quantiles
Split
IgG1 G1FNS
Log distribution
4
3.8
3.6
3.4
−4
−2
0
2
4
Theoretical quantiles
Split
IgG1 G2FNS
Linear distribution
15000
10000
5000
0
−4
−2
0
2
4
Theoretical quantiles
Split
IgG1 G2FNS
Log distribution
4.5
4
3.5
3
−4
−2
0
2
4
Theoretical quantiles

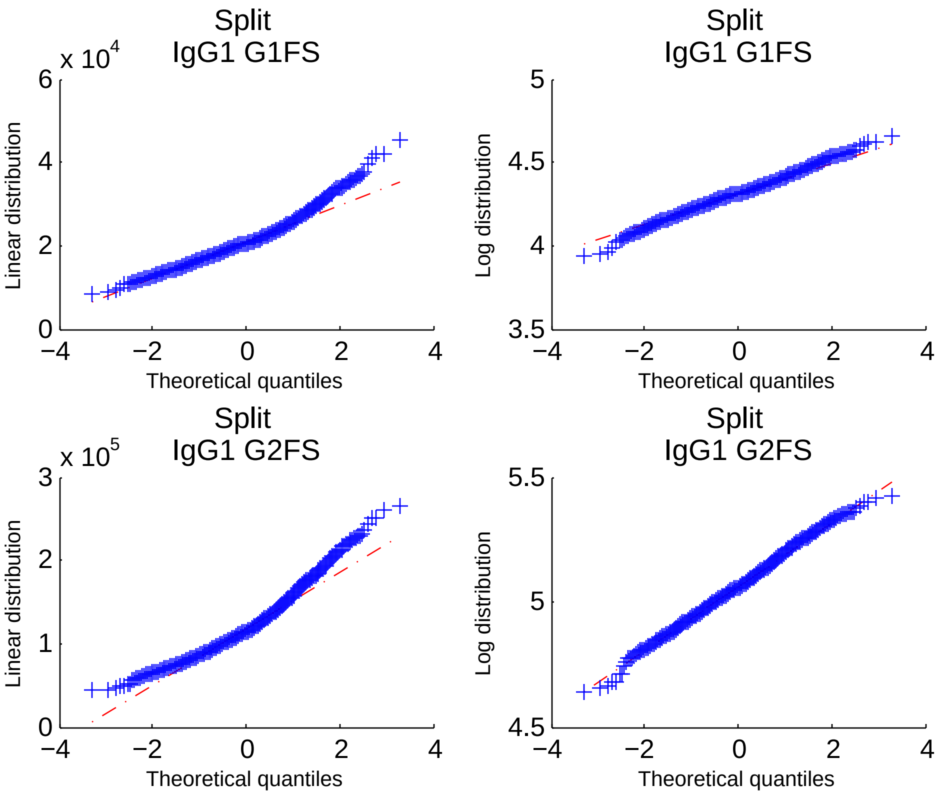
Split
IgG1 G1FS
x 10^4
6
4
2
0
Linear distribution
−4
−2
0
2
4
Theoretical quantiles
Split
IgG1 G1FS
5
4.5
4
3.5
Log distribution
−4
−2
0
2
4
Theoretical quantiles
Split
IgG1 G2FS
x 10^5
3
2
1
0
Linear distribution
−4
−2
0
2
4
Theoretical quantiles
Split
IgG1 G2FS
5.5
5
4.5
Log distribution
−4
−2
0
2
4
Theoretical quantiles

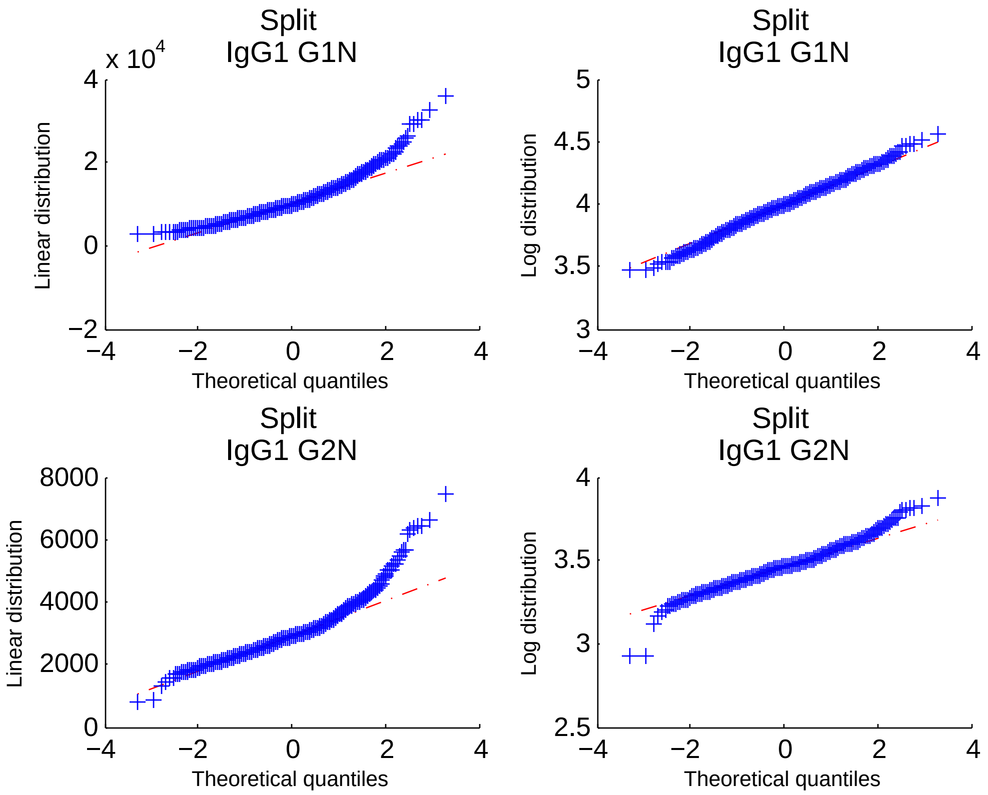
Split
IgG1 G1N
x 10^4
Linear distribution
Theoretical quantiles
Split
IgG1 G1N
Log distribution
Theoretical quantiles
Split
IgG1 G2N
Linear distribution
Theoretical quantiles
Split
IgG1 G2N
Log distribution
Theoretical quantiles

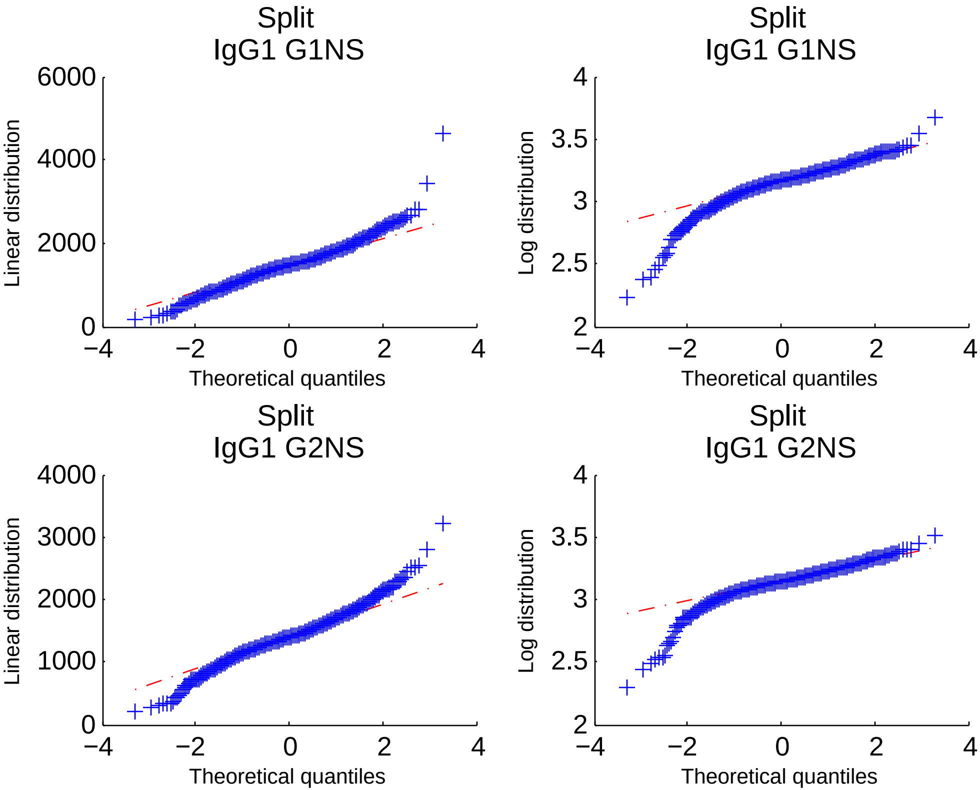
Split
IgG1 G1NS
Linear distribution
Log distribution
Theoretical quantiles
Split
IgG1 G2NS
Linear distribution
Log distribution
Theoretical quantiles
6000
4000
2000
3000
1000
0
4
3.5
3
2.5
2
−4
−2
0
2
4

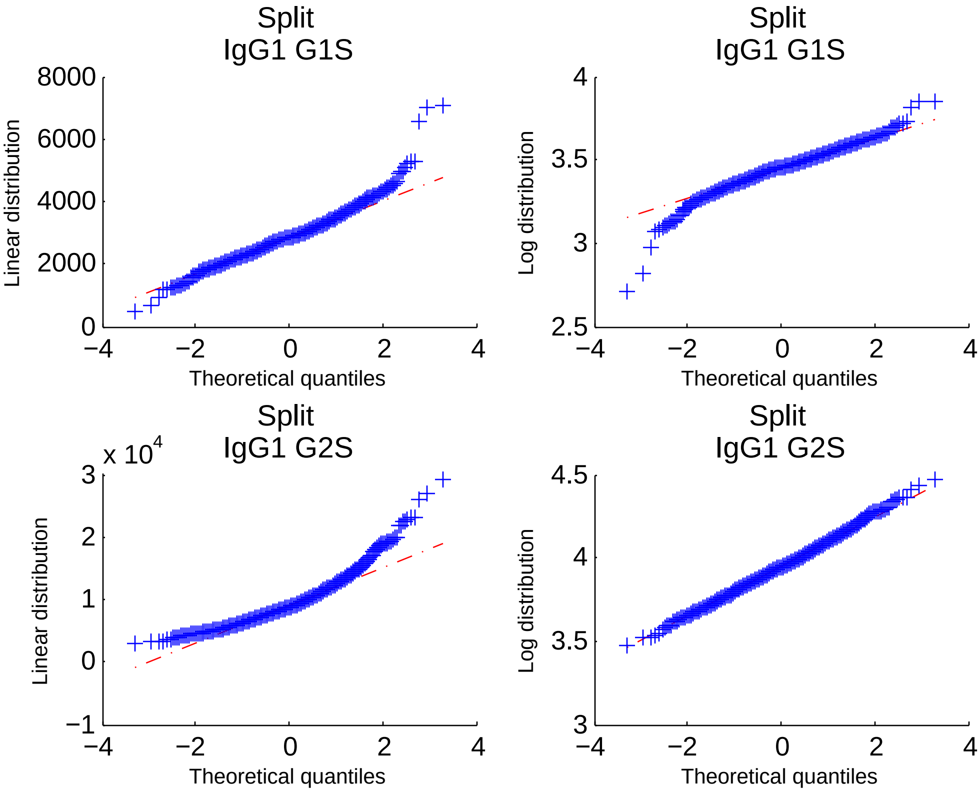
Split
IgG1 G1S
Linear distribution
Theoretical quantiles
8000
6000
4000
2000
0
−4
−2
0
2
4
Split
IgG1 G1S
Log distribution
Theoretical quantiles
4
3.5
3
2.5
Split
IgG1 G2S
x 10^4
Linear distribution
Theoretical quantiles
3
2
1
0
−1
Split
IgG1 G2S
Log distribution
Theoretical quantiles
4.5
4
3.5
3

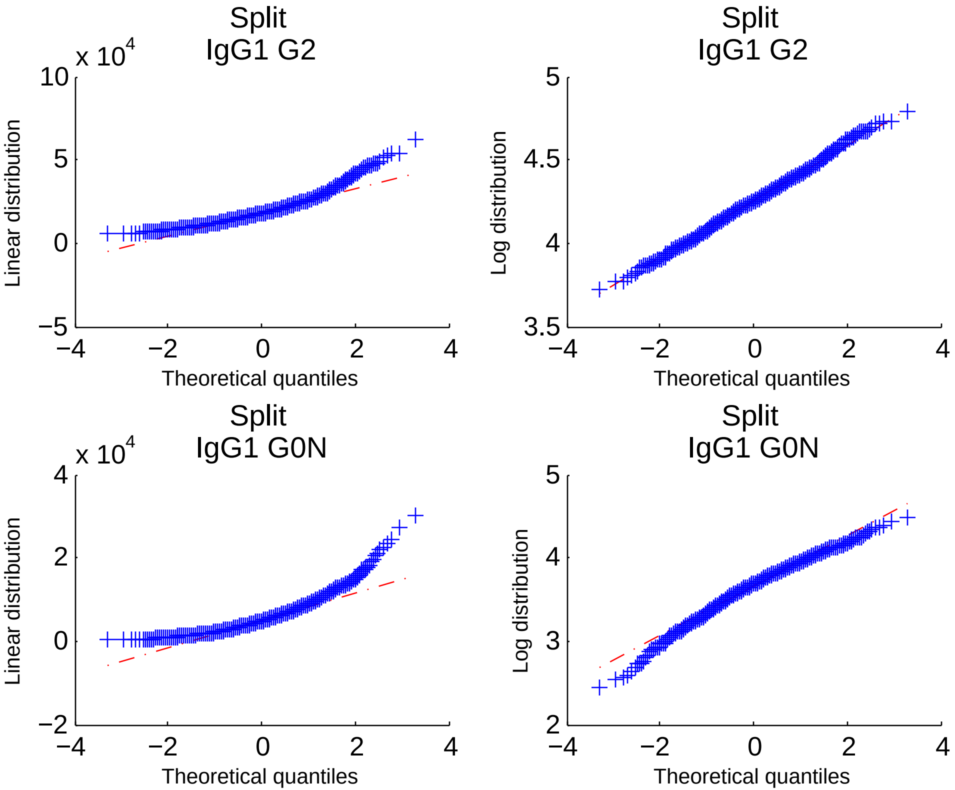
Split
IgG1 G2
x 10^4
Linear distribution
Theoretical quantiles
Split
IgG1 G2
Log distribution
Theoretical quantiles
Split
IgG1 G0N
x 10^4
Linear distribution
Theoretical quantiles
Split
IgG1 G0N
Log distribution
Theoretical quantiles

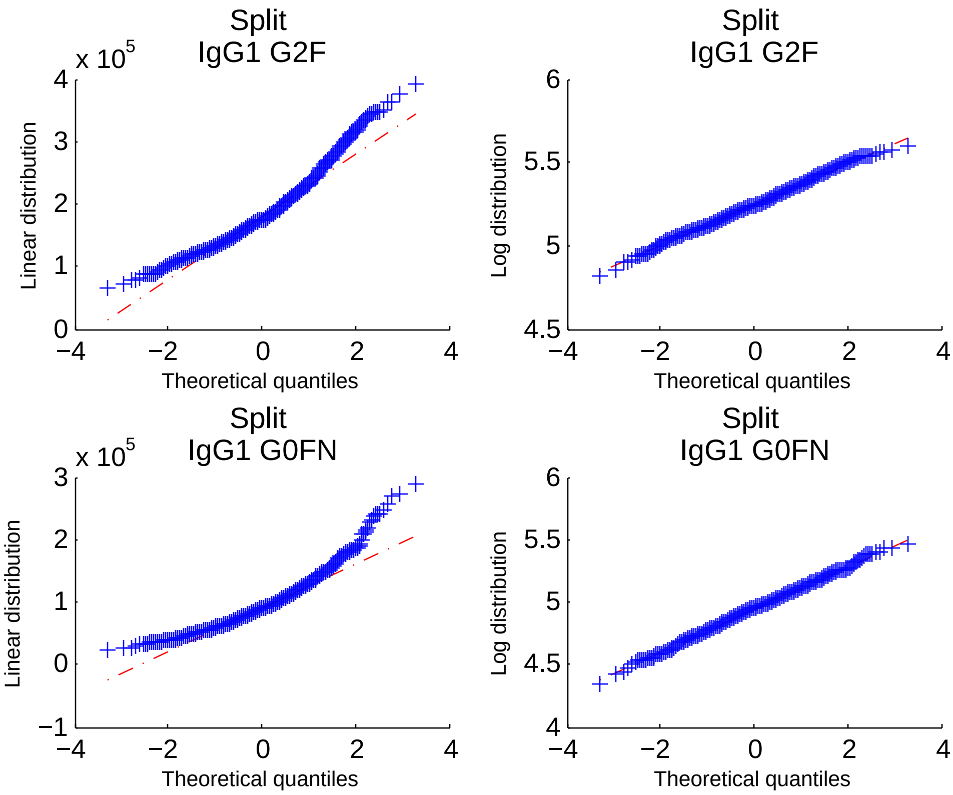

Split
IgG1 G2F
x 10^5
Linear distribution
Theoretical quantiles
Split
IgG1 G2F
Log distribution
Theoretical quantiles
Split
IgG1 G0FN
x 10^5
Linear distribution
Theoretical quantiles
Split
IgG1 G0FN
Log distribution
Theoretical quantiles

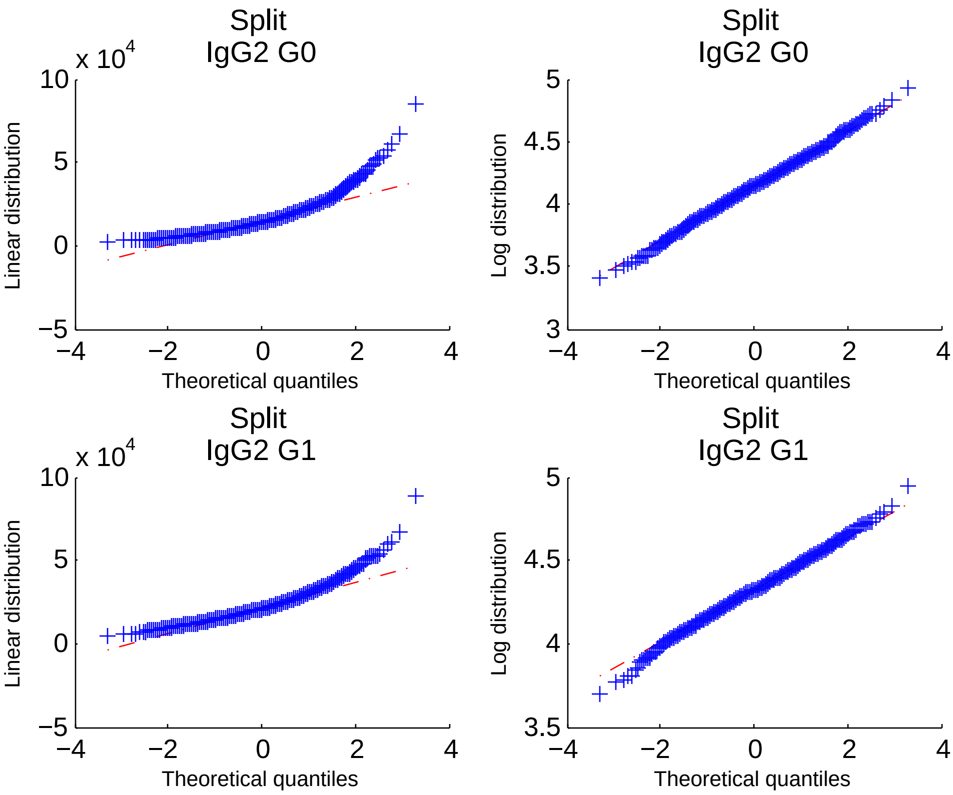
Split
IgG2 G0
x 10^4
Linear distribution
Theoretical quantiles
Split
IgG2 G0
Log distribution
Theoretical quantiles
Split
IgG2 G1
x 10^4
Linear distribution
Theoretical quantiles
Split
IgG2 G1
Log distribution
Theoretical quantiles

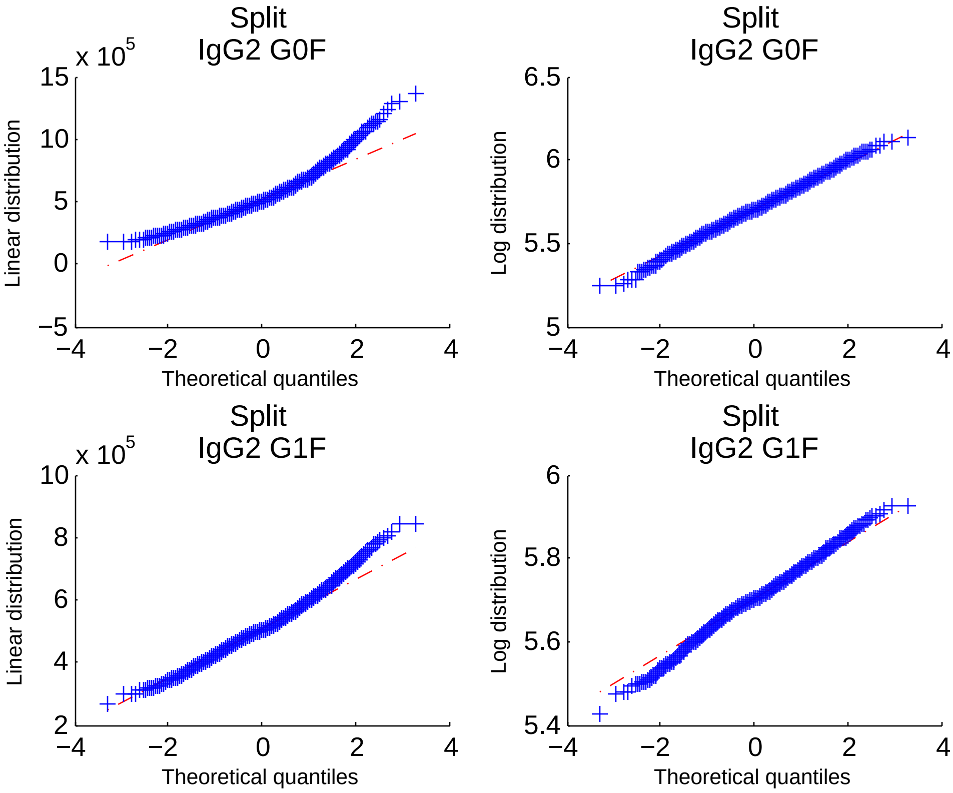
Split
IgG2 G0F
x 10^5
15
10
5
0
−5
Linear distribution
−4
−2
0
2
4
Theoretical quantiles
Split
IgG2 G0F
6.5
6
5.5
5
Log distribution
Theoretical quantiles
Split
IgG2 G1F
x 10^5
10
8
6
4
2
Linear distribution
Theoretical quantiles
Split
IgG2 G1F
6
5.8
5.6
5.4
Log distribution
Theoretical quantiles

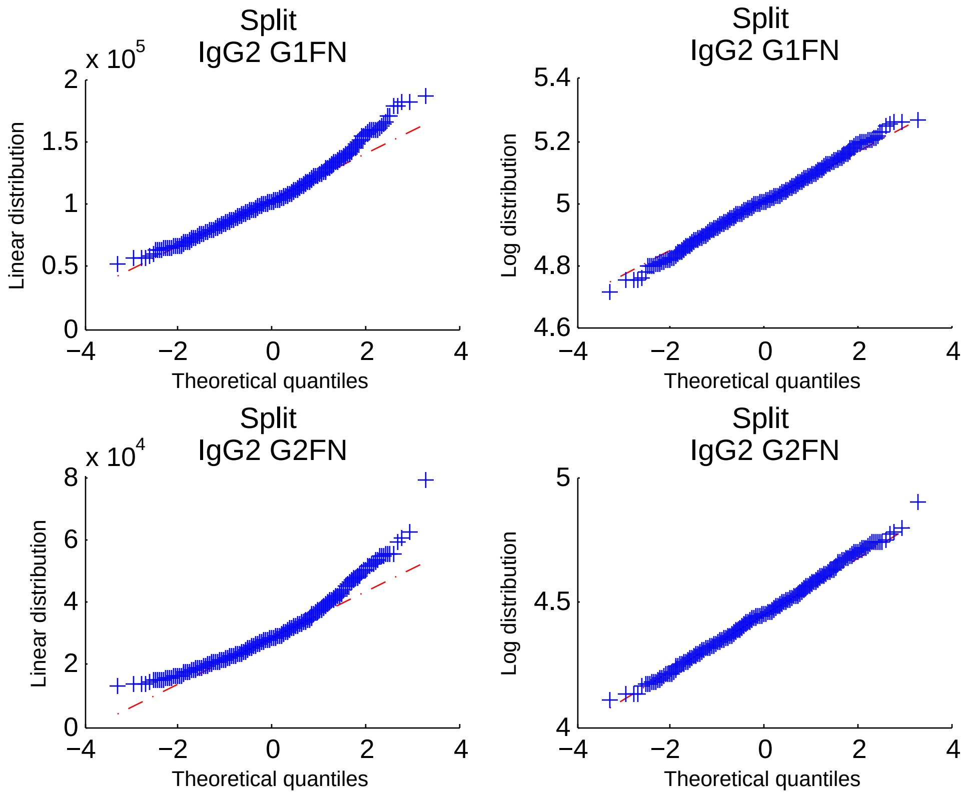

Split
IgG2 G1FN
x 10^5
2
1.5
1
0.5
0
Linear distribution
−4
−2
0
2
4
Theoretical quantiles
Split
IgG2 G1FN
5.4
5.2
5
4.8
4.6
Log distribution
−4
−2
0
2
4
Theoretical quantiles
Split
IgG2 G2FN
x 10^4
8
6
4
2
0
Linear distribution
−4
−2
0
2
4
Theoretical quantiles
Split
IgG2 G2FN
5
4.5
4
Log distribution
−4
−2
0
2
4
Theoretical quantiles

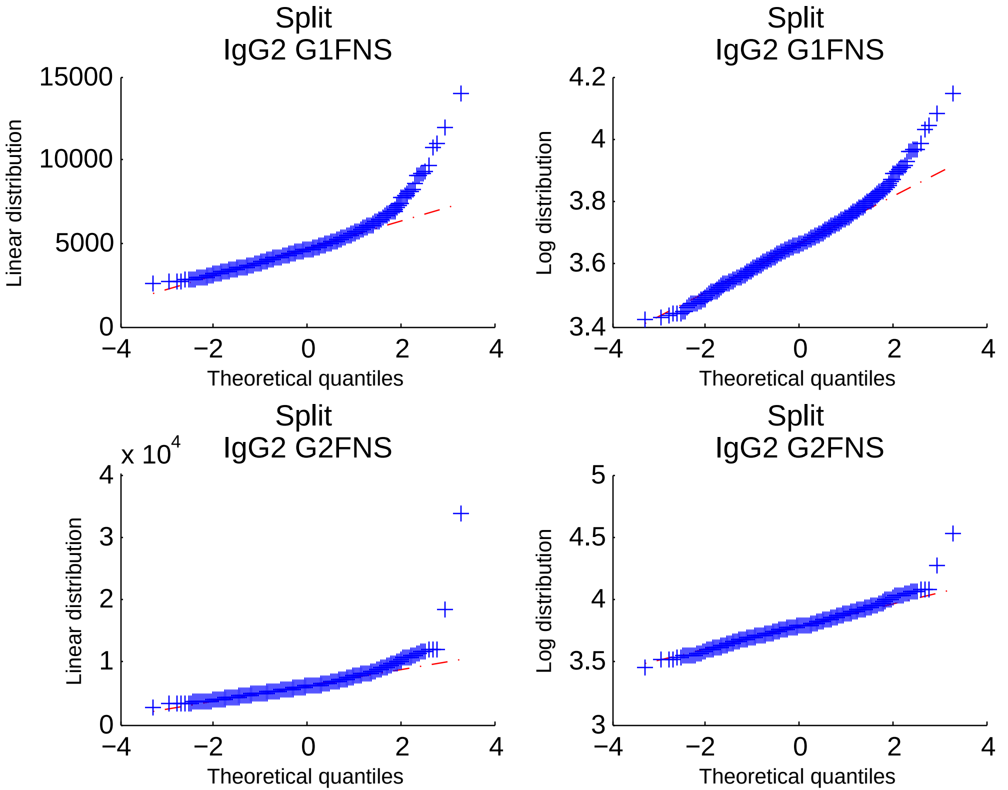

Split
IgG2 G1FNS
Linear distribution
Theoretical quantiles
Split
IgG2 G1FNS
Log distribution
Theoretical quantiles
Split
IgG2 G2FNS
x 10^4
Linear distribution
Theoretical quantiles
Split
IgG2 G2FNS
Log distribution
Theoretical quantiles

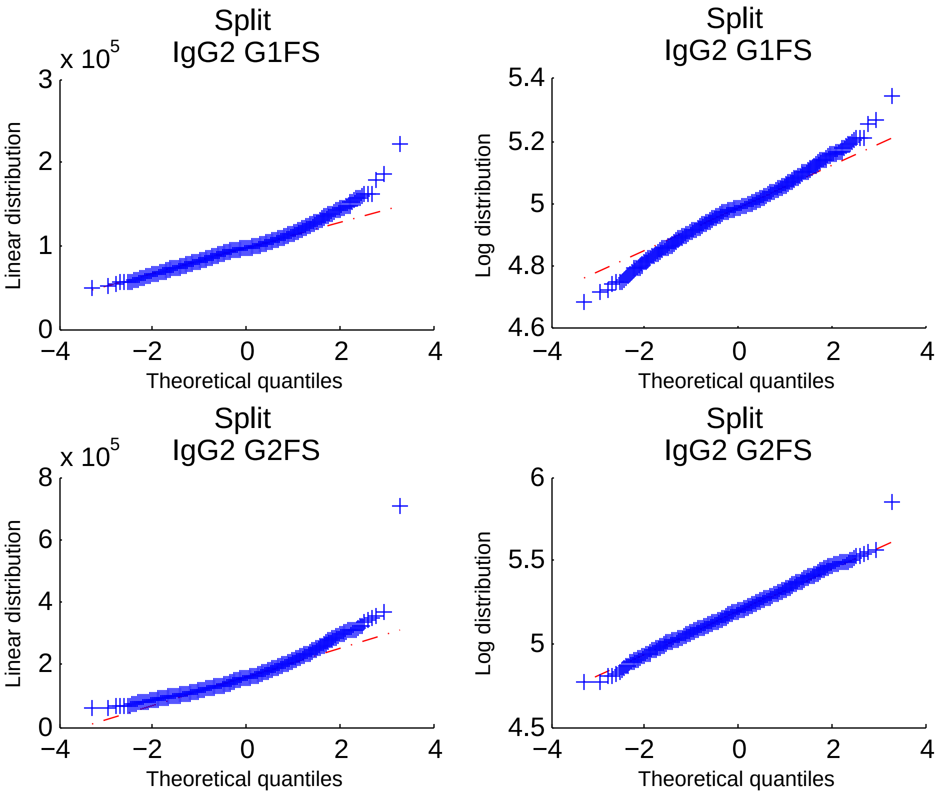

Split
IgG2 G1FS
x 10^5
Linear distribution
Theoretical quantiles
Split
IgG2 G1FS
Log distribution
Theoretical quantiles
Split
IgG2 G2FS
x 10^5
Linear distribution
Theoretical quantiles
Split
IgG2 G2FS
Log distribution
Theoretical quantiles

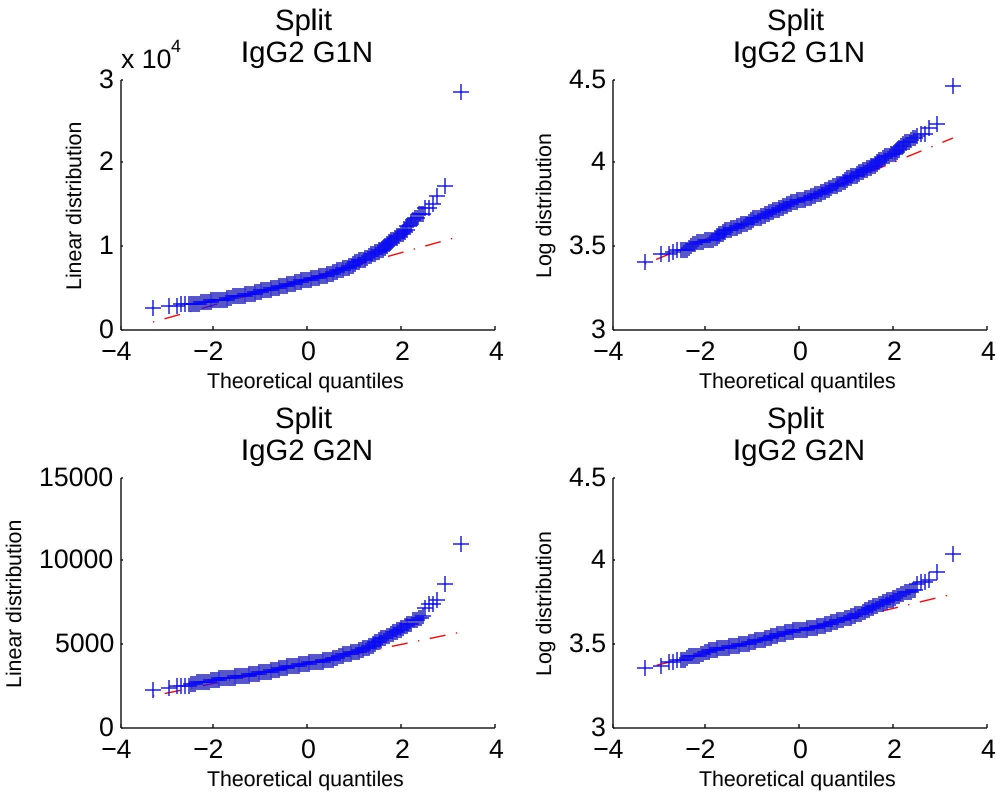
Split
IgG2 G1N
x 10^4
Linear distribution
Theoretical quantiles
Split
IgG2 G1N
Log distribution
Theoretical quantiles
Split
IgG2 G2N
Linear distribution
Theoretical quantiles
Split
IgG2 G2N
Log distribution
Theoretical quantiles

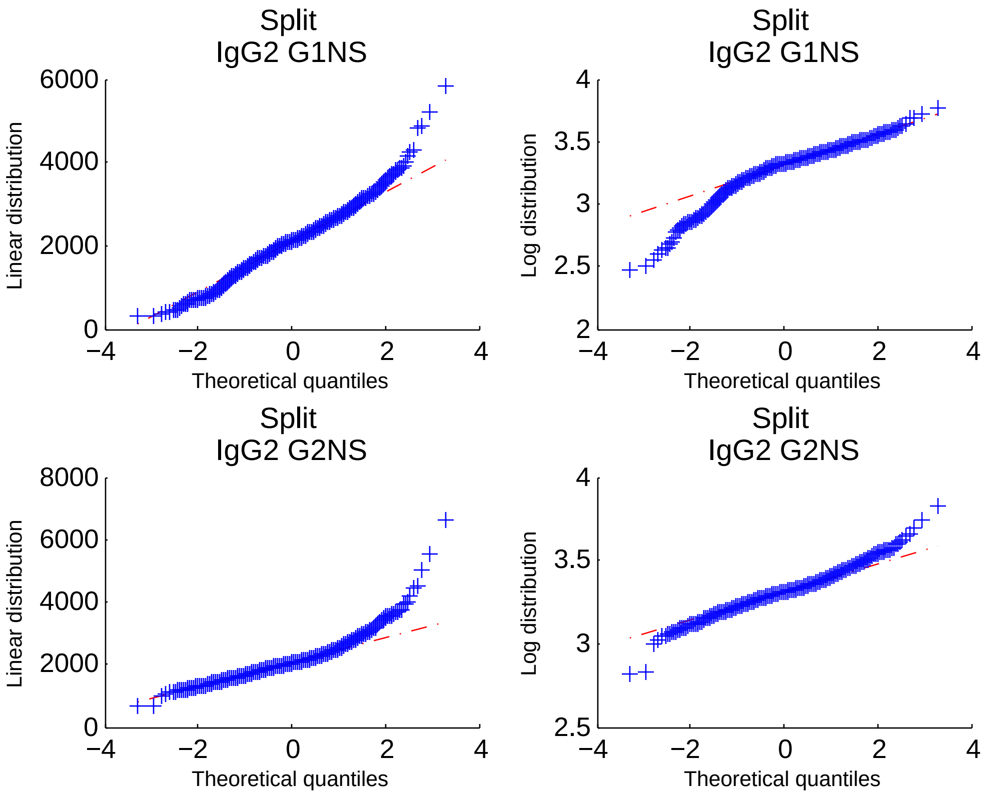
Split
IgG2 G1NS
Linear distribution
Theoretical quantiles
0
2000
4000
6000
−4
−2
0
2
4
Split
IgG2 G1NS
Log distribution
Theoretical quantiles
2
2.5
3
3.5
4
−4
−2
0
2
4
Split
IgG2 G2NS
Linear distribution
Theoretical quantiles
0
2000
4000
6000
8000
−4
−2
0
2
4
Split
IgG2 G2NS
Log distribution
Theoretical quantiles
2.5
3
3.5
4
−4
−2
0
2
4

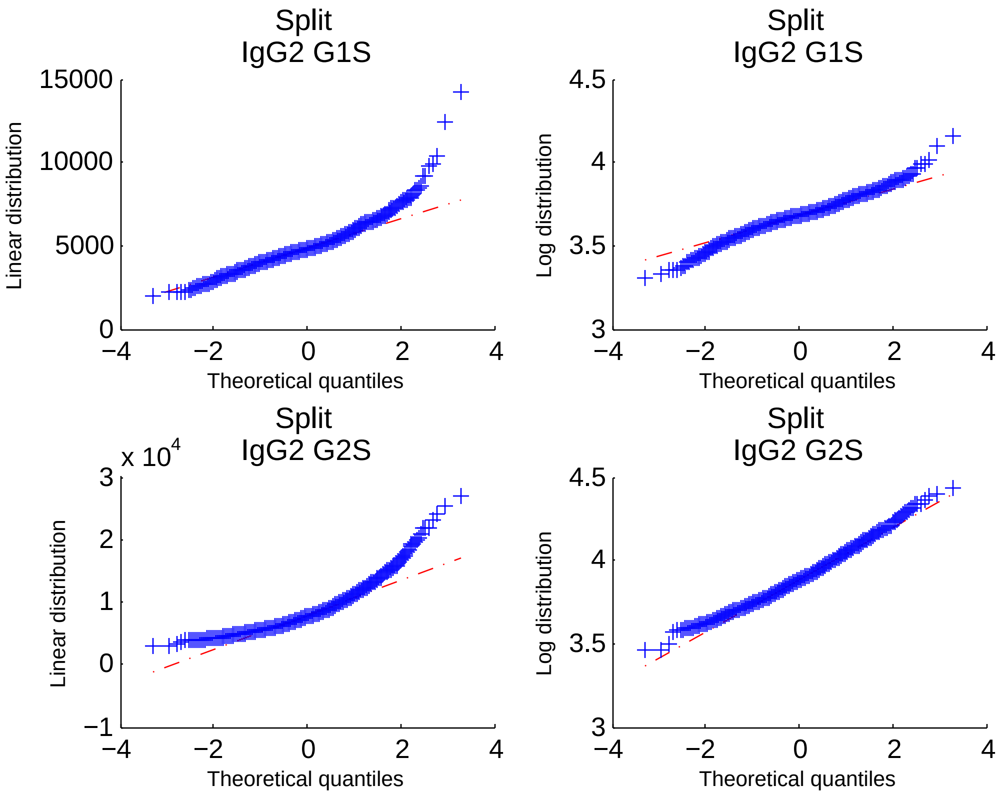
Split
IgG2 G1S
Linear distribution
Theoretical quantiles
Split
IgG2 G1S
Log distribution
Theoretical quantiles
Split
IgG2 G2S
x 10^4
Linear distribution
Theoretical quantiles
Split
IgG2 G2S
Log distribution
Theoretical quantiles

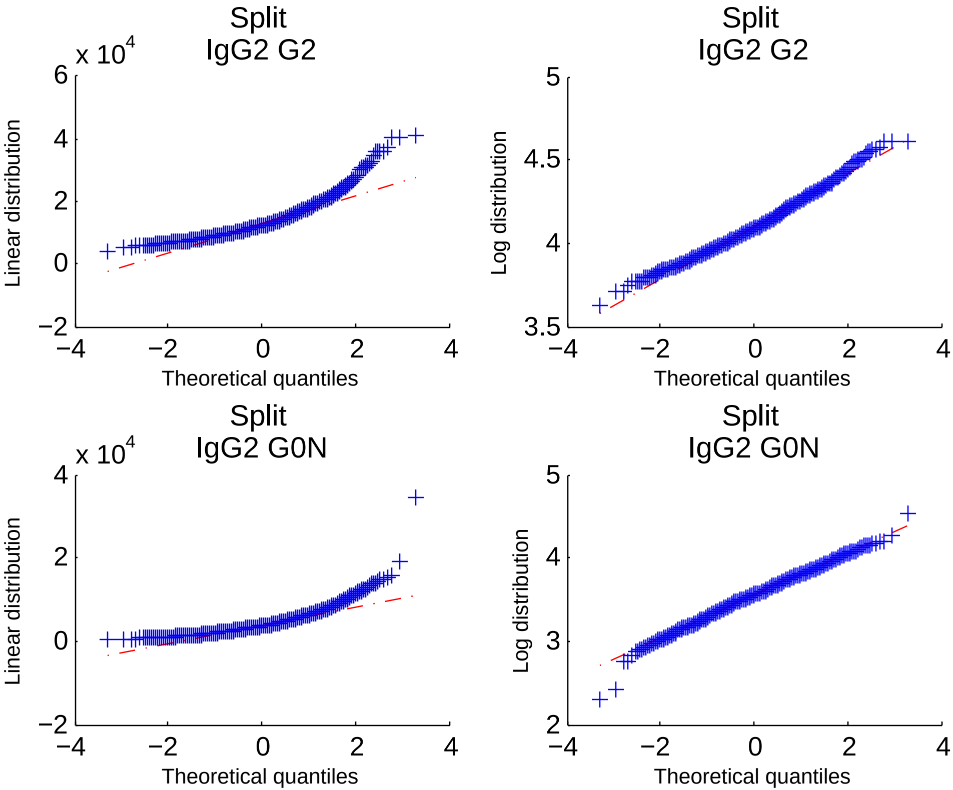
Split
IgG2 G2
x 10^4
Linear distribution
Theoretical quantiles
Split
IgG2 G2
Log distribution
Theoretical quantiles
Split
IgG2 G0N
x 10^4
Linear distribution
Theoretical quantiles
Split
IgG2 G0N
Log distribution
Theoretical quantiles

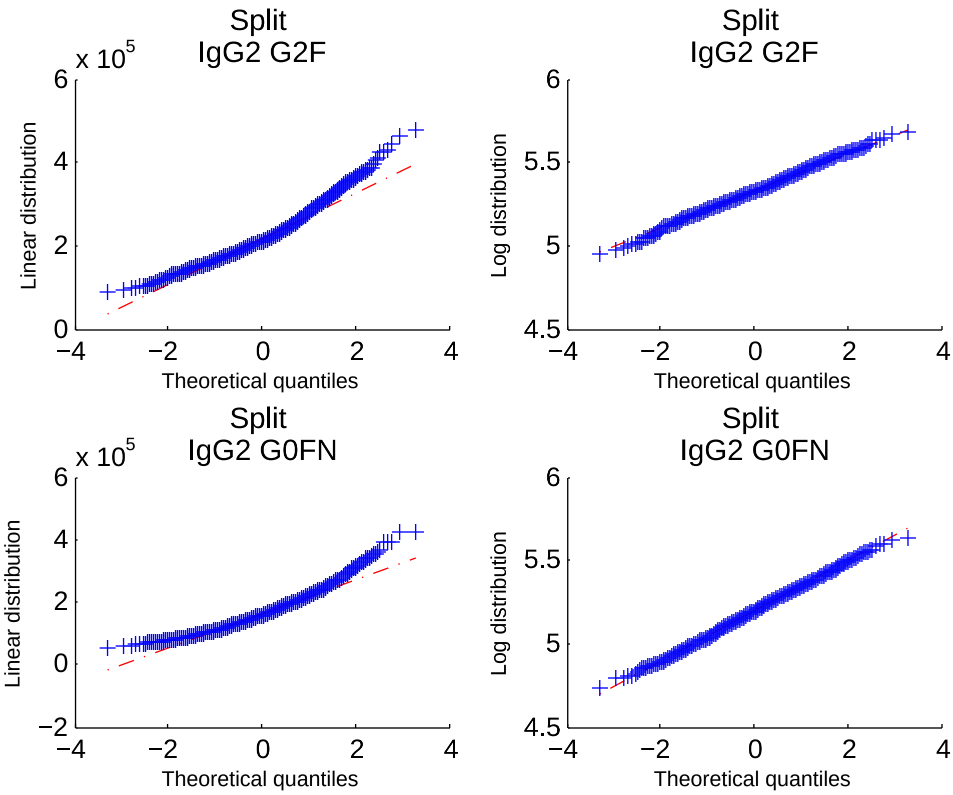
Split
IgG2 G2F
x 10^5
Linear distribution
Theoretical quantiles
Split
IgG2 G2F
Log distribution
Theoretical quantiles
Split
IgG2 G0FN
x 10^5
Linear distribution
Theoretical quantiles
Split
IgG2 G0FN
Log distribution
Theoretical quantiles

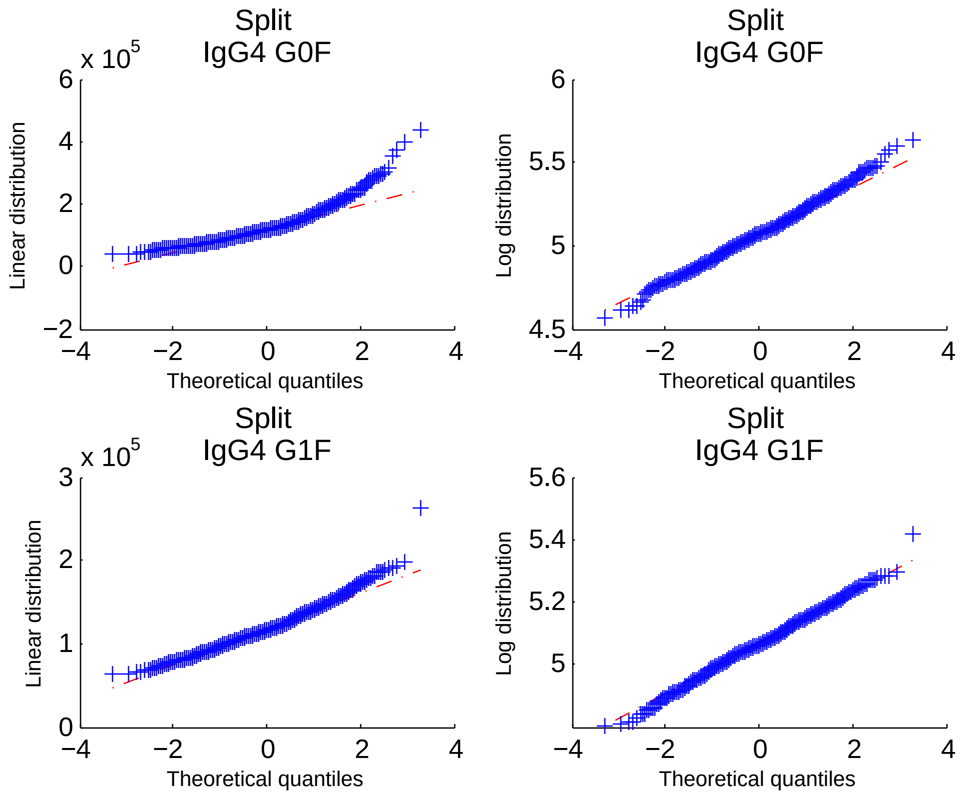

Split
IgG4 G0F
x 10^5
Linear distribution
Theoretical quantiles
Split
IgG4 G0F
Log distribution
Theoretical quantiles
Split
IgG4 G1F
x 10^5
Linear distribution
Theoretical quantiles
Split
IgG4 G1F
Log distribution
Theoretical quantiles

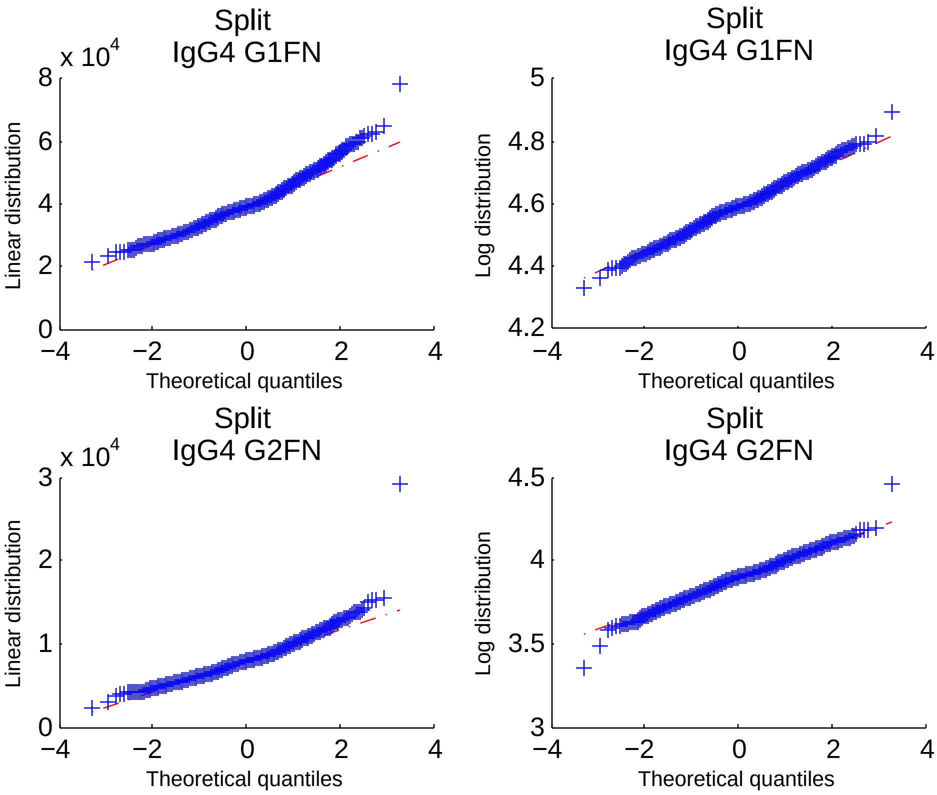
Split
IgG4 G1FN
x 10^4
Linear distribution
Theoretical quantiles
Split
IgG4 G1FN
Log distribution
Theoretical quantiles
Split
IgG4 G2FN
x 10^4
Linear distribution
Theoretical quantiles
Split
IgG4 G2FN
Log distribution
Theoretical quantiles

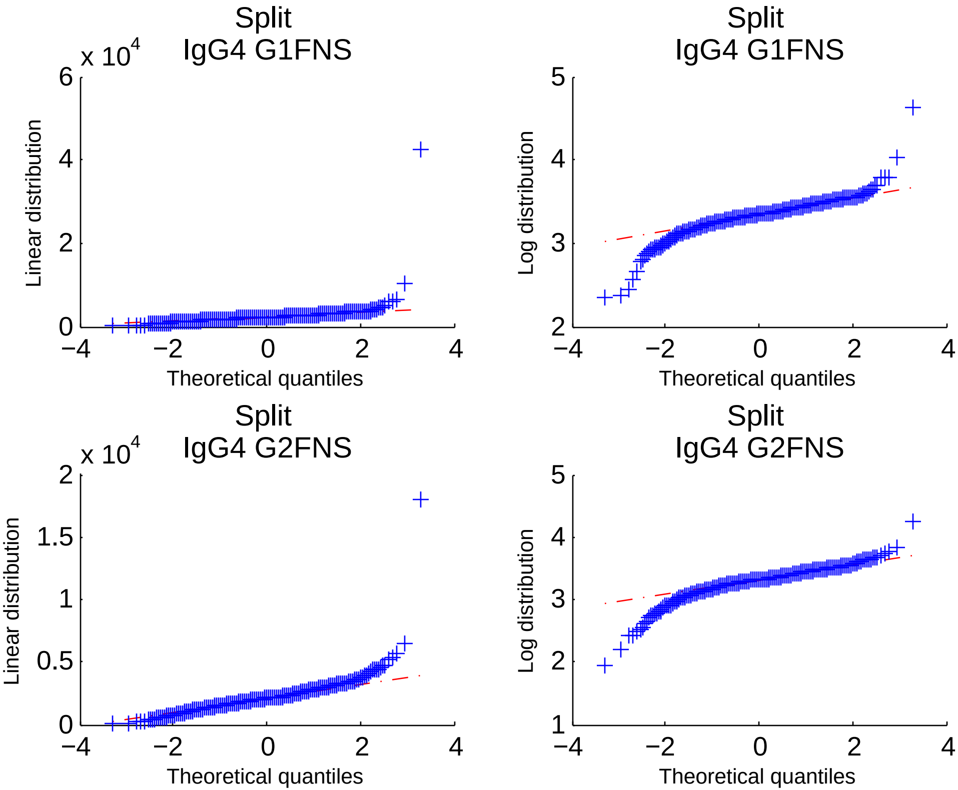
Split
IgG4 G1FNS
x 10^4
Linear distribution
Theoretical quantiles
Split
IgG4 G1FNS
Log distribution
Theoretical quantiles
Split
IgG4 G2FNS
x 10^4
Linear distribution
Theoretical quantiles
Split
IgG4 G2FNS
Log distribution
Theoretical quantiles

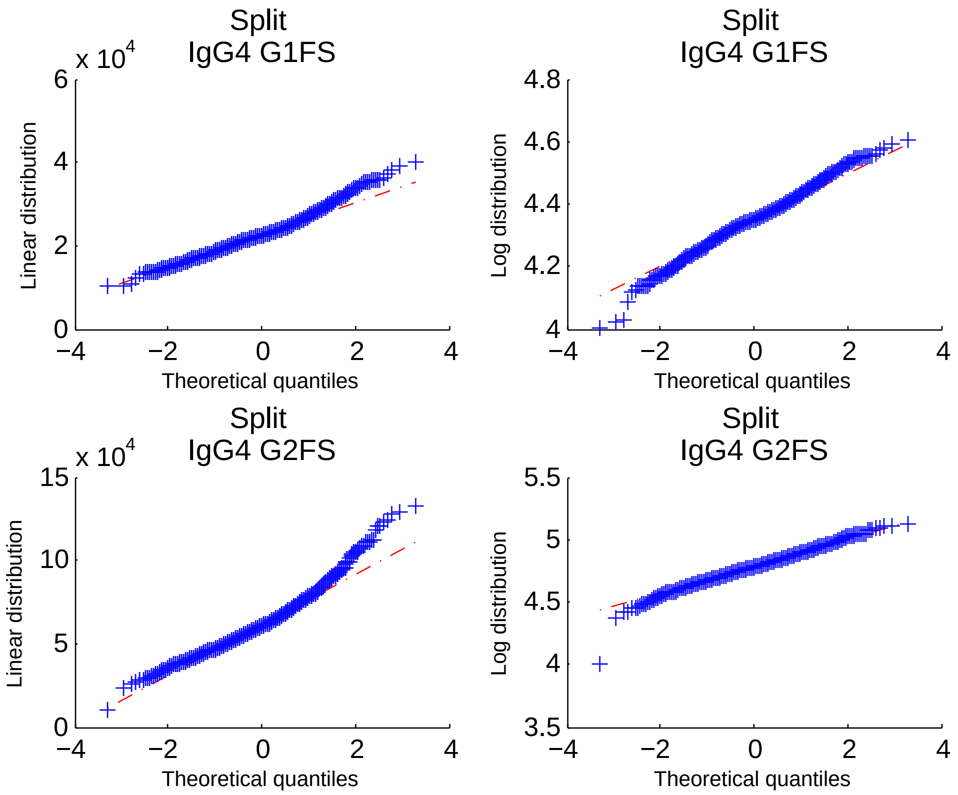

Split
IgG4 G1FS
x 10^4
Linear distribution
Theoretical quantiles
Split
IgG4 G1FS
Log distribution
Theoretical quantiles
Split
IgG4 G2FS
x 10^4
Linear distribution
Theoretical quantiles
Split
IgG4 G2FS
Log distribution
Theoretical quantiles

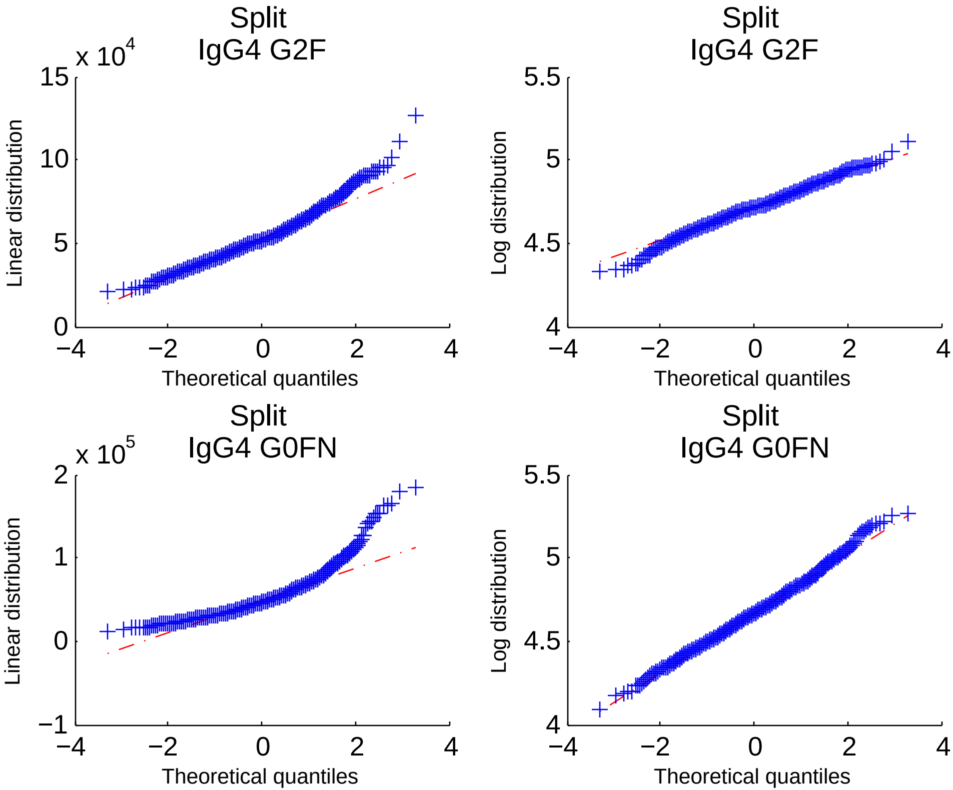

Split
IgG4 G2F
x 10^4
Linear distribution
Theoretical quantiles
Split
IgG4 G2F
Log distribution
Theoretical quantiles
Split
IgG4 G0FN
x 10^5
Linear distribution
Theoretical quantiles
Split
IgG4 G0FN
Log distribution
Theoretical quantiles

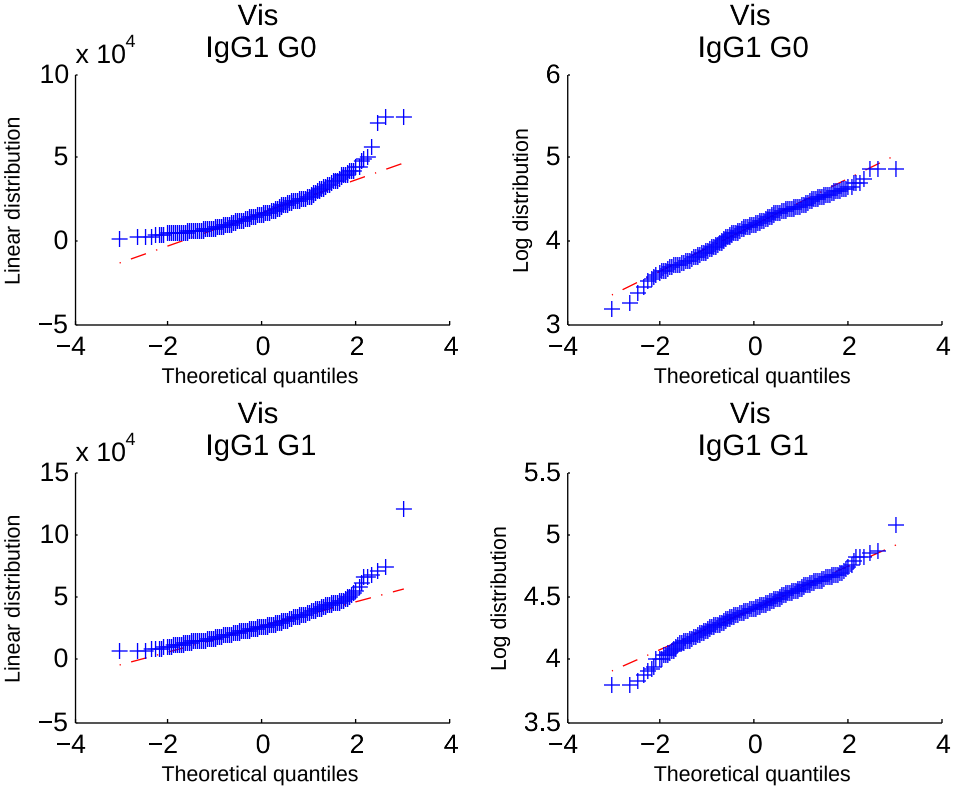

Vis
IgG1 G0
x 10^4
10
5
0
−5
−4
−2
0
2
4
Linear distribution
Theoretical quantiles
Vis
IgG1 G0
6
5
4
3
−4
−2
0
2
4
Log distribution
Theoretical quantiles
Vis
IgG1 G1
x 10^4
15
10
5
0
−5
−4
−2
0
2
4
Linear distribution
Theoretical quantiles
Vis
IgG1 G1
5.5
5
4.5
4
3.5
−4
−2
0
2
4
Log distribution
Theoretical quantiles

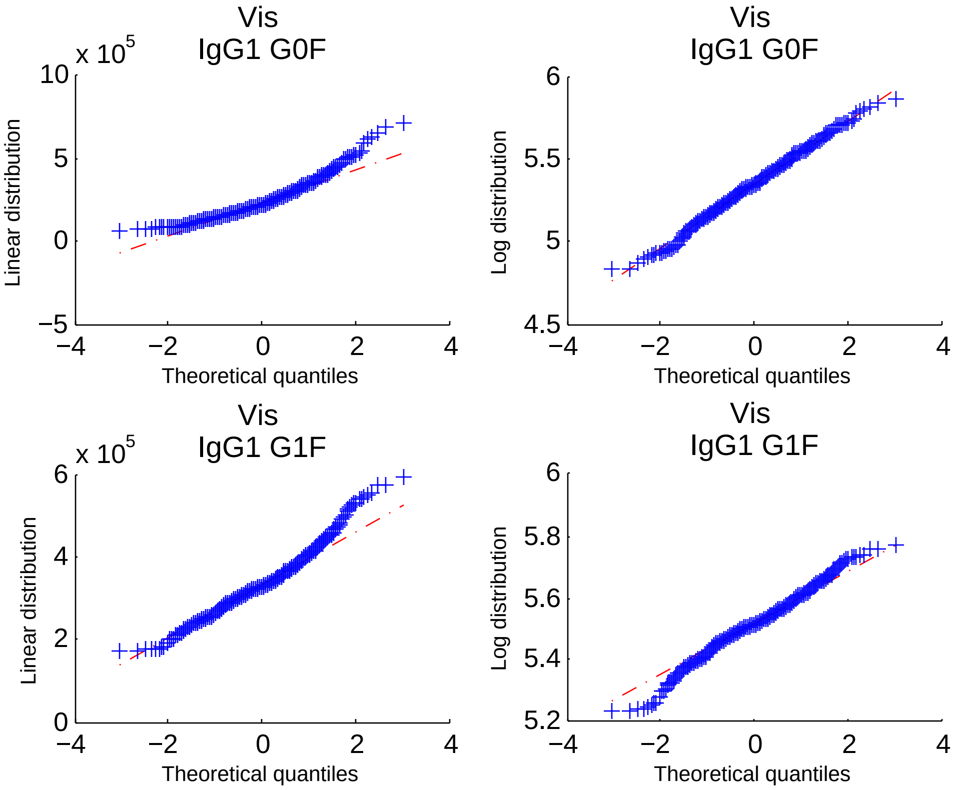

Vis
IgG1 G0F
x 10^5
10
5
0
−5
Linear distribution
−4
−2
0
2
4
Theoretical quantiles
Vis
IgG1 G0F
6
5.5
5
4.5
Log distribution
−4
−2
0
2
4
Theoretical quantiles
Vis
IgG1 G1F
x 10^5
6
4
2
0
Linear distribution
−4
−2
0
2
4
Theoretical quantiles
Vis
IgG1 G1F
6
5.8
5.6
5.4
5.2
Log distribution
−4
−2
0
2
4
Theoretical quantiles

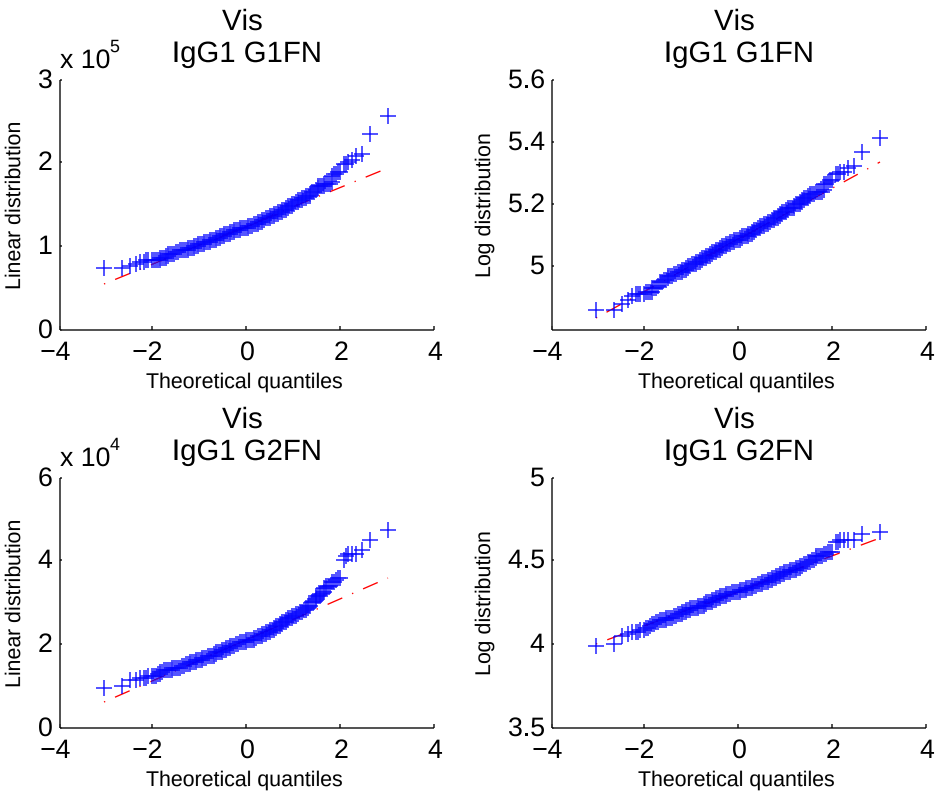

Vis
IgG1 G1FN
x 10^5
Linear distribution
Theoretical quantiles
Vis
IgG1 G1FN
Log distribution
Theoretical quantiles
Vis
IgG1 G2FN
x 10^4
Linear distribution
Theoretical quantiles
Vis
IgG1 G2FN
Log distribution
Theoretical quantiles

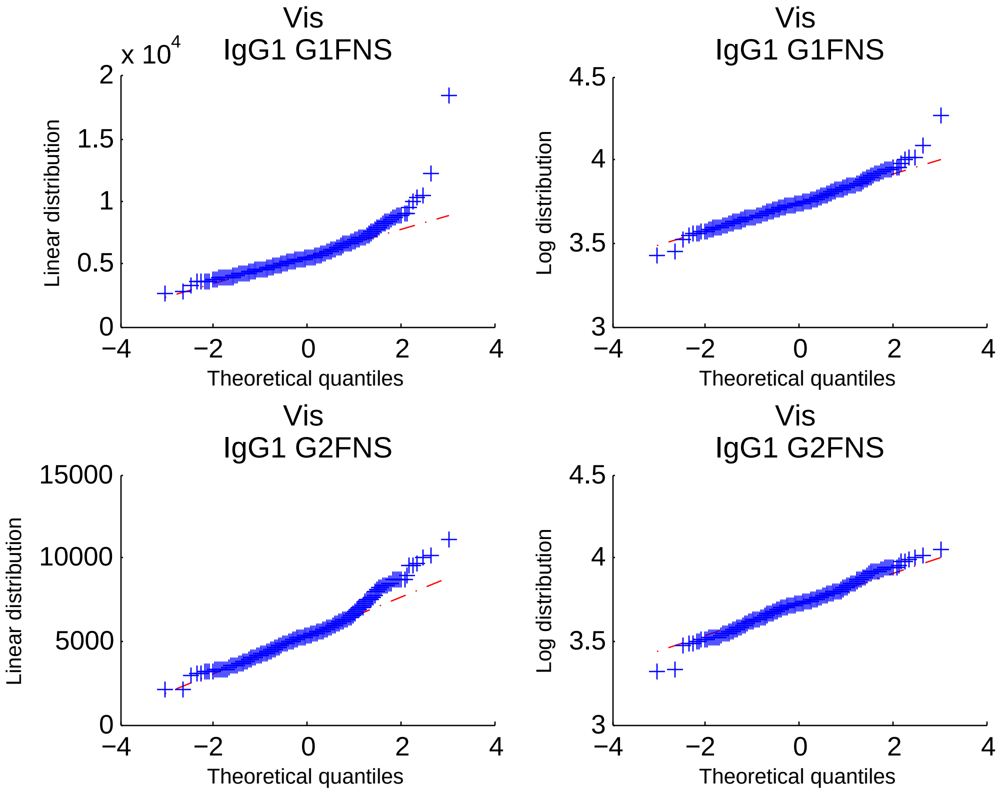
Vis
IgG1 G1FNS
x 10^4
Linear distribution
Theoretical quantiles
Vis
IgG1 G1FNS
Log distribution
Theoretical quantiles
Vis
IgG1 G2FNS
Linear distribution
Theoretical quantiles
Vis
IgG1 G2FNS
Log distribution
Theoretical quantiles

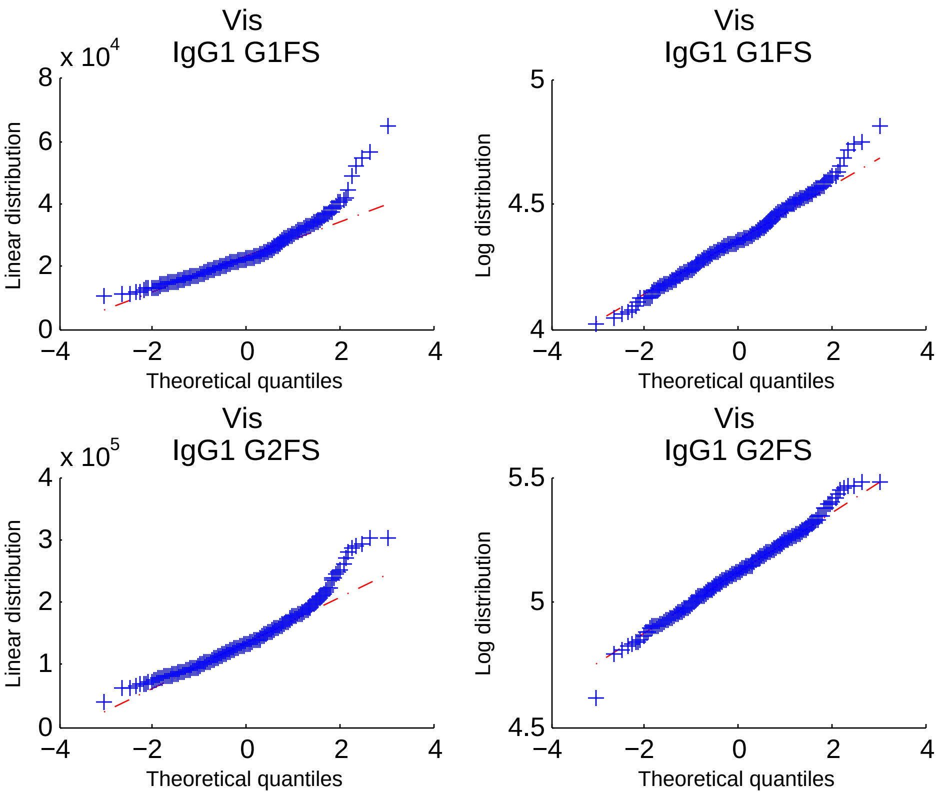

Vis
IgG1 G1FS
x 10^4
Linear distribution
Theoretical quantiles
Vis
IgG1 G1FS
Log distribution
Theoretical quantiles
Vis
IgG1 G2FS
x 10^5
Linear distribution
Theoretical quantiles
Vis
IgG1 G2FS
Log distribution
Theoretical quantiles

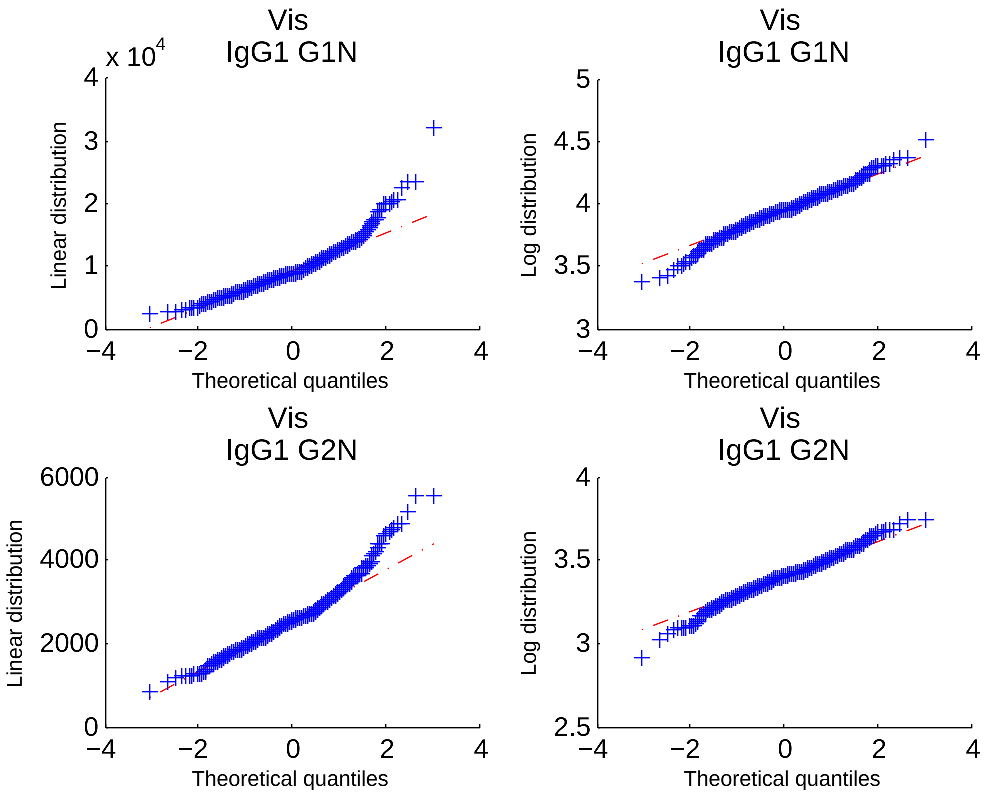
Vis
IgG1 G1N
x 10^4
Linear distribution
Theoretical quantiles
Vis
IgG1 G1N
Log distribution
Theoretical quantiles
Vis
IgG1 G2N
Linear distribution
Theoretical quantiles
Vis
IgG1 G2N
Log distribution
Theoretical quantiles

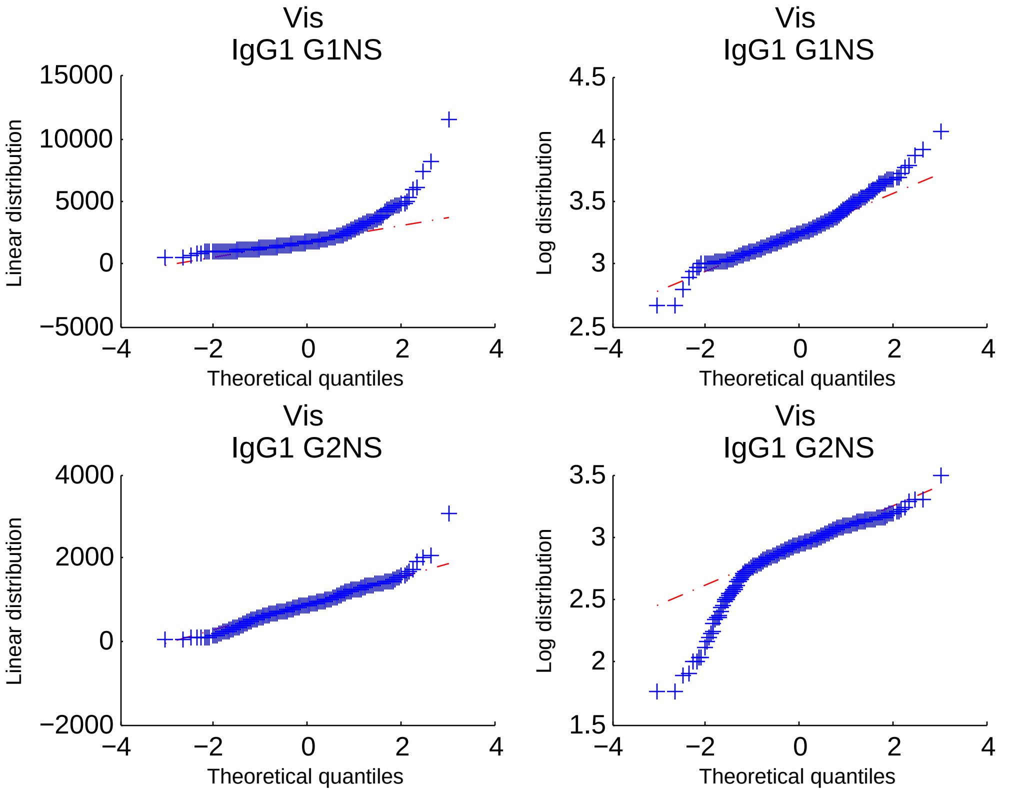

Vis
IgG1 G1NS
Linear distribution
Theoretical quantiles
15000
10000
5000
0
−5000
−4
−2
0
2
4
Vis
IgG1 G1NS
Log distribution
Theoretical quantiles
4.5
4
3.5
3
2.5
Vis
IgG1 G2NS
Linear distribution
Theoretical quantiles
4000
2000
0
−2000
Vis
IgG1 G2NS
Log distribution
Theoretical quantiles
3.5
3
2.5
2
1.5

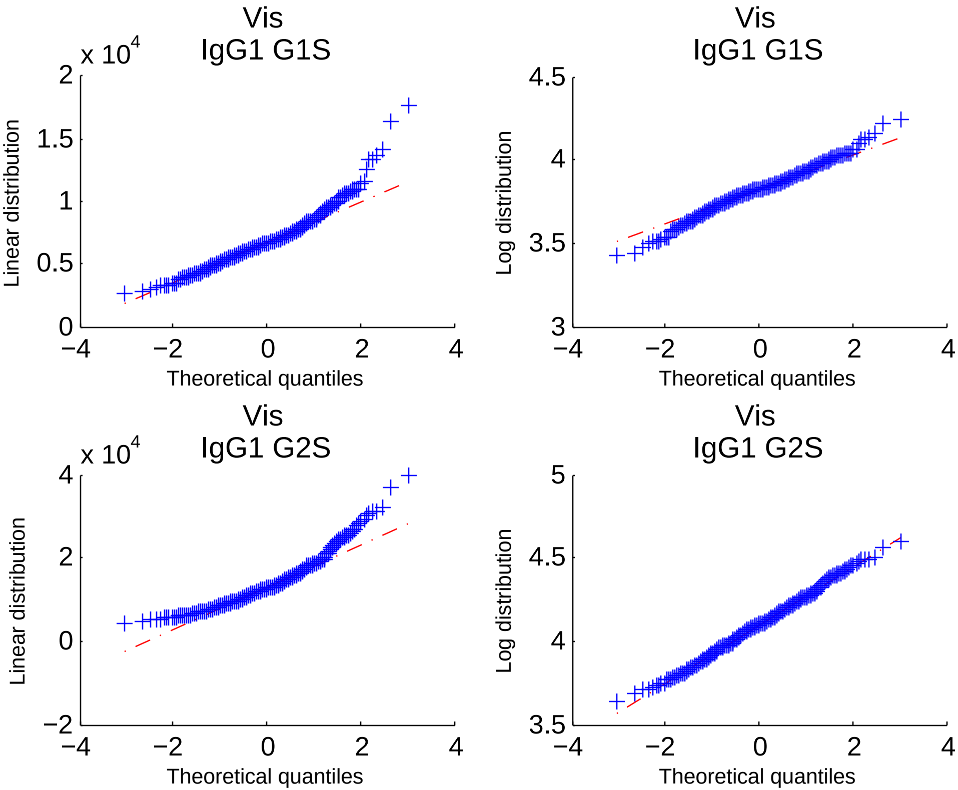
Vis
IgG1 G1S
x 10^4
2
1.5
1
0.5
0
−4
−2
0
2
4
Linear distribution
Theoretical quantiles
Vis
IgG1 G1S
4.5
4
3.5
3
Log distribution
Theoretical quantiles
Vis
IgG1 G2S
x 10^4
4
2
0
−2
Linear distribution
Theoretical quantiles
Vis
IgG1 G2S
5
4.5
4
3.5
Log distribution
Theoretical quantiles

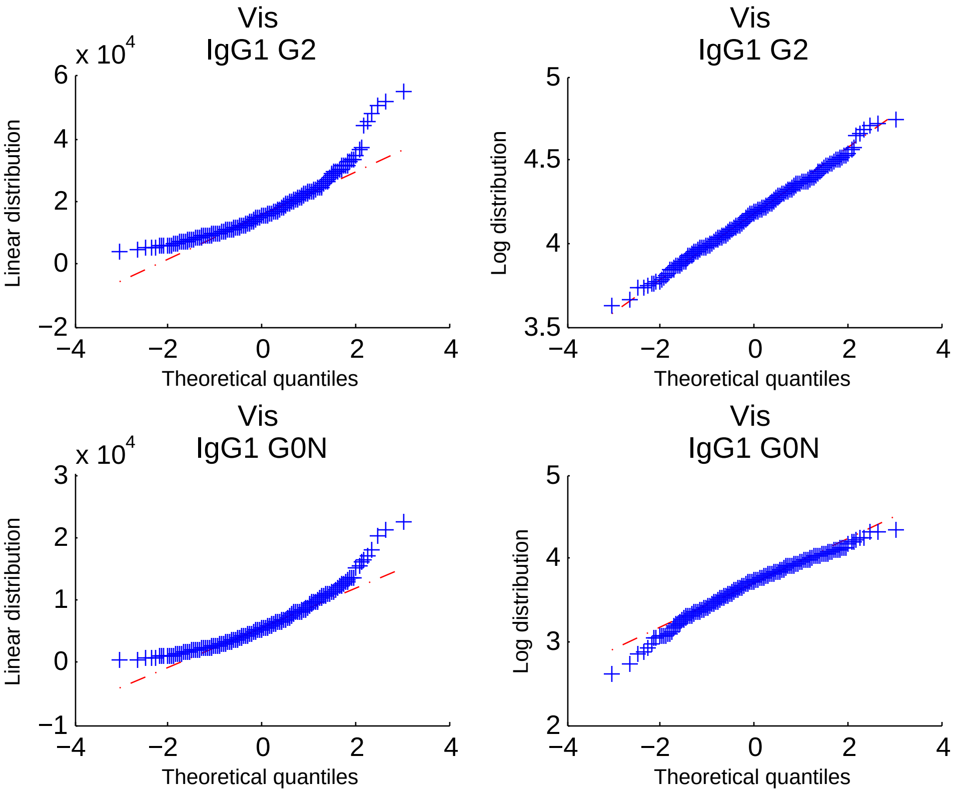
Vis
IgG1 G2
x 10^4
Linear distribution
Theoretical quantiles
Vis
IgG1 G2
Log distribution
Theoretical quantiles
Vis
IgG1 G0N
x 10^4
Linear distribution
Theoretical quantiles
Vis
IgG1 G0N
Log distribution
Theoretical quantiles

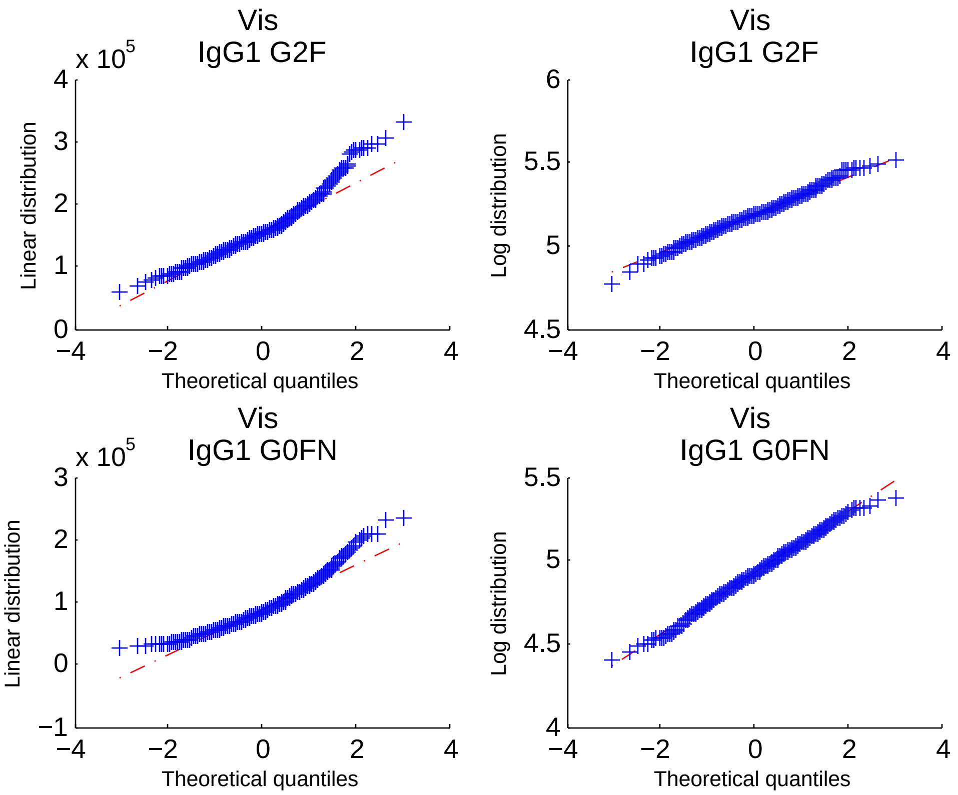
Vis
IgG1 G2F
x 10^5
Linear distribution
Theoretical quantiles
Vis
IgG1 G2F
Log distribution
Theoretical quantiles
Vis
IgG1 G0FN
x 10^5
Linear distribution
Theoretical quantiles
Vis
IgG1 G0FN
Log distribution
Theoretical quantiles

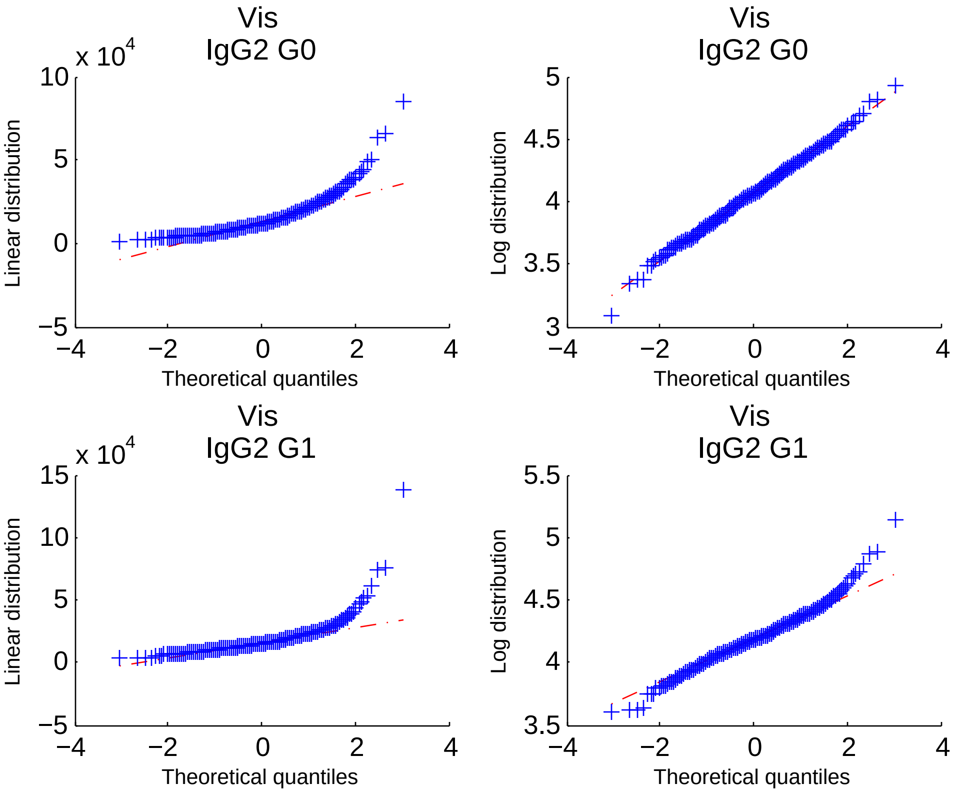

Vis
IgG2 G0
x 10^4
10
5
0
−5
Linear distribution
−4
−2
0
2
4
Theoretical quantiles
Vis
IgG2 G0
5
4.5
4
3.5
3
Log distribution
−4
−2
0
2
4
Theoretical quantiles
Vis
IgG2 G1
x 10^4
15
10
5
0
−5
Linear distribution
−4
−2
0
2
4
Theoretical quantiles
Vis
IgG2 G1
5.5
5
4.5
4
3.5
Log distribution
−4
−2
0
2
4
Theoretical quantiles

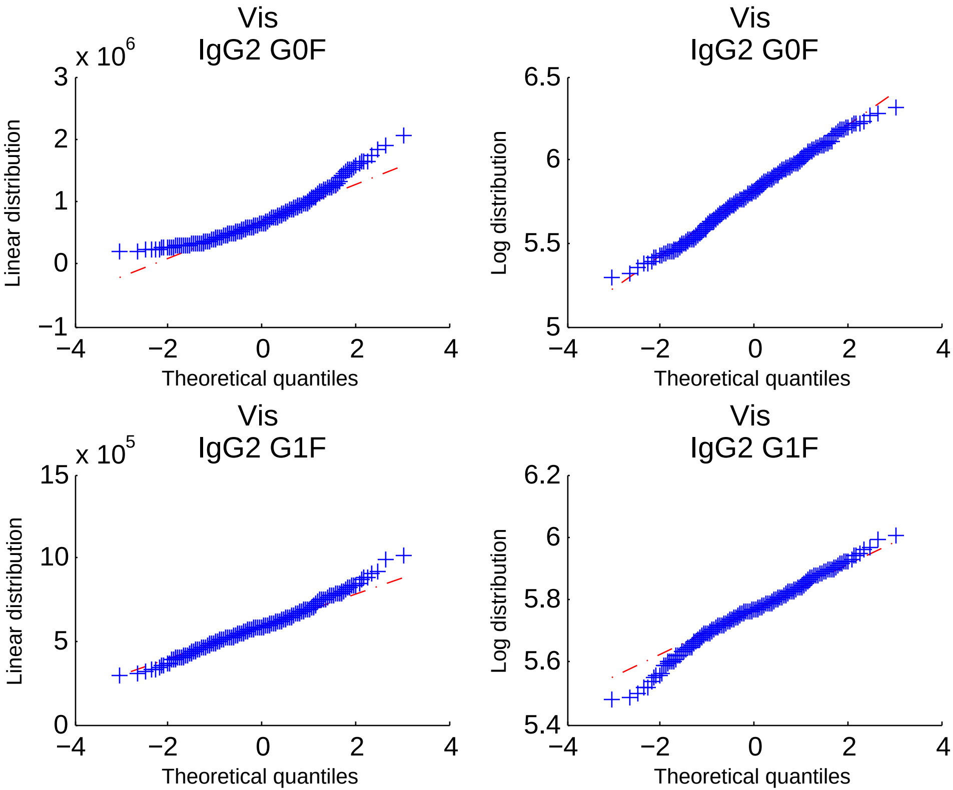

Vis
IgG2 G0F
x 10^6
Linear distribution
Theoretical quantiles
Vis
IgG2 G0F
Log distribution
Theoretical quantiles
Vis
IgG2 G1F
x 10^5
Linear distribution
Theoretical quantiles
Vis
IgG2 G1F
Log distribution
Theoretical quantiles

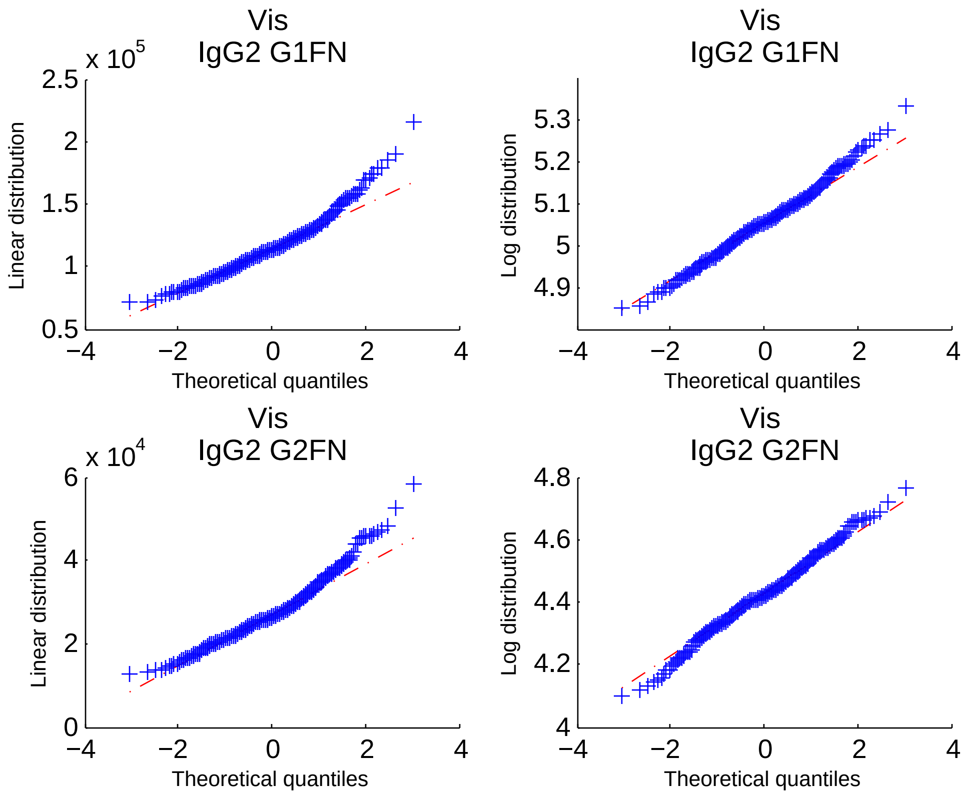
Vis
IgG2 G1FN
x 10^5
Linear distribution
Theoretical quantiles
Vis
IgG2 G1FN
Log distribution
Theoretical quantiles
Vis
IgG2 G2FN
x 10^4
Linear distribution
Theoretical quantiles
Vis
IgG2 G2FN
Log distribution
Theoretical quantiles

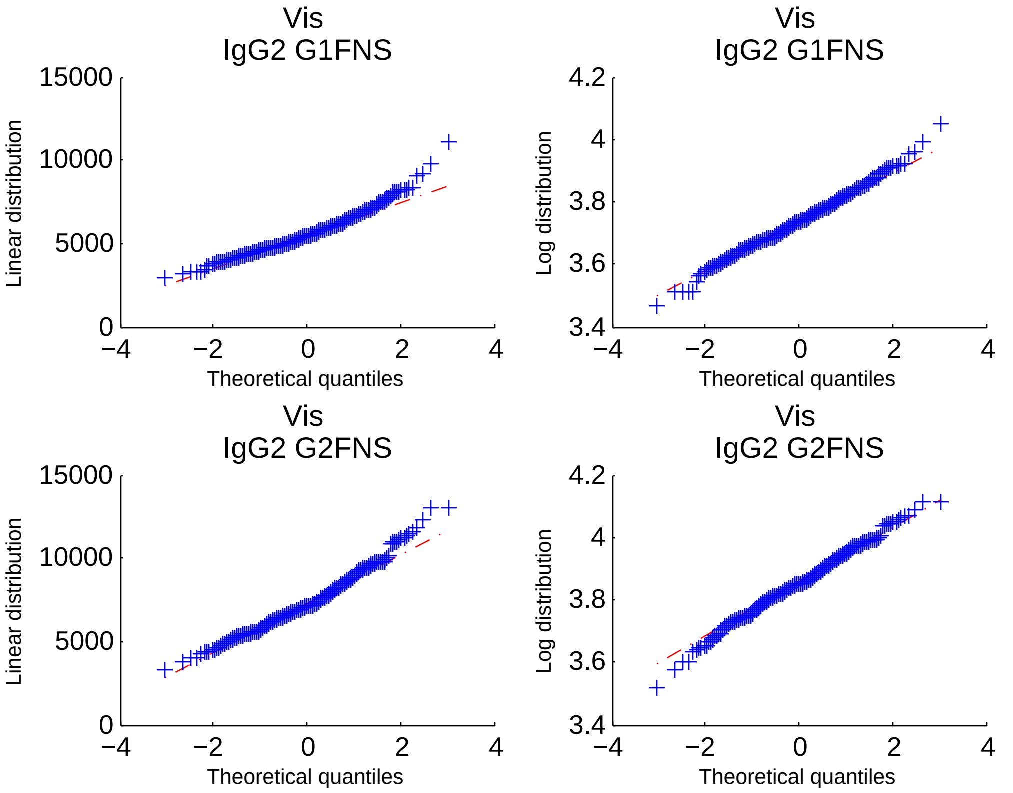
Vis
IgG2 G1FNS
Linear distribution
15000
10000
5000
0
−4
−2
0
2
4
Theoretical quantiles
Vis
IgG2 G1FNS
Log distribution
4.2
4
3.8
3.6
3.4
−4
−2
0
2
4
Theoretical quantiles
Vis
IgG2 G2FNS
Linear distribution
15000
10000
5000
0
−4
−2
0
2
4
Theoretical quantiles
Vis
IgG2 G2FNS
Log distribution
4.2
4
3.8
3.6
3.4
−4
−2
0
2
4
Theoretical quantiles

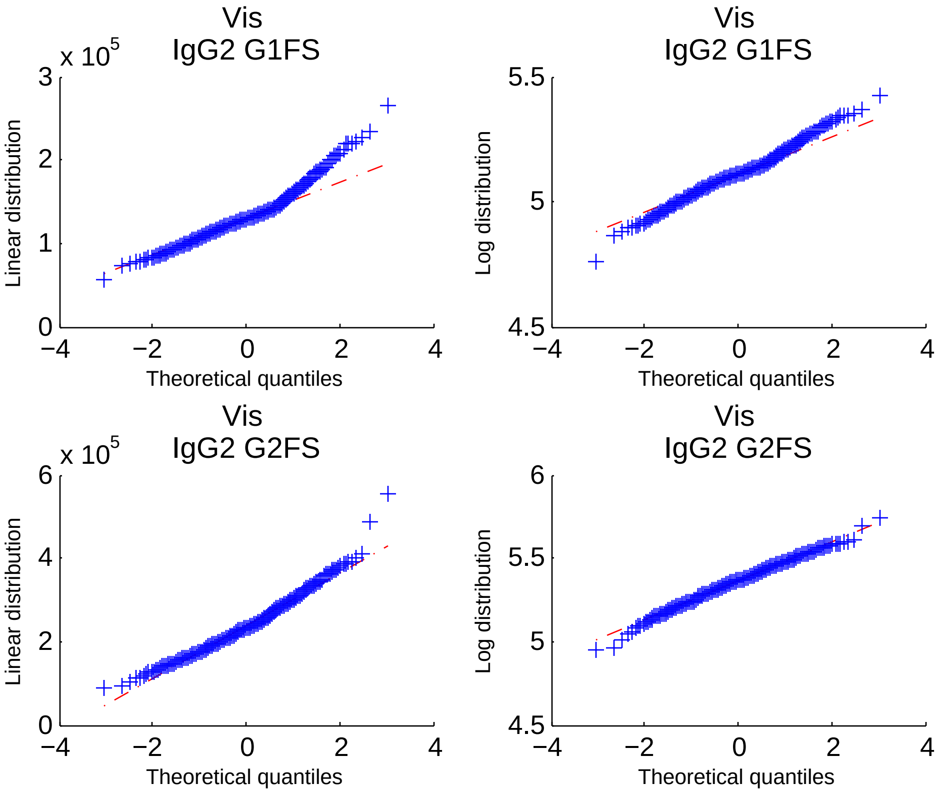
Vis
IgG2 G1FS
x 10^5
Linear distribution
Theoretical quantiles
Vis
IgG2 G1FS
Log distribution
Theoretical quantiles
Vis
IgG2 G2FS
x 10^5
Linear distribution
Theoretical quantiles
Vis
IgG2 G2FS
Log distribution
Theoretical quantiles

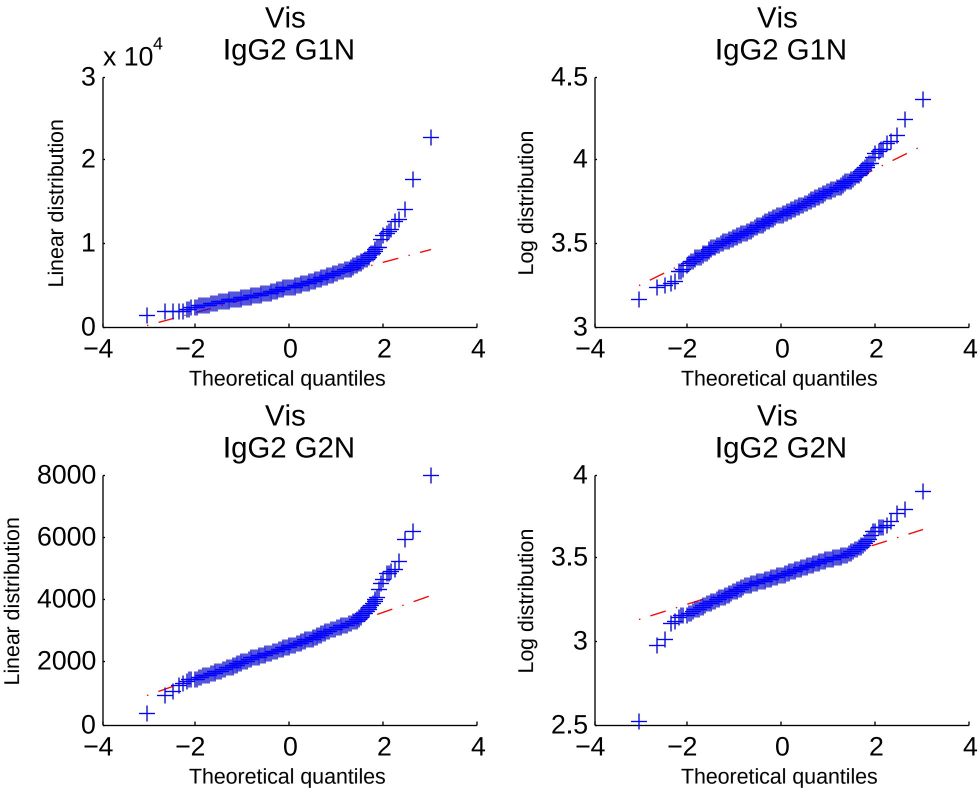

Vis
IgG2 G1N
x 10^4
3
2
1
0
−4
−2
0
2
4
Linear distribution
Theoretical quantiles
Vis
IgG2 G1N
4.5
4
3.5
3
Log distribution
Theoretical quantiles
Vis
IgG2 G2N
8000
6000
4000
2000
0
Linear distribution
Theoretical quantiles
Vis
IgG2 G2N
4
3.5
3
2.5
Log distribution
Theoretical quantiles

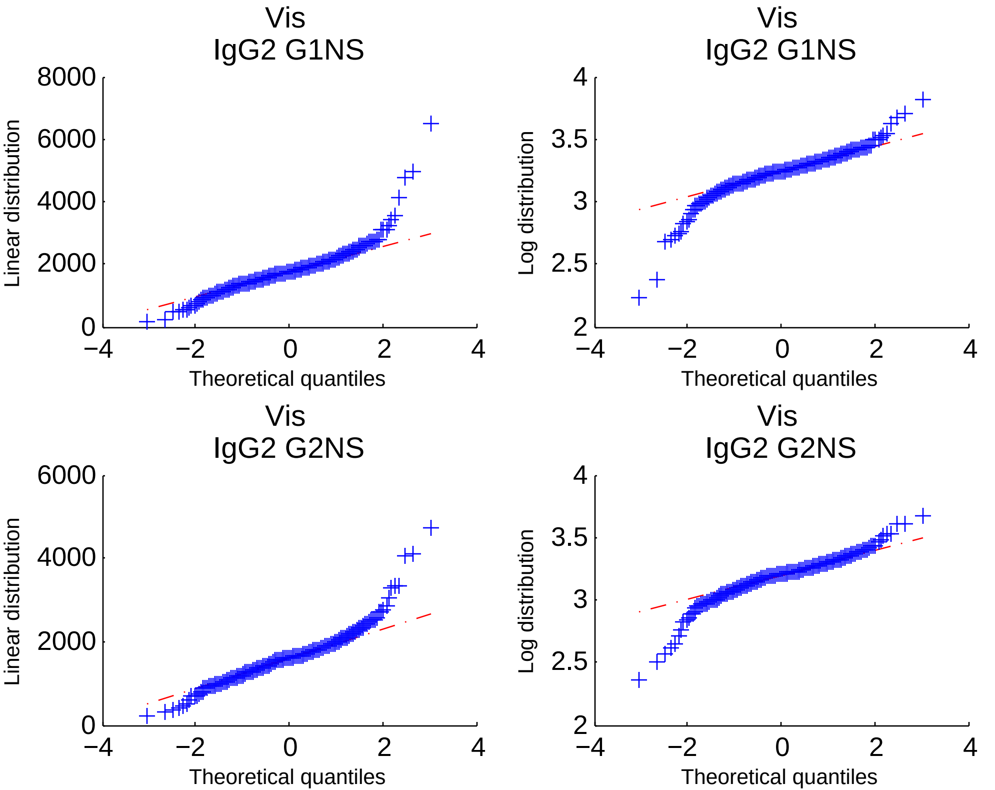

Vis
IgG2 G1NS
Linear distribution
Theoretical quantiles
8000
6000
4000
2000
0
−4
−2
0
2
4
Vis
IgG2 G1NS
Log distribution
Theoretical quantiles
4
3.5
3
2.5
2
Vis
IgG2 G2NS
Linear distribution
Theoretical quantiles
6000
4000
2000
0
Vis
IgG2 G2NS
Log distribution
Theoretical quantiles

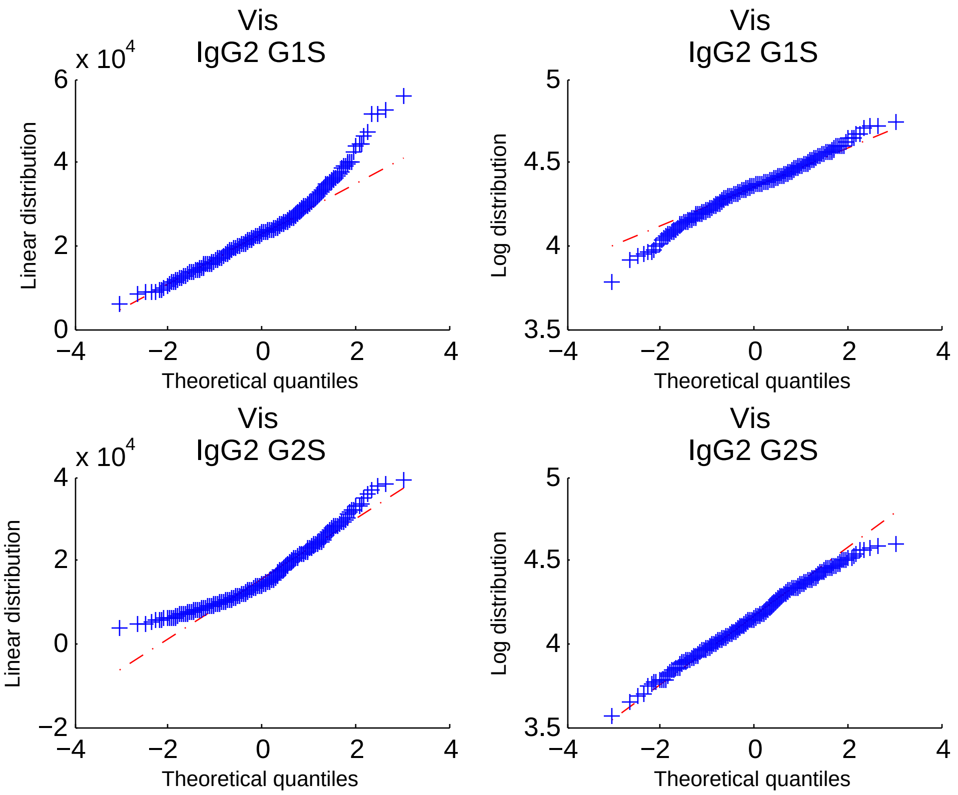
Vis
IgG2 G1S
x 10^4
Linear distribution
Theoretical quantiles
Vis
IgG2 G1S
Log distribution
Theoretical quantiles
Vis
IgG2 G2S
x 10^4
Linear distribution
Theoretical quantiles
Vis
IgG2 G2S
Log distribution
Theoretical quantiles

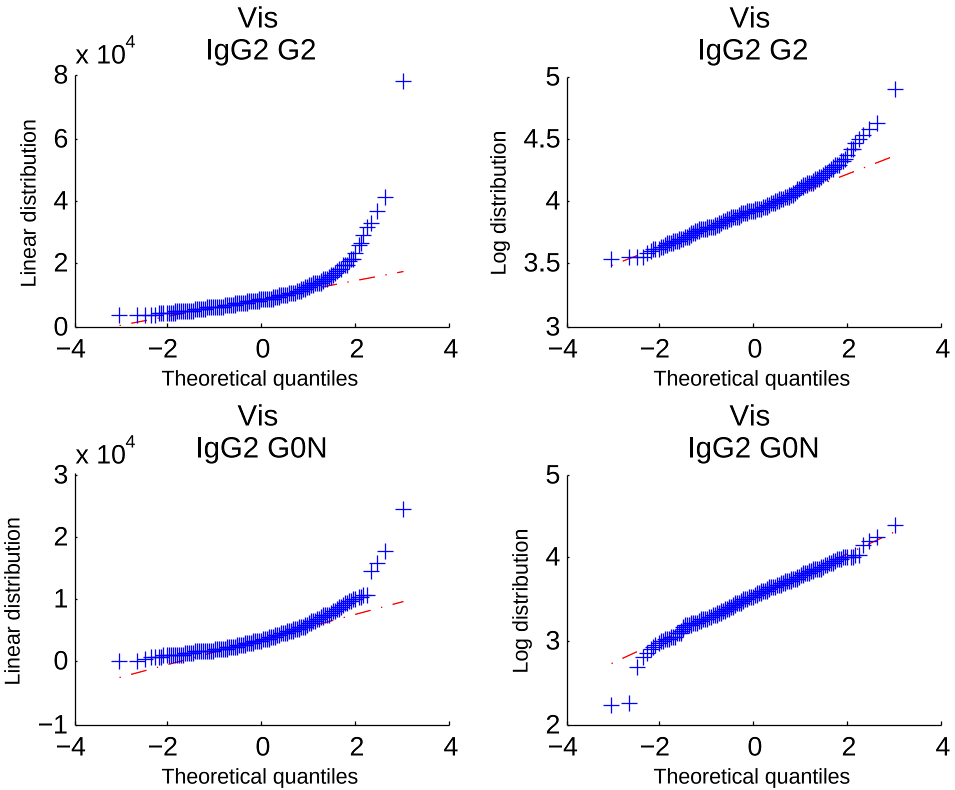
Vis
IgG2 G2
x 10^4
Linear distribution
Theoretical quantiles
Vis
IgG2 G2
Log distribution
Theoretical quantiles
Vis
IgG2 G0N
x 10^4
Linear distribution
Theoretical quantiles
Vis
IgG2 G0N
Log distribution
Theoretical quantiles

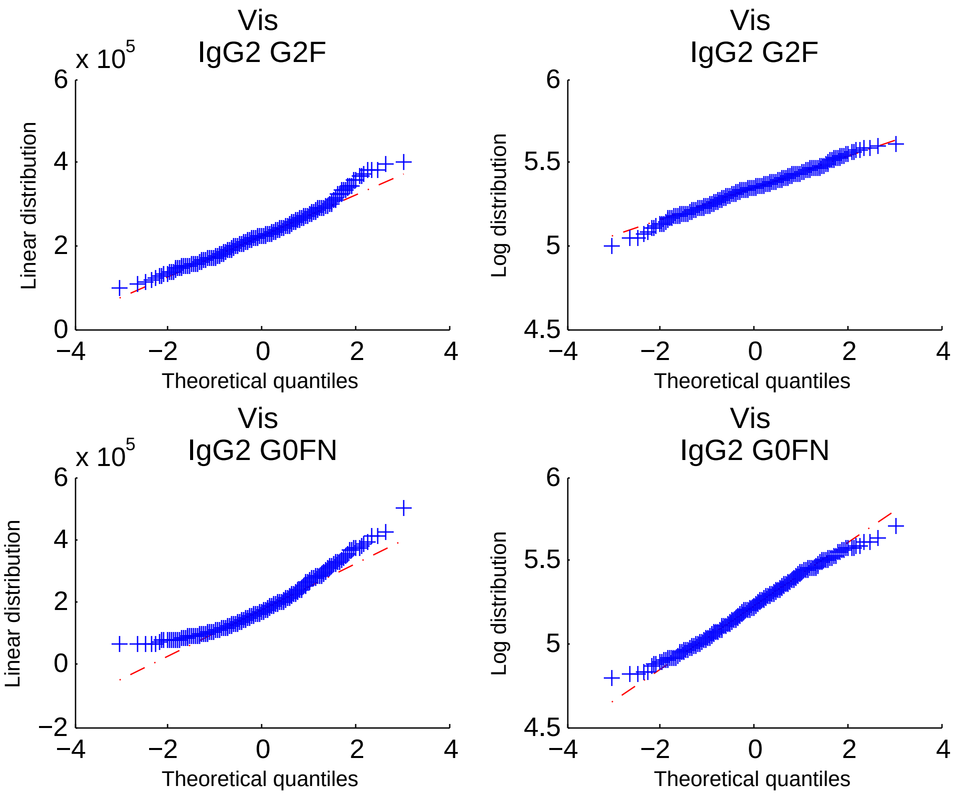
Vis
IgG2 G2F
x 10^5
6
4
2
0
Linear distribution
−4
−2
0
2
4
Theoretical quantiles
Vis
IgG2 G2F
6
5.5
5
4.5
Log distribution
−4
−2
0
2
4
Theoretical quantiles
Vis
IgG2 G0FN
x 10^5
6
4
2
0
−2
Linear distribution
−4
−2
0
2
4
Theoretical quantiles
Vis
IgG2 G0FN
6
5.5
5
4.5
Log distribution
−4
−2
0
2
4
Theoretical quantiles

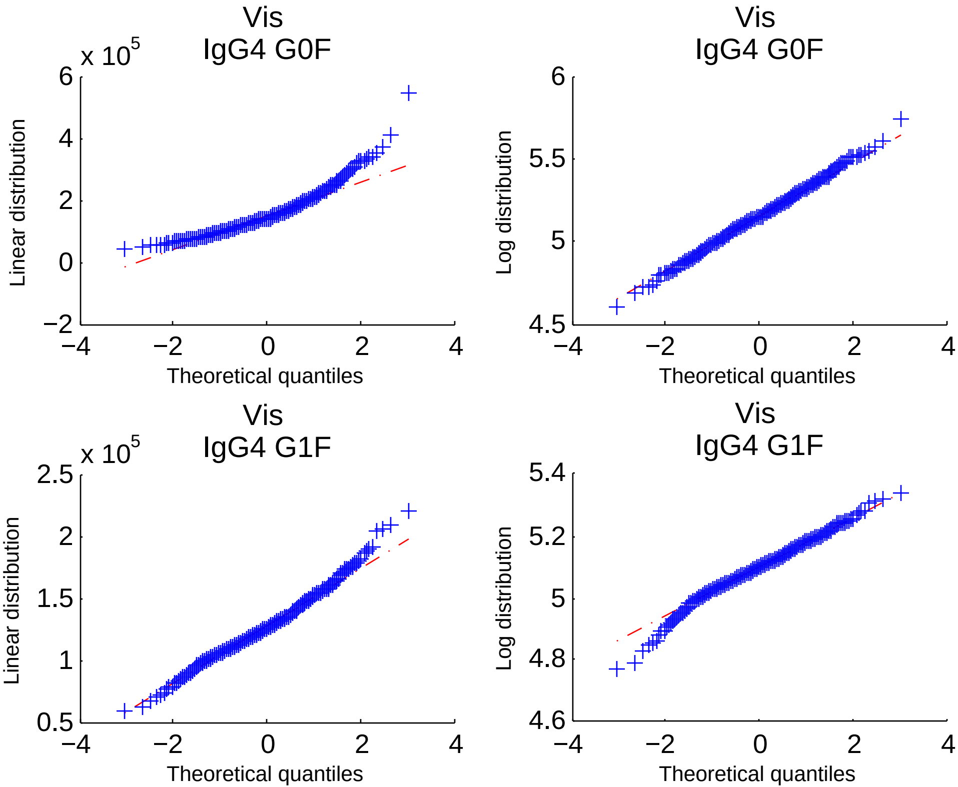
Vis
IgG4 G0F
x 10^5
Linear distribution
Theoretical quantiles
Vis
IgG4 G0F
Log distribution
Theoretical quantiles
Vis
IgG4 G1F
x 10^5
Linear distribution
Theoretical quantiles
Vis
IgG4 G1F
Log distribution
Theoretical quantiles

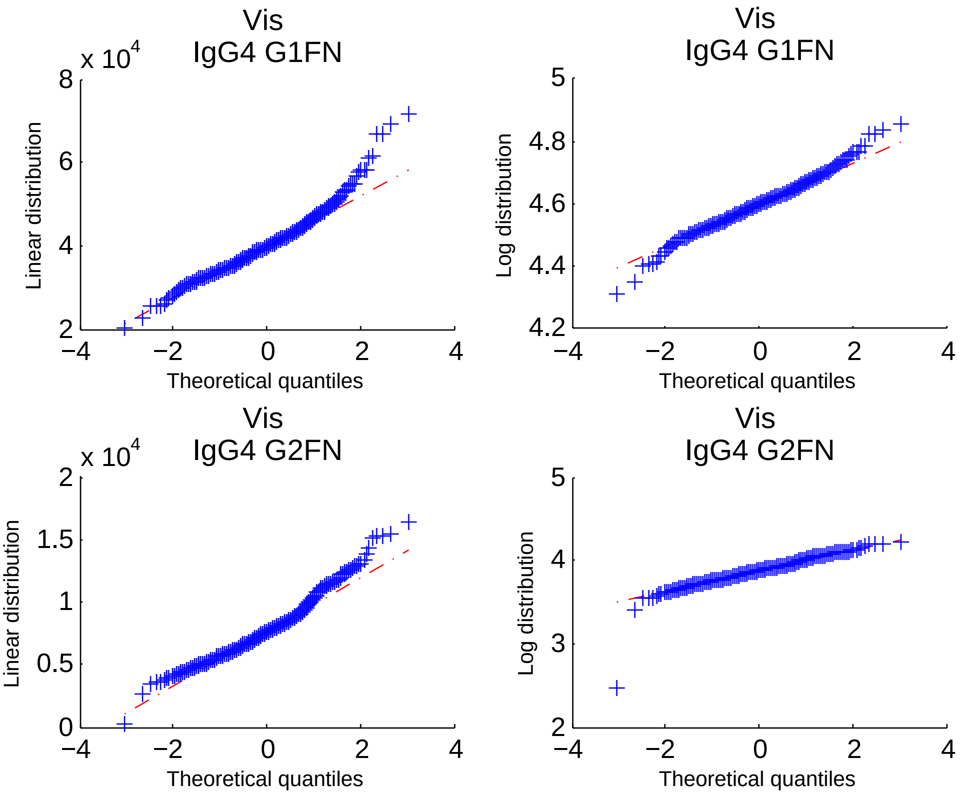
Vis
IgG4 G1FN
x 10^4
Linear distribution
Theoretical quantiles
8
6
4
2
−4
−2
0
2
4
Vis
IgG4 G1FN
Log distribution
Theoretical quantiles
5
4.8
4.6
4.4
4.2
Vis
IgG4 G2FN
x 10^4
Linear distribution
Theoretical quantiles
2
1.5
1
0.5
0
Vis
IgG4 G2FN
Log distribution
Theoretical quantiles
5
4
3
2

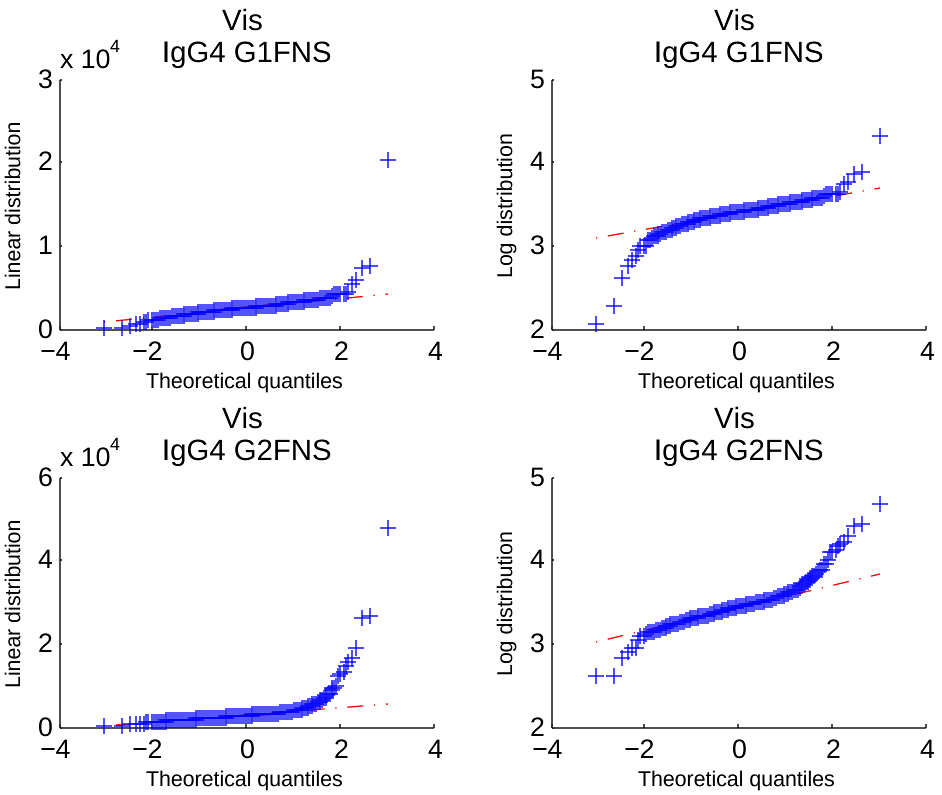
Vis
IgG4 G1FNS
x 10^4
Linear distribution
Theoretical quantiles
Vis
IgG4 G1FNS
Log distribution
Theoretical quantiles
Vis
IgG4 G2FNS
x 10^4
Linear distribution
Theoretical quantiles
Vis
IgG4 G2FNS
Log distribution
Theoretical quantiles

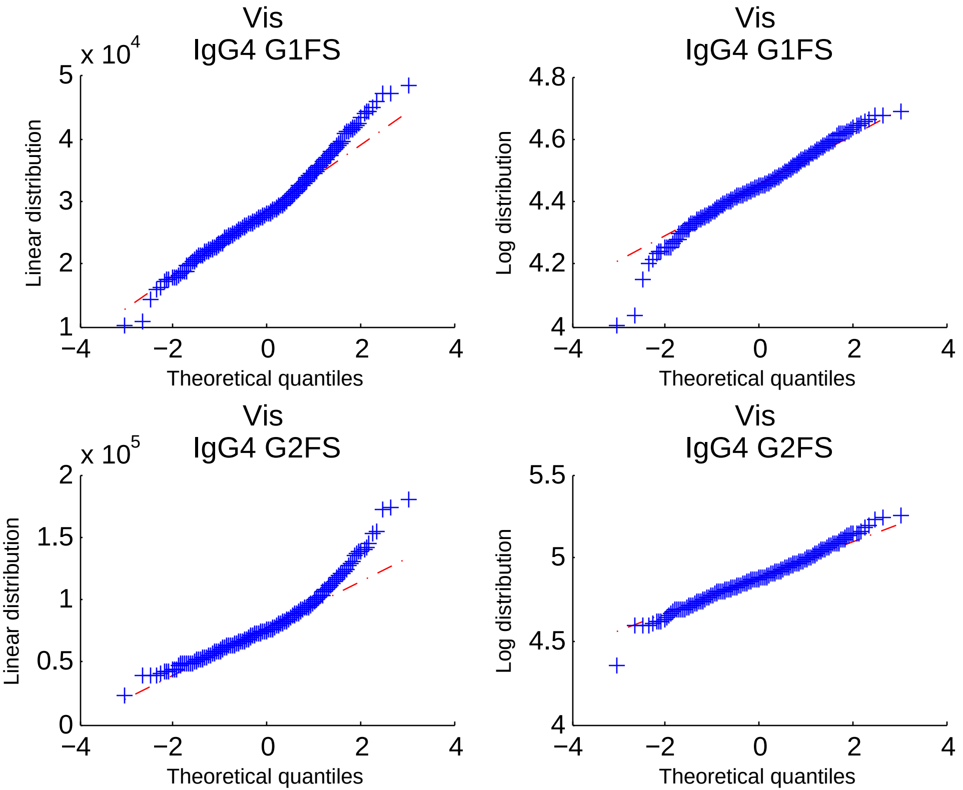

Vis
IgG4 G1FS
x 10^4
Linear distribution
Theoretical quantiles
Vis
IgG4 G1FS
Log distribution
Theoretical quantiles
Vis
IgG4 G2FS
x 10^5
Linear distribution
Theoretical quantiles
Vis
IgG4 G2FS
Log distribution
Theoretical quantiles

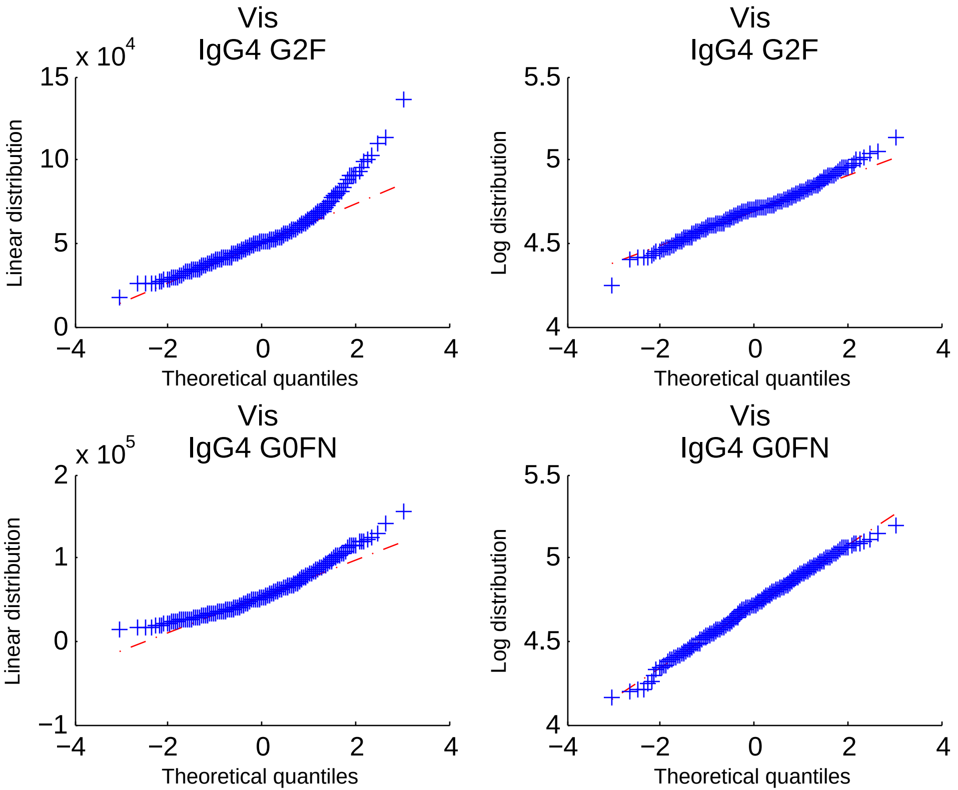
Vis
IgG4 G2F
x 10^4
Linear distribution
Theoretical quantiles
Vis
IgG4 G2F
Log distribution
Theoretical quantiles
Vis
IgG4 G0FN
x 10^5
Linear distribution
Theoretical quantiles
Vis
IgG4 G0FN
Log distribution
Theoretical quantiles

Supplementary Figure 10. QQ plots for all glycan structure in the four Croatian cohorts.

For the four glycomics Croatian cohorts and for each measured glycan structures, a quantile-quantile plot of the quantiles of the data versus the theoretical quantiles values from a normal distribution is produced. If the distribution of the data is normal, then the plot appears linear. The plot is shown for both the original scale and the log10 scale. As it can be observed, in most cases the qq plot of the log-transformed data is more linear.

Supplementary Tables

Supplementary Table 1. Gene regions considered for the genome wide association study.

Gene	Chromosome	Position (GRcH37/hg19) (UCSC Genome Browser, including UTRs)	Extended region
ST6Gal1	3	186,648,315 – 186,796,341	186,149,755 – 187,276,889
B4GalT1	9	33,110,639 – 33,167,356	32,617,358 – 33,633,060
FUT8	14	65,877,310 – 66,210,839	64,927,791 – 66,241,518
MGAT3	22	39,883,229 – 39,888,199	39,355,631 – 40,385,440

Supplementary Methods

G0 and G2 glycopeptide preparation

Egg yolks from fresh whole eggs were separated from egg whites carefully, diluted with equal volume of water, and stirred for 1 h at r.t. The homogenous mixture was lyophilized to dryness to give egg yolk powder. Egg yolk powder (200 g) was stirred with diethyl ether (1 L) for 2 h and filtered. The residue was stirred with diethyl ether (1 L) again and filtered. The obtained residue was further stirred with 70% acetone (1 L) for 2h. After filtration, the residue was extracted by 40% acetone (1 L) twice and filtered. The filtrate was concentrated under reduced pressure to dryness, and re-dissolved in 10 mL water. The crude mixture was loaded to an active carbon/celite (2:1) column for purification. The column was eluted with water (0.1% TFA), 5% acetonitrile (0.1% TFA), 10% acetonitrile (0.1% TFA), and 25% acetonitrile (0.1% TFA). The elution fractions containing sialylglycopeptide, detected by ESI-MS, was pooled and lyophilized to give sialylglycopeptide as a white powder (120 mg). The sialylglycopeptide was treated with Neuraminidase (Clostridium perfringens, New England Biolabs) to cleave off the sialic acid to afford G2-SGP after P2 Bio-Gel size exclusion chromatography purification. The G2-SGP was further treated with galactosidase (Aspergillus niger, Megazyme) to afford the G0-SGP after P2 Bio-Gel size exclusion chromatography purification.

IgG isolation

IgG was isolated from human plasma by affinity chromatography using 96-well protein G monolithic plates (BIA Separations, Ajdovščina, Slovenia). Before isolating the IgG from human plasma, the protein G plate was prepared by washing with 10 column volumes (CV) of ultrapure water, followed by 10 CV of the binding buffer 1 x PBS (137 mM NaCl, 2.7 mM Na_2HPO4, 9.7 mM KH_2PO_4, 2.2 mM KCl, titrated with NaOH to pH 7.4) and then with 5 CV of 0.1 M FA (pH 2.5). Afterwards, the protein G plate was equilibrated with 10 CV of 10 x PBS pH 6.6 and 20 CV of 1 x PBS.

All human plasma samples (100 µl) were defrosted and centrifuged at 1620 g for 10 min (centrifuge 5804, rotor A-4-44, Eppendorf, Hamburg, Germany). Afterwards the plasma samples were diluted 8x (KORA) or 10x (Croatian cohorts) with binding buffer and filtered through 0.45 µm GHP AcroPrep 96-well filter plates. Plasma samples were applied to the protein G plate and instantly washed three times with 30 CV of 1 x PBS to remove unbound proteins. IgG was eluted from the protein G monoliths using 5 CV of 0.1 M formic acid (pH 2.5), into a 96-deep-well plate (Waters, Milford Massachusetts, USA) and immediately neutralized to pH 7.0 with 1 M ammonium bicarbonateto maintain the IgG stability.

After IgG elution, protein G platewas regenerated by washing with the following buffers: 10 CV 0.1 M FA followed by 10 CV of 10 × PBS and afterwards 20 CV of 1 × PBS to re-equilibrate the monoliths. Each step of the isolation was done under vacuum (380 mmHg during washing steps) using a manual set-up consisting of a multichannel pipette, a vacuum manifold (Pall, Ann Arbor, MI, USA) and a vacuum pump (Pall Life Sciences, Ann Arbor, MI, USA). If the plate was not immediately re-used , it was stored in a storage buffer (20% ethanol (v/v), 20 mM Tris, 0.1 mM NaCl, titrated with HCl to pH 7.4) at 4 °C.

Glycopeptide analysis

IgG tryptic digestion and purification

Isolated IgG (approximately 25 µg) was applied to to 96-well PCR plate. Trypsin (Worthington, USA) was disolved in ice-cold 20 mM acetic acid (Merck, Darmstadt, Germany) to a final concentration of 0.2 µg/µl after which it was further diluted to 0.02 µg/µl with ice-cold ultrapure water. To each sample 10 µl of the diluted trypsin was added followed by overnight incubation at 37°C. For reverse-phase desalting and purification of glycopeptides, 5 mg of Chromabond C18ec beads (Marcherey-Nagel, Düren, Germany) were applied to each well of an OF1100 96-well polypropylene filter plate with a 10 µm polyethylene frit (Orochem Technologies Inc., Lombard, IL, USA). The RP stationary phase was activated with 3× 200 µl 80 % ACN containing 0.1 % trifluoroacetic acid (TFA; Fluka, Steinheim, Germany) and conditioned with 3× 200 µl 0.1 % TFA. The IgG digests were diluted 10× in 0.1 % TFA, loaded onto the C18 beads, and washed with 3× 200 µl 0.1 % TFA. The entire procedure was performed on a vacuum manifold (< 3 mmHg). IgG glycopeptides were eluted into a V-bottom microtiter plate by centrifugation at 500 rpm with 90 µl (for the Croatian cohorts) or 120 µL (for KORA) of 18 % ACN containing 0.1 % TFA. Eluates were dried by vacuum centrifugation, reconstituted in 20 µl MQ water and stored at -20°C until analysis by MS.

LC-ESI-MS/MS analysis of IgG tryptic glycopeptides

Croatian cohorts

IgG glycopeptide samples were analysed using an Ultimate 3000 HPLC system (Dionex Corporation, Sunnyvale, CA, USA), consisting of a degasser unit, binary loading pump, dual binary gradient pump, autosampler maintained at 5°C and fitted with a 10µl PEEK sample loop, and two column oven compartments set at 30°C. To protect the trap and analytical column for particulates, samples were centrifuged at 4000 rpm for 5 min and passed through a 2 µm pore size stainless steel frit mounted between the autosampler transfer tubing and the trap column. Samples (250-5000 nl) were applied to a Dionex Acclaim PepMap100 C18 (5 mm x 300 µm i.d.) SPE trap column conditioned with 0.1 % TFA (mobile phase A) for 1 min at 25 µl/min. After sample loading the trap column was switched in-line with the gradient and Ascentis Express C18 nano-LC column (50 mm x 75 µm i.d., 2.7 µm HALO fused core particles; Supelco, Bellefonte, USA) for 8 min while sample elution took place. This was followed by an off-line cleaning of the trap column with three full loop injections containing 5 µl 5 % isopropanol (IPA) + 0.1 % FA and 5 µl 50 % IPA + 0.1 % FA. On-column separation was achieved at 900 nl/min using the following gradient of mobile phase A and 95 % ACN (Biosolve BV, Valkenswaard, the Netherlands; mobile phase B): 0 min 3 % B, 2 min 5 % B, 5 min 20 % B, 6 min 30 % B, 8 min 30 % B, 9 min 0 % B, and 14 min 0 % B. The separation was coupled to a quadrupole-TOF-MS (micrOTOF-Q; Bruker Daltonics) equipped with a standard ESI source (Bruker Daltonics) and a sheath-flow ESI sprayer (capillary electrophoresis ESI-MS sprayer; Agilent Technologies, Santa Clara, USA). The column outlet tubing (20 µm i.d., 360 µm o.d.) was directly applied as sprayer needle. A 2 µl/min sheath-flow of 50 % IPA, 20 % propionic acid (PA) and 30 % ultrapure water was applied by one of the binary gradient pumps to reduce the TFA gas phase ion pairing and assist with ESI spray formation. A nitrogen stream was applied as dry gas at 4 L/min with a nebulizer pressure of 0.4 bars to improve mobile phase evaporation. Glycan decay during ion transfer was reduced by applying 2 and 4 eV quadrupole ion energy and collision energy, respectively. Scan spectra were recorded from m/z 300 to 2000 with 2 averaged scans at a frequency of 1 Hz. Per sample the total analysis time was 16 min. The software used to operate the Ultimate 3000 HPLC system and the Bruker micrOTOF-Q were Chromeleon Client version 6.8 and micrOTOF control version 2.3, respectively.

Each LC-MS dataset was calibrated internally using a list of known glycopeptides, exported to the open mzXML format by Bruker DataAnalysis 4.0 in batch mode and aligned to a master dataset of a typical sample (containing many of the (glyco)peptide species shared between multiple samples) using msalign2 and a simple warping script in AWK. From each dataset a list of 402 pre-defined features, defined as the peak maximum within mass window of + *m/z* 0.04 and a retention time window of +10, were extracted using the in-house developed "Xtractor2D" software and merged to a complete data matrix. As input, Xtractor2D takes a dataset in the mzXML format aligned to the master dataset and a reference list with pre-defined features with *m/z* windows and retention times in seconds. The theoretical *m/z* values used to identify the glycopeptide features are calculated, and the retention times on the chromatographic time scale of the master dataset are used for the alignment. Due to the use of TFA as ion pairing reagent, all glycopeptides belonging to the same IgG subclass have approximately the same retention time, regardless of the number of *N*-acetylneuraminic acid residues.

KORA

Tryptic glycopeptides were analyzed on nanoACQUITY UPLC system (Waters, USA) coupled to Compact mass spectrometer (Bruker Daltonics, Germany). Nine µL of IgG tryptic glycopeptides was loaded on Acclaim PepMap100 C8 (5mm×300 µm i.d.) trap column. The glycopeptides were washed 1 min with 0.1% TFA (solvent A) at a flow rate of 40 µL/min and separated on a HALO C18 nano-LC column (50mm×75 µm i.d., 2.7 µm HALO fused core particles) (Advanced Materials technology, USA) at 30°C, using a 3.5 min gradient from 19% to 25% solvent B (80% ACN) at 1 µL/min flow rate. Mass spectra were acquired from 500 to 2000 *m/z* units with 2 averages at a frequency of 0.5 Hz. The quadrupole ion energy and collision energy were set to 4 eV. NanoACQUITY UPLC system was operated under MassLynx software version 4.1 and the Bruker micrOTOF-Q was operated under HyStar software, version 3.2.

Data extraction was performed using an in-house Python script. In short, data were *m/z* recalibrated based on a subset of hand-picked analytes having a high signal-to-noise ratio and the expected isotopic distribution. Intensities for the top four isotopologues were extracted using a 10 ppm *m/z* window. Retention times were aligned towards the cohort median and retention time bins were determined for the analytes. All of the signals belonging to a single analyte for every sample were summed up.

Synthetic substrate preparation

BODIPY derivatives of 9-OS and 10-OS-Gly, chemical structures

(1) 11-OS-Gly

Neu5Acα2-6Galβ1-4GlcNAcβ1-2Manα1-3

Manβ1-4GlcNAcβ1-4GlcNAcβ1-NHCOCH$_2$NH$_2$

Neu5Acα2-6Galβ1-4GlcNAcβ1-2Manα1-6

(2) 9-OS-Gly

Galβ1-4GlcNAcβ1-2Manα1-3

Manβ1-4GlcNAcβ1-4GlcNAcβ1-NHCOCH$_2$NH$_2$

Galβ1-4GlcNAcβ1-2Manα1-6

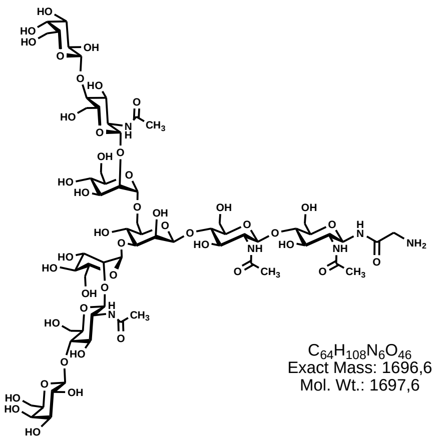

(3) 10-OS-Gly (mixture of two isomers)

Neu5Acα2-6Galβ1-4GlcNAcβ1-2Manα1-3\
Manβ1-4GlcNAcβ1-4GlcNAcβ1-NHCOCH$_2$NH$_2$\
Galβ1-4GlcNAcβ1-2Manα1-6

B

+

Galβ1-4GlcNAcβ1-2Manα1-3\
Manβ1-4GlcNAcβ1-4GlcNAcβ1-NHCOCH$_2$NH$_2$\
Neu5Acα2-6Galβ1-4GlcNAcβ1-2Manα1-6

A

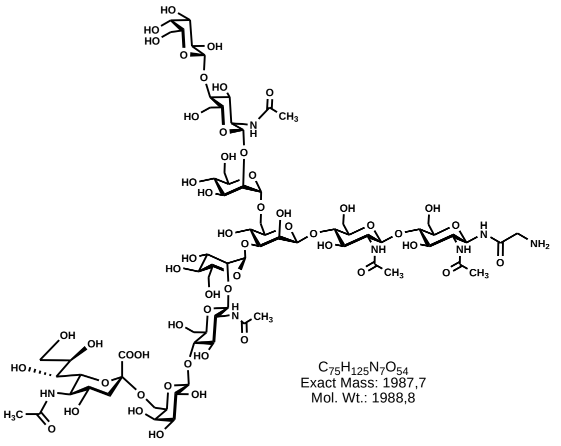

(4) 9-OS-Gly-BODIPY

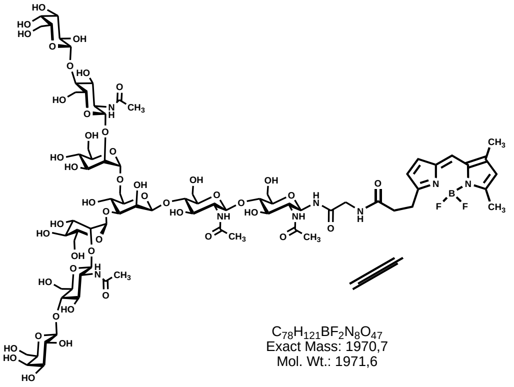

(5) 10-OS-Gly-BODIPY (B isomer)

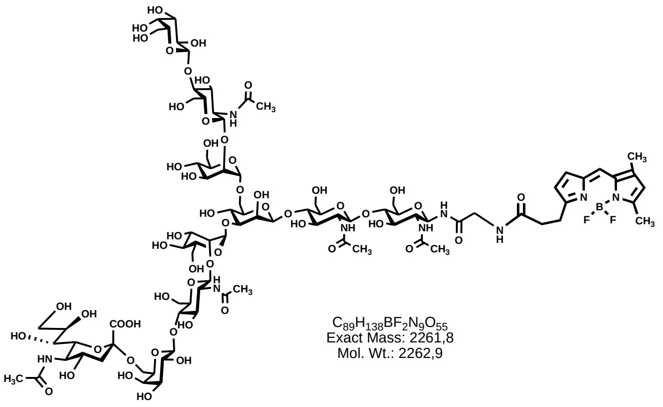

Synthesis

Monosialo-oligosaccharide 10-OS-Gly (**3**) was obtained from disialo-oligosaccharide[16] (**1**) by treatment with *Vibrio cholerae* neuraminidase [Sigma (USA)]. The reaction was carried out in buffer containing 0.1 M NaOAc and 2 mM $CaCl_2$ (pH 5.4) at 37° C, the concentration of oligosaccharide was 2 µmol/ml, the concentration of enzyme was 0.04 units/ml, resulting in an oligosaccharide/enzyme ratio of 50:1. After 3h incubation the target monosialo-oligosaccharide 10-OS-Gly (**3**) was observed as main product (TLC-control: MeOH – CH_3CN – H_2O=3:3:2 v/v); the reaction was stopped by addition of TRIS buffer (pH 9.2) and the reacting mixture was subjected to gel chromatography on Sephadex LH-20 (CH_3CN – H_2O=1:1 v/v). Disialo-11-OS-Gly (**1**), monosialo-10-OS-Gly (**3**) (mixture of two isomers) and asialo-9-OS-Gly (**2**) derivatives were separated by ion-exchange chromatography on Dowex 50X4-200 (H^+). Subsequent elution with water, 1 M aqueous pyridine and 1 M aqueous NH_3 gave unreacted 11-OS-Gly (**1**) (17%), target 10-OS-Gly (**3**) (50%) and 9-OS-Gly (**2**) (15%) respectively.

According to ^{1}HMR data, 10-OS-Gly (**3**) is the mixture ~1:1 of two isomers **A** and **B**, differing by the position of the Neu5Ac-residue.

BODIPY-derivatives were synthesized by condensation of 9-OS-Gly (**2**) or 10-OS-Gly (**3**) with 1 eq. of succinimidyl ester of BODIPY (BODIPY-SE) [Succinimidyl ester of 4,4-difluoro-5,7-dimethyl-4-bora-3a,4a-diaza-s-indacene-3-propionic acid (BODIPY FL-SE) from Invitrogen (USA)]. The condensation was carried out in DMSO at room temperature without Et_3N addition for 9-OS-Gly (**2**) and in the presence of 2 eq. Et_3N for 10-OS-Gly (**3**) [17–19]. The reaction was completed in 10 min (TLC-control: MeOH – 1 M aqueous Py•HOAc 5:1 v/v for (**2**), MeOH – CH_3CN – H_2O= 3:3:2 v/v for (**3**)). After gel filtration on Sephadex LH-20 (CH_3CN – H_2O= 1:1 v/v) 9-OS- and 10-OS- BODIPY derivatives (**4**) and (**5**) were isolated in good yields (~95%). The target products were kept in darkness at -18°C.

According to ^{1}HMR data, 10-OS-BODIPY (**5**), analogously to 10-OS-Gly derivative (**3**), is ~1:1 mixture of two isomers **A** and **B**, which could not be separated by HPLC on reversed-phased C18 silica gel (Phenomenex Luna, 4.6x250 mm, 5 µm, pore size 100 Å, elution CH_3CN – 15 mM aqueous $NH_4H_2PO_4$, 20:80) nor on amino-phased silica gel (Phenomenex Luna, 4.6x250 mm, 5 µm, pore size 100Å, elution CH_3CN – 15 mM aqueous $NH_4H_2PO_4$, 30:70).

The structures of the synthesized compounds were confirmed by ^{1}H NMR spectroscopy and mass-spectrometry data.

Experimental Procedure

^{1}H NMR spectra were recorded on a Bruker BioSpin GmbH (700 MHz) spectrometer at 30 °C: chemical shifts (δ, ppm) were referred to the peak of internal D_2O (δ 4.750 ppm), coupling constants (J) were measured in Hz.

High resolution mass-spectra (HR MS) were recorded in the Department of Structural Studies of Zelinsky Institute of Organic Chemistry (Moscow) on a Bruker micrOTOF II instrument using electrospray ionization (ESI).

Details on glycan identification:

(2) 9-OS-NH-Gly

TLC: R_f 0.4 (MeOH – 1 M aqueous Py•HOAc=5:1).

^{1}H NMR (TFA salt), selected chemical shifts (500 MHz, D_2O) δ: 2.022 (s, 3H, NAc), 2.067 (s, 6H, 2NAc), 2.098 (s, 3H, NAc), 4.128 (dd, $J_{2,3}$ =3.3, $J_{1,2}$ =1.3, 1H, H-2 Man), 4.209 (dd, $J_{2,3}$ = 3.5, $J_{1,2}$ =1.5, 1H, H-2 Man), 4.264 (dd, $J_{2,3}$ = 2.5, $J_{1,2}$ = 0.8, 1H, H-2 Man), 4.487 (d, $J_{1,2}$ =7.8, 1H, H-1 Gal), 4.493 (d, $J_{1,2}$ = 7.8, 1H, H-1 Gal), 4.601 (d, $J_{1,2}$ =7.9, 2H, H-1 GlcNAc), 4.635 (d, $J_{1,2}$ =7.7, 1H, H-1 GlcNAc), 4.785 (d, $J_{1,2}$=0.6, 1H, H-1 Man), 4.946 (d, $J_{1,2}$ =1.8, 1H, H-1 Man), 5.139 (d, $J_{1,2}$ =1.5, 1H, H-1 Man), 5.139 (d, $J_{1,2}$ = 9.7 Hz, 1H, H-1 GlcNAc-(NH-Gly)).

HRESIMS: found *m/z* 1697.6347, calc. for $C_{64}H_{108}N_6O_{46}$, $[M+H]^+$: *m/z* 1697.6369; found *m/z* 849.3219, calc. for $[M+2H]^{2+}$: *m/z* 849.3221; found *m/z* 860.3124, calc. for $[M+H+Na]^{2+}$: *m/z* 860.3131; found *m/z* 868.2966, calc. for $[M+H+K]^{2+}$: *m/z* 868.3000; found *m/z* 879.2887, calc. for $[M+Na+K]^{2+}$: *m/z* 879.2910.

(3) 10-OS-NH-Gly

TLC: R_f 0.33 (MeOH – CH_3CN – H_2O= 3:3:2 v/v)

^{1}H NMR, selected chemical shifts (700 MHz, D_2O) δ: 1.737 (t, $J_{3ax,3eq}$= $J_{3ax,4}$ =12.18, 1H, H-3_{ax} Neu5Ac), 2.025 s, 2.053 s, 2.070 s, 2.073 s, 2.088 s, 2.091 s, 2.102 s, 2.105 s (15H, 5 NAc), 2.689 (m, 1H, H-3_{eq} Neu5Ac), 4.135 (m, 1H, H-2 Man), 4.214 (m, 1H, H-2 Man), 4.273 (m, 1H, H-2 Man), 4.482 (m, 2H, H-1 Gal), 4.623 (m, 3H, H-1 GlcNAc), 4.793 (s, 1H, H-1 Man), 4.952 s, 4.970 s (1H, H-1 Man), 5.142 (d, $J_{1,2}$ = 9.58, 1H, H-1 GlcNAc-(NHGly)), 5.146 s, 5.156 s (1H, H-1 Man).

HRESIMS: found *m/z* 994.8701, calc. for $C_{75}H_{125}N_7O_{54}$,$[M+2H]^{2+}$: *m/z* 994.8698; found *m/z* 1005.8600, calc. for $[M+H+Na]^{2+}$: *m/z* 1005.8608; found *m/z* 1013.8442, calc. for $[M+H+K]^{2+}$: *m/z* 1013.8477; found *m/z* 1024.8350, calc. for $[M+Na+K]^{2+}$: *m/z* 1024.8387.

(4) 9-OS-Gly-BODIPY

TLC: R_f 0.5 (i-PrOH-AcOEt-H_2O= 1:1:1 v/v)

^{1}H NMR, selected chemical shifts (700 MHz, D_2O) δ: 2.017 (s, 3H, NAc), 2.072 (s, 3H, NAc), 2.076 (s, 3H, NAc), 2.102 (s, 3H, NAc), 2.298 (s, 3H, CH_3 BODIPY), 2.549 (s, 3H, CH_3 of BODIPY), 2.797 (dd, 2H, CH_2 of BODIPY), 3.233 (m, 2H, $COCH_2$ of BODIPY), 4.133 (dd, 1H, H-2 Man), 4.215 (dd, 1H, H-2 Man), 4.271 (dd, 1H, H-2 Man), 4.493 (d, $J_{1,2}$ =7.66, 1H, H-1 Gal), 4.498 (d, $J_{1,2}$ = 7.94, 1H, H-1 Gal), 4.615 (m, 3H, H-1 GlcNAc), 4.790 (s, 1H, H-1 Man), 4.951 (s, 1H, H-1 Man), 5.049 (d, $J_{1,2}$ = 9.7 Hz, 1H, H-1 GlcNAc-(NHGly)), 5.146 (s, 1H, H-1 Man), 6.349 (s, 1H, BODIPY), 6.434 (d, 1H, J = 4.02, BODIPY), 7.107 (d, 1H, J = 4.04, BODIPY), 7.507 (s, 1H, BODIPY).

HRESIMS: found *m/z* 986.3774, calc. for $C_{78}H_{121}BF_2N_8O_{47}$, $[M+2H]^{2+}$: *m/z* 986.3772; found *m/z* 997.3679, calc. for $[M+H+Na]^{2+}$: *m/z* 997.36817; found *m/z* 1005.3520, calc. for $[M+H+K]^{2+}$: *m/z* 1005.3551; found *m/z* 1016.3458, calc. for $[M+Na+K]^{2+}$: *m/z* 1016.3658. Fragmentation: found *m/z* 976.3741, calc. for $[M-HF+2H]^{2+}$ 976.3741.

(5) 10-OS-Gly-BODIPY

TLC: R_f 0.2 (i-PrOH – AcOEt – H_2O= 4:3:2 v/v).

^{1}H NMR, selected chemical shifts (700 MHz, D_2O) δ: 1.738 (t, $J_{3ax,3eq}$= $J_{3ax,4}$ = 11.86, 1H, H-3$_{ax}$ Neu5Ac), 2.013 s, 2.052 s, 2.070 s, 2.072 s, 2.088 s, 2.090 s, 2.100 s (15 H, 5 NAc), 2.306 (s, 3H, CH_3 of BODIPY), 2.553 (s, 3H, CH_3 of BODIPY), 2.694 (m, 1H, H-3$_{eq}$ Neu5Ac), 2.798 (m, 2H, CH_2 of BODIPY), 3.237 (m, 2H, $COCH_2$ of BODIPY), 4.132 (s, 1H, H-2 Man), 4.214 (s, 1H, H-2 Man), 4.272 (s, 1H, H-2 Man), 4.476 (m, 2H, H-1 Gal), 4.611 (m, 3H, H-1 GlcNAc), 4.793 (s, 1H, H-1 Man), 4.951 s, 4.966 s (1H, H-1 Man), 5.045 (d, $J_{1,2}$ = 9.77, 1H, H-1 GlcNAc-(NHGly)), 5.145 s, 5.159 s (1H, H-1 Man), 6.356 (s, 1H, BODIPY), 6.439 (d, 1H, *J* = 3.67, BODIPY), 7.117 (d, 1H, *J* = 3.75, BODIPY), 7.523 (s, 1H, BODIPY).

HRESIMS: found *m/z* 1131.9253, calc. for $C_{89}H_{138}BF_2N_9O_{55}$, $[M+2H]^{2+}$: *m/z* 1131.9249; found *m/z* 1143.4172, calc. for $[M+H+Na]^{2+}$: *m/z* 1143.4170; found *m/z* 1150.9004, calc. for $[M+H+K]^{2+}$: *m/z* 1150.9029; found *m/z* 1161.8916, calc. for $[M+Na+K]^{2+}$: *m/z* 1161.8939; found *m/z* 1169.8775, calc. for $[M+2K]^{2+}$: *m/z* 1169.8808.

Validation – IgG glycan analysis by HILIC-UPLC

Fluorescently labeled *N*-glycans were separated by HILIC on an Acquity H-class UPLC instrument (Waters) consisting of a quaternary solvent manager, sample manager and a FLR fluorescence detector, with excitation and emission wavelengths set to 250 and 428 nm, respectively. The instrument was under the control of Empower 3 software, build 3471 (Waters). IgG *N*-glycans were separated on Waters BEH Glycan chromatography column, 100 × 2.1 mm i.d., 1.7 µm BEH particles. Solvent A was 100 mM ammonium formate, pH 4.4; and solvent B was acetonitrile. Sample temperature was 10 °C and column temperature during analysis 60 °C. For separation linear gradient of 25-38 % solvent A at flow rate of 0.40 mL min^{-1} in a 27 min analytical run was used. Solvent composition was then changed to 100 % solvent A in the next minute and column was washed for 2 minutes. After that, solvent composition was changed to initial conditions (25 % solvent A, 75 % solvent B) and system was washed for 5 minutes in these conditions before the next injection. Total analysis time for each sample was 36 minutes.

Data was processed using an automatic processing method created in the Empower software with a traditional integration algorithm, and chromatograms were manually corrected to maintain the same intervals of integration for all the samples. The IgG *N*-glycans were separated into 24 peaks. Glycan structures in each peak have been assigned according to results obtained by exoglycosidases digestion and MS glycan analysis [20].

Supplementary References

1. Brew, K., Vanaman, T. C. & Hill, R. L. The role of alpha-lactalbumin and the A protein in lactose synthetase: a unique mechanism for the control of a biological reaction. *Proc. Natl. Acad. Sci. U. S. A.* **59,** 491–7 (1968).

2. Schachter, H. Biosynthetic controls that determine the branching and microheterogeneity of protein-bound oligosaccharides. *Biochem. Cell Biol.* **64,** 163–81 (1986).

3. Allen, S. D., Tsai, D. & Schachter, H. Control of glycoprotein synthesis. The in vitro synthesis by hen oviduct membrane preparations of hybrid asparagine-linked oligosaccharides containing 5 mannose residues. *J Biol Chem* **259,** 6984–6990 (1984).

4. Bendiak, B. & Schachter, H. Control of glycoprotein synthesis. Kinetic mechanism, substrate specificity, and inhibition characteristics of UDP-N-acetylglucosamine:alpha-D-mannoside beta 1-2 N-acetylglucosaminyltransferase II from rat liver. *J. Biol. Chem.* **262,** 5784–90 (1987).

5. Brockhausen, I., Carver, J. P. & Schachter, H. Control of glycoprotein synthesis. The use of oligosaccharide substrates and HPLC to study the sequential pathway for N-acetylglucosaminyltransferases I, II, III, IV, V, and VI in the biosynthesis of highly branched N-glycans by hen oviduct membranes. *Biochem. Cell Biol.* **66,** 1134–51 (1988).

6. Gleeson, P. a. & Schachter, H. Control of glycoprotein synthesis. *J. Biol. Chem.* **258,** 6162–6173 (1983).

7. Schachter, H., Narasimhan, S., Gleeson, P. & Vella, G. Control of branching during the biosynthesis of asparagine-linked oligosaccharides. *Can. J. Biochem. Cell Biol.* **61,** 1049–66 (1983).

8. Wilson, J. R., Williams, D. & Schachter, H. The control of glycoprotein synthesis: N-acetylglucosamine linkage to a mannose residue as a signal for the attachment of L-fucose to the asparagine-linked N-acetylglucosamine residue of glycopeptide from alpha1-acid glycoprotein. *Biochem. Biophys. Res. Commun.* **72,** 909–16 (1976).

9. Longmore, G. D. & Schachter, H. Product-identification and substrate-specificity studies of the GDP-L-fucose:2-acetamido-2-deoxy-beta-D-glucoside (FUC goes to Asn-linked GlcNAc) 6-alpha-L-fucosyltransferase in a Golgi-rich fraction from porcine liver. *Carbohydr. Res.* **100,** 365–92 (1982).

10. Voynow, J. A., Kaiser, R. S., Scanlin, T. F. & Glick, M. C. Purification and characterization of GDP-L-fucose-N-acetyl beta-D-glucosaminide alpha 1----6fucosyltransferase from cultured human skin fibroblasts. Requirement of a specific biantennary oligosaccharide as substrate. *J. Biol. Chem.* **266,** 21572–7 (1991).

11. Kamińska, J., Glick, M. C. & Kościelak, J. Purification and characterization of GDP-L-Fuc: N-acetyl beta-D-glucosaminide alpha1-->6fucosyltransferase from human blood platelets. *Glycoconj. J.* **15,** 783–8 (1998).

12. Paschinger, K., Staudacher, E., Stemmer, U., Fabini, G. & Wilson, I. B. H. Fucosyltransferase substrate specificity and the order of fucosylation in invertebrates. *Glycobiology* **15,** 463–474 (2004).

13. Weinstein, J., de Souza-e-Silva, U. & Paulson, J. C. Sialylation of glycoprotein oligosaccharides N-linked to asparagine. Enzymatic characterization of a Gal beta 1 to 3(4)GlcNAc alpha 2 to 3 sialyltransferase and a Gal beta 1 to 4GlcNAc alpha 2 to 6 sialyltransferase from rat liver. *J. Biol. Chem.* **257,** 13845–53 (1982).

14. Kent, W. J. *et al.* The human genome browser at UCSC. *Genome Res.* **12,** 996–1006 (2002).

15. Arnold, M., Raffler, J., Pfeufer, A., Suhre, K. & Kastenmüller, G. SNiPA: an interactive, genetic variant-centered annotation browser. *Bioinformatics* **31,** 1334–6 (2015).

16. Tuzikov, A. B., Gambaryan, A. S., Juneja, L. R. & Bovin, N. V. Conversion of Complex Sialooligosaccharides into Polymeric Conjugates and their Anti-Influenza Virus Inhibitory Potency. *J. Carbohydr. Chem.* **19,** 1191–1200 (2000).

17. Mochalova, L. V. *et al.* Fluorescent assay for studying the substrate specificity of neuraminidase. *Anal. Biochem.* **341,** 190–193 (2005).

18. Mochalova, L. *et al.* Oligosaccharide specificity of influenza H1N1 virus neuraminidases. *Arch. Virol.* **152,** 2047–57 (2007).

19. Ovchinnikova, Tatiana V. Pshezhetsky, Alexey V. Tuzikov, A. B. & Bovin, N. V. Synthesis of 1-BODIPY-labeled 2-amino-2-deoxy-d-glucose, substrate for acetyl-CoA:glucosaminide N-acetyltransferase. *Mendeleev Commun.* **25,** 422–423 (2015).

20. Pučić, M. *et al.* High Throughput Isolation and Glycosylation Analysis of IgG-Variability and Heritability of the IgG Glycome in Three Isolated Human Populations. *Mol. Cell. Proteomics* **10,** M111.010090-M111.010090 (2011).
