# Supplementary material for: Network inference from glycoproteomics data reveals new reactions in the IgG glycosylation pathway
Source: Nat Commun. 2017 Nov 14;8:1483. doi: 10.1038/s41467-017-01525-0 (PMC5684356; doi:10.1038/s41467-017-01525-0)
Supplement: Supplementary file 3 — Description of Additional Supplementary Files [file 41467_2017_1525_MOESM3_ESM.pdf]

Description of Supplementary Files

File name: Supplementary Data 1
Description: Full list of Pearson and partial correlation coefficients computed for the four Croatian cohorts

File name: Supplementary Data 2
Description: Fisher's exact test p-values for all rule combination in the four Croatian cohorts

File name: Supplementary Data 3
Description: Detailed results of the GWAS analysis for the four IgG glycosyltranferases

File name: Supplementary Data 4
Description: Enzymatic assays for B4GalT1 and MGAT3

File name: Supplementary Software
Description: GGMs for the four Croatian cohorts
